# Elongated and substituted triazine-based tricarboxylic acid linkers for MOFs

**DOI:** 10.3762/bjoc.12.219

**Published:** 2016-10-27

**Authors:** Arne Klinkebiel, Ole Beyer, Barbara Malawko, Ulrich Lüning

**Affiliations:** 1 Otto-Diels-Institut für Organische Chemie, Christian-Albrechts-Universität zu Kiel, Olshausenstr. 40, D-24098 Kiel, Germany

**Keywords:** isoreticular, linker, MOF, Suzuki coupling, triazine

## Abstract

New triazine-based tricarboxylic acid linkers were prepared as elongated relatives of triazinetribenzoic acid (TATB). Additionally, functional groups (NO_2_, NH_2_, OMe, OH) were introduced for potential post-synthetic modification (PSM) of MOFs. Functionalized tris(4-bromoaryl)triazine “cores” (**3a**,**3b**) were obtained by unsymmetric trimerization mixing one equivalent of an acid chloride (OMe or NO_2_ substituted) with two equivalents of an unsubstituted nitrile. Triple Suzuki coupling of the cores **3** with suitable phenyl- and biphenylboronic acid derivatives provided elongated tricarboxylic acid linkers as carboxylic acids **17** and **20** or their esters **16** and **19**. Reduction of the nitro group and cleavage of the methoxy group gave the respective amino and hydroxy-substituted triazine linkers.

## Introduction

A typical building block for many metal–organic frameworks (MOFs) [1–4] carries two functional groups such as carboxylic acids or heterocycles resulting in a bridging ligand for the metal-containing „edges“ of the porous structures. Linkers with more than two ligating sites (carboxylic acids or heterocycles) have also been used successfully. Tri- to hexatopic linkers are also known. The prototype for a trivalent linker is 1,3,5-benzenetricarboxylic acid. By elongating the „arms“ of the linkers, larger pores can be achieved, and often the MOF structures containing extended linkers resemble those obtained with the smaller parent linker (isoreticular structures, see for instance [5]).

**Figure 1:**
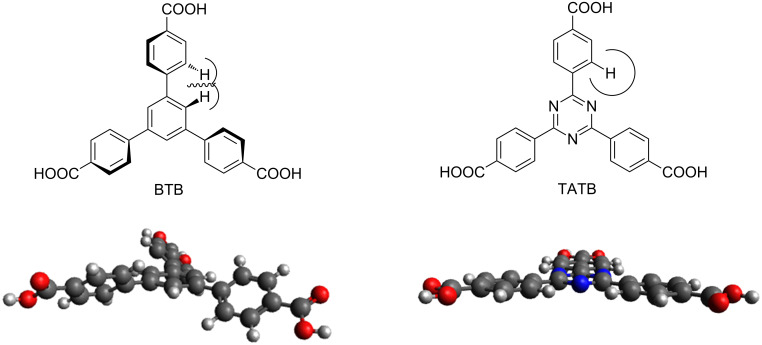
Steric repulsion between *ortho*-hydrogen atoms in benzene-1,3,5-tribenzoic acid (BTB) leads to a non-planar structure (left). The exchange of CH units by nitrogen atoms in the central six-membered ring allows a planar structure: 1,3,5-triazine-2,4,6-tribenzoic acid (TATB, right). The two structures in the lower half have been calculated with B3LYP//6-31G*.

In the case of the tritopic linker 1,3,5-benzenetricarboxylic acid (BTC, forming HKUST-1 [6] in the presence of copper ions), an obvious elongated building block is BTB (1,3,5-benzenetribenzoate, Figure 1, left) forming for instance MOF-14 [7]. But this type of elongation has a drawback: the direct vicinity of the aryl groups does not allow planarity due to the repulsion of the *ortho*-hydrogen atoms in the biphenyl subunits. An exchange of the central benzene ring by a 1,3,5-triazine (1,3,5-triazine-2,4,6-tribenzoate, TATB, Figure 1, right), however, permits planarity. The respective MOF from TATB and copper is PCN-6 [8]. While the orientation of the carboxylate plane relative to the central six-membered ring is in the same plane in the TATB based MOF PCN-6, the BTB analogue MOF-14 shows a tilt angle of ca. 35°. B3LYP//6-31G* calculations of the isolated linkers in the gas phase result in dihedral angles of 38.5° for BTB and 0.006° for TATB, respectively (for further information see Supporting Information File 1). A detailed comparison of triarylbenzene and triaryl-1,3,5-triazine based MOFs and a discussion on the differences in sterical hindrance in benzene and 1,3,5-triazine based structures has been undertaken for tetrazol terminated linkers [9].

The properties of MOFs are determined by the nature of the linker, the nature of the metal-containing secondary building unit (SBU) and of course by the pore size. Additional properties are expected if other functional groups were present in the pores. There are two general strategies to introduce additional functional groups into a MOF: (i) by post-synthetic modification, i.e., a reaction of an assembled MOF with some added reagent, or (ii) by the use of modified linkers in the synthesis of the MOF hoping that functionalized linkers lead to isoreticular structures.

It is obvious that the chance for isoreticular structures decreases with the extend with which the linker is altered. Therefore, in the case of tritopic linkers, it makes a difference whether one additional functional group is introduced per linker or one per „arm“. Also the issue of planarity discussed above is related to the amount of additional substituents in the linker.

**Figure 2:**
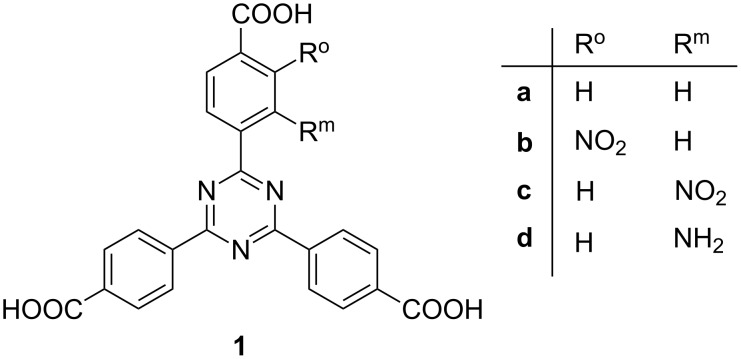
Mono-substituted TATB linkers **1b–d** were successfully employed in the isoreticular syntheses of PCN-6 MOFs [10].

Indeed, when TATB **1a** was substituted with only one substituent per linker, syntheses of isoreticular analogues of MOFs were possible. Structures which are isoreticular to PCN-6 could be synthesized with nitro and amino-substituted TATBs **1b–d** [10] (Figure 2).

**Figure 3:**
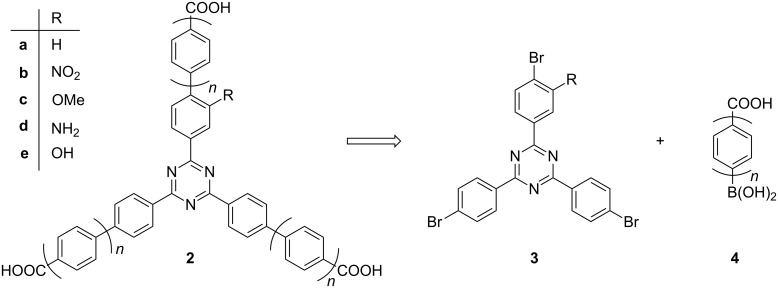
Retrosynthetic analysis for extended TATBs **2**: triple Suzuki coupling between tribromotriazines **3** and boronic acids **4**.

How can this strategy be extended to even larger monofunctionalized triazine linkers? Obviously by the introduction of an additional benzene ring into each arm. Since some decades, palladium-catalyzed cross coupling is the method of choice when aryl–aryl bonds should be constructed, for instance by applying the Suzuki–Miyaura coupling [11]. A retrosynthetic analysis (Figure 3) of extended mono-functionalized triazinetricarboxylic acids **2** calls for tris(4-bromoaryl)-1,3,5-triazines **3** and boronic acids **4** with an additional carboxylic acid functionality. The respective methyl carboxylate of boronic acid **4** with *n* = 1, i.e. **15** (see below, Figure 7), is commercially available. In case of the elongated methyl carboxylate of **4** with *n* = 2, the pinacol boronate **18** (Figure 8) has been described in the literature [12].

## Results and Discussion

**Figure 4:**
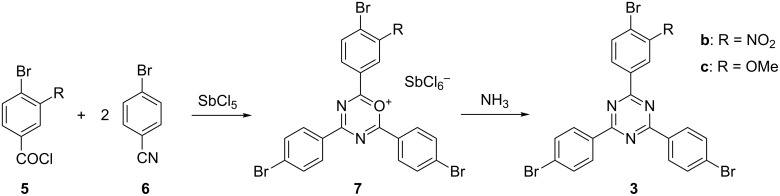
Synthesis of unsymmetrically substituted 2,4,6-tris(bromoaryl)-1,3,5-triazines **3** from one equivalent of a substituted benzoyl chloride **5** and two equivalents of benzonitrile **6**. As intermediates, oxadiazinium salts **7** are formed. The reaction with ammonia yields the desired triazines **3** (overall yields: 53% (**3b**), 61% (**3c**).

Symmetric 1,3,5-triazines such as **3a** are usually synthesized by trimerization of respective nitriles [13]. Unsymmetric 1,3,5-triazines **3** can be made by combining one equivalent of an acid chloride with two equivalents of a nitrile [14–16] (for a recently described alternative access to unsymmetrical 1,3,5-triazines; see [17]). In the presence of a suitable Lewis acid such as antimony(V) chloride, the acid chloride condenses with the nitriles to form an oxadiazinium salt from which the triazine can be obtained by reaction with ammonia (Figure 4).

The respective syntheses have successfully been carried out with nitro and methoxy-substituted benzoyl chlorides **5b** and **5c**. The resulting triazines **3b** and **3c** can then be coupled to boronic acids or boronates to give substituted and elongated triazine-based linkers. Furthermore, the reduction of the nitro group or cleavage of the methoxy group will give additional substituted linkers, amino and hydroxy-substituted ones (see below).

The syntheses of larger linkers **2** therefore need: (i) substituted 4-bromobenzoic acid chlorides **5**, 4-bromobenzonitrile (**6**) and the methoxycarbonyl-substituted phenylboronic acid **15** (*n* = 1) or pinacol biphenylboronate **18** (*n* = 2).

### Syntheses

Four functional groups were chosen as additional substituents in the extended triazine linkers **2**: nitro, methoxy, amino and hydroxy groups. An amino group can be obtained from a nitro group by reduction, and a hydroxy group by cleavage of a methoxy group. Therefore, it was sufficient to synthesize the nitro and methoxy-substituted acid chlorides **5** for the unsymmetric trimerization of the desired tribromotriazines **3**.

**Figure 5:**
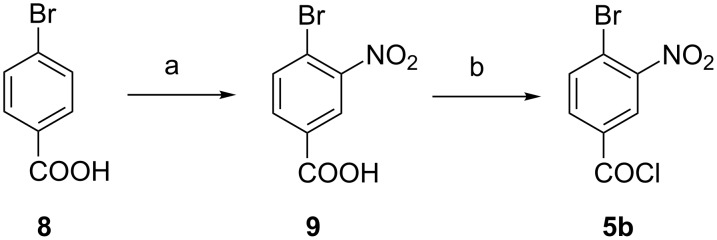
Synthesis of 4-bromo-3-nitrobenzoyl chloride (**5b**). Conditions: a) HNO_3_/H_2_SO_4_, 3 h 0 °C, 2 h, room temperature, 98%, b) SOCl_2_, 2 h reflux, not isolated.

The synthesis of 4-bromo-3-nitrobenzoyl chloride (**5b**) was straightforward. Commercially available 4-bromobenzoic acid (**8**) was nitrated according to a known procedure [18]. The reaction with thionyl chloride provided then acid chloride **5b** (Figure 5).

**Figure 6:**
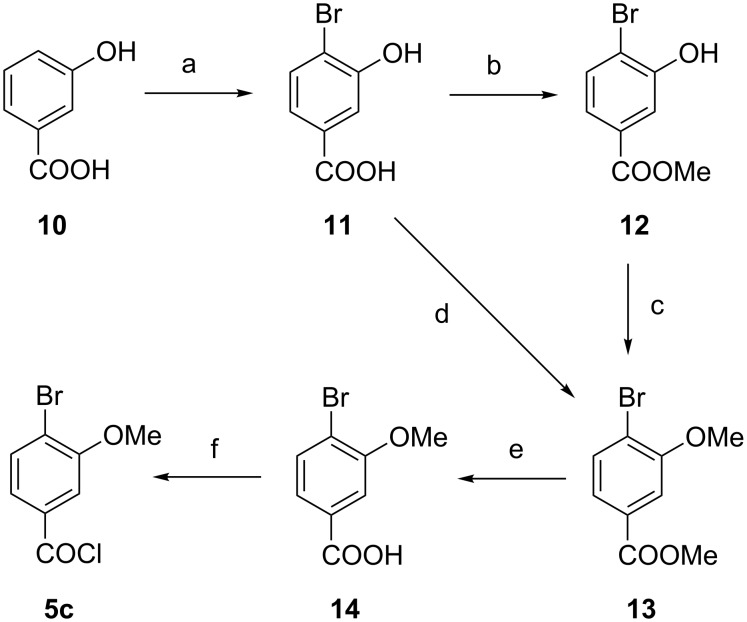
Syntheses of 4-bromo-3-methoxybenzoyl chloride (**5c**). Conditions: a) Br_2_, EtOH/HOAc, 30 min, room temperature, 52%, b) MeOH/H_2_SO_4_, MeOH, 16 h, reflux, 94%, c) Me_2_SO_4_, K_2_CO_3_, acetone, 3 h, reflux, quant., d) MeI, K_2_CO_3_, DMF, 16 h, room temperature, 83%, e) NaOH, H_2_O, MeOH, 6 h, room temperature, 66%, f) SOCl_2_, DMF (cat.), 2 h, reflux, not isolated.

In order to synthesize the respective methoxy compound **5c**, commercially available 3-hydroxybenzoic acid (**10**) was used as starting material (Figure 6). Bromination introduced a bromine atom into the 4 position (**11**) [19], then the phenol was turned into its methyl ether. This methylation can be performed with the free carboxylic acid **11** [20] or with ester **12** [21–22]. In order to generate the methoxy-substituted benzoyl chloride **5c**, methoxy ester **13** was cleaved first [23] and the resulting acid **14** was reacted with thionyl chloride. All steps were known from the literature and have been carried out on a multigram scale.

Cyclotrimerization of one equivalent of an acid chloride **5b** or **5c** with two equivalents of 4-bromobenzonitrile (**6**) was achieved using the Lewis acid antimony(V) chloride as depicted in Figure 4. The resulting colourful oxadiazinium salts **7** were mixed with aqueous ammonia solution resulting in the formation of the desired triazines **3b** and **3c** in 53 and 61% yield, respectively.

**Figure 7:**
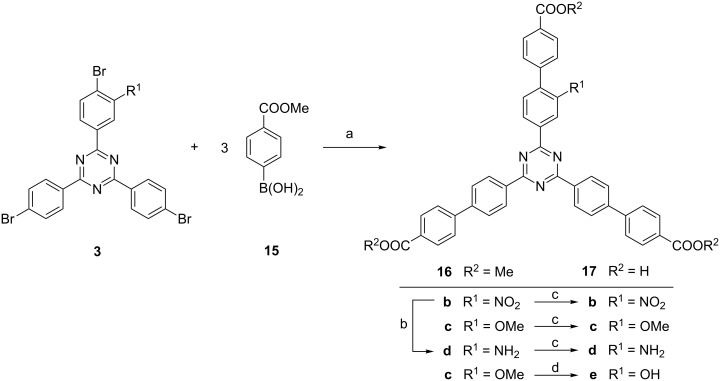
Triple Suzuki–Miyaura coupling between tribromotriazines **3** and boronic acid **15** and subsequent hydrolyses of the esters **16**. Conditions: a) Pd(PPh_3_)_4_, K_3_PO_4_, dioxane, H_2_O, 48 h, reflux, **16b**: 90%, **16c**: 73%, b) Pd/C, H_2_, 5 bar, CH_2_Cl_2_, 5 d, room temperature, **16d**: 77%. c) **17b**: LiOH/H_2_O/dioxane, 48 h, room temp, 96%; **17c**: LiOH/H_2_O/THF, 24 h, 60 °C, quant.; **17d**: H_2_O/THF, 24 h, 60 °C, quant., d) pyridinium hydrochloride, 12 h, 200 °C, **17e**: 52%.

Following the synthetic plan of Figure 3, the tribromides **3b** and **3c** were used in the Suzuki couplings. In this and respective following synthetic steps, optimization of the reaction conditions was very important because each of the reactions had to be performed three times with each triazine. Thus the total yield of the final product depends on the yield of the single step to the power of three. In a triple Suzuki coupling, 4-(methoxycarbonyl)phenylboronic acid (**15**) as the coupling partner yielded elongated and monofunctionalized 1,3,5-triazines **16b** and **16c** carrying methyl ester groups at the end of each „arm“ (Figure 7). The yields of 90% (**16b**) and 73% (**16c**) correspond to 97% and 90% yield per coupling step, respectively.

By selective reduction, the nitro-substituted triester **16b** could be transferred into its amino derivative. The reaction time and the hydrogen pressure had to be optimized. By heterogeneous catalytic hydrogenation at 5 bar with a Pd/C catalyst, aminotriazine **16d** was obtained in 77% yield after 5 days. Hydrolyses of all three methyl esters **16b–d** provided the tricarboxylic acids **17b–d** in 96% to quantitative yields. Also the methoxy group could be cleaved to yield a hydroxy-substituted elongated triazine. In this case, four methoxy, three ester and one ether group had to be cleaved leading to increasingly less soluble products (carboxylic acids are less soluble than the respective esters). In order to improve the yield, a methoxy cleaving procedure which has been found to be effective in other demanding demethylations [24] was used. In liquid pyridinium chloride (which melts at 144 °C), apparently all intermediates were sufficiently soluble and consequently all four methoxy groups of **16c** could be cleaved in a one-pot reaction. The tetrafold cleavage yielded hydroxytriazine **17e** in 52% yield. All tricarboxylic acids **17** are considerably less soluble in most solvents when compared to the respective methyl esters **16**. Nevertheless, they are sufficiently soluble in, for instance, DMSO or in base to allow proper analyses and future use in solvothermal syntheses of MOFs.

Tribromotriazines **3** are not only good starting materials for the syntheses of biphenyl-substituted triazine linkers **16** and **17**. Also longer terphenyl-substituted linkers can be obtained if the same approach is chosen but a biphenylboronic acid derivative such as boronate **18** [12] is used instead of the phenyl compound **15**.

**Figure 8:**
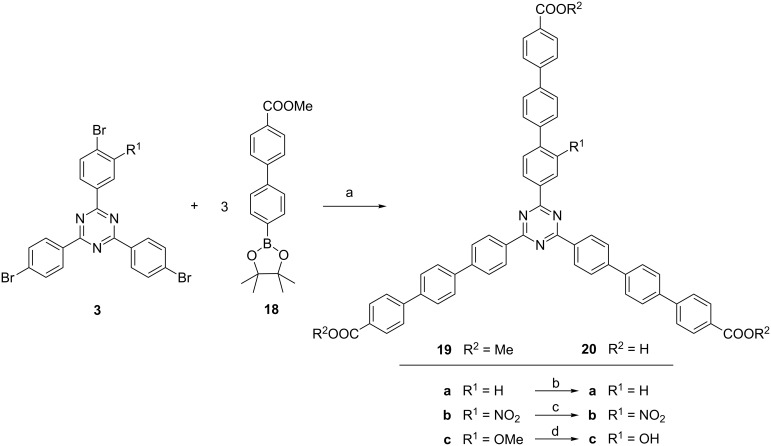
Triple Suzuki coupling between tribromotriazines **3** and boronate **18**. Conditions: a) **19a**: Pd(PPh_3_)_4_, K_3_PO_4_, dioxane/H_2_O, 2 d, 120 °C, 24%; **19b**: Pd(dppf)Cl_2_, K_3_PO_4_, dioxane/H_2_O, 3 d, 120 °C, 16%; **19c**: Pd(PPh_3_)_4_, K_3_PO_4_, dioxane/H_2_O, 2 d, 100 °C, 81%; b) LiOH/H_2_O/THF, 24 h, 120 °C, **20a**: 99%; c) NaOH/H_2_O/dioxane, 24 h, 120 °C, **20b**: 79%; d) pyridinium hydrochloride, 12 h, 200 °C, **20e**: 62%.

As in the case of the biphenyl derivatives **16** and **17**, the nitro and the methoxy-substituted triazines **3b** and **3c** were used to obtain the terphenyl-based nitro and methoxy-substituted triesters **19b** and **19c** (Figure 8). But also the non-substituted precursor **3a** was coupled leading to the unsubstituted parent compound **19a**, a triester which has not been described yet. Direct hydrolyses of triesters **19a** and **19b** gave the carboxylic acids **20a** and **20b**.

Finally, a hydroxy-functionalized terphenyl-based linker was synthesized from the methoxy-substituted triester **19c**. As successfully applied for **16c**, molten pyridinium chloride was used as the cleaving reagent and all four methoxy groups of **19c** could be cleaved leading to the hydroxy-substituted triacid **20e** in 62% yield.

All three triacids **20a, b** and **e** precipitate from water with decreasing pH. In DMSO, they are soluble and consequently, NMR analytics have been carried out in DMSO-*d*_6._

## Conclusion

In conclusion, 13 new elongated relatives of TATB **16**, **17**, **19** and **20** have been synthesized in batch sizes up to several grams, of which 11 were mono-substituted. Syntheses, purifications and solubilities are satisfying in the case of the biphenyl derivatives **16** and **17**. The handling of the terphenyl derivatives **19**, and especially **20**, becomes more difficult due to a lower solubility. If the solublities were not sufficient for the solvothermal syntheses of MOFs, “discrete” methylation [25] could find a remedy.

## Supporting Information

File 1 Experimental details.
