# Supplementary material for: Elongated and substituted triazine-based tricarboxylic acid linkers for MOFs
Source: Beilstein J Org Chem. 2016 Oct 27;12:2267–73. doi: 10.3762/bjoc.12.219 (PMC5238681; doi:10.3762/bjoc.12.219)
Supplement: File 1 — Experimental details. [file Beilstein_J_Org_Chem-12-2267-s001.pdf]

Supporting information

for

Elongated and substituted triazine-based tricarboxylic acid linkers for MOFs

Arne Klinkebiel, Ole Beyer, Barbara Malawko and Ulrich Lüning*

Address: Otto-Diels-Institut für Organische Chemie, Christian-Albrechts-Universität zu Kiel, Olshausenstr. 40, D-24098 Kiel, Germany

*Corresponding author

Email: Ulrich Lüning - luening@oc.uni-kiel.de

Experimental details

General remarks: Antimony(V) chloride (99%, Alfa Aesar), [1,1′-bis(diphenylphosphino)ferrocene]dichloro palladium(II) (99.9%, ABCR), 4-bromobenzoic acid (98 %, Sigma-Aldrich), 4-bromobenzonitrile (99%, Sigma-Aldrich), 3-hydroxybenzoic acid (97%, Sigma-Aldrich), 4-(methoxycarbonyl)phenylboronic acid (97%, ABCR), palladium on charcoal (10%, Alfa Aesar) and tetrakis(triphenylphosphine)palladium(0) (99%, ABCR) were purchased and used without further purification. Methyl 4'-(4,4,5,5-tetramethyl-1,3,2-dioxaborolan-2-yl)-1,1'-biphenyl-4-carboxylate (**18**) was synthesized as described.[1] Dry solvents were obtained with suitable desiccants. Other solvents were distilled before use. Column chromatography was carried out with silica gel (Macherey-Nagel, particle size 0.04–

0.063 mm). Melting points were measured with a Gallenkamp MPD350.BM2.5 instrument. NMR spectra were recorded with a Bruker AC 200, DRX 500 or Avance 600 instrument at 300 K. Assignments are supported by COSY, HSQC, and HMBC. In some cases, not all ^{13}C signals could be found in the 1D ^{13}C NMR spectra. In these cases, the listed chemical shifts have been extracted from the 2D spectra. Even when obtained by DEPT, the type of ^{13}C signal is always listed as singlet, doublet, etc. All chemical shifts are referenced to tetramethylsilane or the residual proton or carbon of the solvent. The NMR signals of atoms in the different arms of the triazines are assigned by using for instance Ar2 for atoms which are part of an benzene ring in the substituent at C-2 of the triazine. Aromatic rings within a biphenyl or terphenyl carry ’ or ’’ according to the biphenyl or terphenyl nomenclature. EI/CI mass spectra were recorded with a Finnigan MAT 8200, MAT 8230 or JEOL AccuTOF GCV 4G the latter one being used for HRMS as well. ESI mass spectra were recorded with an Applied Biosystems Mariner 5280. MALDI-TOF mass spectra were recorded with a Bruker-Daltronics Biflex III with Cl-CCA (4-chloro-α-cyano-cinnamic acid) as matrix. IR spectra were recorded with a Perkin-Elmer Spectrum 100 spectrometer equipped with a Golden Gate Diamond ATR unit A-531-G. Elemental analyses were carried out with a Euro EA 3000 Elemental Analyzer from Euro Vector.

2-(4-Bromo-3-nitrophenyl)-4,6-bis(4-bromophenyl)-1,3,5-triazine (3b)

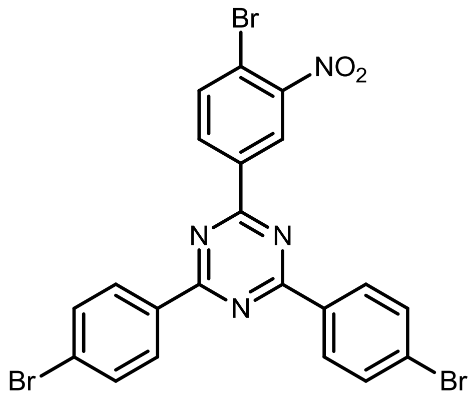

Under nitrogen and at 0 °C, antimony(V) chloride (5.23 mL, 40.8 mmol) was added to a solution of crude 4-bromo-3-nitrobenzoyl chloride (**5b**, 8.86 g, max. 33.5 mmol) and *p*-bromobenzonitrile (**6**, 12.5 g, 68.7 mmol) in dry chloroform (120 mL). The mixture was stirred for 1 h at room temp. and for 16 h at reflux. A yellow solid formed which was filtered off and washed with chloroform. At 0 °C, aq. ammonia (390 mL, 28%) was added in portions to the residue and the suspension was stirred for 2 h at room temp. The solid changed its color from yellow to white. After filtration, the product was separated from insoluble side products by a Soxhlet extraction (chloroform, 48 h). Yield: 10.3 g (17.6 mmol, 53%). M. p.: >300 °C. ^1H NMR (500 MHz, $CDCl_3$, 323 K): δ = 9.11 (d, 1H, ^4J = 1.8 Hz, 2-Ar-*H*-2), 8.74 (dd, 1H, ^3J = 8.4 Hz, ^4J = 1.8 Hz, 2-Ar-*H*-6), 8.58 (d, 4H, ^3J = 8.5 Hz, 4,6-Ar-*H*-2,6), 7.98 (d, 1H, ^3J = 8.4 Hz, 2-Ar-*H*-5), 7.72 (d, 4H, ^3J = 8.5 Hz, 4,6-Ar-*H*-3,5) ppm. ^{13}C NMR (125 MHz, $CDCl_3$, 323 K): δ = 171.7 (s, triazine-*C*-4,6), 169.4 (s, triazine-*C*-2), 150.5 (s, 2-Ar-*C*-3), 136.7 (s, 2-Ar-*C*-4), 135.3 (d, 2-Ar-*C*-5), 134.4 (s, 4,6-Ar-*C*-4), 132.6 (d, 2-Ar-*C*-6), 132.3 (d, 4,6-Ar-*C*-3,5), 130.6 (d, 4,6-Ar-*C*-2,6), 128.4 (s, 4,6-Ar-*C*-1), 125.6 (d, 2-Ar-*C*-2), 118.8 (s, 2-Ar-*C*-1) ppm. MS (EI, 70 eV): *m/z* = 588/590/592/594 (34/100/90/33) $[M]^{+\cdot}$. MS (CI, isobutane): *m/z* = 589/591/593/595 (34/100/87/43) $[M + H]^+$. IR (ATR): $\tilde{\nu}$ = 3082 (aryl-H), 1592, 1509, 1486 (arom. C=C, arom. C=N), 1578 (NO_2), 1350 (C-N-val.), 806 (1,4-disubst. aryl, 1,2,4-trisubst. aryl) cm^{-1}. HRMS (EI): *m/z* = calcd. 589.8411; found 589.8391 (Δ 3.43 ppm). Elemental analysis ($C_{21}H_{11}Br_3N_4O_2$) (591.05): calcd. C 42.67 H 1.88 N 9.48; found C 42.67 H 1.77 N 9.07.

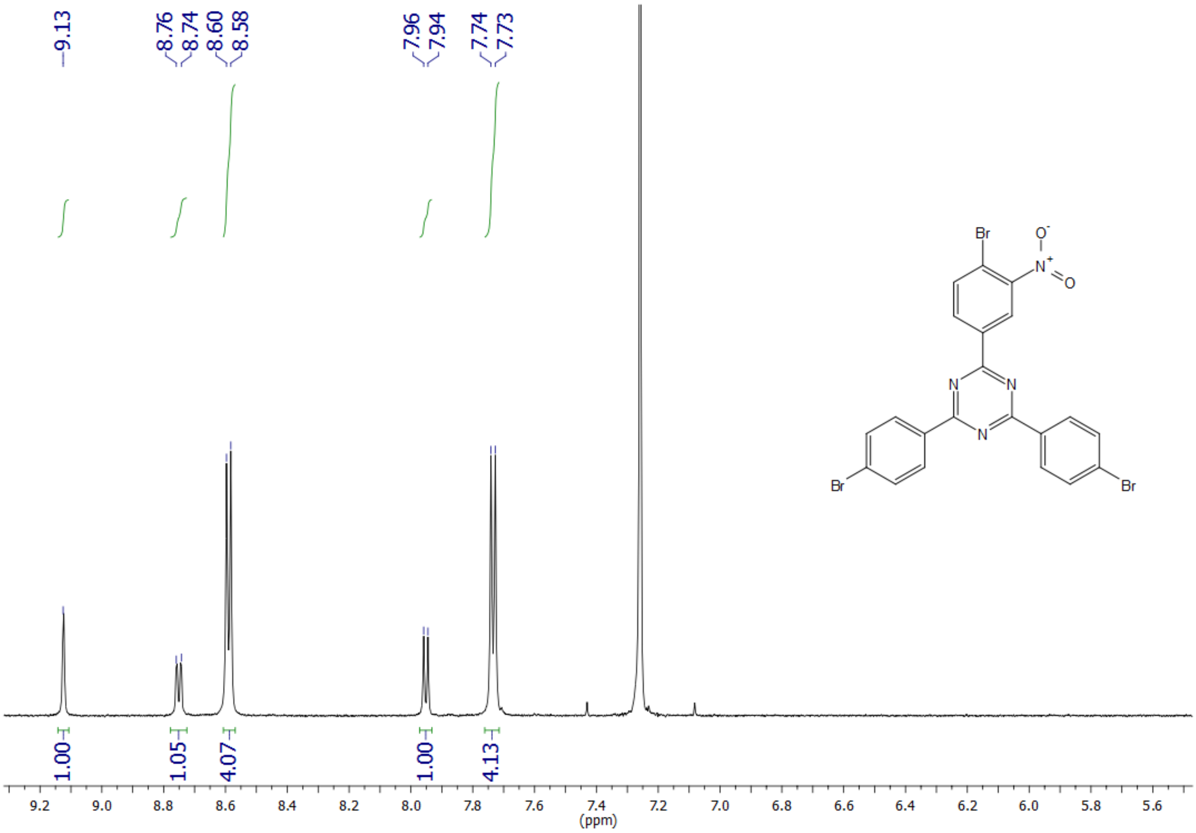
9.13
8.76
8.74
8.60
8.58
7.96
7.94
7.74
7.73
Br
O
N
O
N
N
N
Br
Br
1.00
1.05
4.07
1.00
4.13
9.2
9.0
8.8
8.6
8.4
8.2
8.0
7.8
7.6
7.4
(ppm)
7.2
7.0
6.8
6.6
6.4
6.2
6.0
5.8
5.6

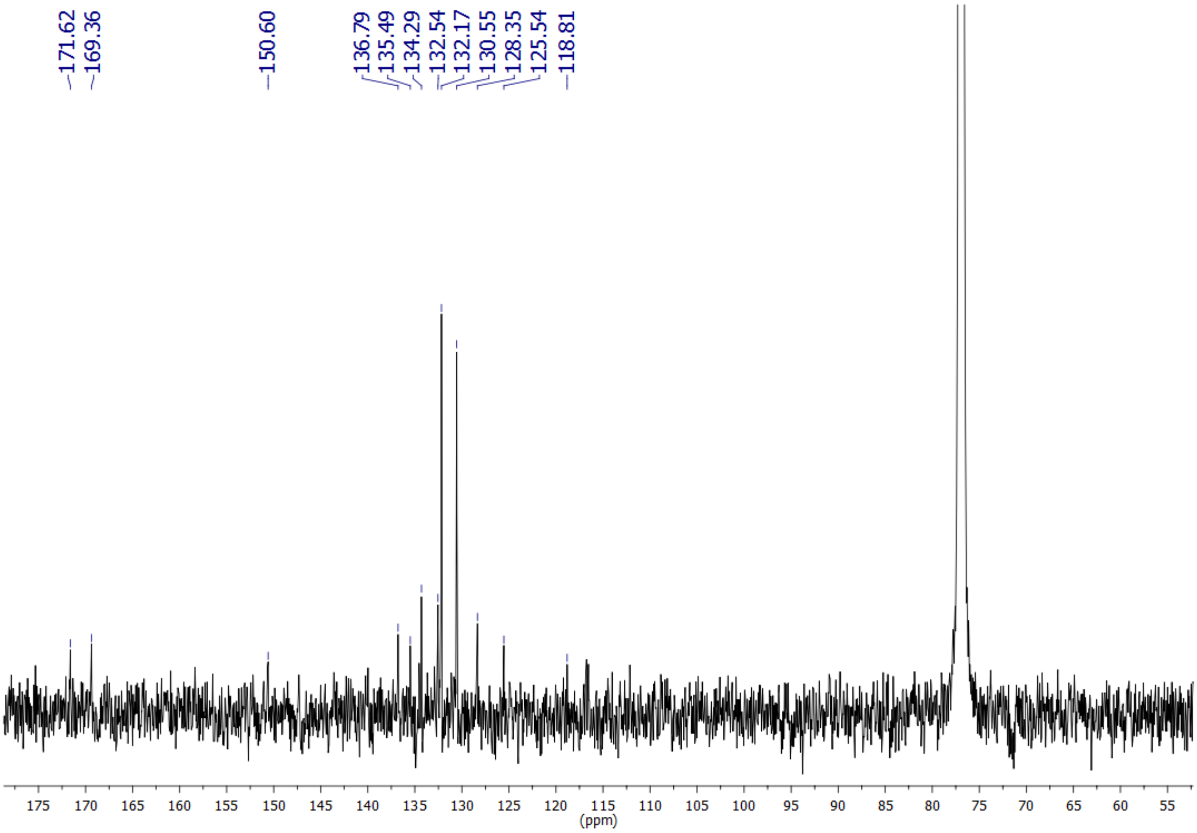
171.62
169.36
150.60
136.79
135.49
134.29
132.54
132.17
130.55
128.35
125.54
118.81
175
170
165
160
155
150
145
140
135
130
125
120
115
(ppm)
110
105
100
95
90
85
80
75
70
65
60
55

2-(4-Bromo-3-methoxyphenyl)-4,6-bis(4-bromophenyl)-1,3,5-triazine (3c)

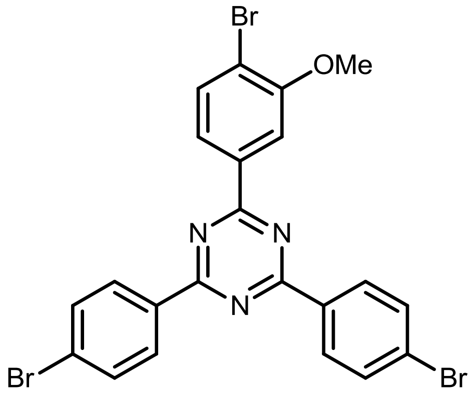

Under nitrogen, 4-bromo-3-methoxy-benzoyl chloride [**5c**, crude, from 1.00 g (4.01 mmol) of **14**] and *p*-bromobenzonitrile (**6**, 1.46 g, 8.02 mmol) were dissolved in dry chloroform (25 mL). At 0 °C, antimony(V) chloride (616 µL, 4.81 mmol) was added and the mixture was stirred for 1 h at room temp. followed by 16 h of reflux. The resulting red oxonium salt was filtered off and washed with chloroform. At 0 °C, aq. ammonia (50 mL, 28%) was added to the residue and the suspension was stirred for 2 h at room temp. The solid changed its color from yellow to white. After filtration, the product was filtered off and was extracted for 24 h with chloroform (Soxhlet). After evaporation of the solvent, the residue was recrystallized from a boiling mixture of chloroform and petrol ether (30–60 °C) yielding a colourless solid. Yield: 1.41 g (2.45 mmol, 61%). M. p.: 203 °C. ^{1}H NMR (500 MHz, $CDCl_3$): δ = 8.57 (m_c (d), 4H, ^{3}J = 8.7 Hz, 4,6-Ar-*H*-2,6), 8.23 (d, 1H, ^{4}J = 1.8 Hz, 2-Ar-*H*-2), 8.21 (dd, 1H, ^{3}J = 8.2 Hz, ^{4}J = 1.8 Hz, 2-Ar-*H*-6), 7.72 (d, 1H, ^{3}J = 8.2 Hz, 2-Ar-*H*-5), 7.70 (m_c (d), 4H, ^{3}J = 8.7 Hz, 4,6-Ar-*H*-3,5), 4.09 (s, 3H, OCH_3) ppm. ^{13}C NMR (125 MHz, $CDCl_3$): δ = 171.1 (s, triazine-*C*-4,6), 171.0 (s, triazine-*C*-2), 156.3 (s, 2-Ar-*C*-3), 136.4 (s, 2-Ar-*C*-1), 134.7 (s, 4,6-Ar-*C*-1), 133.6 (d, 2-Ar-*C*-5), 132.0 (d, 4,6-Ar-*C*-3,5), 130.4 (d, 4,6-Ar-*C*-2,6), 127.8 (s, 4,6-Ar-*C*-4), 122.5 (d, 2-Ar-*C*-6), 117.3 (s, 2-Ar-*C*-4), 111.6 (d, 2-Ar-*C*-2), 56.4 (q, OCH_3) ppm. MS (EI, 70 eV): *m/z* = 572/574/576/578 (34/100/96/34) $[M]^{+\cdot}$, 180 (85) $[M - C_{15}H_{10}Br_2N_2O]^{+}$. IR (ATR): $\tilde{\nu}$ = 3033 (aryl-H), 1598, 1505, 1485 (arom. C=C, arom. C=N), 1414 (C-N-val.), 1251 (aryl-OCH_3), 831 (1,4-disubst. aryl, 1,2,4-trisubst. aryl) cm^{-1}. HRMS (EI): *m/z* = calcd. 578.8625; found 578.8608 (Δ 3.12 ppm).

Elemental analysis ($C_{22}H_{14}Br_3N_3O$) (576.08): calcd. C 45.87 H 2.45 N 7.29; found C 46.09 H 2.46 N 6.94.

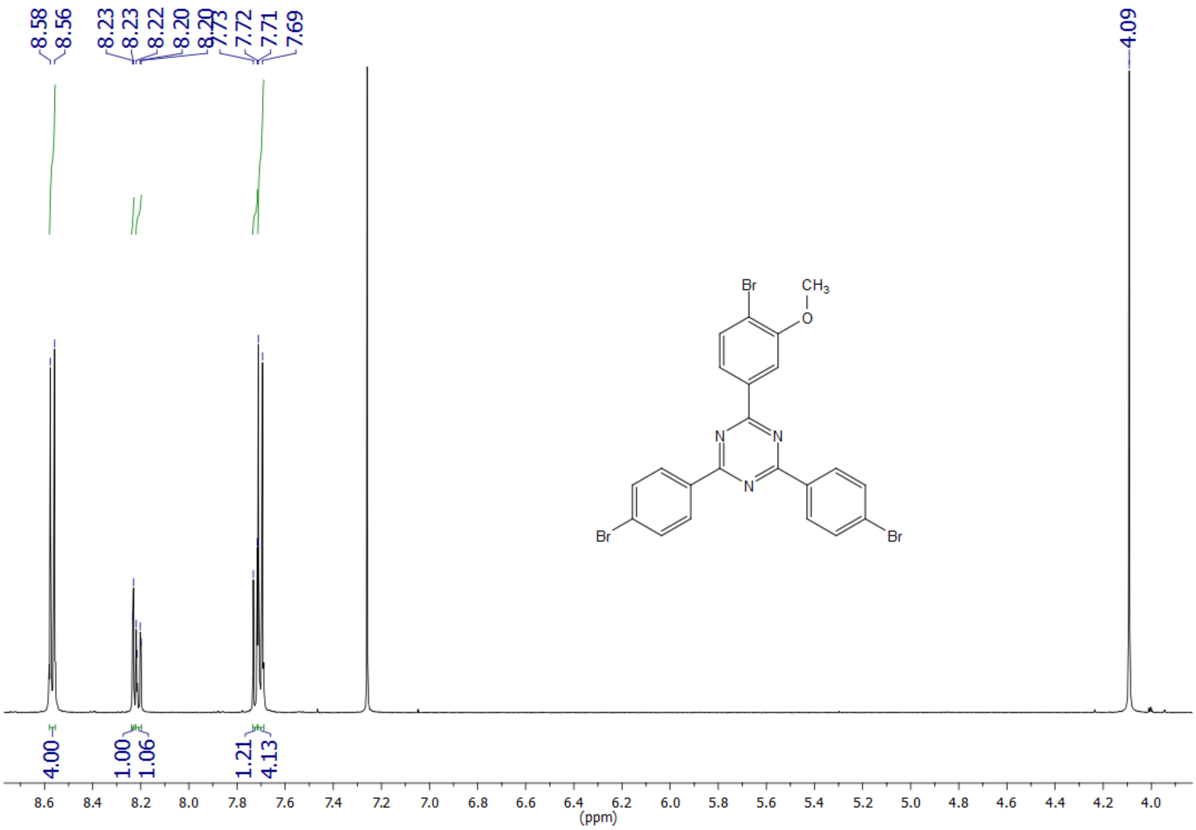

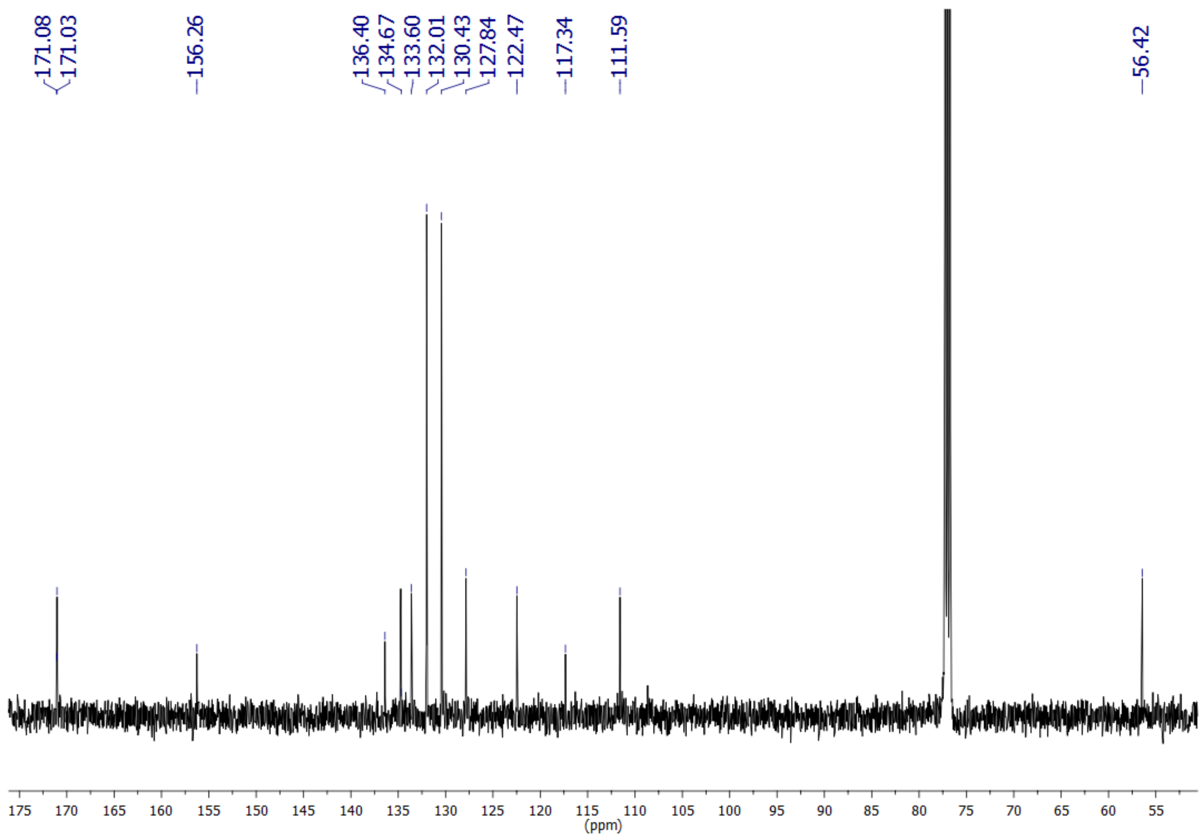
171.08
171.03
156.26
136.40
134.67
133.60
132.01
130.43
127.84
122.47
117.34
111.59
56.42
175
170
165
160
155
150
145
140
135
130
125
120
115
110
105
100
95
90
85
80
75
70
65
60
55
(ppm)

4-Bromo-3-nitrobenzoyl chloride (5b)

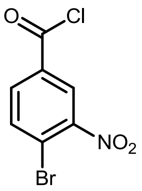

A solution of 4-bromo-3-nitrobenzoic acid (**9**, 4.00 g, 80.0 mmol) in thionyl chloride (24 mL) was heated to reflux for 2 h. Removal of excess thionyl chloride in vacuo yielded a yellow oil which was used without further purification. ^{1}H NMR (500 MHz, $CDCl_3$): δ = 8.54 (d, 1H, ^4J = 2.2 Hz, Ar-*H*-2), 8.13 (dd, 1H, ^3J = 8.5 Hz, ^4J = 2.2 Hz, Ar-*H*-6), 7.94 (d, 1H, ^3J = 8.5 Hz, Ar-*H*-5) ppm. ^{13}C NMR (125 MHz, $CDCl_3$): δ = 166.0 (s, *C*OCl), 150.2 (s, Ar-*C*-3), 136.2 (d, Ar-*C*-6), 134.2 (d, Ar-*C*-5), 133.6 (s, Ar-*C*-1), 127.7 (d, Ar-*C*-2), 122.6 (s, Ar-*C*-4) ppm. MS (EI, 70 eV): *m/z* = 264/262 (14/11) $[M]^{+\cdot}$, 229/227 (96/100) $[M - Cl]^+$. MS (CI, isobutane): *m/z* = 265/263 (100/94) $[M + H]^+$, 229/227 (45/40) $[M - Cl]^+$.

4-Bromo-3-methoxy-benzoyl chloride (5c)

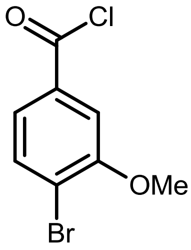

N,*N*-Dimethylformamide (10 µL) was added to a mixture of 4-bromo-3-methoxy-benzoic acid (**14**, 1.00 g, 4.01 mmol) and thionyl chloride (5 mL). After heating to reflux for 2 h, excess thionyl chloride was distilled off and the crude colourless solid was used without further purification. ^{1}H NMR (500 MHz, $CDCl_3$): δ = 7.70 (d, 1H, ^3J = 8.3 Hz, Ar-*H*-5), 7.63 (dd, 1H, ^3J = 8.3 Hz, ^4J = 2.0 Hz, Ar-*H*-6), 7.54 (d, 1H, ^4J = 2.0 Hz, Ar-*H*-2), 3.97 (s, 3H, OCH_3) ppm. ^{13}C NMR (125 MHz, $CDCl_3$): δ = 167.7 (s, *C*OCl), 156.3 (s, Ar-*C*-3), 133.8 (d, Ar-*C*-5), 133.5 (s, Ar-*C*-1), 125.0 (d, Ar-*C*-6), 120.9 (s, Ar-*C*-4), 113.0 (d, Ar-*C*-2), 56.5 (q, OCH_3) ppm.

4-Bromo-3-nitrobenzoic acid (9)

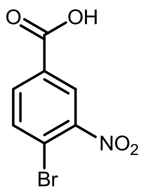

p-Brombenzoic acid (**8**, 5.00 g, 24.9 mmol) was slowly added in portions to a cold mixture (0 °C) of conc. nitric acid (6.2 mL, 0.15 mmol) and conc. sulfuric acid (15.2 mL, 300 mmol). The addition was performed in such a way that the temperature did not exceed 5 °C. After stirring for 3 h at 0 °C and 2 h at room temp., the mixture was poured on ice-containing water. The resulting colourless solid was filtered off and washed extensively with water. Yield: 6.00 g (24.4 mmol, 98%) (ref. [2]: 96%), m. p. 202 °C. ^{1}H NMR (200 MHz, DMSO-d_6): δ = 8.46 – 8.42 (m, 1H, Ar-*H*-2), 8.09–8.05 (m, 2H, Ar-*H*-5,6) ppm. ^{13}C NMR (50 MHz, DMSO-d_6): δ = 165.0 (s, *C*O_2H), 149.7 (s, Ar-*C*-3), 135.4 (d, Ar-*C*-6), 132.4 (d, Ar-*C*-5), 131.7 (s, Ar-*C*-1), 125.9 (d, Ar-*C*-2), 118.0 (Ar-*C*-4) ppm. MS (EI, 70 eV): *m/z* = 245/247 (95/100) $[M]^{+\cdot}$. MS (CI, isobutane): *m/z* = 246/248 (100/99) $[M + H]^{+}$. IR (ATR): $\tilde{\nu}$ = 3082 (aryl-H), 2820 (br., OH), 1684 (C=O), 1595 (arom. C=C), 1530, 1303 (NO_2), 1034 (aryl-Br), 907, 809 (1,2,4-trisubst. aryl) cm^{-1}.

4-Bromo-3-hydroxybenzoic acid (11)

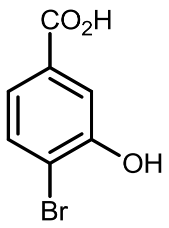

To a solution of 3-hydroxybenzoic acid (**10**, 15.0 g, 109 mmol) in a mixture of ethanol (60 mL) and acetic acid (30 mL), bromine (11.2 mL, 218 mmol) was added slowly and dropwise. After stirring for 30 min at room temp., aq. sodium thiosulfate solution (90 mL) was added and ethanol was removed in vacuo. The remaining aq. layer was extracted with ethyl acetate (5 x 200 mL) and the combined organic layer was dried with magnesium sulfate. After removal of the solvent, the crude product was recrystallized from boiling water and a colourless solid was obtained. Yield: 12.19 g (56.2 mmol, 52%) (ref.[3]:

40%). M. p.: 225 – 227 °C. ^{1}H NMR (500 MHz, DMSO-d_6): δ = 13.04 (br. s, 1H, CO_2H), 10.62 (s, 1H, O*H*), 7.59 (d, 1H, ^3J = 8.3 Hz, Ar-*H*-5), 7.51 (d, 1H, ^4J = 2.0 Hz, Ar-*H*-2), 7.28 (dd, 1H, ^3J = 8.3 Hz, ^4J = 2.0 Hz, Ar-*H*-6) ppm. ^{13}C NMR (125 MHz, DMSO-d_6): δ = 167.2 (s, CO_2H), 154.6 (s, Ar-*C*-3), 133.5 (d, Ar-*C*-5), 131.8 (s, Ar-*C*-1), 121.6 (d, Ar-*C*-6), 117.1 (d, Ar-*C*-2), 115.0 (s, Ar-*C*-4) ppm. MS (EI, 70 eV): *m/z* = 216/218 (100/97) $[M]^{+\cdot}$, 199, 201 (73, 70) $[M – OH]^{+}$.

Methyl 4-bromo-3-hydroxybenzoate (12)

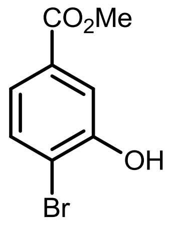

Conc. sulfuric acid (12 mL) was added to a suspension of 4-bromo-3-hydroxybenzoic acid (**11**, 12.2 g, 56.2 mmol) in methanol (120 mL). After heating to reflux for 16 h, the solution was neutralized with sat. aq. sodium bicarbonate solution and methanol was distilled off in vacuo. The remaining aq. phase was extracted with ethyl acetate (3 x 100 mL) and the combined organic layer was dried with magnesium sulfate. Filtration and removal of the solvent in vacuo yielded a colourless solid. Yield: 12.2 g (52.7 mmol, 94%) (ref.[4]: no yield given). M. p.: 124 °C (ref.[4]: 120 – 122 °C). ^{1}H NMR (500 MHz, $CDCl_3$): δ = 7.68 (d, 1H, ^4J = 2.0 Hz, Ar-*H*-2), 7.54 (d, 1H, ^3J = 8.3 Hz, Ar-*H*-5), 7.48 (dd, 1H, ^3J = 8.3 Hz, ^4J = 2.0, Ar-*H*-6), 5.75 (br. s, 1H, O*H*), 3.91 (s, 3H, CO_2CH_3) ppm. ^{13}C NMR (125 MHz, $CDCl_3$): δ = 166.2 (s, CO_2CH_3), 152.4 (s, Ar-*C*-3), 132.2 (d, Ar-*C*-5), 131.3 (s, Ar-*C*-1), 122.7 (d, Ar-*C*-6), 117.1 (d, Ar-*C*-2), 115.6 (s, Ar-*C*-4), 52.4 (q, CO_2CH_3) ppm. MS (EI, 70 eV): *m/z* = 230, 232 (63, 60) $[M]^{+\cdot}$.

Methyl 4-bromo-3-methoxy-benzoate (13)

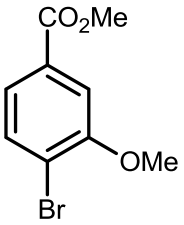

Method A: Potassium carbonate (4.56 g, 33.0 mmol) was added to a solution of methyl 4-bromo-3-hydroxybenzoate (**12**, 6.24 g, 27.0 mmol) in acetone (40 mL). Dimethyl sulfate (3.03 mL, 32.0 mmol) was added and the mixture was heated to reflux for 3 h. At room temp., deionized water was added (5 mL) and acetone was distilled off in vacuo. The remaining aq. phase was extracted with dichloromethane (3 x 100 mL), the combined organic layer was dried with magnesium sulfate, filtered and the solvent was removed in vacuo yielding a colourless solid. Yield: 6.59 g (26.9 mmol, >99%) (ref.[5]: 99%).

Method B: Potassium carbonate (2.54 g, 18.4 mmol) was added to a solution of 4-bromo-3-hydroxybenzoic acid (**11**, 1.00 g, 4.61 mmol) in *N*,*N*-dimethylformamide (10 mL). Methyl iodide (863 µL, 13.8 mmol) was added and the mixture was stirred for 16 h at room temp. Deionized water (50 mL) was added and the aq. phase was extracted with *tert*-butyl methyl ether (3 x 100 mL). The combined organic layer was washed with brine (100 mL) and dried with magnesium sulfate. After filtration, the solvent was evaporated in vacuo yielding a colourless solid. Yield: 938 mg (3.83 mmol, 83%) (ref.[3]: 91%).

M. p. 53 °C. ^{1}H NMR (500 MHz, $CDCl_3$): δ = 7.61 (d, 1H, ^{3}J = 8.2 Hz, Ar-*H*-5), 7.55 (d, 1H, ^{4}J = 1.8 Hz, Ar-*H*-2), 7.51 (dd, 1H, ^{3}J = 8.2 Hz, ^{4}J = 1.8 Hz, Ar-*H*-6), 3.95 (s, 3H, OCH_3), 3.92 (s, 3H, CO_2CH_3) ppm. ^{13}C NMR (125 MHz, $CDCl_3$): δ = 166.4 (s, CO_2Me), 155.9 (s, Ar-*C*-3), 133.3 (d, Ar-*C*-5), 130.6 (s, Ar-*C*-1), 122.9 (d, Ar-*C*-6), 117.5 (s, Ar-*C*-4), 112.4 (d, Ar-*C*-2), 56.4 (q, OCH_3), 52.4 (q, CO_2CH_3) ppm. HRMS (EI): *m/z* = calcd. 243.9735; found 243.9744 (Δ 4.01 ppm).

4-Bromo-3-methoxybenzoic acid (14)

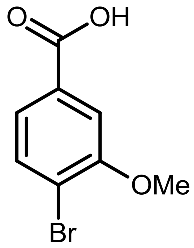

Aq. sodium hydroxide (2 M, 40 mL) was added to a solution of methyl 4-bromo-3-methoxy-benzoate (**13**, 6.52 g, 27.1 mmol) in methanol (200 mL) and the mixture was stirred for 6 h at room temp. Deionized water (100 mL) was added and methanol was distilled off in vacuo. The remaining aq. phase was washed with dichloromethane (3 x 100 mL) and acidified with hydrochloric acid (6 M). The precipitate was filtered off and was washed thoroughly with water yielding a colourless solid. Yield: 4.10 g (17.8 mmol, 66%) (ref[5]: 89%). M. p.: 220 °C. ^{1}H NMR (500 MHz, DMSO-d_6): δ = 13.21 (br. s, 1H, CO_2H), 7.71 (d, 1H, ^3J = 8.2 Hz, Ar-*H*-5), 7.54 (d, 1H, ^4J = 1.8 Hz, Ar-*H*-2), 7.46 (dd, 1H, ^3J = 8.2 Hz, ^4J = 1.8 Hz, Ar-*H*-6), 3.91 (s, 3H, OCH_3) ppm. ^{13}C NMR (125 MHz, DMSO-d_6): δ = 167.1 (s, CO_2H), 155.9 (s, Ar-*C*-3), 133.6 (d, Ar-*C*-5), 132.1 (s, Ar-*C*-1), 123.2 (d, Ar-*C*-6), 116.5 (s, Ar-*C*-4), 113.0 (d, Ar-*C*-2), 56.8 (q, OCH_3) ppm. MS (EI, 70 eV): *m/z* = 230/232 (100/98) $[M]^{+\cdot}$.

2,4-Bis[4'-(methoxycarbonyl)-biphenyl-4-yl]-6-[4'-(methoxycarbonyl)-2-nitro-biphenyl-4-yl)-1,3,5-triazine (16b)

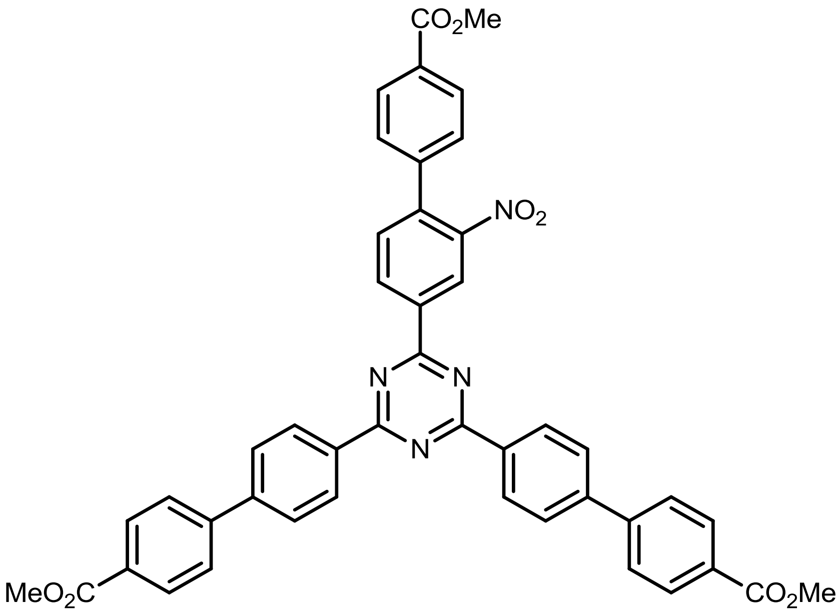

Under nitrogen, 2-(4-bromo-3-nitrophenyl)-4,6-bis(4-bromophenyl)-1,3,5-triazine (**3b**, 3.00 g, 5.08 mmol), 4-methoxycarbonyl-phenylboronic acid (**15**, 4.10 g, 22.8 mmol), potassium phosphate (7.00 g, 33.0 mmol) and tetrakis(triphenylphosphine) palladium(0) (300 mg, 260 µmol) in a mixture of 1,4-dioxane (300 mL) and deionized water (20 mL) were heated to reflux for 48 h. Conversion was checked by TLC (silica gel, cyclohexane/ethyl acetate, 1:1, R_f = 0.69). After evaporation of the 1,4-dioxane in vacuo, chloroform (150 mL) was added and the organic layer was washed with deionized water (2 x 100 mL) and brine (50 mL). The organic layer was dried with magnesium sulfate. After filtration and evaporation of the solvent in vacuo, the crude product was dissolved in chloroform, activated charcoal was added, and after filtration, the product was recrystallized from chloroform yielding a colourless solid. Yield: 3.46 g (4.57 mmol, 90%). M. p.: 174 °C. ^{1}H NMR (500 MHz, $CDCl_{3,}$): δ = 9.22 (d, 1H, ^4J = 1.7 Hz, 6-Ar-*H*-3), 8.96 (dd, 1H, ^3J = 8.0 Hz, ^4J = 1.7 Hz, 6-Ar-*H*-5), 8.81 (m_c (d), 4H, ^3J = 8.6 Hz, 2,4-Ar-*H*-2,6), 8.15 (m_c (d), 4H, ^3J = 8.6 Hz, 2,4-Ar'-*H*-3,5), 8.13 (m_c (d), 2H, ^3J = 8.6 Hz, 6-Ar'-*H*-3,5), 7.81 (m_c (d), 4H, ^3J = 8.6 Hz, 2,4-Ar-*H*-3,5), 7.74 (m_c (d), 4H, ^3J = 8.6 Hz, 2,4-Ar'-*H*-2,6), 7.61 (d, 1H, ^3J = 8.0 Hz, 6-

Ar-*H*-6), 7.44 (m_c (d), 2H, ^3J = 8.6 Hz, 6-Ar'-*H*-2,6), 3.97 (s, 9H, OCH_3) ppm. ^{13}C NMR (125 MHz, $CDCl_3$): δ = 171.6 (s, triazine-*C*-2,4), 169.3 (s, triazine-*C*-6), 166.8 (s, 2,4-Ar'-CO_2Me), 166.5 (s, 6-Ar'-CO_2Me), 149.6 (s, 6-Ar-*C*-2), 144.4 (s, 2,4-Ar'-*C*-1), 144.3 (s, 2,4-Ar-*C*-4), 141.4 (s, 6-Ar'-*C*-1), 138.8 (s, 6-Ar-*C*-1), 137.3 (s, 6-Ar-*C*-4), 135.2 (s, 2,4-Ar-*C*-1), 132.2 (d, 6-Ar-*C*-5), 132.1 (d, 6-Ar-*C*-6), 130.4 (s, 6-Ar'-*C*-4), 130.3 (d, 2,4-Ar'-*C*-3,5), 130.1 (d, 2,4-Ar-*C*-3,5), 129.7 (s, 2,4-Ar'-*C*-4), 129.7 (d, 6-Ar'-*C*-2,6), 128.0 (d, 2,4-Ar'-*C*-2,6), 127.6 (6-Ar'-*C*-3,5), 127.2 (d, 2,4-Ar-*C*-2,6), 124.6 (d, 6-Ar-*C*-3), 52.3 (q, 6-Ar'-CO_2CH_3), 52.2 (q, 2,4-Ar'-CO_2CH_3) ppm. MS (MALDI, Cl-CCA): *m/z* = 757 $[M + H]^+$, 767 $[M + Na]^+$, 776 $[M + K]^+$. IR (ATR): $\tilde{\nu}$ = 3076, 3006 (aryl-H), 2951, 2895 (CH-val.), 2844 (OCH_3), 1717 (C=O), 1607, 1580, 1563, 1506 (arom. C=C, arom. C=N), 1535, 1314 (NO2), 1434 (CH-Def.), 1357 (C-N-val.), 1257, 1105 (CO_2Me), 813 (1,4-disubst. aryl, 1,2,4-trisubst. aryl) cm^{-1}. MS (EI, 70 eV): *m/z* (%) = 756 (65) $[M]^{+\cdot}$, 726 (100) $[M – CH_3O + H]^{+\cdot}$, 238 (61) $[(M – C_{15}H_{10}N_2O_4)/2]^+$, 206 (70) $[(M – C_{15}H_{10}N_2O_4)/2 – CH_3O]^+$, 178 (63) $[(M – C_{15}H_{10}N_2O_4)/2 – C_2H_3O]^+$. HRMS (EI): *m/z* = calcd. 756.2219; found 756.2191 (Δ 3.9 ppm). Elemental analysis ($C_{45}H_{32}N_4O_8$) (756.76): calcd. C 71.42 H 4.26 N 7.40; found C 71.54 H 4.16 N 7.32.

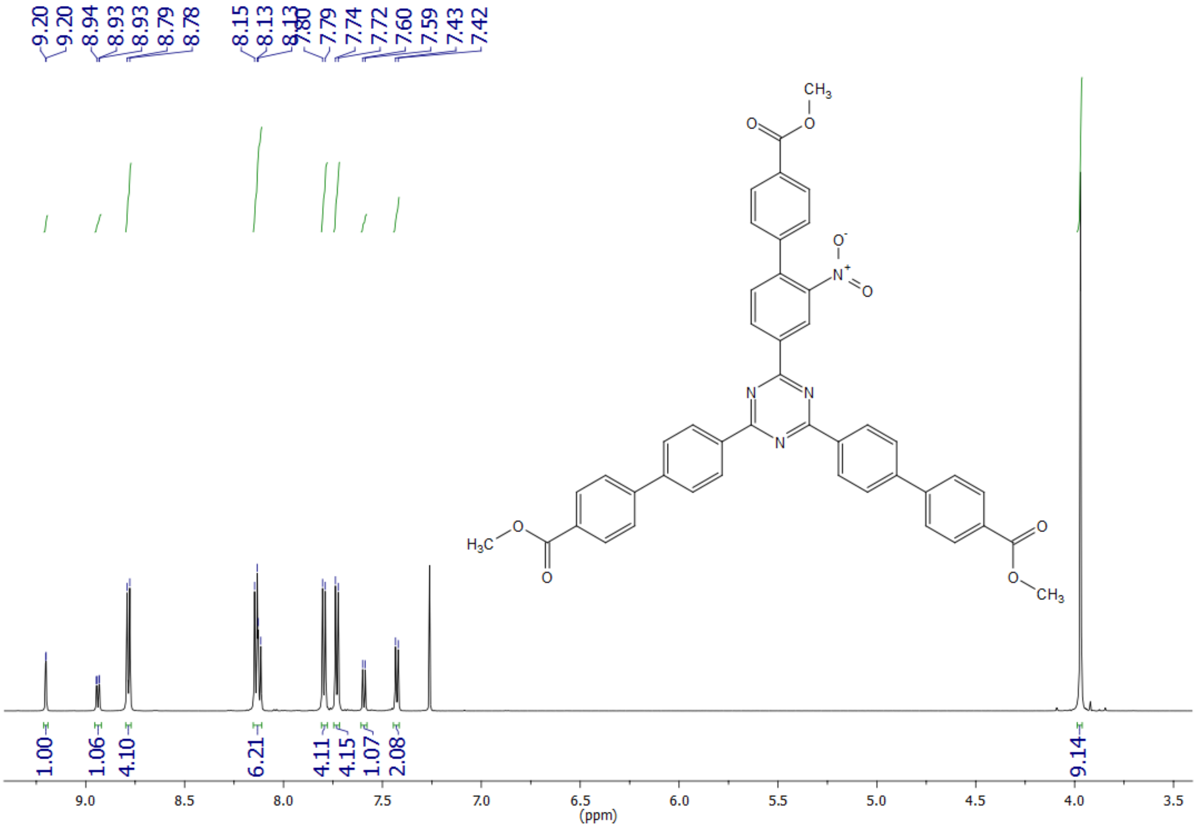
9.20
9.20
8.94
8.93
8.93
8.79
8.78
8.15
8.13
8.13
7.80
7.79
7.74
7.72
7.60
7.59
7.43
7.42
1.00
1.06
4.10
6.21
4.11
4.15
1.07
2.08
9.14
CH3
O
N+
N
H3C
(ppm)

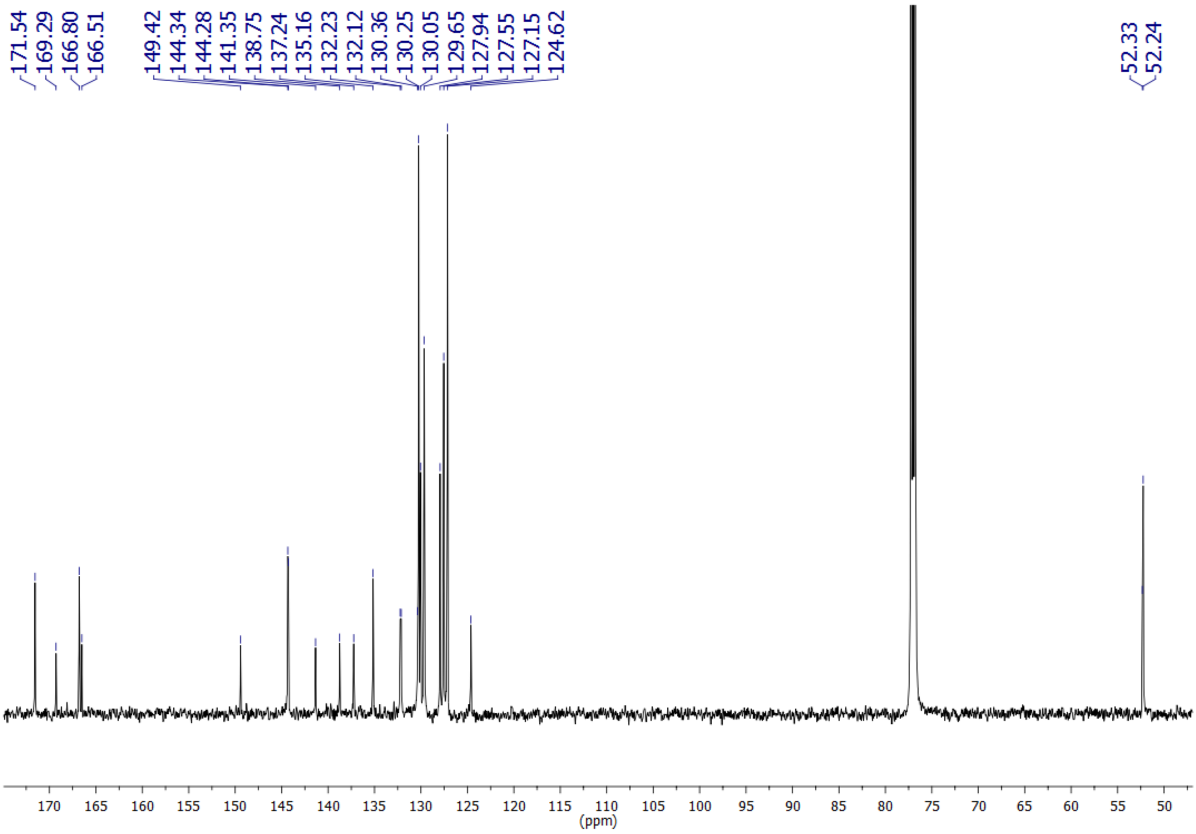
171.54
169.29
166.80
166.51
149.42
144.34
144.28
141.35
138.75
137.24
135.16
132.23
132.12
130.36
130.25
130.05
129.65
127.94
127.55
127.15
124.62
52.33
52.24
(ppm)

2,4-Bis[4’-(methoxycarbonyl)-biphenyl-4-yl]-6-[2-methoxy-4’-(methoxycarbonyl)-biphenyl-4-yl]-1,3,5-triazine (16c)

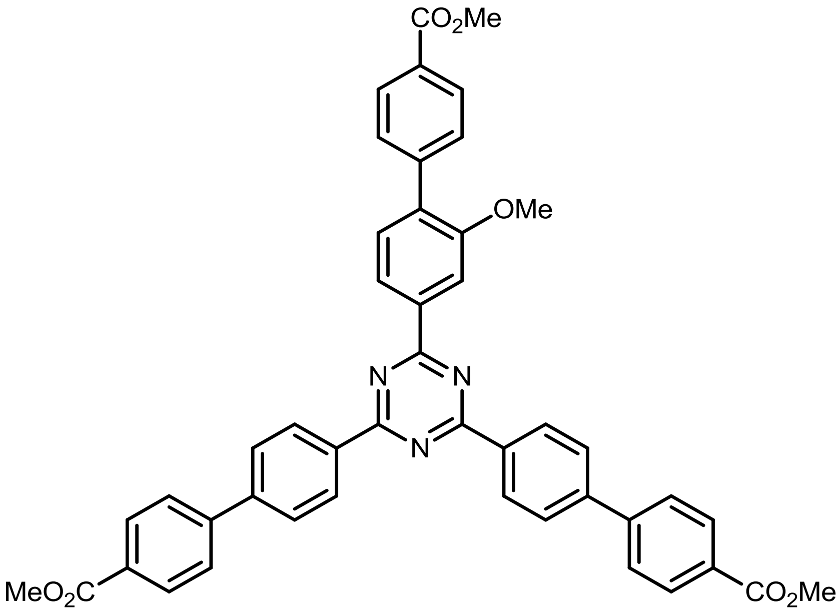

To a mixture of 2-(4-bromo-3-methoxyphenyl)-4,6-bis(4-bromophenyl)-1,3,5-triazine (**3c**, 100 mg, 174 µmol), 4-methoxycarbonyl-phenylboronic acid (**15**, 130 mg, 783 µmol) and potassium phosphate (250 mg, 1.18 mmol) in 1,4-dioxane (10 mL) and deionized water (1 mL) under nitrogen, tetrakis(triphenylphosphine)palladium(0) (19 mg, 16 µmol) was added. The mixture was stirred for 48 h at 100 °C, the solvent was evaporated in vacuo, the residue was dissolved in chloroform (50 ml) and washed with deionized water (3 x 25 mL) and brine (25 mL). The organic layer was dried with magnesium sulfate and heated after activated charcoal had been added. After filtration through celite, the solvent was evaporated in vacuo and the crude product was recrystallized from a boiling mixture of toluene and *n*-heptane. A colourless solid was obtained. Yield: 94 mg (127 µmol, 73%). M. p.: 203 °C. ^{1}H NMR (500 MHz, $CDCl_{3,}$): δ = 8.85 (d, 4H, ^3J = 8.5 Hz, 2,4-Ar-*H*-2,6), 8.47 (dd, 1H, ^3J = 7.9 Hz, ^4J = 1.5 Hz, 6-Ar-*H*-5), 8.40 (d, 1H, ^3J = 1.5 Hz, 6-Ar-*H*-3), 8.16 (d, 4H, ^3J = 8.5 Hz, 2,4-Ar’-*H*-3,5), 8.12 (d, 2H, ^3J = 8.5 Hz, 6-Ar’-*H*-3,5), 7.83 (d, 4H, ^3J = 8.5 Hz, 2,4-Ar-*H*-3,5), 7.76 (d, 4H, ^3J = 8.5 Hz, 2,4-Ar’-*H*-2,6), 7.70 (d, 2H, ^3J = 8.5 Hz, 6-Ar’-*H*-2,6), 7.54 (d, 1H, ^3J = 7.9 Hz, 6-Ar-*H*-6), 4.04 (s, 3H, OCH_3), 3.97 (s, 6H, 2,4-Ar’-CO_2CH_3), 3.96 (s,

3H, 6-Ar'-CO_2CH_3) ppm. ^{13}C NMR (125 MHz, $CDCl_3$): δ = 171.2 (s, triazine-*C*-2,4), 171.2 (s, triazine-*C*-6), 167.0 (s, 2,4-Ar'-*C*O_2Me), 166.9 (s, 6-Ar'-*C*O_2Me), 156.8 (s, 6-Ar-*C*-2), 144.6 (s, 2,4-Ar'-*C*-1), 144.0 (s, 2,4-Ar-*C*-4), 142.6 (s, 6-Ar'-*C*-1), 137.2 (s, 6-Ar-*C*-4), 135.8 (s, 2,4-Ar-*C*-1), 133.8 (s, 6-Ar-*C*-1), 131.0 (s, 6-Ar-*C*-6), 130.2 (d, 2,4-Ar'-*C*-3,5), 129.6 (d, 2,4-Ar-*C*-2,6), 129.6 (d, 6-Ar'-*C*-2,6), 129.5 (s, 2,4-Ar'-*C*-4), 129.4 (d, 6-Ar'-*C*-3,5), 129.0 (s, 6-Ar'-*C*-4), 127.5 (d, 2,4-Ar-*C*-3,5), 127.2 (d, 2,4-Ar'-*C*-2,6), 121.9 (d, 6-Ar-*C*-5), 111.4 (d, 6-Ar-*C*-3), 55.8 (q, OCH_3), 52.2 (q, 2,4-Ar'-CO_2CH_3), 52.1 (q, 6-Ar'-CO_2CH_3) ppm. MS (EI, 70 eV): *m/z* = 741 (100) $[M]^{+\cdot}$, 238 (69) $[M - C_{31}H_{23}N_2O_5]^+$. IR (ATR): $\tilde{\nu}$ = 3005 (aryl-H), 2954, 2878, 2840 (CH-val.), 1715 (C=O), 1607, 1582, 1508, 1419 (arom. C=C, arom. C=N), 1434 (CH-Def.), 1360 (C-N-val.), 1273 (aryl-OCH_3), 812 (1,4-disubst. aryl, 1,3,4-trisubst. aryl) cm^{-1}. HRMS (EI): *m/z* = calcd. 741.2475; found 741.2465 (Δ 1.42 ppm). Elemental analysis ($C_{46}H_{35}N_3O_7$) (741.79): calcd. C 74.48 H 4.76 N 5.66; found C 74.59 H 4.93 N 5.69.

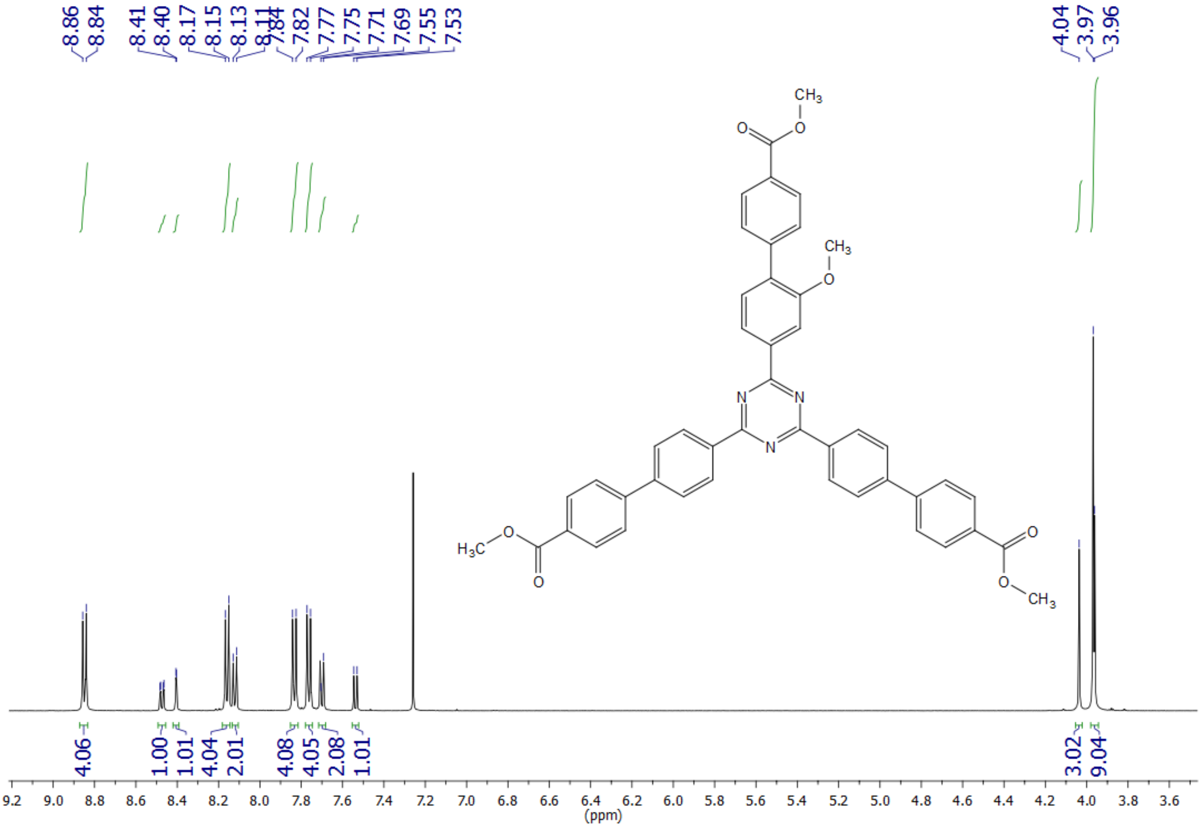
8.86
8.84
8.41
8.40
8.17
8.15
8.13
8.11
7.84
7.82
7.77
7.75
7.71
7.69
7.55
7.53
4.04
3.97
3.96
CH3
O
CH3
N
N
N
H3C
CH3
4.06
1.00
1.01
4.04
2.01
4.08
4.05
2.08
1.01
3.02
9.04
(ppm)

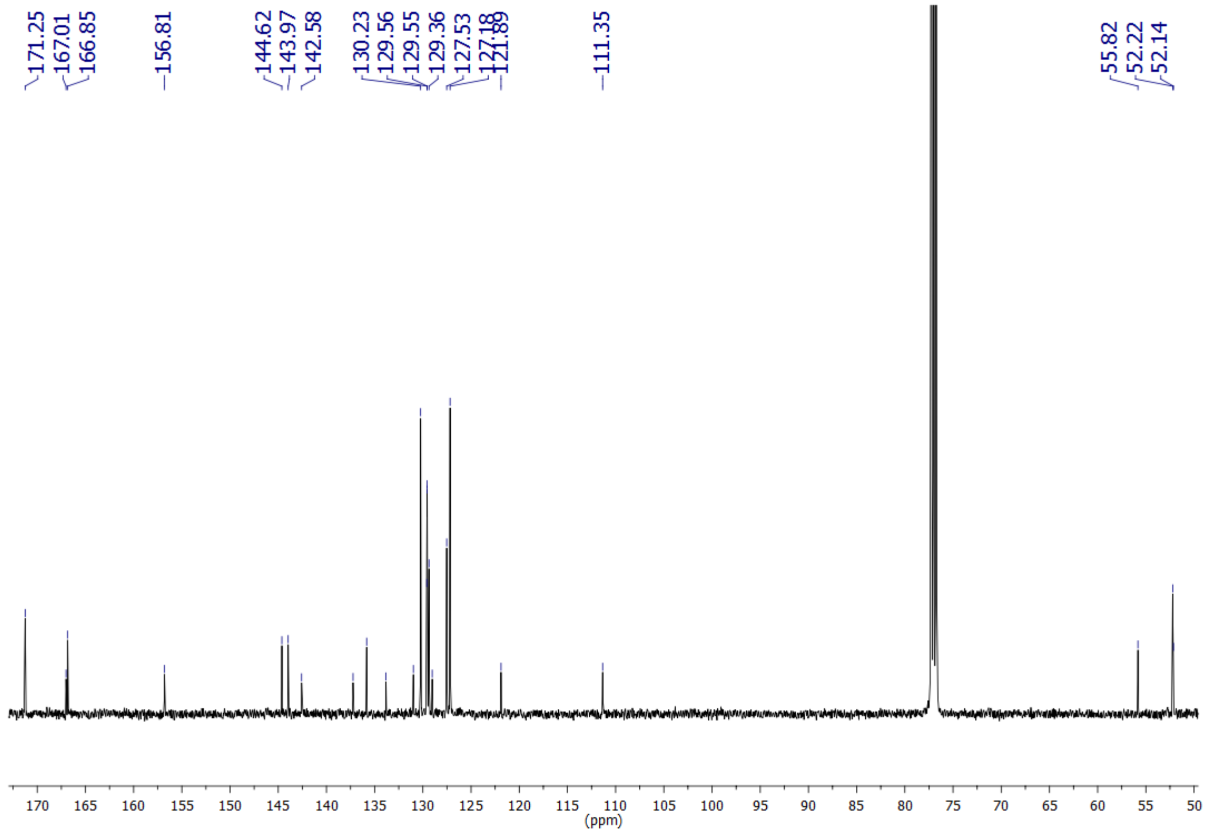
171.25
167.01
166.85
156.81
144.62
143.97
142.58
130.23
129.56
129.55
129.36
127.53
127.18
121.89
111.35
55.82
52.22
52.14
(ppm)

2-[2-Amino-4'-(methoxycarbonyl)-biphenyl-4-yl]-4,6-bis[4'-(methoxycarbonyl)-biphenyl-4-yl]-1,3,5-triazine (16d)

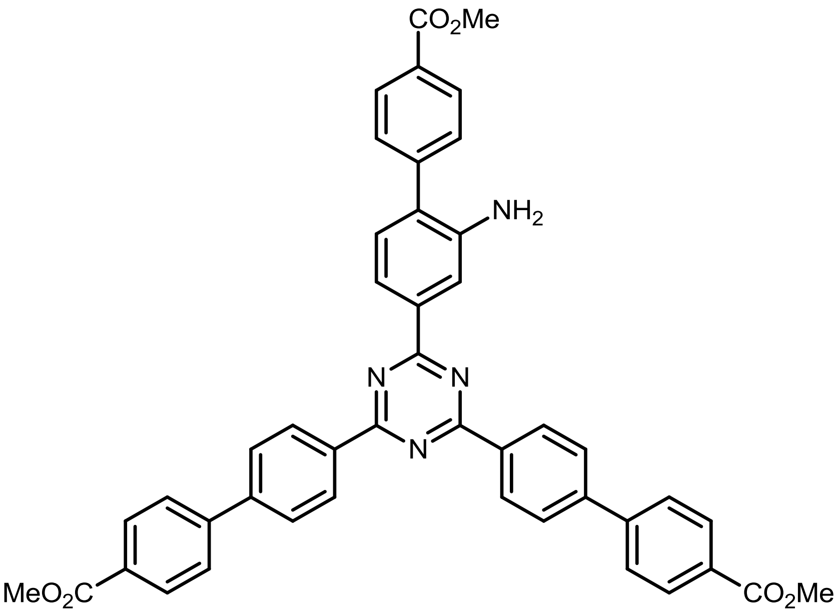

An autoclave was filled with 2-[4'-(methoxycarbonyl)-2-nitro-biphenyl-4-yl]-4,6-bis[4'-(methoxycarbonyl)-biphenyl-4-yl]-1,3,5-triazine (**16b**, 100 mg, 132 µmol) and palladium on charcoal (45 mg, 10%) in dichloromethane (5 mL). The apparatus was evacuated five times and refilled with hydrogen (5 bar). After stirring under a 5 bar hydrogen atmosphere for 5 d at room temp., the mixture was filtered through celite and the solvent was evaporated in vacuo. The crude product was recrystallized from a boiling mixture of toluene and *n*-heptane yielding a yellow solid. Yield: 77 mg (0.11 mmol, 77%). M. p.: 225 °C. ^{1}H NMR (600 MHz, $CDCl_3$): δ = 8.85 (d, 4H, ^3J = 8.3 Hz, 4,6-Ar-*H*-3,5), 8.25 (dd, 1H, ^3J = 8.0 Hz, ^{4}J = 1.3 Hz, 2-Ar-*H*-5), 8.20 (br. s, 1H, 2-Ar-*H*-3), 8.17 (d, 2H, ^3J = 8.2 Hz, 2-Ar'-*H*-3,5), 8.16 (d, 4H, ^3J = 8.4 Hz, 4,6-Ar'-*H*-3,5), 7.83 (d, 4H, ^3J = 8.4 Hz, 4,6-Ar'-*H*-2,6), 7.70 (d, 4H, ^3J = 8.3 Hz, 4,6-Ar-*H*-2,6), 7.64 (d, 2H, ^3J = 8.2 Hz, 2-Ar-*H*-2,6), 7.34 (d, 1H, ^3J = 8.0 Hz, 2-Ar-*H*-6), 3.97 (s, 3H, 2-Ar'-CO_2CH_3), 3.96 (s, 6H, 4,6-Ar'-CO_2CH_3) ppm. ^{13}C NMR (150 MHz, $CDCl_3$): δ = 171.4 (s, triazine-*C*-2), 171.1 (s, triazine-*C*-4,6), 166.9 (s, 4,6-Ar'-CO_2Me), 166.8 (s, 2-Ar'-CO_2Me), 144.6 (s, 2-Ar-*C*-2), 143.8 (s, 2-Ar'-*C*-1), 143.7 (s, 4,6-Ar-*C*-1), 143.7 (s, 4,6-Ar'-*C*-1), 136.8 (s, 2-Ar-*C*-4), 135.9 (s, 4,6-Ar-*C*-4),

130.9 (d, 2-Ar-*C*-6), 130.7 (d, 4,6-Ar'-*C*-3,5), 130.5 (s, 4,6-Ar'-*C*-4), 130.2 (d, 2-Ar'-*C*-3,5), 130.0 (s, 2-Ar-*C*-1), 129.5 (d, 4,6-Ar-*C*-3,5), 129.3 (s, 2-Ar-*C*-4), 128.9 (d, 2-Ar'-*C*-2,6), 127.4 (d, 4,6-Ar-*C*-2,6), 127.2 (d, 4,6-Ar'-*C*-2,6), 119.6 (d, 2-Ar-*C*-5), 116.1 (d, 2-Ar-*C*-3), 52.2 (q, 2-Ar'-$CO_2\mathit{C}H_3$), 52.2 (q, 4,6-Ar'-$CO_2\mathit{C}H_3$) ppm. MS (EI, 70 eV): *m/z* = 726 (100) $[M]^{+\cdot}$. IR (ATR): $\tilde{\nu}$ = 3350 (NH_2), 3001 (aryl-H), 2954, 2846 (CH–val.), 1715 (C=O), 1607, 1582, 1507, 1419 (arom. C=C, arom. C=N), 1434 (CH-Def.) 1360 (C-N-val.), 812 (1,4-disubst. aryl, 1,3,4-trisubst. aryl) cm^{-1}. HRMS (EI): *m/z* = calcd. 726.2478; found 726.2465 (Δ 1.78 ppm). Elemental analysis ($C_{45}H_{34}N_4O_6$) (726.77): calcd. C 74.73 H 7.71 N 4.72; ($C_{45}H_{34}N_4O_6\cdot1.3H_2O\cdot0.5C_7H_8$) (796.26): calcd. C 73.16 H 5.14 N 7.04; found C 73.27 H 5.46 N 7.35.

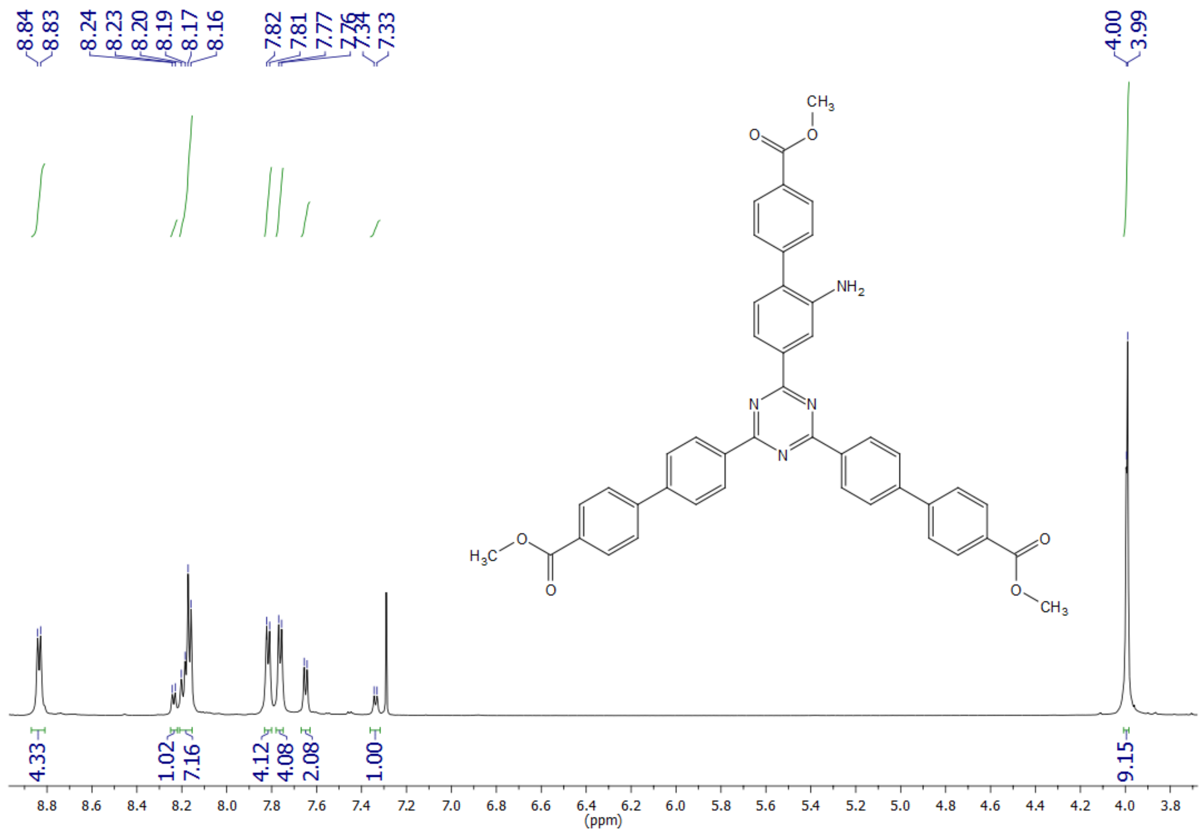

8.84
8.83
8.24
8.23
8.20
8.19
8.17
8.16
7.82
7.81
7.77
7.76
7.34
7.33
4.00
3.99
CH3
O
O
NH2
N
N
N
H3C
O
O
O
O
CH3
4.33
1.02
7.16
4.12
4.08
2.08
1.00
9.15
(ppm)

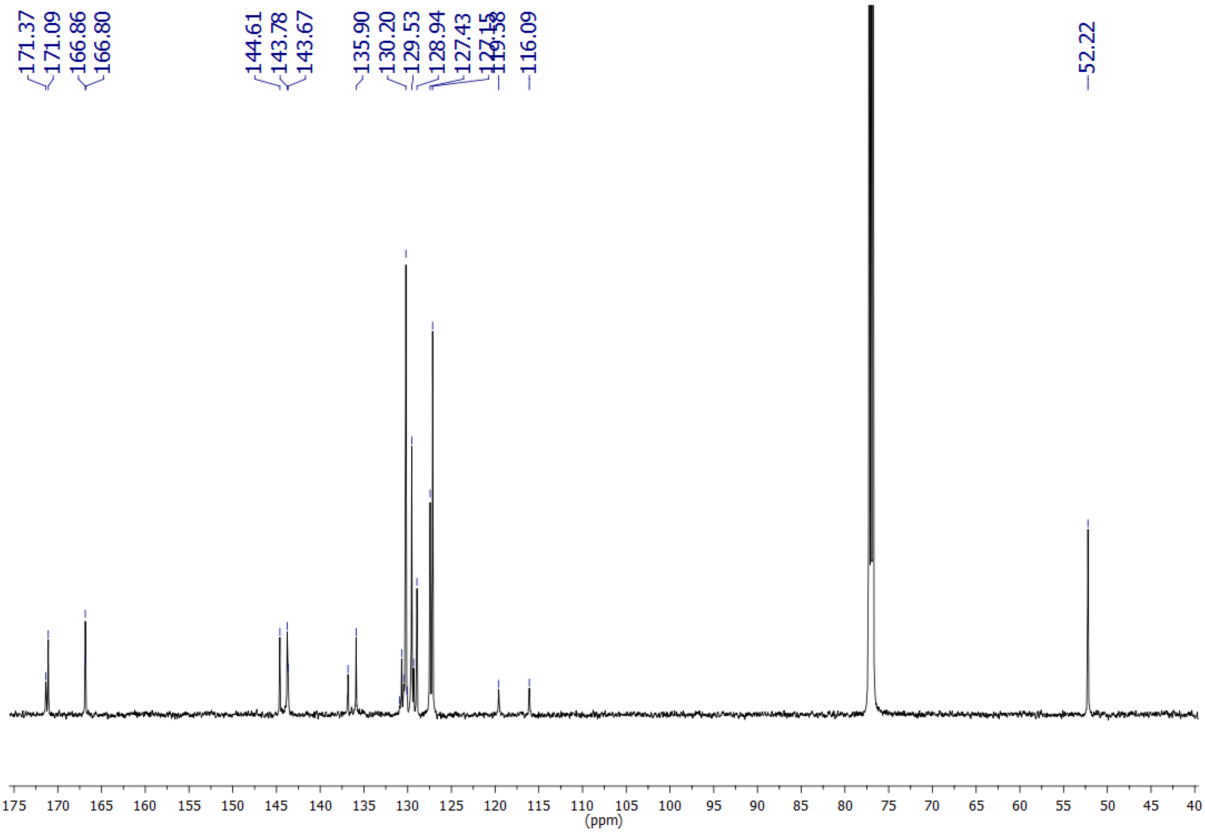

171.37
171.09
166.86
166.80
144.61
143.78
143.67
135.90
130.20
129.53
128.94
127.43
127.15
119.58
116.09
52.22
(ppm)

2,4-Bis(4’-carboxy-biphenyl-4-yl)-6-(4’-carboxy-2-nitro-biphenyl-4-yl)-1,3,5-triazine (17b)

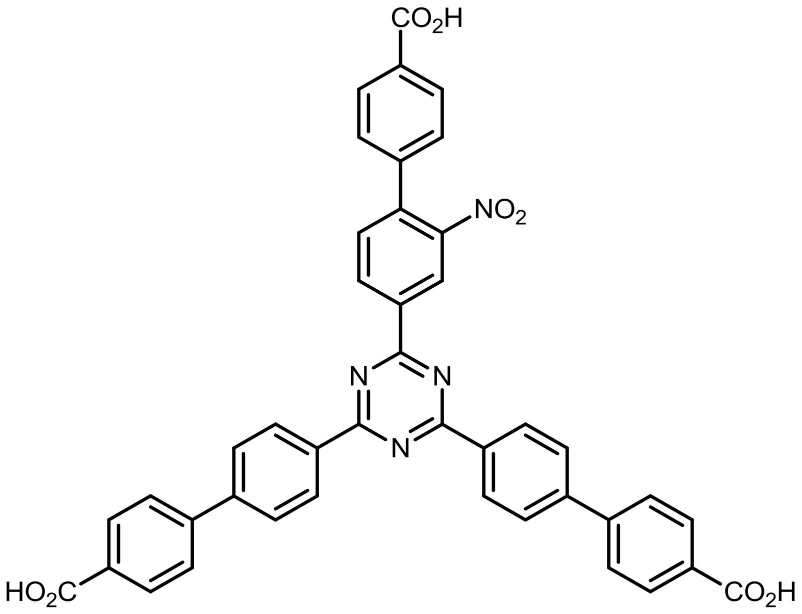

Lithium hydroxide monohydrate (5.12 g, 122 mol) was added to 2-[4’-(methoxycarbonyl)-2-nitro-biphenyl-4-yl]-4,6-bis[4’-(methoxycarbonyl)-biphenyl-4-yl]-1,3,5-triazine (**16b**, 3.30 g, 4.36 mmol) in a mixture of tetrahydrofuran (430 mL) and deionized water (45 mL). After stirring for 48 h at room temp., the solvent was evaporated in vacuo, the yellow residue was dissolved in water and acidified with hydrochloric acid (3 M). The yellow precipitate was filtered off and washed with deionized water and chloroform. Yield: 2.99 g (4.18 mmol, 96%). M. p.: >300 °C. ^{1}H NMR (500 MHz, DMSO-d_6): δ = 8.99 (d, 1H, ^{4}J = 1.4 Hz, 6-Ar-*H*-2), 8.77 (dd, 1H, ^{3}J = 7.9 Hz, ^{4}J = 1.4 Hz, 6-Ar-*H*-6), 8.58 (d, 4H, ^{3}J = 8.3 Hz, 2,4-Ar-*H*-2,6), 8.00 (d, 4H, ^{3}J = 8.3 Hz, 2,4-Ar’-*H*-3,5), 7.98 (d, 2H, ^{3}J = 8.3 Hz, 6-Ar’-*H*-3,5), 7.82 (d, 4H, ^{3}J = 8.3 Hz, 2,4-Ar-*H*-3,5), 7.77 (d, 4H, ^{3}J = 8.3 Hz, 2,4-Ar’-*H*-2,6), 7.64 (d, 1H, ^{3}J = 7.9 Hz, 6-Ar-*H*-5), 7.38 (d, 2H, ^{3}J = 8.3 Hz, 6-Ar’-*H*-2,6) ppm. ^{13}C NMR (125 MHz, DMSO-d_6): δ = 171.1 (s, triazine-*C*-2,4), 169.1 (s, triazine-*C*-6), 167.4 (s, 2,4-Ar-*C*O$_2$H), 167.2 (s, 6-Ar-*C*O$_2$H), 149.2 (d, 6-Ar-*C*-3), 143.7 (s, 2,4-Ar’-*C*-1), 143.4 (s, 2,4-Ar-*C*-4), 141.1 (s, 6-Ar’-*C*-1), 138.4 (s, 6-Ar-*C*-4), 136.7 (s, 6-Ar-*C*-1), 134.9 (s, 2,4-Ar-*C*-1), 132.9 (s, 6-Ar’-*C*-4), 132.7 (d, 6-Ar-*C*-6), 131.3 (s, 2,4-Ar’-*C*-4), 130.8 (d, 6-Ar-*C*-5), 130.4 (d, 2,4-Ar’-*C*-3,5), 130.0 (d, 2,4-Ar-*C*-3,5), 129.7 (d, 6-Ar’-

C-2,6), 129.0 (d, 2,4-Ar-*C*-2,6), 127.7 (6-Ar'-*C*-3,5), 127.4 (d, 2,4-Ar'-*C*-2,6), 124.3 (s, 6-Ar-*C*-2) ppm. MS (MALDI, Cl-CCA): *m/z* = 715 $[M + H]^+$. IR (ATR): $\tilde{\nu}$ = 3400 (br., OH), 3005 (aryl-H), 1714 (C=O), 1607, 1582, 1506, 1419 (arom. C=C, arom. C=N), 1510, 1316 (NO_2), 1359 (C-N–val.), 813 (1,4-disubst. aryl, 1,3,4-trisubst. aryl) cm^{-1}. Elemental analysis ($C_{42}H_{26}N_4O_8$) (714.69): calcd. C 70.58 H 3.67 N 7.84; ($C_{42}H_{26}N_4O_8 \cdot 1.35H_2O$) (738.99): calcd. C 68.69 H 3.91 N 7.58; found C 68.69 H 4.35 N 7.36.

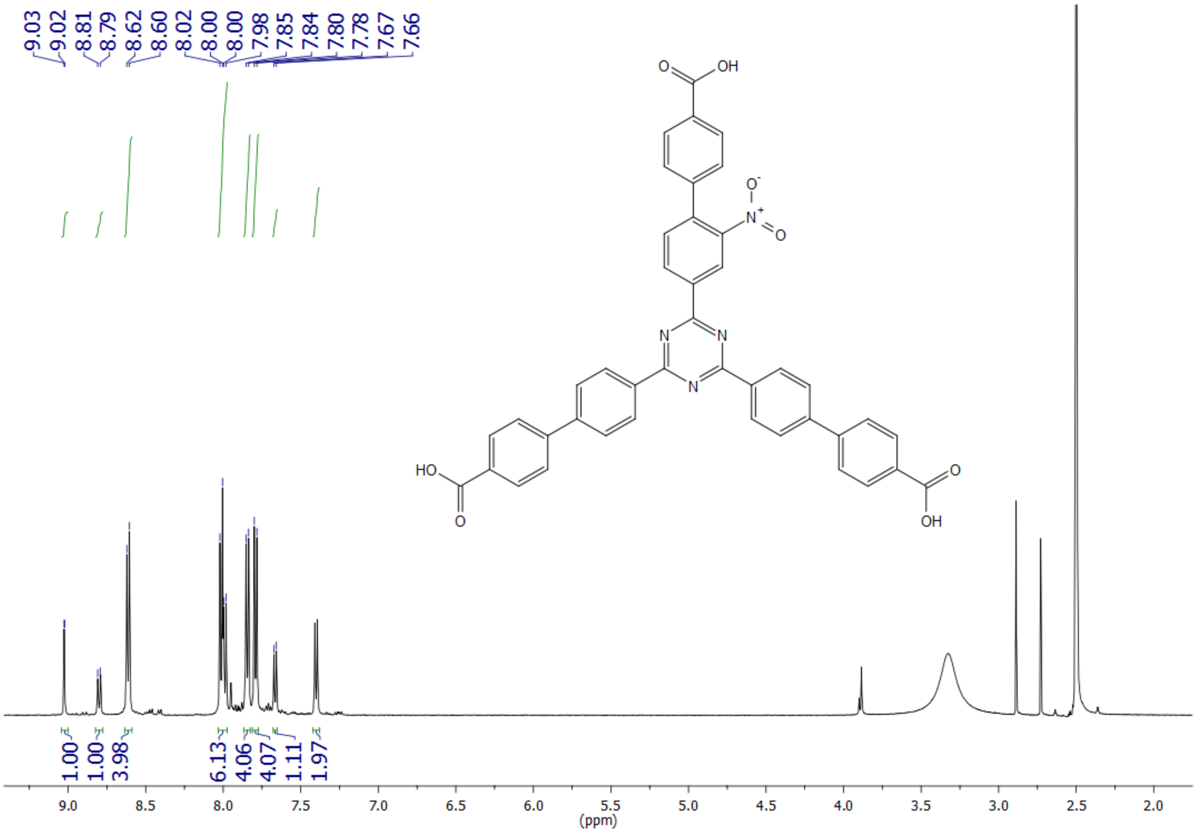
9.03
9.02
8.81
8.79
8.62
8.60
8.02
8.00
8.00
7.98
7.85
7.84
7.80
7.78
7.67
7.66
O
OH
O-
N+
O
N
N
N
HO
O
O
OH
1.00
1.00
3.98
6.13
4.06
4.07
1.11
1.97
9.0
8.5
8.0
7.5
7.0
6.5
6.0
5.5
5.0
4.5
4.0
3.5
3.0
2.5
2.0
(ppm)

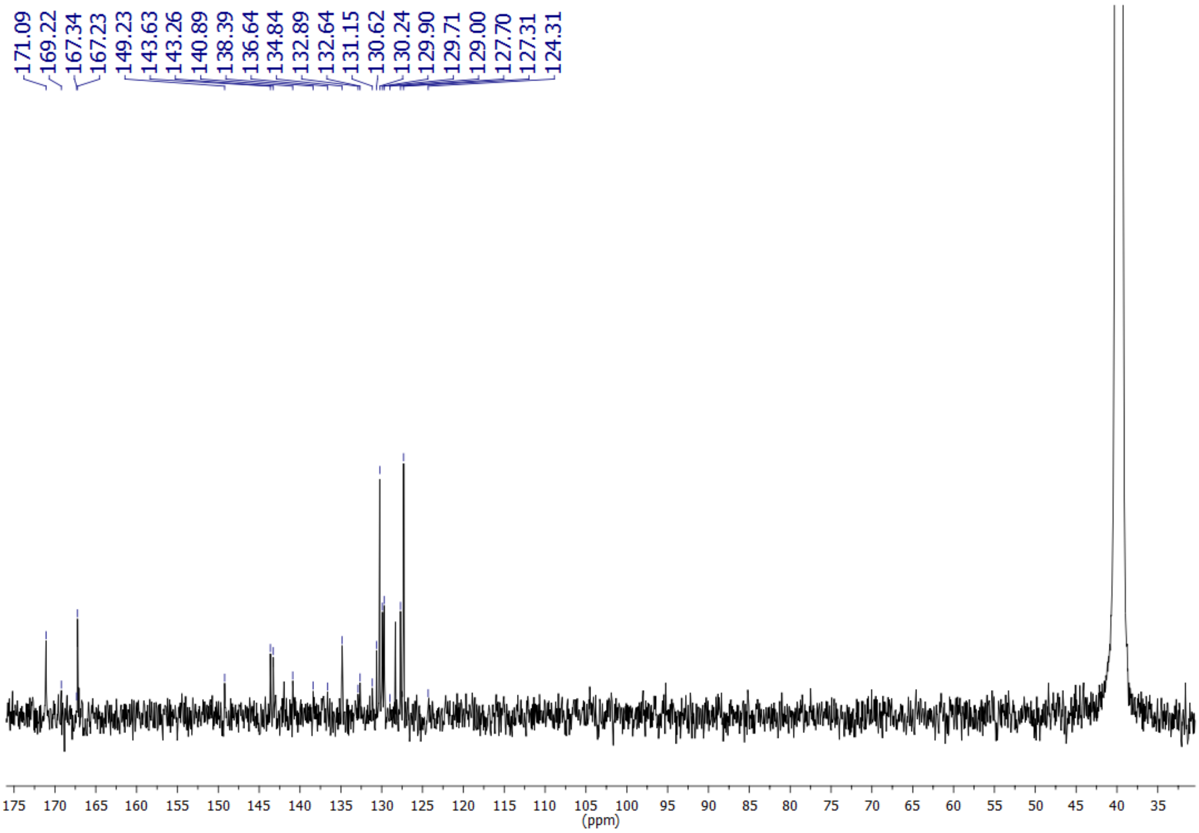
171.09
169.22
167.34
167.23
149.23
143.63
143.26
140.89
138.39
136.64
134.84
132.89
132.64
131.15
130.62
130.24
129.90
129.71
129.00
127.70
127.31
124.31
175
170
165
160
155
150
145
140
135
130
125
120
115
110
105
100
95
90
85
80
75
70
65
60
55
50
45
40
35
(ppm)

2,4-Bis(4'-carboxy-biphenyl-4-yl)-6-(4'-carboxy-2-methoxy-biphenyl-4-yl]-1,3,5-triazine (17c)

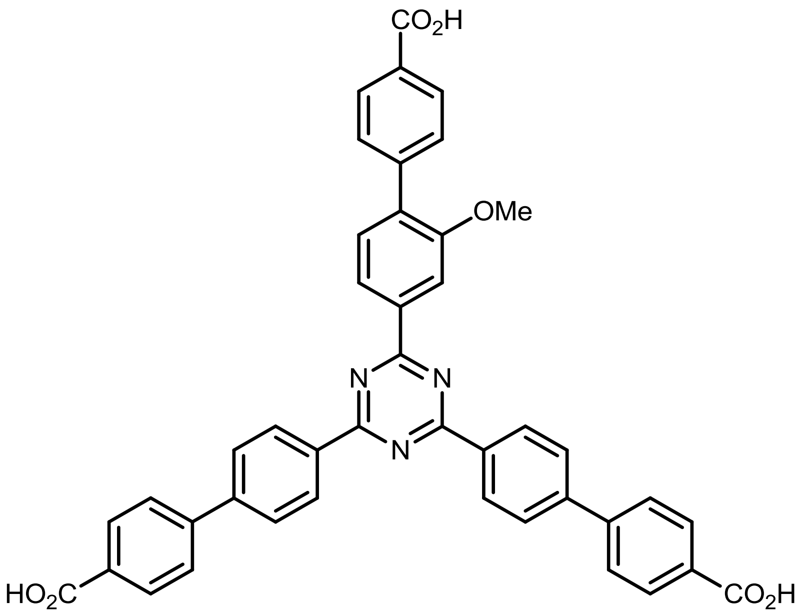

A suspension of 2,4-bis[4'-(methoxy-carbonyl)biphenyl-4-yl]-6-[2-methoxy-4'-(methoxycarbonyl)-biphenyl-4-yl]-1,3,5-triazine (**16c**, 50 mg, 67.5 µmol) and lithium hydroxide monohydrate (101 mg, 2.41 mmol) in a mixture of tetrahydrofuran (10 mL) and deionized water (1.5 mL) was stirred at 60 °C for 24 h. After removal of the tetrahydrofuran in vacuo, the aq. layer was diluted slightly and acidified with hydrochloric acid (3 M). The precipitate was filtered off and washed thoroughly with deionized water and chloroform. Yield: 47 mg (67 µmol, >99%). M. p.: >300 mg. ^{1}H NMR (500 MHz, DMSO-d_6): δ = 13.01 (br. s, 3H, CO_2H), 8.74 (d, 4H, ^3J = 8.4 Hz, 2,4-Ar-*H*-3,5), 8.36 (m_c (dd), 1H, J = 7.9 Hz, ^4J = 1.2 Hz, 6-Ar-*H*-5), 8.29 (m_c (d), 1H, ^4J = 1.2 Hz, 6-Ar-*H*-3), 8.05 (d, 4H, ^3J = 8.4 Hz, 2,4-Ar'-*H*-3,5), 8.00 (d, 2H, ^3J = 8.4 Hz, 6-Ar'-*H*-3,5), 7.94 (d, 4H, ^3J = 8.4 Hz, 2,4-Ar-*H*-2,6), 7.86 (d, 4H, ^3J = 8.4 Hz, 2,4-Ar'-*H*-2,6), 7.67 (d, 2H, ^3J = 8.4 Hz, 6-Ar'-*H*-2,6), 7.56 (d, 1H, ^3J = 7.9 Hz, 6-Ar-*H*-6), 3.95 (s, 3H, OCH_3) ppm. ^{13}C NMR (125 MHz, DMSO-d_6): δ = 171.0 (s, triazine-*C*-2,4), 170.9 (s, triazine-*C*-6), 167.6 (s, 6-Ar'-CO_2H), 167.5 (s, 2,4-Ar'-CO_2H), 156.9 (s, 6-Ar-*C*-2), 143.6 (br. s, 2,4-Ar-*C*-1, 2,4-Ar'-*C*-1), 142.1 (s, 6-Ar'-*C*-1), 136.9 (s, 6-Ar-*C*-4), 135.4 (s, 2,4-Ar-*C*-4), 133.6 (s, 6-Ar-*C*-1), 131.7 (d, 6-Ar-*C*-6), 130.8 (s, 2,4-Ar'-*C*-4), 130.5 (d, 2,4-Ar'-*C*-

3,5), 130.1 (s, 6-Ar'-*C*-4), 129.9 (d, 6-Ar'-*C*-2,6), 129.8 (d, 2,4-Ar-*C*-3,5), 129.5 (d, 6-Ar'-*C*-3,5), 127.9 (d, 2,4-Ar-*C*-2,6), 127.5 (d, 2,4-Ar'-*C*-2,6), 121.9 (d, 6-Ar-*C*-5), 111.5 (d, 6-Ar-*C*-3), 56.1 (q, OCH_3) ppm. MS (MALDI, Cl-CCA): *m/z* = 700 $[M + H]^+$. IR (ATR): $\tilde{\nu}$ = 3100 (br., OH), 1684 (C=O), 1605, 1581, 1505, 1417 (arom. C=C, arom. C=N), 1357 (C-N-val.), 1231 (aryl-OCH_3), 813 (1,4-disubst. aryl, 1,3,4-trisubst. aryl) cm^{-1}. Elemental analysis ($C_{43}H_{29}N_3O_7$) (699.70): calcd. C 73.81 H 4.18 N 6.01; ($C_{43}H_{29}N_3O_7 \cdot 0.4H_2O \cdot 0.25CHCl_3$) (738.99): calcd. C 70.51 H 4.11 N 5.70; found C 70.47 H 4.11 N 5.73.

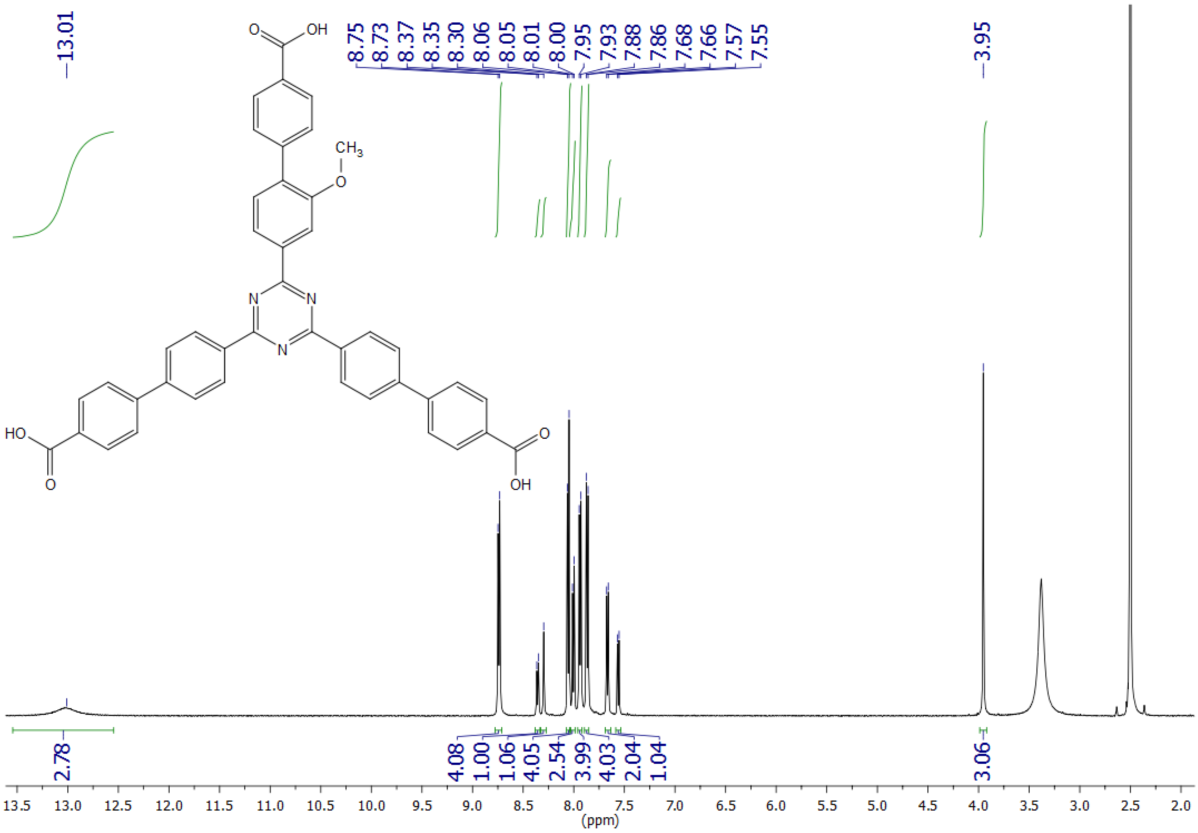
13.01
8.75
8.73
8.37
8.35
8.30
8.06
8.05
8.01
8.00
7.95
7.93
7.88
7.86
7.68
7.66
7.57
7.55
3.95
OH
O
CH3
O
N
N
N
HO
O
O
OH
2.78
4.08
1.00
1.06
4.05
2.54
3.99
4.03
2.04
1.04
3.06
13.5 13.0 12.5 12.0 11.5 11.0 10.5 10.0 9.5 9.0 8.5 8.0 7.5 7.0 6.5 6.0 5.5 5.0 4.5 4.0 3.5 3.0 2.5 2.0
(ppm)

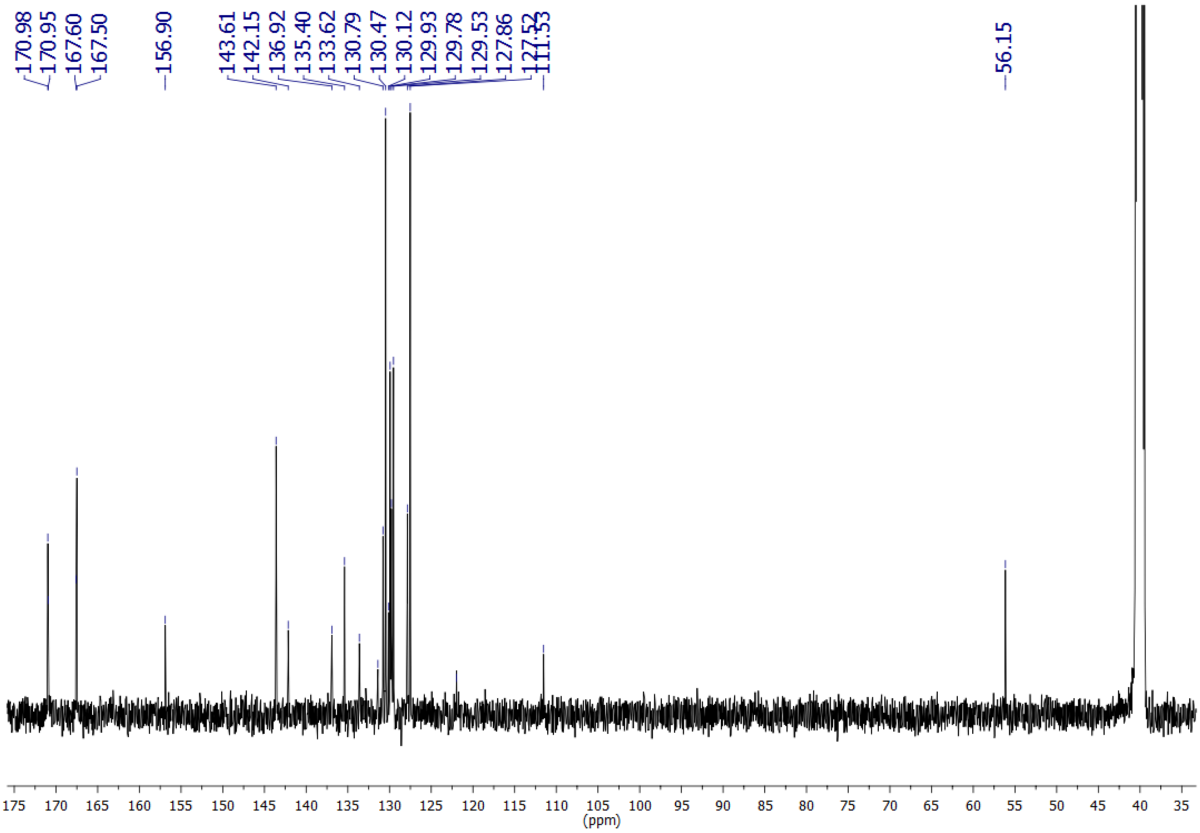
170.98
170.95
167.60
167.50
156.90
143.61
142.15
136.92
135.40
133.62
130.79
130.47
130.12
129.93
129.78
129.53
127.86
127.52
111.53
56.15
175 170 165 160 155 150 145 140 135 130 125 120 115 110 105 100 95 90 85 80 75 70 65 60 55 50 45 40 35
(ppm)

2-(2-Amino-4'-carboxy-biphenyl-4-yl)-4,6-bis(4'-carboxy-biphenyl-4-yl)-1,3,5-triazine (17d)

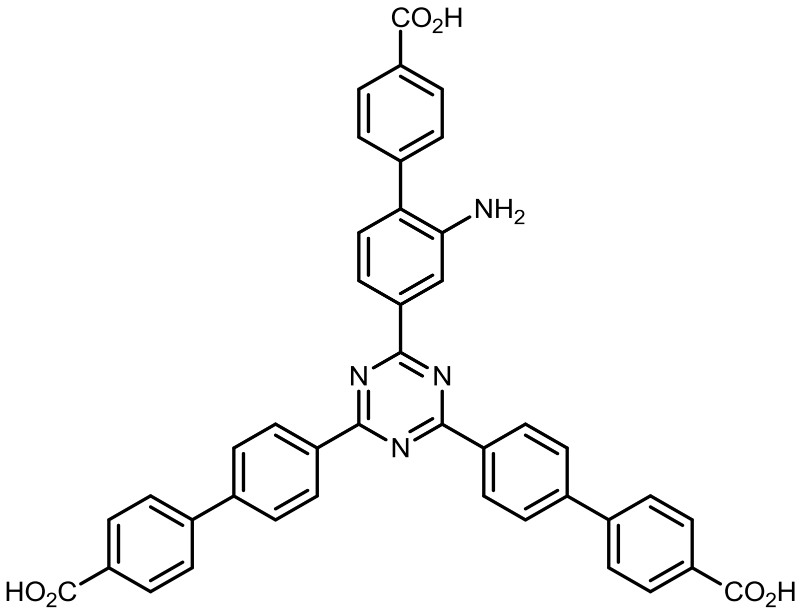

Lithium hydroxide monohydrate (101 mg, 2.41 mmol) and 2-[2-amino-4'-carboxy-biphenyl-4-yl]-4,6-bis[4'-carboxy-biphenyl-4-yl]-1,3,5-triazine (**16d**, 50 mg, 69 µmol) in a mixture of tetrahydrofuran (430 mL) and deionized water (45 mL) were stirred at 60 °C for 48 h. After evaporation of the solvent in vacuo, the yellow residue was dissolved in deionized water and acidified with hydrochloric acid (3 M). The yellow precipitate was filtered off and washed with deionized water and chloroform. Yield: 47 mg (68 µmol, >99%). M. p.: >300 °C. ^{1}H NMR (500 MHz, DMSO-d_6): δ = 13.02 (br. s, 3H, CO_2*H*), 8.81 (d, 4H, ^3J = 8.5 Hz, 4,6-Ar-*H*-3,5), 8.25 (d, 1H, ^4J = 1.6 Hz, 2-Ar-*H*-3), 8.08 (d, 4H, ^3J = 8.5 Hz, 4,6-Ar'-*H*-3,5), 8.05 (d, 2H, ^3J = 8.4 Hz, 2-Ar'-*H*-3,5), 8.03* (dd, 1H, ^4J = 1.6 Hz, 2-Ar-*H*-5), 8.01 (d, 4H, ^3J = 8.5 Hz, 4,6-Ar'-*H*-2,6), 7.93 (d, 4H, ^3J = 8.5 Hz, 4,6-Ar-*H*-2,6), 7.66 (d, 2H, ^3J = 8.4 Hz, 2-Ar'-*H*-2,6), 7.29 (d, 1H, ^3J = 8.0 Hz, 2-Ar-*H*-6) ppm. *signal overlaps with adjacent one unabling the determination of all coupling constants. ^{13}C NMR (125 MHz, DMSO-d_6): δ = 171.1 (s, triazine-*C*-2), 171.0 (s, triazine-*C*-4,6), 167.6 (s, 2-Ar'-CO_2*H*), 167.5 (s, 4,6-Ar'-CO_2*H*), 146.2 (s, 2-Ar-*C*-2), 144.0 (s, 2-Ar'-*C*-1), 143.7 (s, 4,6-Ar-*C*-1), 143.7 (s, 4,6-Ar'-*C*-1), 136.1 (s, 2-Ar-*C*-4), 135.7 (s, 4,6-Ar-*C*-4), 131.2 (d, 2-Ar-*C*-6), 130.8 (s, 4,6-Ar'-*C*-4), 130.5 (d, 4,6-Ar'-*C*-3,5), 130.3 (d, 2-

Ar'-*C*-3,5), 129.9 (s, 2-Ar'-*C*-4), 129.8 (d, 4,6-Ar-*C*-3,5), 129.4 (s, 2-Ar-*C*-1), 129.2 (d, 2-Ar'-*C*-2,6), 127.9 (d, 4,6-Ar-*C*-2,6), 127.6 (d, 4,6-Ar'-*C*-2,6), 117.7 (d, 2-Ar-*C*-5), 116.2 (d, 2-Ar-*C*-3) ppm. MS (MALDI, Cl-CCA): *m/z* = 686 $[M + H]^+$. IR (ATR): $\tilde{\nu}$ = 3100 (br., OH, NH_2), 1684 (C=O), 1606, 1581, 1507, 1419 (arom. C=C, arom. C=N), 1561 (NH_2), 1434 (CH-Def.) 1360 (C-N-val.), 810 (1,4-disubst. aryl, 1,3,4-trisubst. aryl) cm^{-1}. Elemental analysis ($C_{42}H_{18}N_4O_6$) (684.69): calcd. C 73.68 H 4.12 N 8.18; ($C_{42}H_{18}N_4O_6 \cdot 0.4CHCl_3 \cdot 0.2H_2O$) (753.89): calcd. C 69.19 H 3.94 N 7.61; found C 69.41 H 3.91 N 7.31.

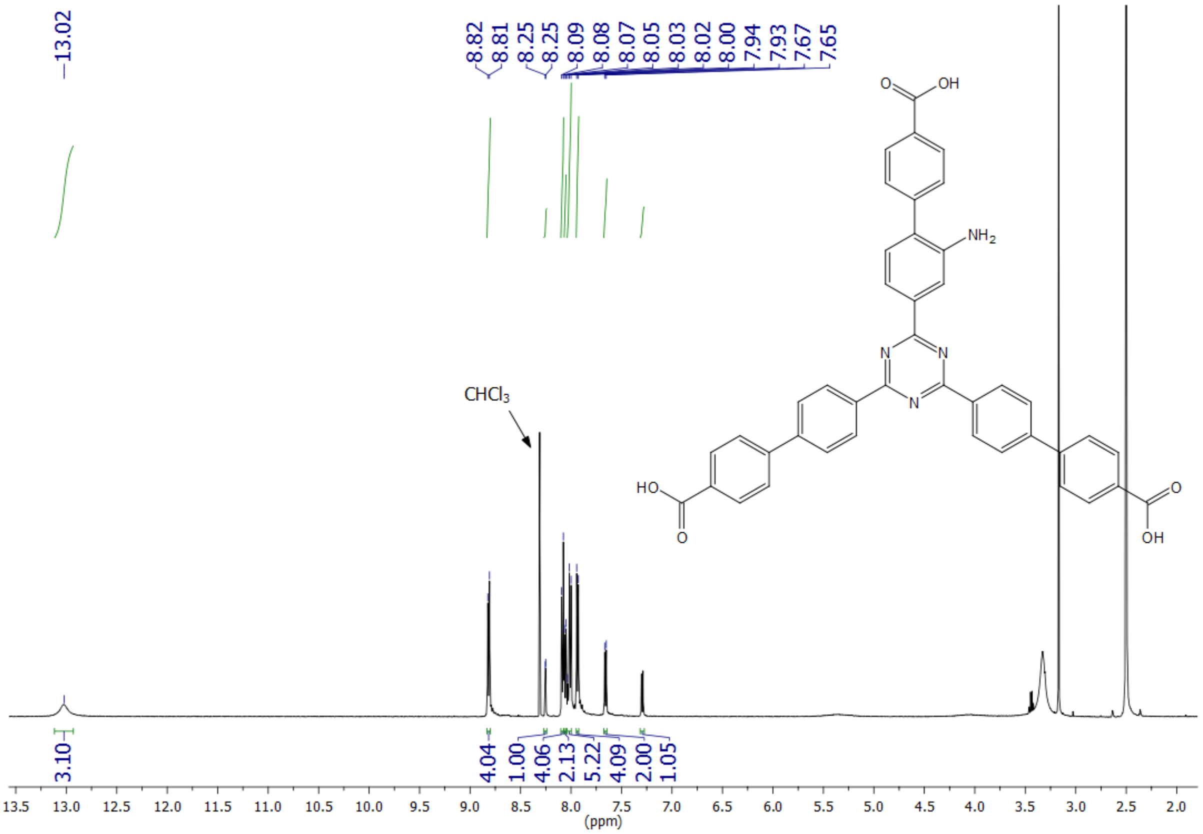

13.02
8.82
8.81
8.25
8.25
8.09
8.08
8.07
8.05
8.03
8.02
8.00
7.94
7.93
7.67
7.65
CHCl3
NH2
N
OH
HO
O
3.10
4.04
1.00
4.06
2.13
5.22
4.09
2.00
1.05
(ppm)

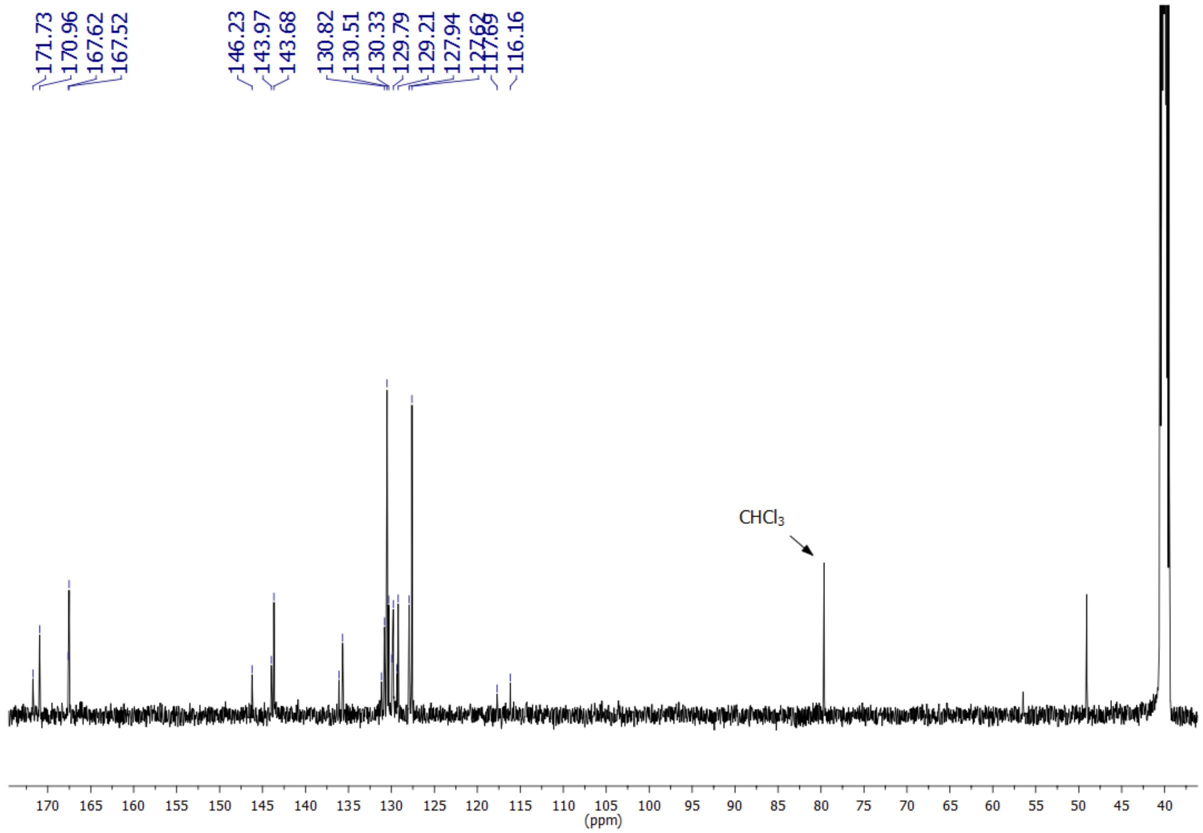

171.73
170.96
167.62
167.52
146.23
143.97
143.68
130.82
130.51
130.33
129.79
129.21
127.94
127.62
116.16
CHCl3
(ppm)

2,4-Bis(4'-carboxy-biphenyl-4-yl)-6-(4'-carboxy-2-hydroxy-biphenyl-4-yl)-1,3,5-triazine (17e)

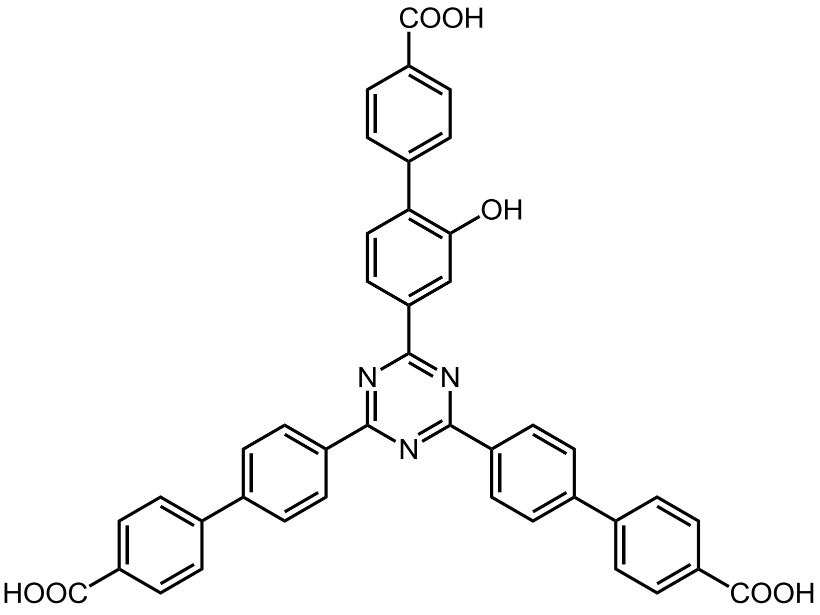

2,4-Bis[4'-(methoxy-carbonyl)-biphenyl-4-yl]-6-[2-methoxy-4'-(methoxycarbonyl)-biphenyl-4-yl]-1,3,5-triazine (**16c**, 300 mg, 400 µmol) and pyridinium hydrochloride (3.60 g, 31.2 mmol) were mixed and stirred for 12 h at 200 °C. After cooling to room temp., sodium hydroxide (2 M) was added to the solution until pH 14 was reached. After stirring for 12 h at 120 °C, the reaction mixture was acidified with conc. hydrochloric acid and the precipitate was filtered off. Washing with water and drying gave a dark brown solid. Yield: 143 mg (209 µmol, 52%). M. p.: > 300 °C. ^{1}H NMR (500 MHz, DMSO-d$_6$): δ = 12.99 (br. s, 3H, COO*H*), 10.18 (s, 1H, O*H*), 8.70 (d, 4H, ^{3}J = 7.6 Hz, 2,4-Ar-*H*-3,5), 8.33 (s, 1H, 6-Ar-*H*-3), 8.18 (d, ^{3}J = 7.8 Hz, 1H, 6-Ar-*H*-5), 8.05 (d, ^{3}J = 8.0 Hz, 4H, 2,4-Ar'-*H*-3,5), 8.00 (d, ^{3}J = 8.2 Hz, 2H, 6-Ar'-*H*-3,5), 7.92 (d, ^{3}J = 7.6 Hz, 4H, 2,4-Ar-*H*-2,6), 7.86 (d, ^{3}J = 8.0 Hz, 4H, 2,4-Ar'-*H*-2,6), 7.74 (d, ^{3}J = 8.2 Hz, 2H, 6-Ar'-*H*-2,6), 7.50 (d, ^{3}J = 7.8 Hz, 1H, 6-Ar-*H*-6) ppm. ^{13}C NMR (125 MHz, DMSO-d$_6$): δ = 171.1 (s, triazine-*C*-6), 170.9 (s, triazine-*C*-2,4), 167.7 (s, 6-Ar'-*C*OOH), 167.5 (s, 2,4-Ar'-*C*OOH), 155.3 (s, 6-Ar-*C*-2), 143.6 (s, 2,4-Ar-*C*-1, 2,4-Ar'-*C*-1), 142.6 (s, 6-Ar'-*C*-1), 136.6 (s, 6-Ar-*C*-4), 135.5 (s, 2,4-Ar-*C*-4), 131.4 (s, 6-Ar-*C*-1), 131.2 (d, 6-Ar-*C*-6), 130.8 (s, 2,4-Ar'-*C*-4), 130.4 (d, 2,4-Ar'-*C*-3,5), 129.8 (d, 6-Ar'-*C*-2,6), 129.7 (d, 2,4-

Ar-*C*-3,5), 129.7 (s, 6-Ar'-*C*-4), 129.5 (d, 6-Ar'-*C*-3,5), 127.8 (d, 2,4-Ar-*C*-2,6), 127.5 (d, 2,4-Ar'-*C*-2,6), 120.5 (d, 6-Ar-*C*-5), 116.5 (d, 6-Ar-*C*-3) ppm. MS (MALDI, Cl-CCA): *m/z* = 686 $[M + H]^+$. IR (ATR): $\tilde{\nu}$ = 3050 (OH), 1681 (C=O), 1606, 1580, 1562, 1504 (arom. C=C, arom. C=N), 1418 (CH-Def.), 1360 (O-H-Def.), 1238, 1175, 1101 (C-O), 852, 813, 772 (1,4-disubst. aryl, 1,2,4-trisubst. aryl) cm^{-1}. Elemental analysis ($C_{42}H_{27}N_3O_7$) (685.18): calcd. C 73.57 H 3.97 N 6.13; ($C_{42}H_{27}N_3O_7 \cdot H_2O$) (703.20): calcd. C 71.69 H 4.15 N 5.97; found C 71.47 H 3.95 N 5.91.

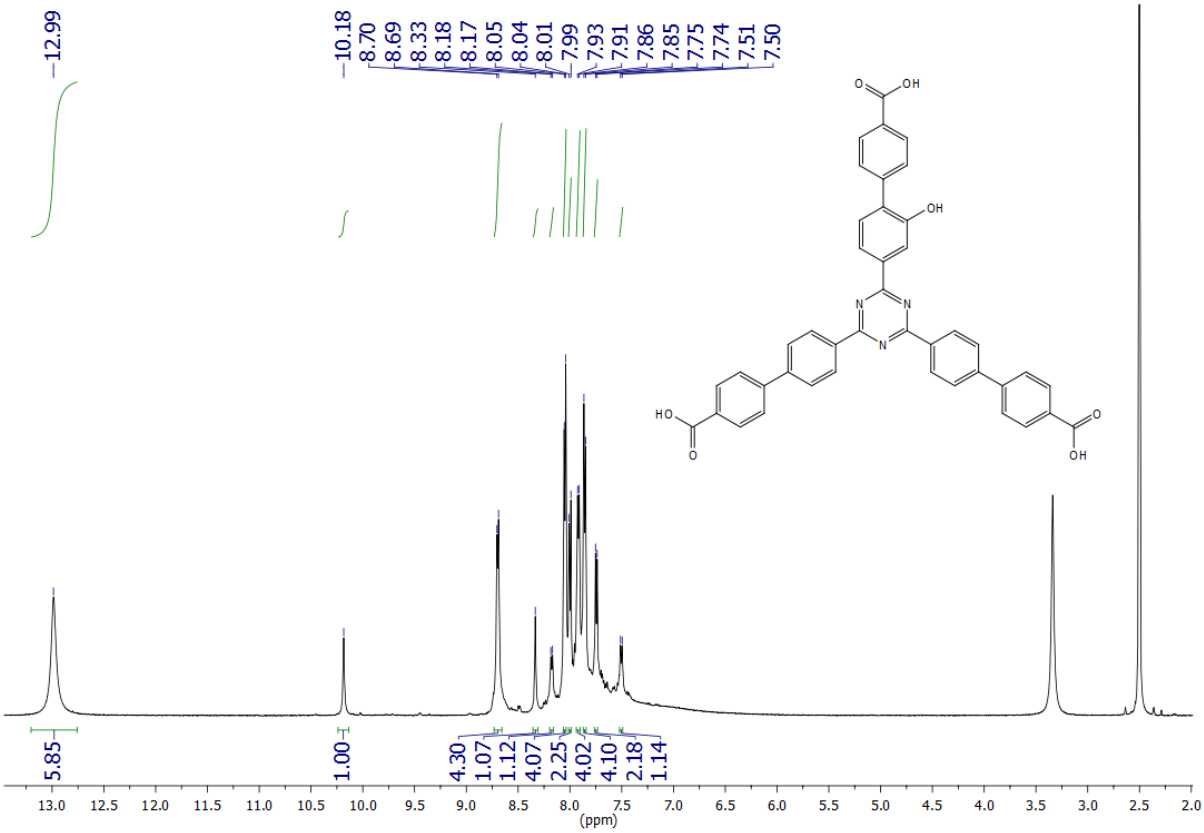
12.99
10.18
8.70
8.69
8.33
8.18
8.17
8.05
8.04
8.01
7.99
7.93
7.91
7.86
7.85
7.75
7.74
7.51
7.50
5.85
1.00
4.30
1.07
1.12
4.07
2.25
4.02
4.10
2.18
1.14
(ppm)

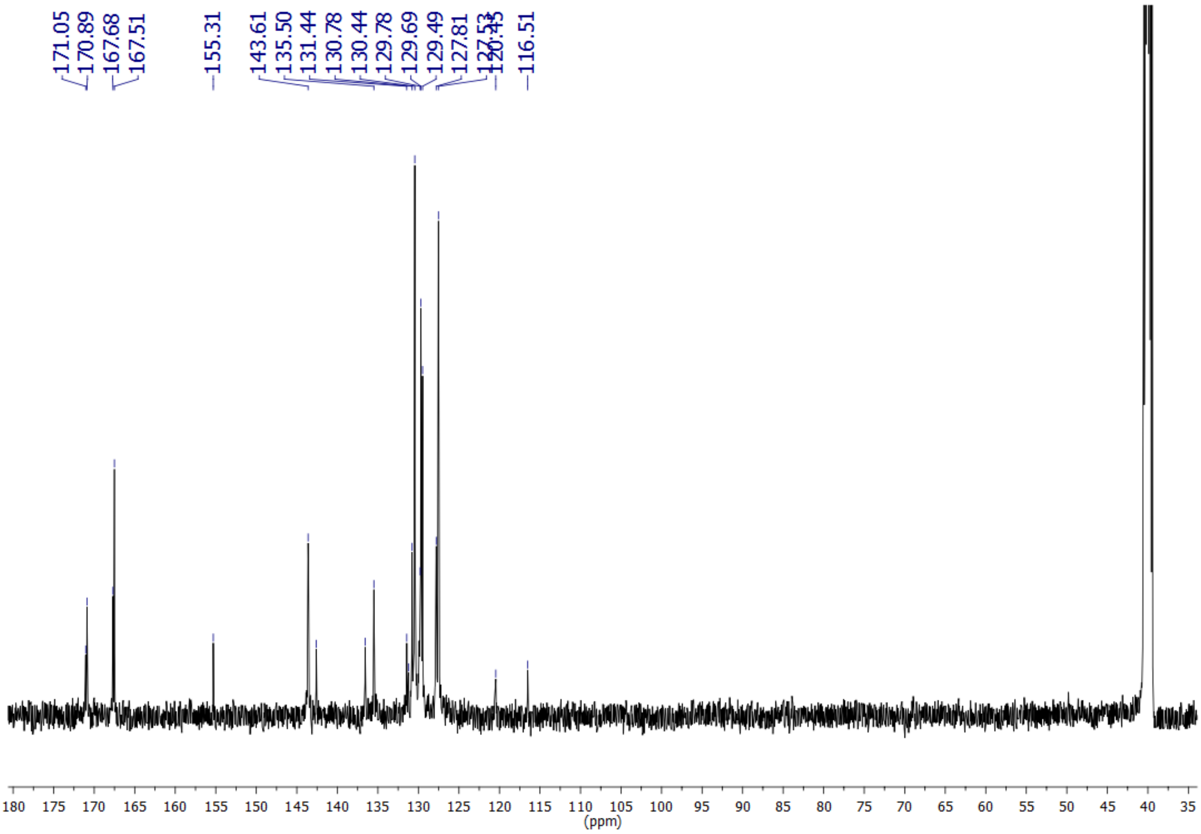
171.05
170.89
167.68
167.51
155.31
143.61
135.50
131.44
130.78
130.44
129.78
129.69
129.49
127.81
(ppm)

2,4,6-Tris[4''-(methoxycarbonyl)-1,1':4',1''-terphenyl-4-yl]-1,3,5-triazine (19a)

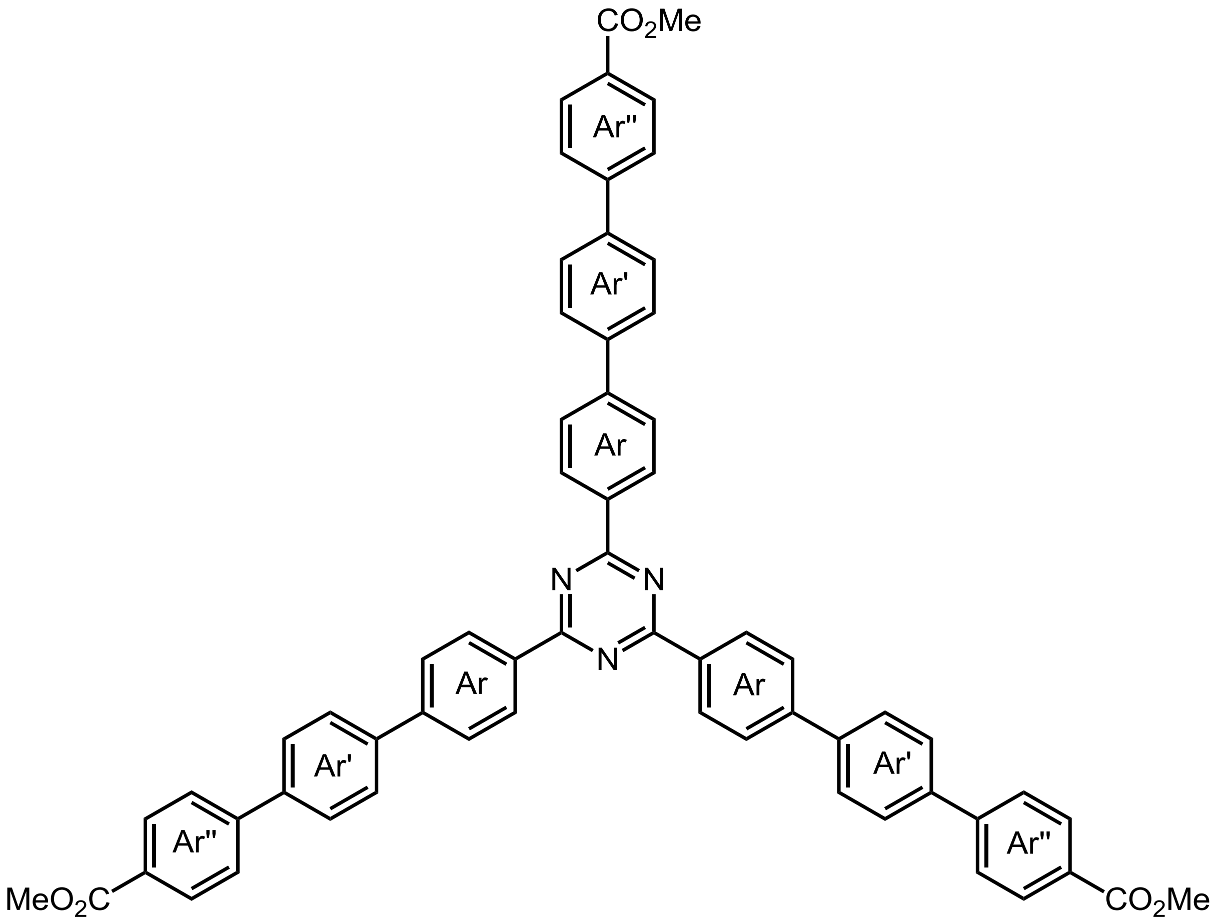

A suspension of 2,4,6-tris(4-bromophenyl)-1,3,5-triazine (**3a**, 413 mg, 758 µmol), methyl 4'-(4,4,5,5-tetramethyl-1,3,2-dioxaborolan-2-yl)-1,1'-biphenyl-4-carboxylate (**18**, 1.00 g, 2.96 mmol)**,** potassium phosphate (1.44 g, 6.82 mmol) and tetrakis(triphenylphosphine)palladium(0) (79 mg, 68 µmol) in a mixture of 1,4-dioxane (70 mL) and deionized water (15 mL) was stirred under nitrogen for 2 d at 120 °C. Deionized water (50 mL) was added to the reaction mixture. The precipitate was filtered off and washed with water. The crude product was recrystallized from chloroform. A colourless solid was obtained. Yield: 174 mg (185 µmol, 24%). M. p.: > 300 °C. ^{1}H NMR (600 MHz, $CDCl_{3,}$): δ = 8.90 (d, ^{3}J = 8.3 Hz, 6H, Ar-*H*-3,5), 8.15 (d, ^{3}J = 8.3 Hz, 6H, Ar''-*H*-3,5), 7.88 (d, ^{3}J = 8.3 Hz, 6H, Ar-*H*-2,6), 7.83 (d, ^{3}J = 8.3 Hz,

6H, Ar'-*H*-2,6), 7.78 (d, ^3J = 8.3 Hz, 6H, Ar'-*H*-3,5), 7.74 (d, ^3J = 8.3 Hz, 6H, Ar''-*H*-2,6), 3.97 (s, 9H, CO_2CH_3) ppm. ^{13}C NMR (150 MHz, $CDCl_3$): δ = 171.4 (s, triazine-*C*-2,4,6), 167.0 (s, CO_2CH_3), 145.0 (s, Ar''-*C*-1), 144.4 (s, Ar-*C*-1), 140.1 (s, Ar'-*C*-1), 139.6 (s, Ar'-*C*-4), 135.5 (s, Ar-*C*-4), 130.2 (d, Ar''-*C*-3,5), 129.6 (d, Ar-*C*-3,5), 129.1 (s, Ar''-*C*-4), 127.8 (d, Ar'-*C*-2,3,5,6), 127.3 (d, Ar-*C*-2,6), 127.0 (s, Ar''-*C*-2,6), 52.2 (q, CH_3) ppm. MS (MALDI, Cl-CCA): *m/z* = 940 $[M + H]^+$. IR (ATR): $\tilde{\nu}$ = 2950 (CH-val.), 1716 (C=O), 1606, 1581, 1552, 1505 (arom. C=C, arom. C=N), 1434 (CH-Def.), 1361 (C-N-val.), 1272, 1184, 1108 (C-O), 808, 773 (1,4-disubst. aryl) cm^{-1}. Elemental analysis ($C_{63}H_{45}N_3O_6$) (939.33): calcd. C 80.49 H 4.82 N 4.47; found C 80.15 H 4.74 N 4.27.

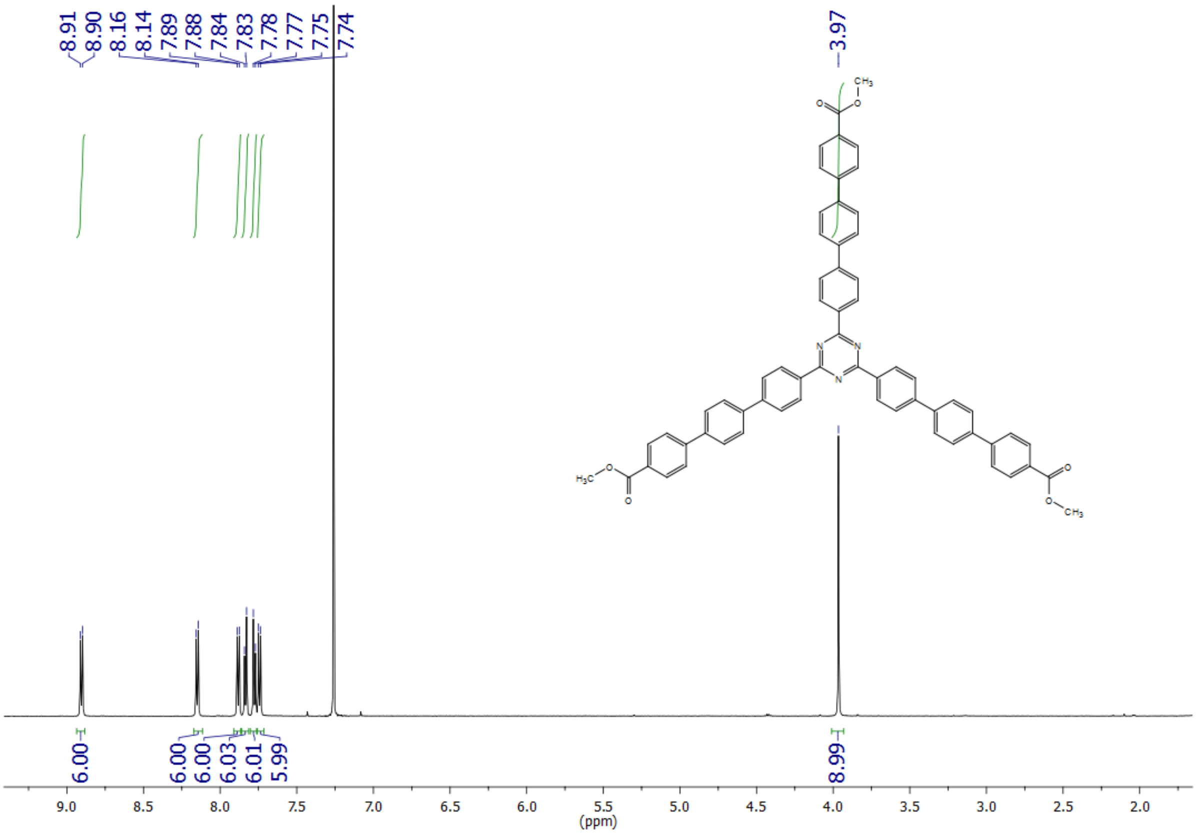
8.91
8.90
8.16
8.14
7.89
7.88
7.84
7.83
7.78
7.77
7.75
7.74
3.97
6.00
6.00
6.00
6.03
6.01
5.99
8.99
(ppm)

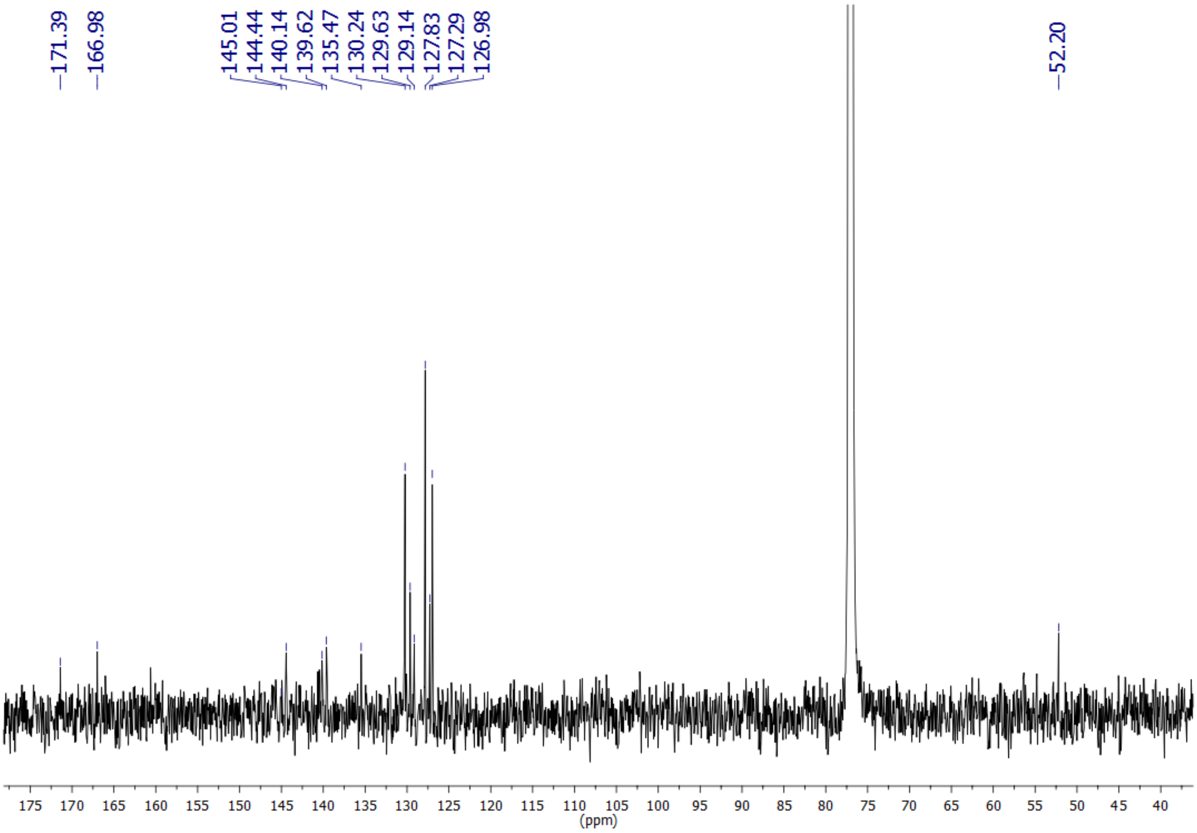
171.39
166.98
145.01
144.44
140.14
139.62
135.47
130.24
129.63
129.14
127.83
127.29
126.98
52.20
(ppm)

2-[4''-(Methoxycarbonyl)-2-nitro-1,1':4',1''-terphenyl-4-yl]-4,6-bis[4''-(methoxycarbonyl)-1,1':4',1''-terphenyl-4-yl]-1,3,5-triazine (19b)

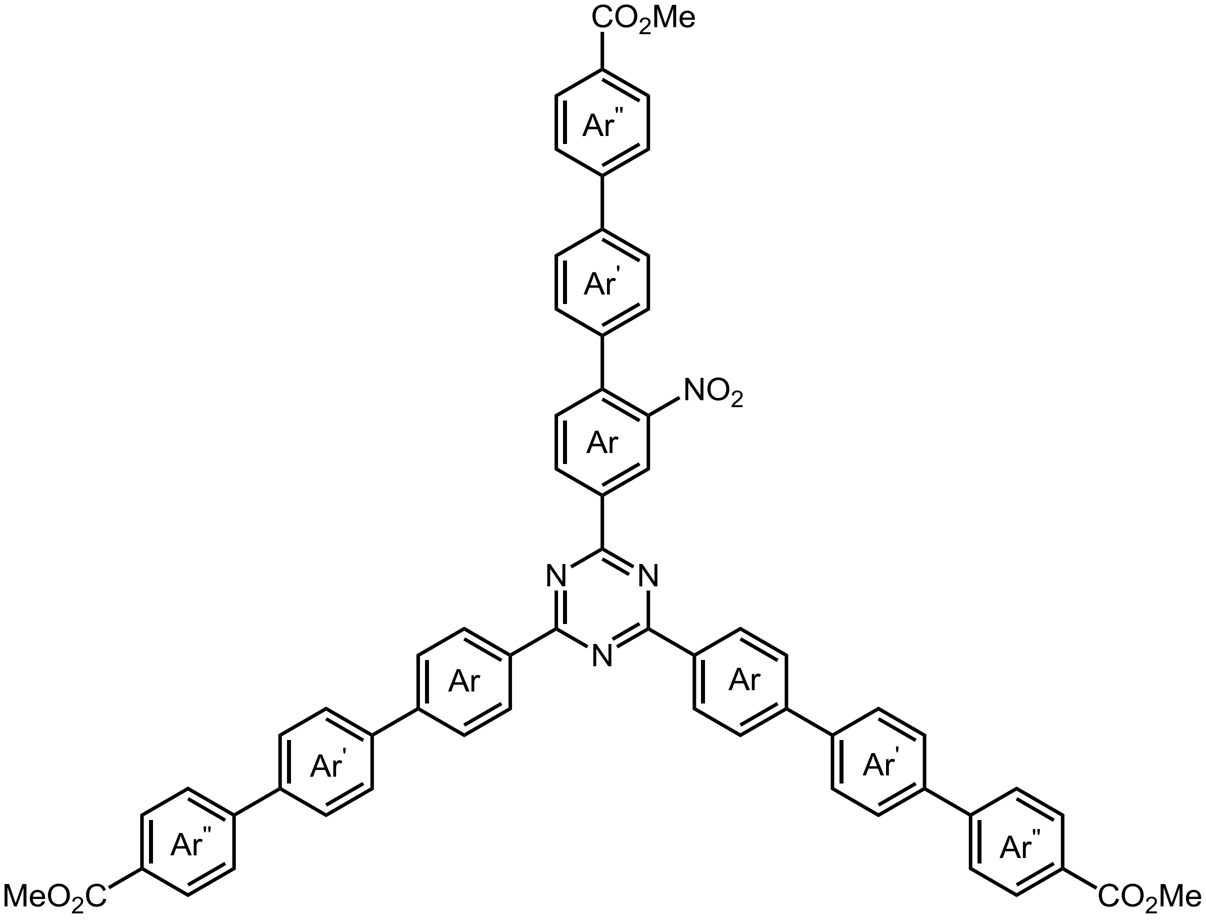

A mixture of 2-(4-bromo-3-nitrophenyl)-4,6-bis(4-bromophenyl)-1,3,5-triazine (**3b**, 500 mg, 846 µmol), methyl 4'-(4,4,5,5-tetramethyl-1,3,2-dioxaborolan-2-yl)-1,1'-biphenyl-4-carboxylate (**18**, 1.12 g, 3.30 mmol), potassium phosphate (1.62 g, 7.61 mmol) and [1,1′-bis(diphenylphosphino)ferrocene]dichloropalladium(II) (62.0 mg, 84.6 µmol) in a mixture of 1,4-dioxane (100 mL) and deionized water (10 mL) was stirred under nitrogen for 3 d at 120 °C. The solvent was evaporated in vacuo, the residue was dissolved in chloroform (2 L) and washed with deionized water (3 x 250 mL) and brine (250 mL). The organic layer was evaporated in vacuo until 1 L was left. Charcoal (5 g) was added and the solution was heated to 80 °C. The hot suspension was filtered through celite and the colourless filtrate was evaporated in vacuo. After

the addition ethyl acetate (50 mL), the undissolved solid was filtrated and dried to obtain the slightly yellow product. Yield: 132 mg (134 µmol, 16%). M. p.: > 300 °C. ^{1}H NMR (500 MHz, $CDCl_3$): δ = 9.28 (d, ^{4}J = 1.4 Hz, 1H, 2-Ar-*H*-3), 9.06 (dd, ^{3}J = 8.1 Hz, ^{4}J = 1.4 Hz, 1H, 2-Ar-*H*-5), 8.90 (d, ^{3}J = 8.5 Hz, 4H, 4,6-Ar-*H*-3,5), 8.15 (d, ^{3}J = 8.5 Hz, 6H, 2,4,6-Ar''-*H*-3,5), 7.90 (d, ^{3}J = 8.5 Hz, 4H, 4,6-Ar-*H*-2,6), 7.84 (d, ^{3}J = 8.5 Hz, 4H, 4,6-Ar'-*H*-2,6), 7.78 (d, ^{3}J = 8.5 Hz, 4H, 4,6-Ar'-*H*-3,5), 7.77 – 7.70 (m, 9H, 2,4,6-Ar''-*H*-2,6, 2-Ar-*H*-6, 2-Ar'-*H*-3,5), 7.53 (d, ^{3}J = 8.3 Hz, 2H, 2-Ar'-*H*-2,6), 3.97 (s, 9H, CO_2CH_3) ppm. ^{13}C NMR (125 MHz, $CDCl_3$): δ = 171.5 (s, triazine-*C*-4,6), 169.5 (s, triazine-*C*-2), 166.9 (s, CO_2CH_3), 149.9 (s, 2-Ar-*C*-2), 144.9 (s, 4,6-Ar-*C*-1), 144.8 (s, 2,4,6-Ar''-*C*-1), 140.5 (s, 2-Ar'-*C*-4), 139.8 (s, 4,6-Ar'-*C*-1), 139.7 (s, 4,6-Ar'-*C*-4), 139.1 (s, 2-Ar-*C*-1), 137.6 (s, 2-Ar-*C*-4), 136.5 (s, 2-Ar'-*C*-1), 134.9 (s, 4,6-Ar-*C*-4), 132.4 (d, 2-Ar-*C*-6), 132.0 (d, 2-Ar-*C*-5), 130.2 (d, 2,4,6-Ar''-*C*-3,5), 129.7 (d, 4,6-Ar-*C*-3,5), 129.1 (s, 2,4,6-Ar''-*C*-4), 128.4 (d, 2-Ar'-*C*-2,6), 127.9 (d, 4,6-Ar'-*C*-3,5), 127.8 (d, 4,6-Ar'-*C*-2,6), 127.4 (d, 4,6-Ar-*C*-2,6), 127.1 (d, 2-Ar'-*C*-3,5), 126.9 (d, 2,4,6-Ar''-*C*-2,6), 124.6 (d, 2-Ar-*C*-3), 52.1 (q, CO_2CH_3) ppm. MS (MALDI, Cl-CCA): *m/z* = 986 $[M + H]^+$. IR (ATR): $\tilde{\nu}$ = 2952 (CH-val.), 1718 (C=O), 1606, 1572 (arom. C=C, arom. C=N), 1508 (asymm. N=O), 1400, 1433 (CH-def.), 1357 (symm. N=O), 1274, 1184, 1108 (C-O), 810, 771 (1,4-disubst. aryl, 1,2,4-trisubst. aryl) cm^{-1}. Elemental analysis ($C_{63}H_{44}N_4O_8$) (985.05): calcd. C 76.82 H 4.50 N 5.69; found C 77.04 H 4.83 N 5.38.

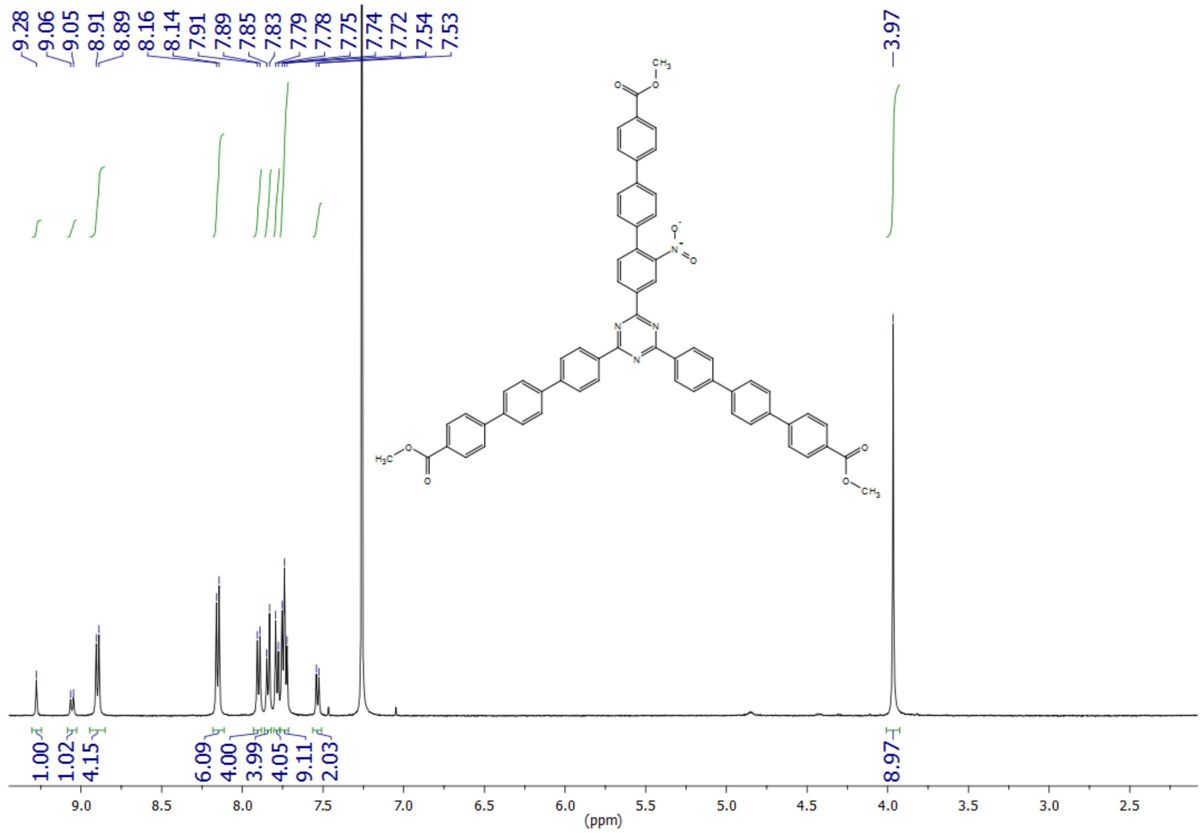
9.28
9.06
9.05
8.91
8.89
8.16
8.14
7.91
7.89
7.85
7.83
7.79
7.78
7.75
7.74
7.72
7.54
7.53
3.97
1.00
1.02
4.15
6.09
4.00
3.99
4.05
9.11
2.03
8.97
(ppm)

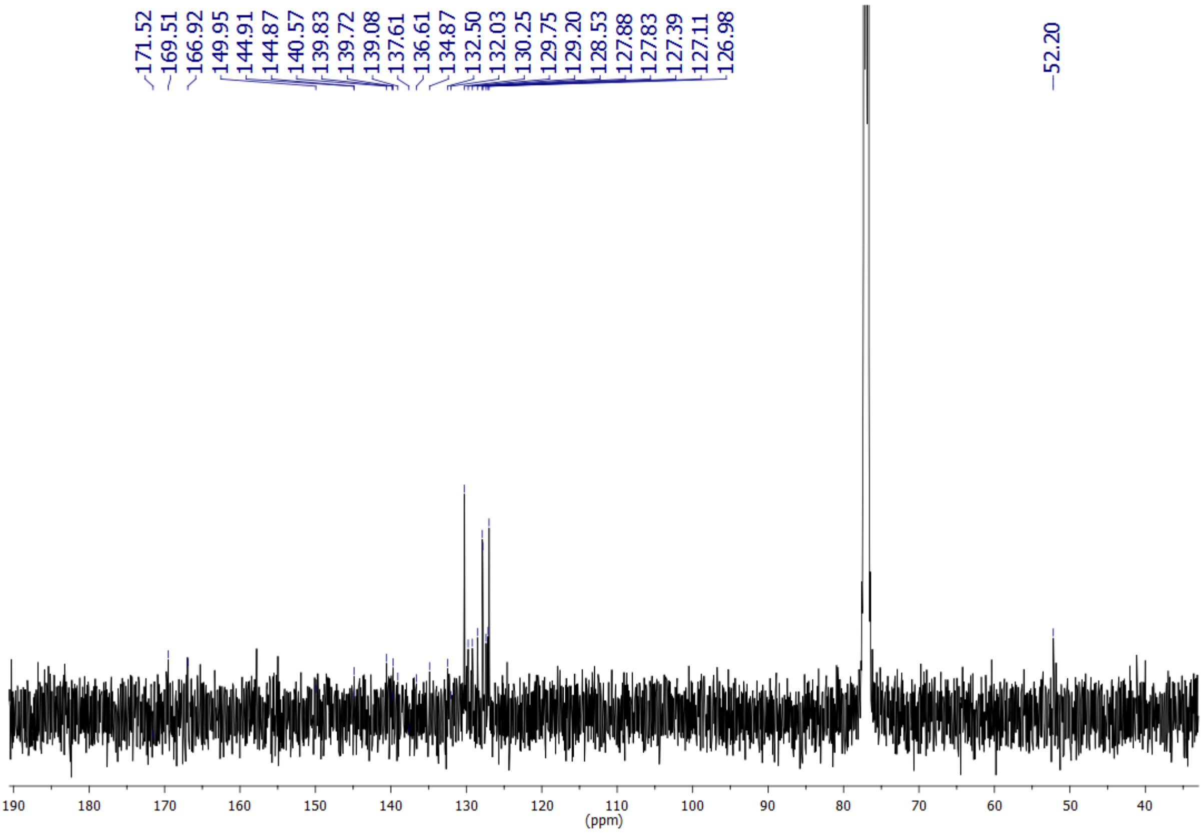
171.52
169.51
166.92
149.95
144.91
144.87
140.57
139.83
139.72
139.08
137.61
136.61
134.87
132.50
132.03
130.25
129.75
129.20
128.53
127.88
127.83
127.39
127.11
126.98
52.20
(ppm)

2,4-Bis[4''-(methoxycarbonyl)-1,1':4',1''-terphenyl-4-yl]-6-[2-methoxy-4''-(methoxycarbonyl)-1,1':4',1''-terphenyl-4-yl]-1,3,5-triazine (19c)

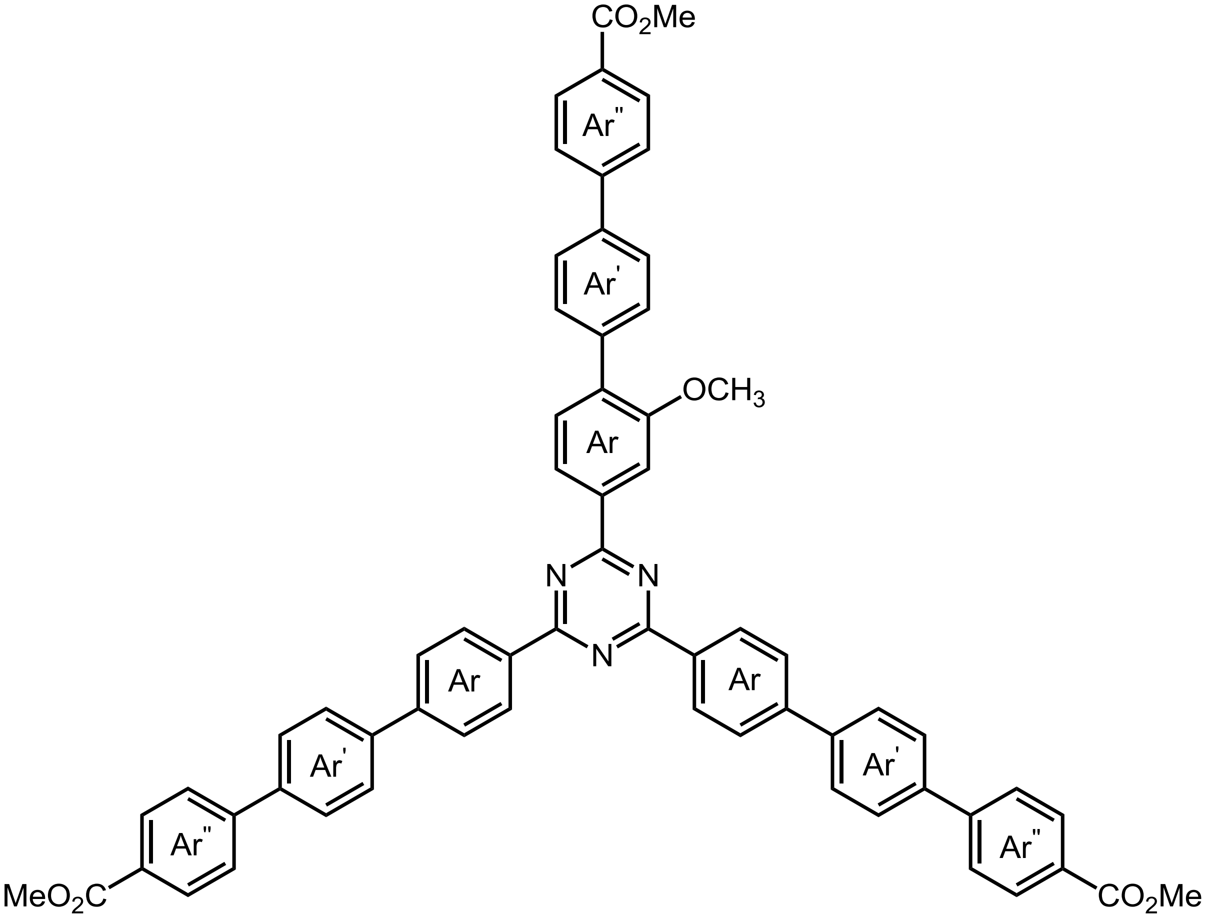

A mixture of 2-(4-bromo-3-methoxyphenyl)-4,6-bis(4-bromophenyl)-1,3,5-triazine (**3c**, 102 mg, 180 µmol), methyl 4'-(4,4,5,5-tetramethyl-1,3,2-dioxaborolan-2-yl)-1,1'-biphenyl-4-carboxylate (**18**, 269 mg, 796 µmol), potassium phosphate (259 mg, 1.22 mmol) and tetrakis(triphenylphosphine)palladium(0) (19.4 mg, 17.0 µmol) in a mixture of 1,4-dioxane (15 mL) and deionized water (1 mL) was stirred under nitrogen for 3 d at 100 °C. The solvent was evaporated in vacuo, the residue was dissolved in chloroform (50 mL) and washed with deionized water (3 x 25 mL) and brine (25 mL). The organic layer was dried with magnesium sulfate and the solvent was evaporated in vacuo. The crude product was recrystallized from a mixture of toluene and *n*-heptane. A colourless solid was obtained. Yield: 140 mg (144 µmol,

81%). M. p.: > 300 °C. ^{1}H NMR (500 MHz, $CDCl_3$): δ = 8.87 (d, ^3J = 8.4 Hz, 4H, 2,4-Ar-*H*-3,5), 8.51 (dd, ^3J = 7.9 Hz, ^4J = 1.5 Hz, 1H, 6-Ar-*H*-5), 8.44 (d, ^4J = 1.5 Hz, 1H, *6*-Ar-*H*-3), 8.14 (d, ^3J = 8.4 Hz, 4H, 2,4-Ar''-*H*-3,5), 8.13 (d, ^3J = 8.4 Hz, 2H, 6-Ar''-*H*-3,5), 7.87 (d, ^3J = 8.4 Hz, 4H, 2,4-Ar-*H*-2,6), 7.82 (d, ^3J = 8.4 Hz, 4H, 2,4-Ar'-*H*-2,6), 7.78 – 7.71 (m, 14H, 2,4,6-Ar'-*H*-3,5, 6-Ar'-*H*-2,6, 2,4,6-Ar''-*H*-2,6), 7.59 (d, ^3J = 7.9 Hz, 1H, 6-Ar-*H*-6), 4.08 (s, 3H, OCH_3), 3.962 (s, 6H, 2,4-Ar''-CO_2CH_3), 3.960 (s, 3H, 6-Ar''-CO_2CH_3) ppm. ^{13}C NMR (125 MHz, $CDCl_3$): δ = 171.32 (s, triazine-*C*-2,4), 171.29 (s, triazine-*C*-6), 167.0 (s, 6-Ar''-CO_2CH_3), 166.9 (s, 2,4-Ar''-CO_2CH_3), 156.9 (s, 6-Ar-*C*-2), 145.3 (s, 6-Ar''-*C*-1), 144.9 (s, 2,4-Ar''-*C*-1), 144.4 (s, 2,4-Ar''-*C*-4), 140.1 (s, 2,4-Ar-*C*-1), 139.6 (s, 2,4-Ar'-*C*-4), 139.0 (s, 2,4-Ar'-*C*-1), 136.9 (s, 6-Ar'-*C*-1), 135.8 (s, 6-Ar-*C*-4), 135.3 (s, 2,4-Ar-*C*-4), 134.2 (s, 6-Ar-*C*-1), 130.9 (d, 6-Ar-*C*-6), 130.21 (d, 2,4-Ar''-*C*-3,5), 130.16 (d, 6-Ar''-*C*-3,5), 130.1 (d, 2,4-Ar-*C*-3,5), 129.0 (s, 6-Ar''-*C*-4), 128.2 (d, 6-Ar'-*C*-2,6), 127.8 (d, 2,4-Ar'-*C*-3,5), 127.76 (d, 2,4-Ar'-*C*-2,6), 127.2 (d, 2,4-Ar-*C*-2,6), 127.0 (d, 6-Ar'-*C*-3,5), 126.9 (d, 2,4,6-Ar''-*C*-2,6, 6-Ar'-*C*-4), 122.0 (d, 6-Ar-*C*-5), 111.3 (d, 6-Ar-*C*-3), 55.9 (q, OCH_3), 52.2 (q, 2,4-Ar''-CO_2CH_3), 52.1 (q, 6-Ar''-CO_2CH_3) ppm. MS (MALDI, Cl-CCA): *m/z* = 971 $[M + H]^+$. IR (ATR): $\tilde{\nu}$ = 3030, 3001 (aryl-H), 2949, 2842 (CH-val.), 1715 (C=O), 1605, 1582, 1505, 1403 (arom. C=C, arom. C=N), 1434 (CH-Def.), 1360 (C-N-val.), 1272 (Aryl-OCH_3), 807 (1,4-disubst. aryl, 1,2,4-trisubst. aryl) cm^{-1}. Elemental analysis ($C_{64}H_{47}N_3O_7$) (970.07): calcd. C 79.24 H 4.88 N 4.33; found C 79.10 H 5.11 N 4.02.

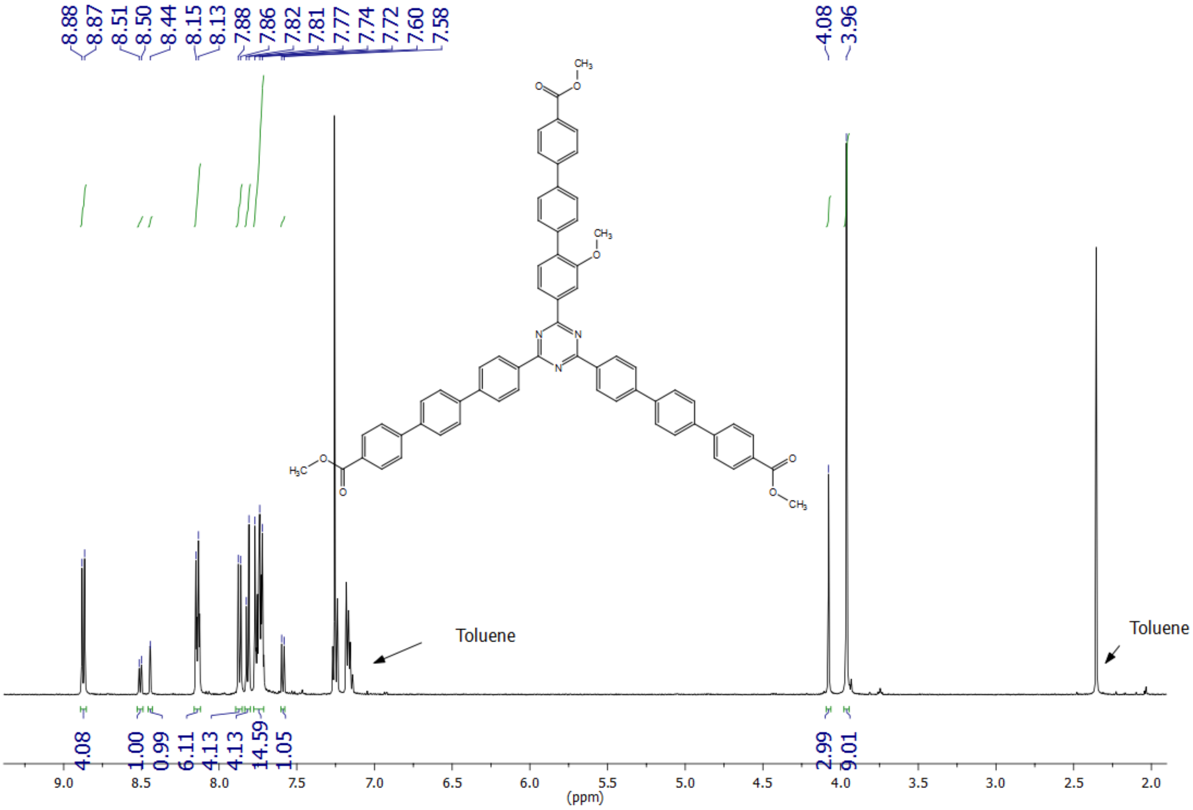
8.88
8.87
8.51
8.50
8.44
8.15
8.13
7.88
7.86
7.82
7.81
7.77
7.74
7.72
7.60
7.58
4.08
3.96
Toluene
Toluene
4.08
1.00
0.99
6.11
4.13
4.13
14.59
1.05
2.99
9.01
(ppm)

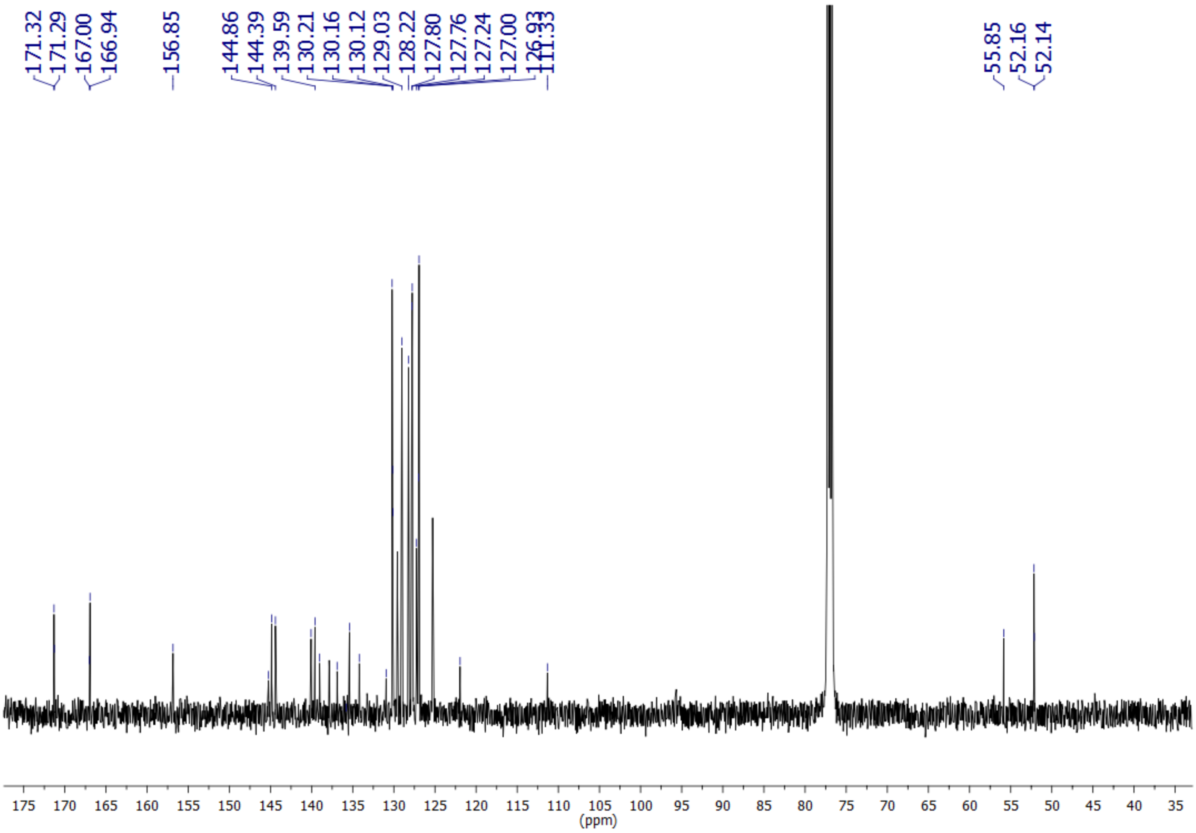
171.32
171.29
167.00
166.94
156.85
144.86
144.39
139.59
130.21
130.16
130.12
129.03
128.22
127.80
127.76
127.24
127.00
126.93
111.33
55.85
52.16
52.14
(ppm)

2,4,6-Tris(4''-carboxy-1,1':4',1''-terphenyl-4-yl)-1,3,5-triazine (20a)

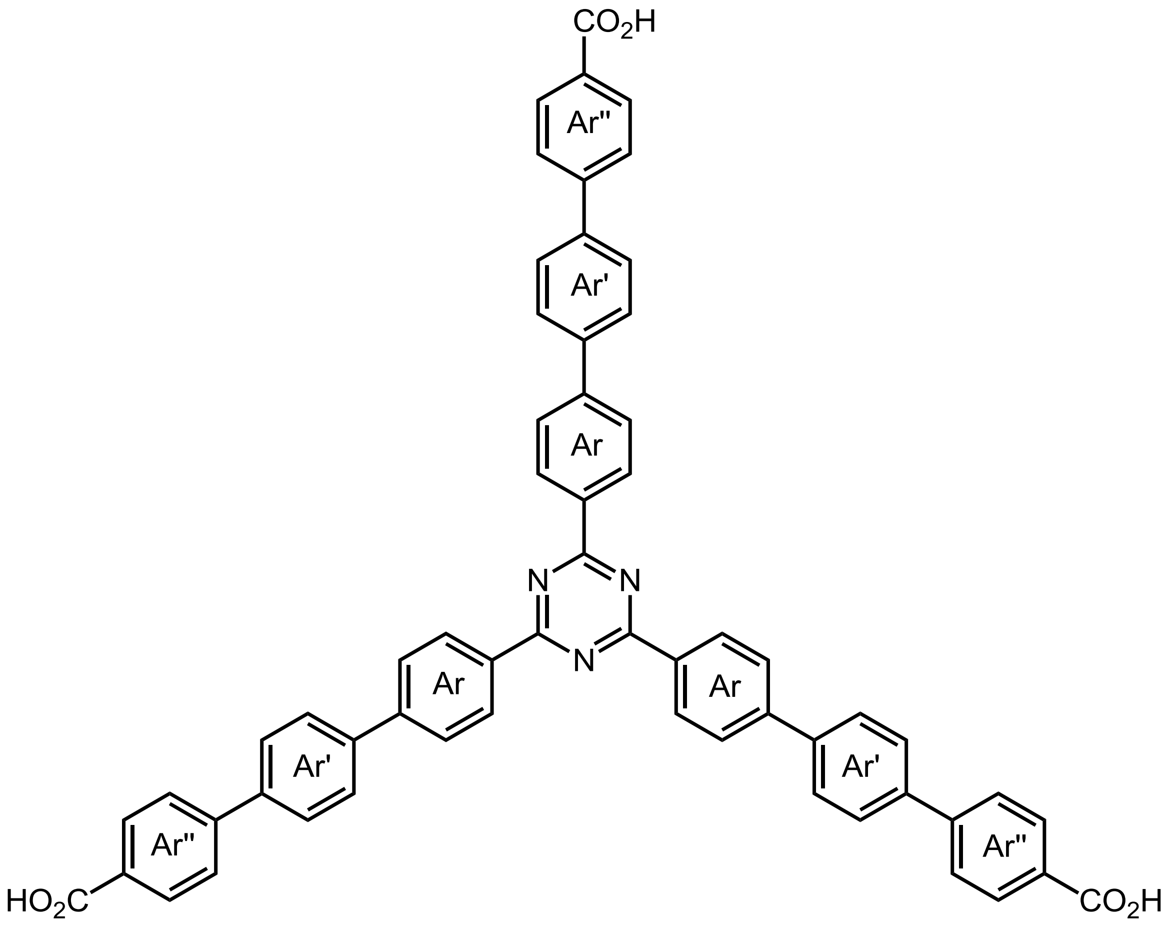

Lithium hydroxide monohydrate (733 mg, 17.5 mmol) was added to 2,4,6-tris[4''-(methoxycarbonyl)-1,1':4',1''-terphenyl-4-yl]-1,3,5-triazine (**19a**, 587 mg, 624 µmol) in a mixture of tetrahydrofuran (100 mL) and deionized water (10 mL). After stirring for 24 h at 120 °C, the solvent was evaporated in vacuo and enough sodium hydroxide (2 M) was added to the residue to dissolved it. Then it was acidified with conc. hydrochloric acid. The precipitate was filtered off and washed with deionized water and chloroform. A white solid was obtained. Yield: 555 mg (618 µmol, 99%). M. p.: > 300 °C. ^{1}H NMR (600 MHz, DMSO-d$_6$): δ = 8.89 (d, 6H, ^3J = 8.1 Hz, Ar-*H*-3,5), 8.10 – 8.05 (m, 12H, Ar''-*H*-3,5, Ar-*H*-2,6), 7.99 (d, ^3J = 8.3 Hz, 6H, Ar'-*H*-2,6), 7.94 (d, ^3J = 8.3 Hz, 6H, Ar'-*H*-3,5), 7.91 (d, ^3J = 7.9 Hz, 6H, Ar''-*H*-2,6) ppm. ^{13}C NMR (150 MHz, DMSO-d$_6$): δ = 171.2 (s, triazine-*C*-2,4,6), 167.6 (s, *C*OOH), 144.1 (s, Ar-*C*-1),

143.8 (s, Ar''-*C*-1), 139.3 (s, 6-Ar'-*C*-4), 139.2 (s, Ar'-*C*-1), 135.0 (s, Ar-*C*-4), 130.6 (s, Ar''-*C*-4),130.5 (d, Ar''-*C*-3,5), 129.9 (d, Ar-*C*-3,5), 128.1 (d, Ar'-*C*-2,3,5,6), 127.6 (d, Ar-*C*-2,6), 127.2 (s, Ar''-*C*-2,6) ppm. MS (MALDI, Cl-CCA): *m/z* = 898 $[M + H]^+$. IR (ATR): $\tilde{\nu}$ = 1683 (C=O), 1605, 1580, 1504 (arom. C=C, arom. C=N), 1404 (CH-Def.), 1359 (C-N-val.), 1283, 1182 (C-O), 808, 773 (1,4-disubst. aryl) cm^{-1}. Elemental analysis ($C_{60}H_{39}N_3O_6$) (897.28): calcd. C 80.25 H 4.38 N 4.68; ($C_{60}H_{39}N_3O_6 \cdot 0.66 H_2O$) (909.29): calcd. C 79.19 H 4.47 N 4.62; found C 78.97 H 4.57 N 4.66.

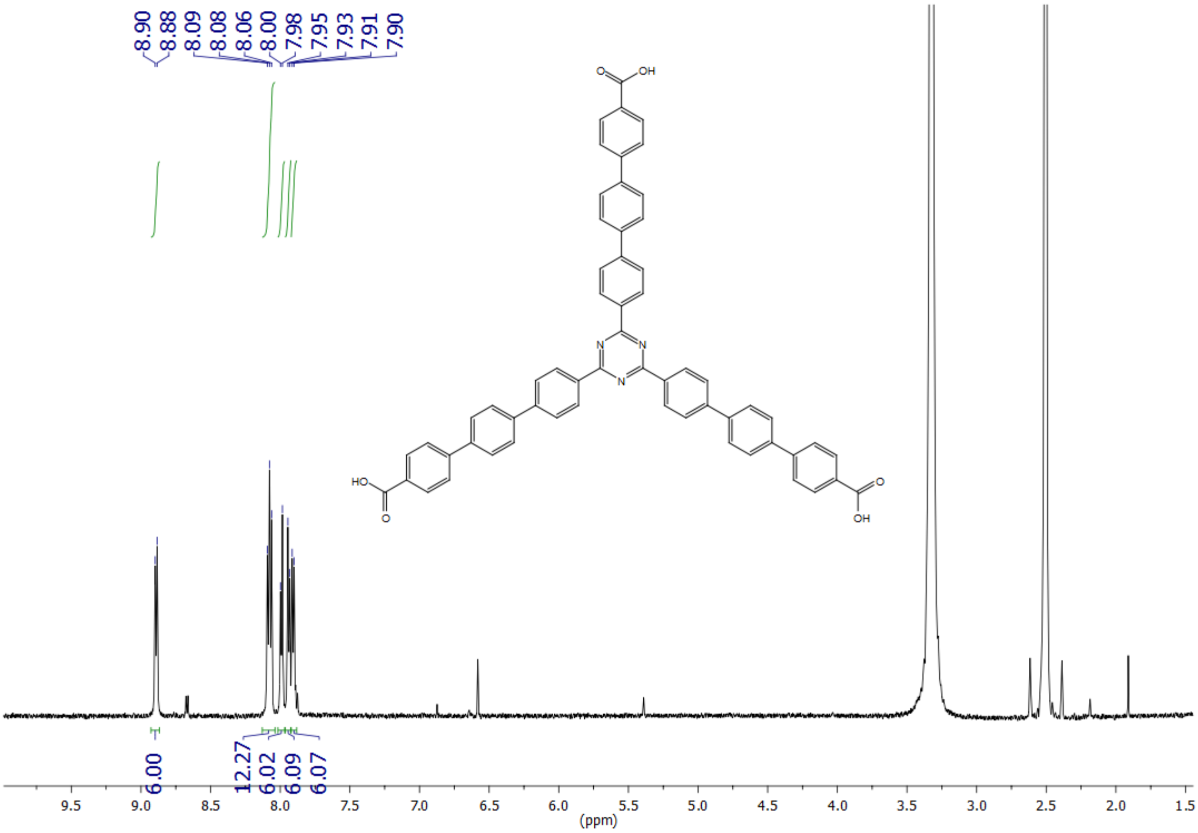

8.90
8.88
8.09
8.08
8.06
8.00
7.98
7.95
7.93
7.91
7.90
6.00
12.27
6.02
6.09
6.07
(ppm)

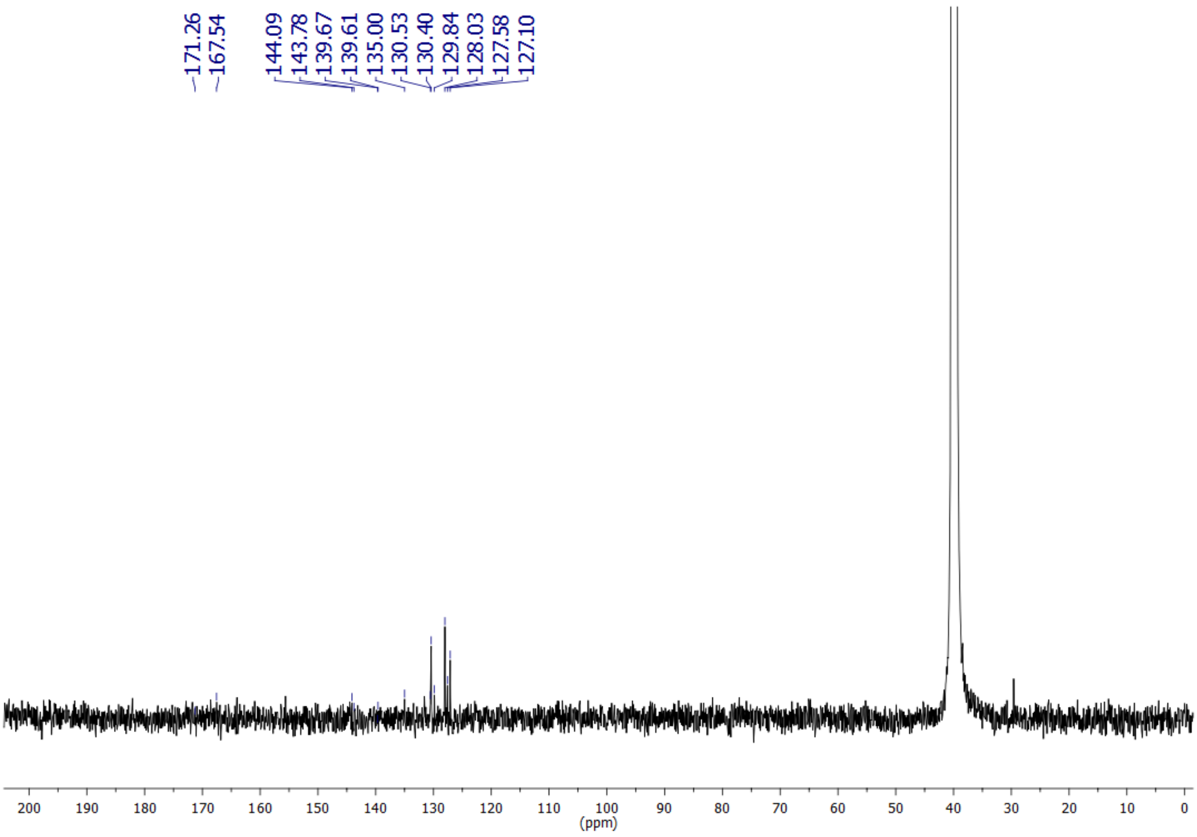

171.26
167.54
144.09
143.78
139.67
139.61
135.00
130.53
130.40
129.84
128.03
127.58
127.10
(ppm)

2-(4''-Carboxy-2-nitro-1,1',4',1''-terphenyl-4-yl)-4,6-bis(4''-carboxy-1,1':4',1''-terphenyl-4-yl)-1,3,5-triazine (20b)

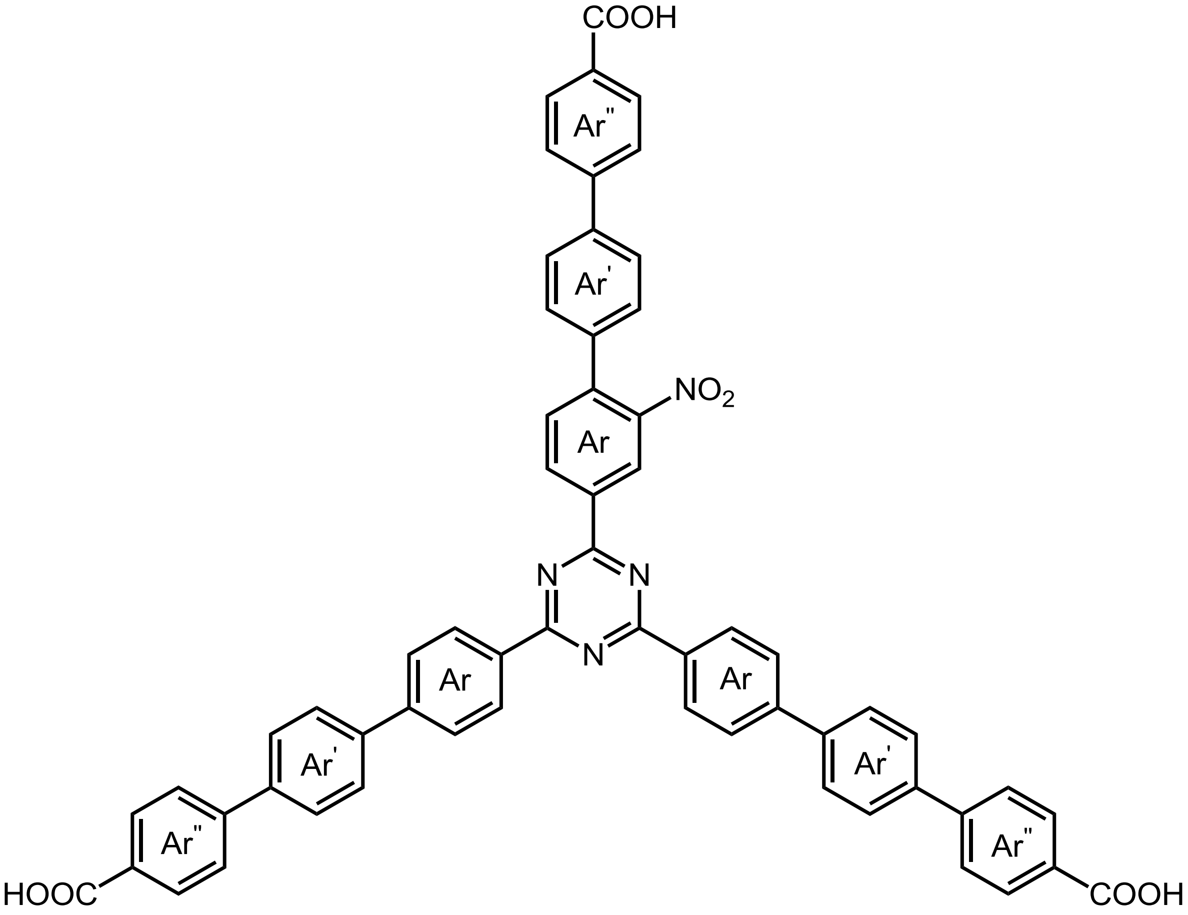

To a mixture of 1,4-dioxane (10 mL) and sodium hydroxide (6 M, 50 mL) was added 2-[4''-(methoxycarbonyl)-2-nitro-1,1':4',1''-terphenyl-4-yl]-4,6-bis[4''-(methoxycarbonyl)-1,1':4',1''-terphenyl-4-yl]-1,3,5-triazine (**19b**, 93 mg, 94 µmol). After stirring for 24 h at 120 °C the solvent was evaporated in vacuo. The residue was dissolved in 10 mL water and acidified with conc. hydrochloric acid. The precipitate was filtered off and washed with deionized water. A yellow solid was obtained. Yield: 70 mg (74 µmol, 79%). M. p.: > 300 °C. ^{1}H NMR (600 MHz, DMSO-d_6): δ = 13.0 (br.s, 3H, COO*H*), 9.27 (d, ^{4}J = 1.3 Hz, 1H, 2-Ar-*H*-2), 9.10 (d, ^{3}J = 8.0 Hz, 1H, 2-Ar-*H*-5), 8.90 (d, ^{3}J = 8.3 Hz, 4H, 4,6-Ar-*H*-3,5), 8.12 – 8.04 (m, 10H, 2,4,6-Ar''-*H*-3,5, 4,6-Ar-*H*-2,6), 7.98 (d, ^{3}J = 8.3 Hz, 4H, 4,6-Ar'-*H*-2,6), 7.96 –

7.88 (m, 13H, 4,6-Ar'-*H*-3,5, 2,4,6-Ar''-*H*-2,6, 2-Ar-*H*-6, 2-Ar'-*H*-3,5), 7.60 (d, ^3J = 8.1 Hz, 2H, 2-Ar'-*H*-2,6) ppm. ^{13}C NMR (150 MHz, DMSO-d_6): δ = 170.9 (s, triazine-*C*-4,6), 169.0 (s, triazine-*C*-2), 167.2 (s, CO_2H), 149.5 (s, 2-Ar-*C*-2), 143.9 (s, 4,6-Ar-*C*-1), 143.3 (s, 2,4,6-Ar''-*C*-1), 139.3 (s, 2-Ar'-*C*-4), 138.8 (s, 4,6-Ar'-*C*-1), 138.7 (s, 4,6-Ar'-*C*-4), 138.0 (s, 2-Ar-*C*-1), 137.5 (s, 2-Ar-*C*-4), 136.3 (s, 2-Ar'-*C*-1), 134.1 (s, 4,6-Ar-*C*-4), 133.7 (d, 2-Ar-*C*-6), 132.6 (d, 2-Ar-*C*-5), 130.4 (d, 2,4,6-Ar''-*C*-3,5), 130.0 (d, 4,6-Ar-*C*-3,5), 129.8 (s, 2,4,6-Ar''-*C*-4), 129.0 (d, 2-Ar'-*C*-2,6), 128.0 (d, 4,6-Ar'-*C*-2,6), 127.9 (d, 4,6-Ar'-*C*-3,5), 127.6 (d, 4,6-Ar-*C*-2,6), 127.3 (d, 2-Ar'-*C*-3,5), 127.3 (d, 2,4,6-Ar''-*C*-2,6), 124.4 (d, 2-Ar-*C*-3) ppm. MS (MALDI, Cl-CCA): *m/z* = 944 $[M + H]^+$. IR (ATR): $\tilde{\nu}$ = 3031 (aryl-H), 1684 (C=O), 1605, 1571 (arom. C=C, arom. C=N), 1506 (asymm. N=O), 1424, 1401 (CH-Def.), 1356 (symm. N=O), 1272, 1182, 1110 (C-O), 811, 773 (1,4-disubst. aryl, 1,2,4-trisubst. aryl) cm^{-1}. Elemental analysis ($C_{60}H_{38}N_4O_8$) (942.97): calcd. C 76.42 H 4.06 N 5.94; ($C_{60}H_{38}N_4O_8 \cdot 0.5\ H_2O$) (951.96): calcd. C 75.70 H 4.13 N 5.89; found C 75.43 H 4.06 N 5.87.

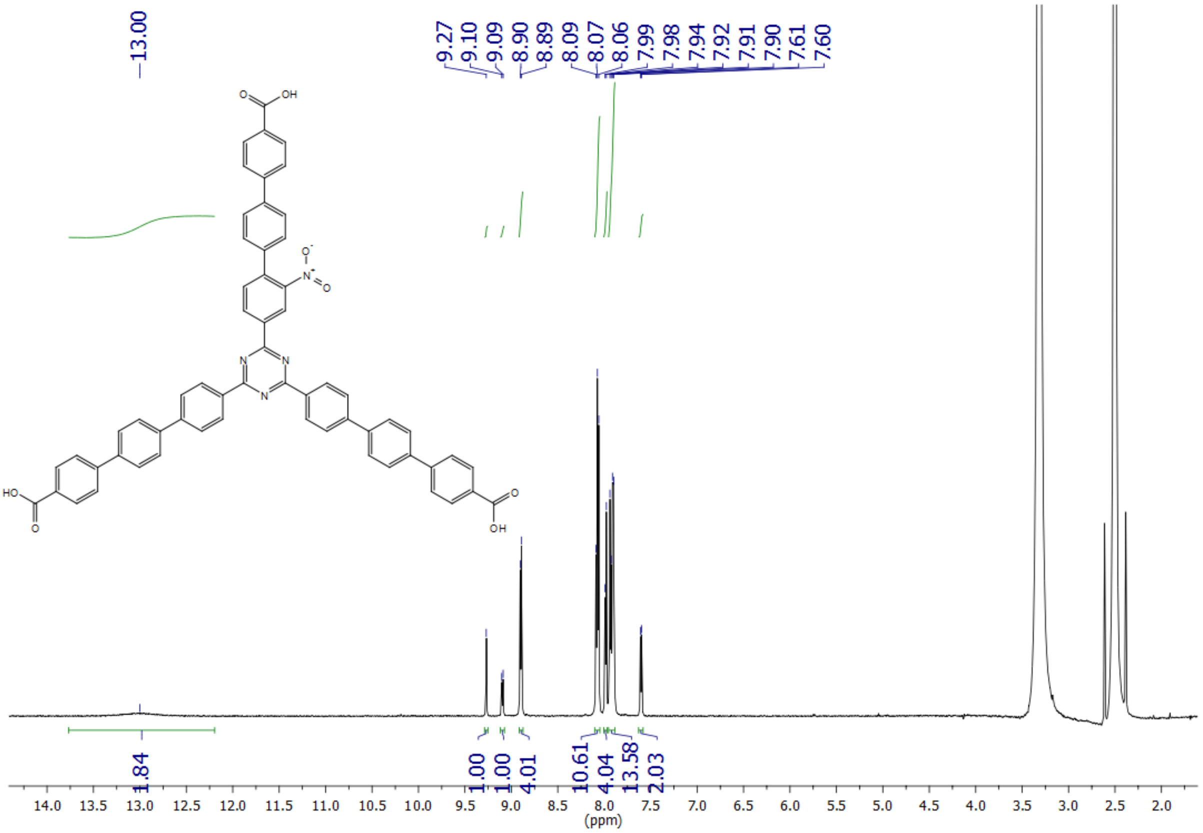

13.00
9.27
9.10
9.09
8.90
8.89
8.09
8.07
8.06
7.99
7.98
7.94
7.92
7.91
7.90
7.61
7.60
OH
O
HO
O
OH
O
N
N
N
O
N
O
1.84
1.00
1.00
4.01
10.61
4.04
13.58
2.03
14.0 13.5 13.0 12.5 12.0 11.5 11.0 10.5 10.0 9.5 9.0 8.5 8.0 7.5 7.0 6.5 6.0 5.5 5.0 4.5 4.0 3.5 3.0 2.5 2.0
(ppm)

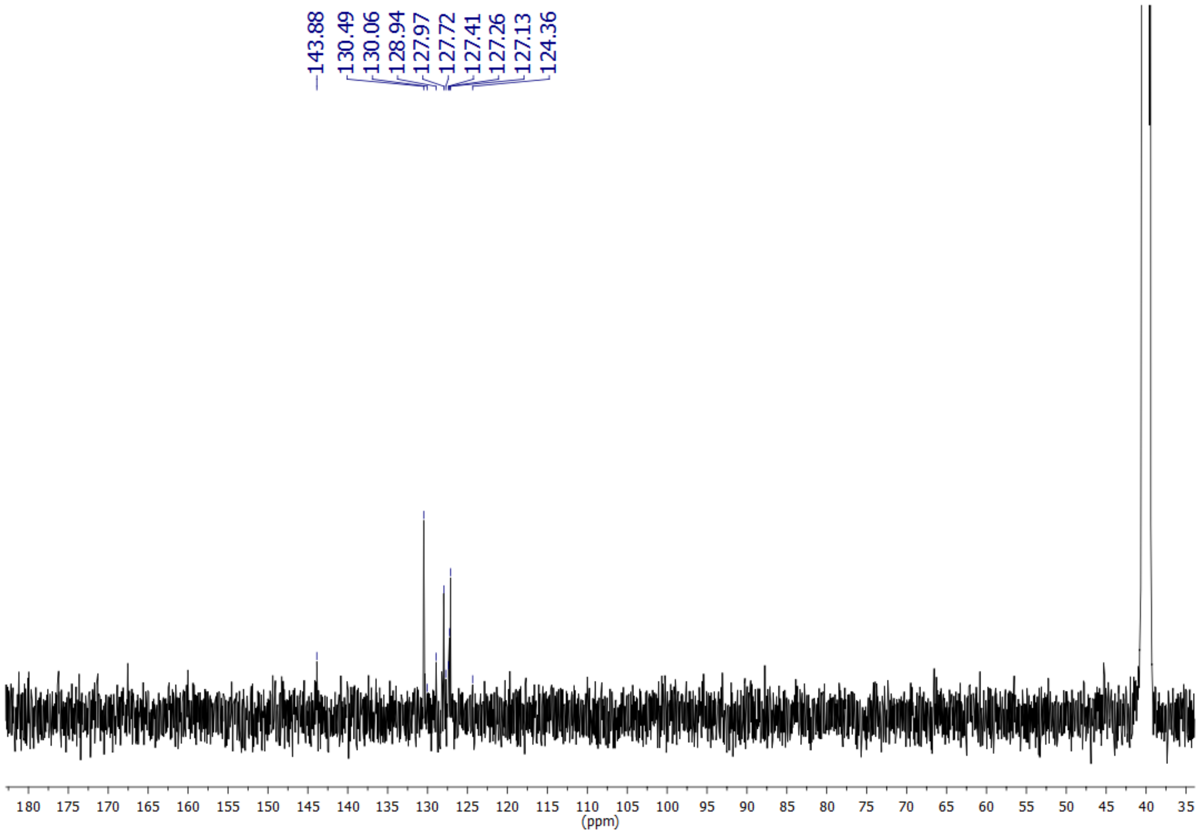

143.88
130.49
130.06
128.94
127.97
127.72
127.41
127.26
127.13
124.36
180 175 170 165 160 155 150 145 140 135 130 125 120 115 110 105 100 95 90 85 80 75 70 65 60 55 50 45 40 35
(ppm)

2-(4''-Carboxy-2-hydroxy-1,1':4',1''-terphenyl-4-yl)-4,6-bis(4''-carboxy-1,1':4',1''-terphenyl-4-yl)-1,3,5-triazine (20e)

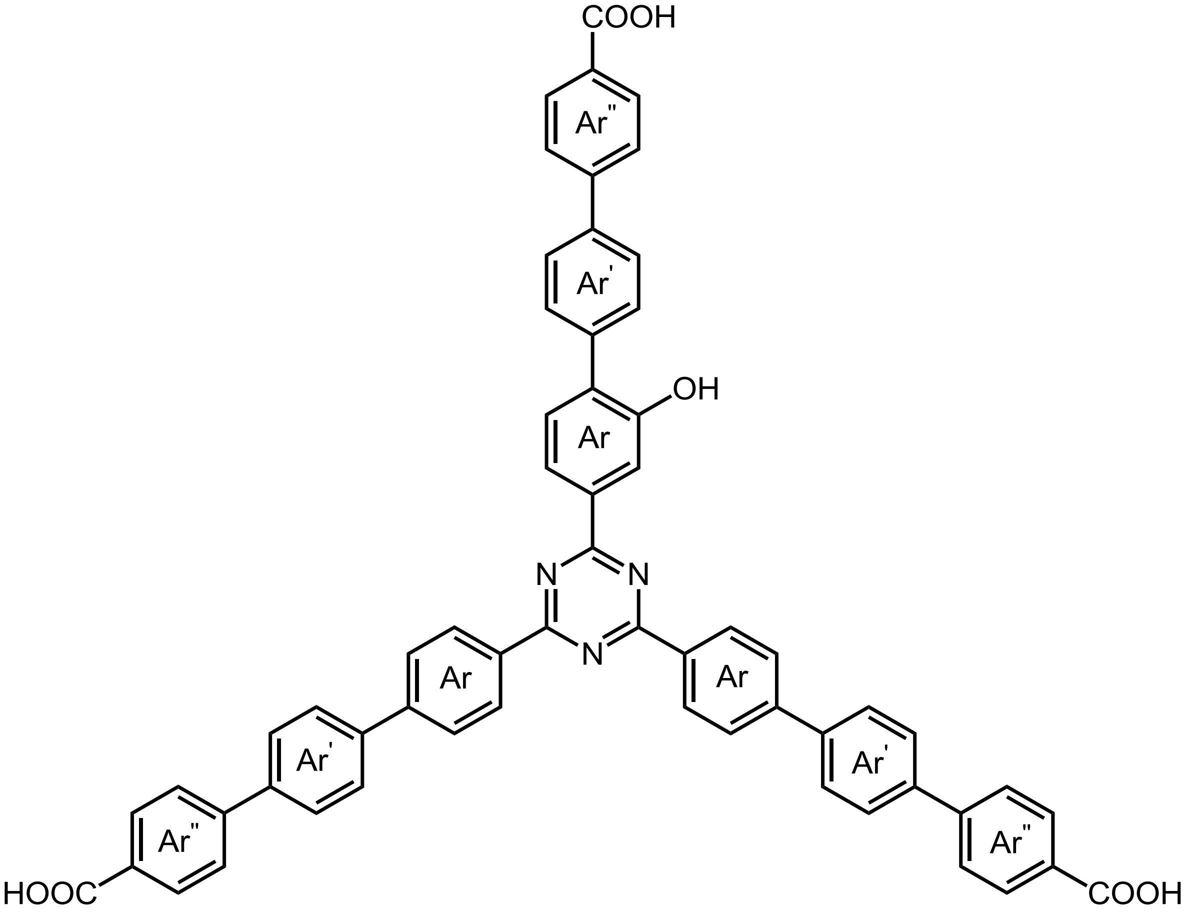

2,4-Bis[4''-(methoxycarbonyl)-(1,1':4',1''-terphenyl)-4-yl]-6-[2-methoxy-4''-(methoxycarbonyl)-(1,1':4',1''-terphenyl)-4-yl]-1,3,5-triazine (**19c**, 109 mg, 113 µmol) and pyridinium hydrochloride (1.80 g, 15.6 mmol) were mixed and stirred for 12 h at 200 °C. After cooling to room temp., sodium hydroxide (2 M) was added to the solution until pH 14 was reached. The reaction mixture was stirred for 12 h at 120 °C before it was acidified with conc. hydrochloric acid and the resulting precipitate was filtered off. Washing with water and drying gave a dark red solid. Yield: 64 mg (70.0 µmol, 62%). M. p.: > 300 °C. ^{1}H NMR (600 MHz, DMSO-d$_6$): δ = 12.99 (br.s, 3H, COO*H*), 10.09 (s, 1H, O*H*), 8.63 (d, ^{3}J = 7.7 Hz, 4H, 4,6-Ar-*H*-3,5), 8.31 (s, 1H, *2*-Ar-

H-3), 8.11 (d, ^3J = 7.6 Hz, 1H, 2-Ar-*H*-5), 8.06 - 7.99 (m, 6H, 4,6-Ar''-*H*-3,5, 2-Ar''-*H*-3,5), 7.86 (d, ^3J = 7.7 Hz, 4H, 4,6-Ar-*H*-2,6), 7.82 - 7.75 (m, 14H, 2-Ar''-*H*-2,6, 4,6-Ar'-2,3,5,6, 4,6-Ar''-2,6), 7.74 – 7.68 (m, 4H, 2-Ar'-*H*-2,3,5,6), 7.44 (d, ^3J = 7.6 Hz, 1H, 2-Ar-*H*-6) ppm. ^{13}C NMR (150 MHz, DMSO-d$_6$): δ = 170.5 (s, triazine-*C*-2), 170.4 (s, triazine-*C*-4,6), 167.2 (s, 2-Ar''-*C*OOH), 167.1 (s, 4,6-Ar''-*C*OOH), 154.8 (s, 2-Ar-*C*-2), 143.9 (s, 2-Ar''-*C*-1), 143.4 (s, 4,6-Ar-*C*-1), 143.3 (s, 4,6-Ar''-*C*-1), 138.8 (s, 4,6-Ar'-*C*-1), 138.6 (s, 4,6-Ar'-*C*-4), 137.6 (s, 2-Ar'-*C*-4), 137.5 (s, 2-Ar'-*C*-1), 135.7 (s, 2-Ar-*C*-4), 134.6 (s, 4,6-Ar-*C*-4), 131.4 (s, 2-Ar''-*C*-4), 131.2 (s, 2-Ar-*C*-1), 130.4 (d, 2-Ar-*C*-6), 130.0 (d, 2-Ar''-*C*-3,5, 4,6-Ar''-*C*-3,5), 129.8 (d, 2-Ar'-*C*-2,6), 129.6 (s, 4,6-Ar''-*C*-4), 129.1 (d, 4,6-Ar-*C*-3,5), 127.5 (d, 2-Ar''-*C*-2,6, 4,6-Ar''-*C*-2,6), 126.8 (d, 4,6-Ar-*C*-2,6), 126.6 (d, 4,6-Ar'-*C*-2,3,5,6), 126.4 (d, 2-Ar'-*C*-3,5), 120.0 (d, 2-Ar-*C*-5), 115.9 (d, 2-Ar-*C*-3) ppm. MS (MALDI, Cl-CCA): *m/z* = 914 [M + H]$^+$. IR (ATR): $\tilde{\nu}$ = 2925 (O-H), 1682 (C=O), 1605, 1572, 1504 (arom. C=C, arom. C=N), 1407 (CH-Def.), 1361 (O-H-Def.), 1251, 1180, 1100 (C-O), 808, 773 (1,4-disubst. aryl, 1,2,4-trisubst. aryl) cm^{-1}. Elemental analysis ($C_{60}H_{39}N_3O_7$) (913.28): calcd. C 78.35 H 4.30 N 4.60; ($C_{60}H_{39}N_3O_7 \cdot 2.5\ H_2O$) (958.31): calcd. C 75.14 H 4.62 N 4.38; found C 75.23 H 4.29 N 4.35.

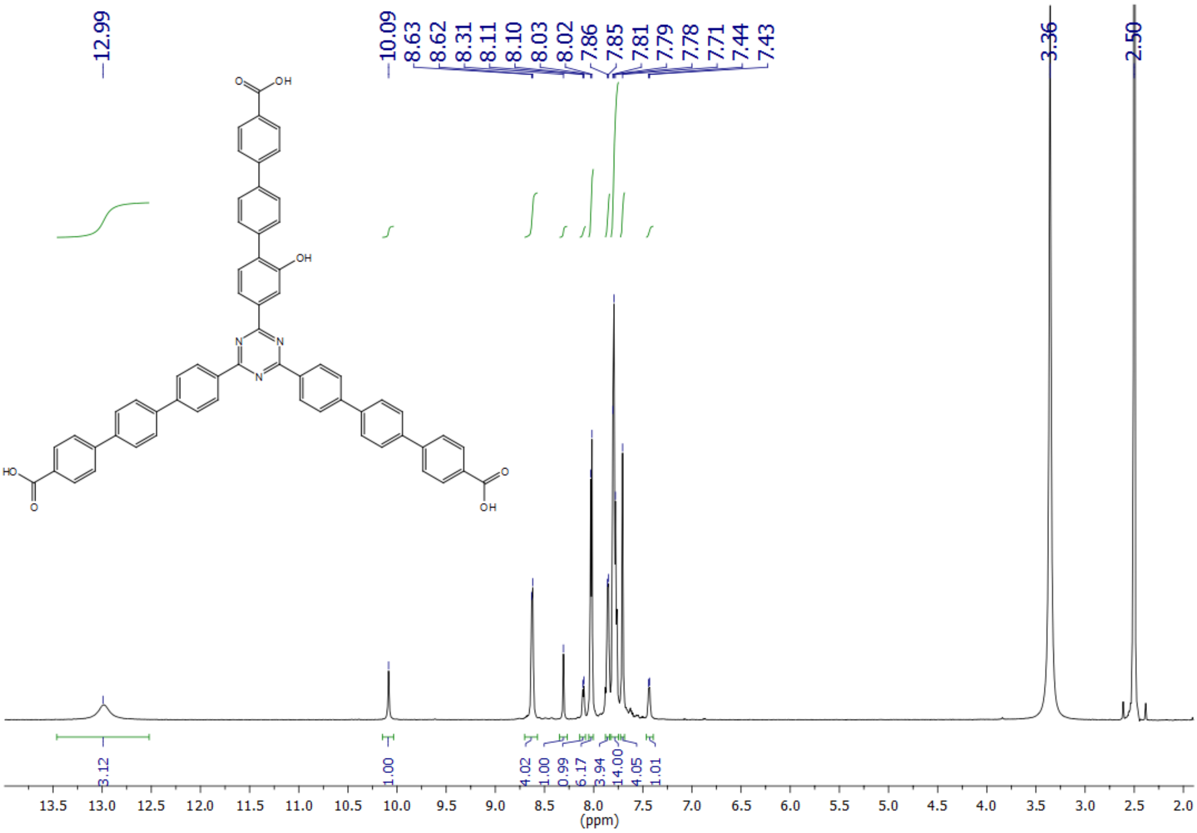
12.99
10.09
8.63
8.62
8.31
8.11
8.10
8.03
8.02
7.86
7.85
7.81
7.79
7.78
7.71
7.44
7.43
3.36
2.50
OH
HO
O
N
3.12
1.00
4.02
1.00
0.99
6.17
3.94
14.00
4.05
1.01
(ppm)

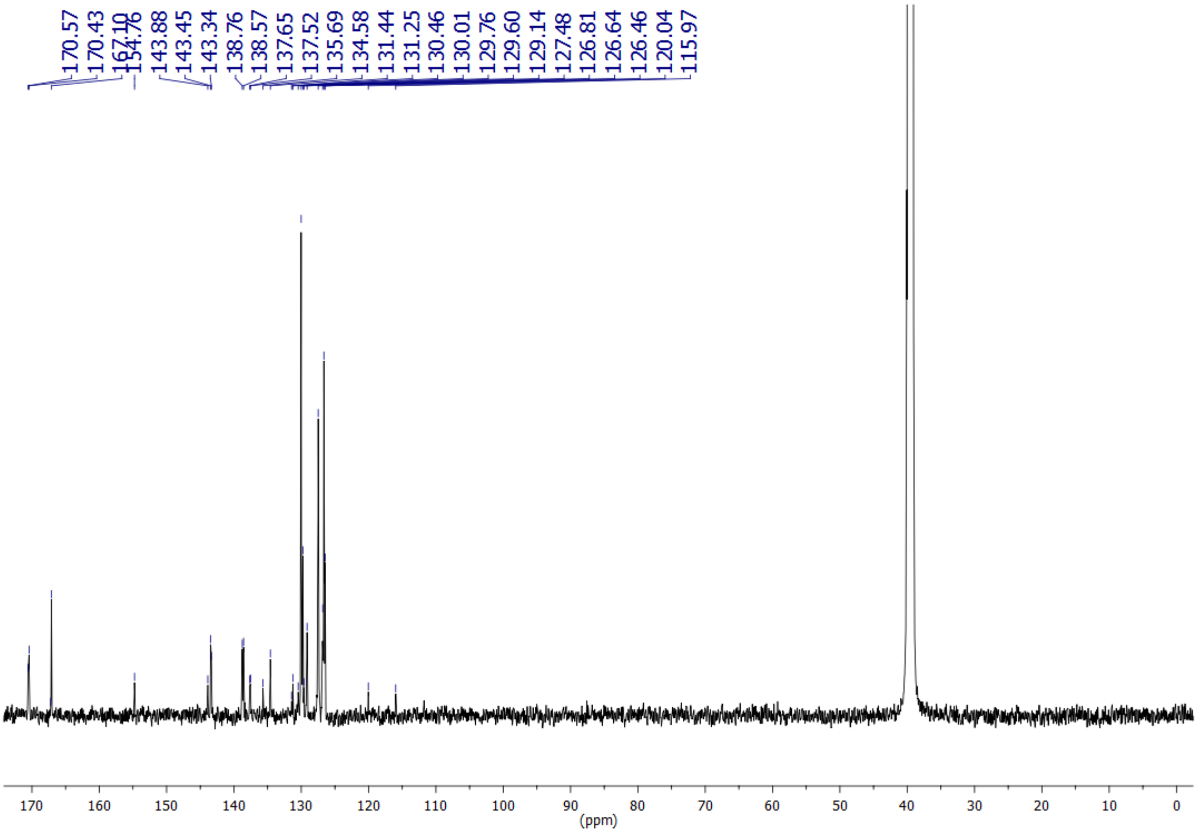
170.57
170.43
167.10
154.76
143.88
143.45
143.34
138.76
138.57
137.65
137.52
135.69
134.58
131.44
131.25
130.46
130.01
129.76
129.60
129.14
127.48
126.81
126.64
126.46
120.04
115.97
(ppm)

Calculations

BTB (benzene-1,3,5-tribenzoic acid)

The BTB molecule was optimized using Gaussian® 09 [6] with B3LYP//6-31G*.

Energy: -935705.0864025

C	-1.32248	-0.43370	-0.01257
C	-1.05177	0.94191	-0.01132
C	0.28557	1.36279	-0.01013
C	1.34177	0.44074	-0.00996
C	1.03767	-0.92784	-0.01051
C	-0.28902	-1.38108	-0.01175
H	0.50783	2.42534	-0.00981
H	1.84625	-1.65211	-0.01107
C	2.75334	0.90349	-0.01055
C	3.14559	2.02476	0.74141
C	4.46639	2.46146	0.74298
C	5.43222	1.78183	-0.01149
C	5.05345	0.66267	-0.76452
C	3.73277	0.23106	-0.76360
H	3.44801	-0.62099	-1.37383
H	2.41210	2.54322	1.35190
H	4.75505	3.32301	1.33479
H	5.80830	0.14997	-1.35162
C	-0.59388	-2.83506	-0.01234
C	-1.66500	-3.34777	-0.76629

C	0.18032	-3.73462	0.74129
C	-0.10190	-5.09678	0.74396
C	-1.17237	-5.59414	-0.01145
C	-1.95124	-4.70739	-0.76651
H	-2.25991	-2.67566	-1.37769
H	-2.77180	-5.10540	-1.35441
H	0.99544	-3.35799	1.35220
H	0.49916	-5.77694	1.33719
C	-2.15862	1.93276	-0.01160
C	-2.06575	3.11893	-0.76197
C	-3.10062	4.04607	-0.76230
C	-4.26001	3.81213	-0.01119
C	-4.36575	2.63434	0.74086
C	-3.32654	1.70960	0.73856
H	-3.40889	0.81385	1.34711
H	-5.25663	2.45183	1.33147
H	-1.18473	3.30043	-1.37051
H	-3.03402	4.95729	-1.34761
C	-5.33614	4.83500	-0.04728
O	-5.29134	5.87119	-0.68044
O	-6.41155	4.50300	0.71394
C	6.85643	2.20110	-0.04824
O	7.73018	1.64472	-0.68353
H	-7.04181	5.23992	0.61211
H	-2.35372	-0.77267	-0.01424

C	-1.52117	-7.03727	-0.04713
O	-2.43930	-7.51650	-0.68272
O	-0.69892	-7.80259	0.71735
H	-1.02248	-8.71666	0.61509
O	7.10850	3.29649	0.71515
H	8.06188	3.47320	0.61263

1\1\GINC-ACID10\Freq\RB3LYP\6-31G(d)\C27H18O6\WINKLER\13-Sep-2016\0\\#N Geom=AllCheck Guess=TCheck SCRF=Check GenChk RB3LYP/6-31G(d) Freq\\Title\\0,1\C,-3.2140041378,-0.6172921952,-0.2479328446\C,-3.0543624924,0.7734489249,-0.1708180694\C,-1.7550541616,1.2988606464,-0.1335922137\C,-0.6284156648,0.4652125211,-0.1716207432\C,-0.8219107006,-0.92123097,-0.2480040197\C,-2.1080012288,-1.4779947373,-0.286887202\H,-1.618625132,2.3741791368,-0.0745415932\H,0.0420820541,-1.5776980837,-0.2787832946\C,0.7415290321,1.0383055855,-0.1331889001\C,1.0382288058,2.1451918463,0.6812606238\C,2.3197758834,2.6850043614,0.7195437517\C,3.3414071623,2.1260442243,-0.0600624112\C,3.0579822654,1.0223926949,-0.8753326411\C,1.7761310802,0.4875220023,-0.9108495929\H,1.5641674813,-0.350452225,-1.5683688716\H,0.261950865,2.5699756055,1.3109121021\H,2.5349794236,3.5337380213,1.3592093914\H,3.8549485896,0.6037807432,-1.4810929319\C,-2.2954050591,-2.9494946625,-0.3678520482\C,-3.3175154162,-3.5046107187,-1.1588950561\C,-1.4561312984,-3.8237483871,0.3447853757\C,-1.6283542537,-5.2022262097,0.272190587\C,-2.6510332033,-5.7419376183,-0.5194913704\C,-3.4939318394,-4.880725489,-1.2342859378\H,-3.9607105256,-2.8501307896,-1.7397729482\H,-4.2764586288,-5.3106524383,-1.8508622076\H,-0.6774425979,-3.4167801551,0.9831501188\H,-0.9782751619,-5.86325938,0.8345556889\C,-4.2369990213,1.6715507747,-0.1297663641\C,-4.2349809417,2.8999795868,-0.8150561397\C,-5.3407663434,3.7404052625,-0.7767045447\C,-6.4821383638,3.374783892,-0.0509788183\C,-6.4976597719,2.1535803135,0.6363011794\C,-5.3877232144,1.3160315535,0.5954941582\H,-5.4016828947,0.3851427949,1.1547113495\H,-7.3745581305,1.8691770374,1.2072406985\H,-3.3677284592,3.1835595732,-1.4040563655\H,-5.3438922615,4.6841947743,-1.3120576274\C,-7.6365058307,4.3091253525,-0.0436701698\O,-7.6710828222,5.3781423121,-0.6203764124\O,-8.6863737796,3.8520534953,0.6878012449\C,4.7276482998,2.658812237,-0.0599146195\O,5.6469191123,2.2087884349,-0.7149119885\H,-9.3730208055,4.540823784,0.6188358729\H,-4.2147450489,-1.0367511931,-0.2781589991\C,-2.8828965289,-7.2042532382,-0.6353625543\O,-3.7559033711,-7.7202187838,-1.3048783025\O,-2.0065488432,-7.9416405149,0.09573392\H,-2.2552336336,-8.8717533116,-0.0582539482\O,4.8866296102,3.7281545247,0.763135783\H,5.8233819036,3.985

5090837,0.6798999055\\Version=EM64L-G09RevD.01\State=1-A\HF=-
1491.1409829\RMSD=7.732e-09\RMSF=2.978e-
05\ZeroPoint=0.3888715\Thermal=0.4158876\Dipole=-0.0058257,-
0.0517846,0.9647202\DipoleDeriv=-0.2123376,0.014317,-0.001957,0.0092191,-
0.2295066,-0.0013425,0.0551711,-0.135959,-0.1211125,0.2184088,-0.2090566,-
0.0025019,-0.2107199,0.1003499,0.0139116,0.0247487,0.0492045,0.0185459,-
0.2408764,0.0041136,0.0048781,0.0110684,-0.2077143,-0.0050715,-
0.1397874,0.0124897,-
0.1145737,0.3125572,0.1540484,0.0154044,0.1529198,0.012343,-
0.0112887,0.0318354,-0.0528362,0.0117322,-0.2195733,-0.0168652,-0.0043031,-
0.0220785,-0.2348681,-0.0110097,0.0834282,0.1063643,-0.1083642,-
0.0484593,0.0553458,-0.0105329,0.0573706,0.369419,0.0207137,-
0.0547606,0.0262484,0.0160536,0.1404525,-0.0275277,-0.0081961,-0.0291558,-
0.0749516,-0.0086685,0.0183357,-
0.0119319,0.0948145,0.0030941,0.1072718,0.0092823,0.1081648,0.0627145,0.004
5718,-0.0064726,-
0.0167554,0.0946771,0.5207996,0.2133427,0.0058673,0.2257111,0.1005585,0.022
1145,0.0236565,0.0066319,0.0489266,-0.2648834,-0.2069151,-0.1401039,-
0.2079987,-0.1842338,0.0226003,-0.172153,-0.0075917,-
0.0568098,0.2206469,0.1840834,0.0615894,0.0089431,0.0330961,0.0887102,-
0.0524557,0.0537686,-0.0222927,-0.8488674,-0.3412069,-0.0115159,-0.3612062,-
0.1917615,-0.0486672,-0.0308539,-0.0556002,-0.0273345,0.1924395,-0.0117066,-
0.0964248,0.1784427,0.0279584,0.023549,0.0268019,0.077711,-0.0226329,-
0.411529,-0.0280183,0.0722425,-0.0525441,-
0.0215862,0.1146967,0.0959782,0.1092378,-0.044181,0.0862994,-0.0348436,-
0.0234273,-0.0399156,-0.0073764,-0.0855636,-0.0541638,-0.0875329,0.0391127,-
0.0077038,0.0429911,0.0762314,0.050031,0.0779452,-0.0411478,0.1001557,-
0.021028,0.0450992,0.0811632,-0.0181513,-0.007278,-0.0329877,0.0200148,-
0.0651861,-0.0193871,-
0.0704642,0.0569307,0.0347347,0.031339,0.0514369,0.0257135,0.0832814,-
0.0288676,0.0469428,-
0.0312242,0.0675988,0.0156072,0.0630273,0.0230516,0.0779078,0.6056134,0.029
0167,0.0017334,0.0222472,0.0489093,-0.0801035,-0.1464832,0.0522403,-
0.1687568,-0.3256195,-0.1378497,0.0337401,-0.1566686,-0.0713866,-
0.0184173,0.0673123,0.0942099,0.0653587,-
0.4441757,0.0747285,0.083929,0.1174226,-
0.043881,0.0008047,0.1172337,0.0553709,-0.0639965,0.2625146,-
0.0709362,0.0668257,0.0386983,-0.031391,-0.0546291,-0.0939146,-0.0435809,-
0.1145114,-0.9898163,-0.0439721,-0.0417494,-0.0243493,-0.0235802,0.0013382,-
0.0732935,0.0617228,0.1221298,0.220908,0.088384,0.0637382,-0.0384077,-
0.0245786,0.0443878,0.0704189,-0.0567177,0.0629589,0.0177232,0.0588199,-
0.0437813,0.0862499,0.0557409,0.0428391,-0.0289366,-0.0524978,-
0.0348023,0.0760029,-0.0283398,-0.0527591,-0.0234465,0.066823,0.0111214,-
0.0602528,-0.0779179,-0.0525642,0.0638727,-0.042125,-0.071712,-
0.0724898,0.0403789,0.0536176,0.0527642,-
0.0483786,0.037454,0.0363407,0.0356401,-
0.0481359,0.0482647,0.068047,0.3976683,-0.2972173,-0.0252846,-
0.2837267,0.2252062,-0.0082511,-0.0201103,0.0142724,0.0472002,-
0.1380544,0.20979,-0.1249446,0.1875032,-0.2815145,-0.0022852,-
0.1339284,0.0224263,-0.0574174,0.1438397,-0.208894,0.0213614,-
0.0068712,0.087853,-0.087224,-0.0718691,-0.0189723,-0.033971,-

0.6660937,0.4667553,0.0479223,0.4448546,-0.3824067,0.0119823,0.0620654,-
0.0000441,-0.0199543,0.1705591,-0.0299928,-0.0992675,-
0.2157822,0.0951978,0.0061424,-0.0265147,-0.0648437,-0.0340599,-
0.3919998,0.1440428,0.0430521,0.1386764,-0.0443489,-0.1239989,0.0852812,-
0.1367715,-0.0702201,0.098534,0.0048956,0.0008084,0.014911,-
0.0404115,0.0809258,-0.0279138,0.0911017,0.0572131,0.0062032,-
0.0119295,0.056301,-
0.0278853,0.0844909,0.0266726,0.0658427,0.0197957,0.0675901,-0.0268479,-
0.0256195,0.0802534,-0.0335469,0.0937222,0.0246005,0.0969185,-
0.0006481,0.0512337,0.0955284,0.0064818,0.0012588,0.0002803,0.0135694,0.054
8948,0.0050549,0.0526198,0.0765705,2.1696996,-0.8247187,-0.4019514,-
0.6569581,1.5222193,-0.3873841,-0.4862778,-0.3041481,0.7044318,-
0.8625805,0.3914195,0.0159295,0.4027662,-
1.1623779,0.2730799,0.0065431,0.2720015,-0.5183417,-
1.3347732,0.1151707,0.3860769,-0.1166833,-0.4092016,0.0796632,0.5046374,-
0.0276813,-0.5927966,2.3185377,0.6027767,-
0.1439883,0.7578064,1.266377,0.5619784,-0.0158459,0.5995831,0.8111146,-
1.4120869,-0.1017486,0.2551191,-0.0909936,-0.5678875,-0.1508338,0.2598293,-
0.1416451,-0.5631662,0.39193,-0.0202366,-
0.0142696,0.0735642,0.2068111,0.038281,-0.0647778,0.0795732,0.3256328,-
0.0457093,-0.0795596,-0.0024633,-0.0790086,0.1103127,-0.0061305,-
0.0129312,0.0184322,0.0958889,0.9803002,-
0.0194799,0.5526848,0.1424017,2.6691295,-0.0009934,0.5349108,-
0.125348,0.7463476,-0.714297,-0.3055447,-0.2796056,-0.2951413,-1.2556726,-
0.1946946,-0.273154,-0.2027816,-0.5732354,-0.6125956,0.5079242,-
0.2257643,0.2841264,-1.1588225,0.2703552,-0.2090618,0.4398565,-
0.5651756,0.2799936,-0.1320525,-0.0386658,-0.041622,0.3328623,-0.0333706,-
0.0423627,-0.1032011,0.3114137,-0.6565443,-0.2895175,-0.1482042,-0.5047695,-
1.0320065,-0.3938382,-0.3196413,-0.4515053,-
0.6483051,0.2358556,0.0168188,0.0454478,0.1039419,0.3678513,-
0.0069924,0.114457,0.019675,0.3207243\Polar=428.4983183,-
0.0434111,427.635721,1.6570072,14.5598098,158.4307952\PG=C01
[X(C27H18O6)]\NImag=0\\0.72394149,0.02385239,0.67579398,0.00410931,0.03341
200,0.13308120,-0.12773320,0.00147151,0.00246242,0.65289289,-0.05675666,-
0.31092263,-0.01799160,0.01592761,0.66097582,-0.00032025,-0.01383385,-
0.06355740,-0.00778041,0.01161376,0.16244813,0.01661316,-0.07830657,-
0.00382260,-0.28446348,-0.04151616,-0.00483963,0.66827295,-0.03519454,-
0.01485495,-0.00150737,-0.09974192,-0.15455734,-
0.00250242,0.00759082,0.73076273,-0.00201079,-0.00140015,0.00703095,-
0.01150102,-0.00350782,-0.06270747,0.00768148,0.03200012,0.13273346,-
0.00804873,0.02340100,0.00100850,-0.05574646,-0.00805475,-0.00046181,-
0.24192186,0.12282735,0.00061180,0.64570221,0.02334931,-0.05388910,-
0.00193421,0.03736606,0.05774239,0.00064130,0.06431324,-0.19661444,-
0.00732588,-0.01098070,0.66416361,0.00160917,-0.00341127,-
0.00483926,0.00262627,0.00508708,0.00518137,0.00052372,-0.00917648,-
0.06312203,-0.00531713,0.04517928,0.16630628,-
0.05615974,0.03629075,0.00134870,-0.02250428,-0.03163095,-
0.00125902,0.04216031,0.02050007,0.00109210,-
0.12593134,0.00829859,0.00328229,0.71001655,-
0.00678615,0.05777424,0.00242197,-0.03157493,-0.03950681,-
0.00133984,0.06355744,-0.04046393,-0.00244486,-0.05001384,-0.31313206,-

0.01446141,-0.03190412,0.69056255,-0.00091357,0.00336525,0.00728640,-
0.00222336,-0.00261172,-0.00476267,0.00404474,-
0.00249358,0.00707044,0.00066636,-0.01033875,-0.06318632,-
0.00051566,0.02714706,0.13220195,-
0.24882822,0.06235533,0.00289024,0.04219516,0.06469965,0.00127408,-
0.06279338,0.00817979,0.00069293,0.01676408,-0.07909749,-0.00655401,-
0.28960390,-0.03686946,-0.00140871,0.67216070,0.12089020,-0.18971565,-
0.01095597,0.01903028,-0.04018811,-
0.00261214,0.00826082,0.00067062,0.00026691,-0.03388323,-0.01454533,-
0.00036876,-0.09506285,-0.14976426,-0.00084785,-
0.00511848,0.64009532,0.00311302,-0.00895035,-0.06334203,0.00323615,-
0.00048155,0.00505660,-0.00089559,0.00046401,-0.00468345,-0.00026798,-
0.00358523,0.00486963,-0.00808608,-0.00202104,-
0.06244824,0.02272772,0.02327627,0.16428356,-0.00543636,-0.00106921,-
0.00028916,0.00023684,-0.02945474,-0.00167133,-0.06329891,-0.03477447,-
0.00097203,0.00684330,0.02520503,0.00132245,-0.00424978,0.00247735,-
0.00009714,-0.00098243,0.00019637,-0.00004154,0.06659229,-
0.00209438,0.00111537,-0.00021465,-0.00047242,-0.00894174,-0.00067073,-
0.03480177,-0.33442063,-0.01651835,-0.00371849,-0.01556892,-
0.00095488,0.00353659,-0.00002190,-
0.00037319,0.00016351,0.00051309,0.00008129,0.03720334,0.35744072,-
0.00063934,0.00018350,0.00643600,0.00095507,-0.00179901,0.00286490,-
0.00199632,-0.01640069,-0.03651199,0.00103829,-0.00013573,0.00293832,-
0.00042136,-0.00068818,0.00638411,0.00053165,0.00000750,-
0.00083336,0.00366590,0.01782913,0.02673534,-0.00369089,-0.00383507,-
0.00022852,-0.00001897,-0.00073229,-0.00000238,0.00086578,-
0.00152601,0.00003202,0.00620227,-0.00300373,-0.00012593,-
0.23701918,0.13516271,0.00558914,-
0.01949440,0.01878009,0.00094451,0.00030004,-
0.00026241,0.00006774,0.25307347,-0.00278080,-0.00063353,-0.00023624,-
0.00072628,-0.00042633,0.00005931,-0.00259327,-0.00516278,-
0.00055842,0.02593787,-0.01482289,-0.00071103,0.13520356,-0.16170300,-
0.00692083,-0.01020124,0.01065644,0.00037977,0.00138511,-0.00079711,-
0.00009226,-0.14494306,0.17211661,-0.00024692,0.00022460,0.00644357,-
0.00034584,-0.00038757,-0.00085564,0.00045067,-
0.00048364,0.00640793,0.00010579,-0.00135876,0.00283903,0.00624422,-
0.00610850,-0.03601213,-0.00075732,-0.00088276,0.00288251,0.00004467,-
0.00014684,0.00029767,-0.00747148,0.00595857,0.02616222,-
0.00090980,0.00003173,-0.00004753,0.00145301,0.00489126,0.00044364,-
0.03753526,-0.00483324,0.00285986,-0.20058749,-0.04290897,0.00167099,-
0.00633030,-0.01775510,-0.00463785,0.00443660,0.00181242,-
0.00040799,0.00138285,-0.00168659,-
0.00105237,0.00011074,0.00177628,0.00129954,0.61733733,0.00002541,-
0.00096249,0.00011025,0.00468855,-
0.00212596,0.00009049,0.01261790,0.01570599,0.00252306,-0.04285574,-
0.11500181,-0.01400158,-0.03520474,-0.01519298,-0.00236299,0.00197834,-
0.00508281,-0.00039071,0.00062430,0.00003311,-0.00054047,-
0.00052019,0.00107929,0.00041104,0.05736509,0.48799311,0.00019786,-
0.00046548,-0.00098239,0.00090763,-
0.00032948,0.00648762,0.00070599,0.00139436,0.00349254,0.00159499,-
0.01379102,-0.07056920,-0.00268064,-0.00077678,0.00316065,-0.00046604,-

0.00103324,0.00640523,0.00004402,0.00017130,-0.00347910,-
0.00000064,0.00026187,-0.00341093,-0.08690790,0.22745419,0.36521037,-
0.00009997,-0.00086082,-0.00000466,0.00063439,-0.00015349,0.00095643,-
0.00120438,-0.00266067,-0.00151214,-0.00871648,-0.02921915,-0.01850790,-
0.00413473,0.00230006,0.00017788,-0.00093368,-0.00130595,0.00105512,-
0.00054417,-0.00021984,-0.00124166,0.00027319,-0.00029817,-0.00064565,-
0.12790370,-
0.01066153,0.00868753,0.69161317,0.00057556,0.00001401,0.00001385,-
0.00043300,-0.00075004,0.00021348,0.00368086,-0.00176307,-0.00201564,-
0.01753905,-0.01202624,-0.00726051,0.00086783,0.00386252,-0.00039770,-
0.00140980,-
0.00034285,0.00052860,0.00019490,0.00006586,0.00049095,0.00007664,-
0.00025949,-0.00057238,-0.05351179,-0.21547862,-
0.10649092,0.06708312,0.46091048,0.00020715,-
0.00036526,0.00013104,0.00065154,-0.00027536,-
0.00013960,0.00033410,0.00013428,-0.00083164,-0.00573697,-
0.00234121,0.00385322,0.00219255,0.00230014,0.00109212,-
0.00052479,0.00014231,-0.00014946,0.00006228,-
0.00083253,0.00036890,0.00010767,-0.00028968,-0.00014013,-0.02860083,-
0.12242894,-0.15487257,-0.12869783,0.25660848,0.38838777,-
0.00012535,0.00036288,-0.00007168,-0.00010431,0.00019035,-
0.00062817,0.00017225,0.00092092,0.00097973,0.00503233,0.00054405,0.001542
08,-0.00102628,-0.00181487,-0.00048339,0.00085550,0.00005886,-0.00048792,-
0.00037225,0.00004705,0.00061358,-
0.00003983,0.00033351,0.00045392,0.00745117,-0.06317423,-0.05163048,-
0.31319909,-0.06349844,0.02735556,0.65235019,0.00005905,-0.00018491,-
0.00007141,-0.00002252,0.00042877,0.00005709,0.00000565,-
0.00041807,0.00043599,0.00037328,-0.00062975,-0.00562328,-0.00032026,-
0.00003643,0.00000854,-0.00015607,-0.00004796,0.00049940,-
0.00032668,0.00037017,-0.00007570,0.00002020,0.00010427,-0.00022319,-
0.02685384,-0.01422998,-0.00758665,-0.11425107,-0.13486626,-
0.02735846,0.09981728,0.51499295,0.00004872,0.00039241,0.00004192,-
0.00043730,-
0.00029190,0.00007102,0.00092122,0.00017865,0.00019359,0.00157644,-
0.00579736,0.00124401,-0.00157411,-0.00030293,-0.00009052,0.00043470,-
0.00054987,0.00015739,0.00011971,0.00012962,-0.00034747,0.00003176,-
0.00001795,-0.00011989,-0.02382993,0.00462021,0.01599004,-0.01369793,-
0.04360335,-0.09121443,-0.08050403,0.28327015,0.39632339,-0.00044261,-
0.00028779,0.00001057,0.00004967,-0.00045338,0.00045874,-0.00000774,-
0.00112458,-0.00094060,-0.00179259,-0.00032772,-0.00010661,-
0.00040146,0.00105348,0.00076589,-0.00046273,0.00030719,-
0.00022102,0.00020991,-0.00007364,-0.00016430,0.00003170,0.00003012,-
0.00007070,-0.00202263,0.01600017,0.01145717,-0.05089455,-
0.01193962,0.01018156,-0.21841038,0.07153560,0.10758090,0.62330079,-
0.00029145,0.00010508,-0.00005627,-0.00019432,-0.00032017,-
0.00014253,0.00066751,-0.00033952,-0.00016422,-0.00032448,-
0.00112603,0.00016783,-
0.00080419,0.00007361,0.00053229,0.00006716,0.00020009,-0.00044323,-
0.00013665,0.00006546,0.00024790,0.00009284,0.00022654,0.00030720,0.015885
52,-0.03185925,-0.02512180,0.02408311,0.03823062,0.01753871,0.03012268,-
0.13721821,-0.07082035,0.05733559,0.48426175,-0.00009071,0.00012600,-

0.00004892,-0.00016809,-0.00002092,-0.00025435,0.00054686,-
0.00006714,0.00025665,0.00010387,-0.00026453,-0.00072141,-0.00042759,-
0.00038281,0.00028284,0.00012452,0.00015055,-0.00028527,-
0.00019376,0.00017522,0.00025192,0.00000406,0.00025604,0.00027120,0.011732
81,-0.02610106,-0.03129939,0.03869279,0.02953405,0.01950693,0.07260611,-
0.08399686,-0.15452147,-0.10493269,0.26306897,0.38954724,0.00006124,-
0.00013392,0.00009845,0.00034281,0.00038109,-0.00000675,-
0.00168011,0.00018104,0.00025354,0.00232378,0.00299779,0.00309284,0.000431
26,-0.00012741,-0.00099003,0.00020563,-0.00037617,0.00040156,0.00021824,-
0.00022150,-0.00014162,-0.00011110,-0.00054029,-0.00037839,-
0.05106996,0.02622907,0.03867608,-0.02898957,-0.02599767,-
0.01446987,0.04770426,0.01638186,0.00092734,-0.12426097,-
0.01292569,0.00929388,0.70551850,0.00018956,-
0.00036718,0.00000004,0.00052421,0.00045253,0.00072585,-
0.00129698,0.00052698,0.00029706,0.00315759,0.00199173,-
0.00487677,0.00080374,-0.00058033,-0.00037688,-
0.00018663,0.00006623,0.00048947,0.00011265,-0.00013939,-0.00047069,-
0.00016052,0.00014935,-0.00049450,-0.00957124,0.04093842,0.03161517,-
0.02840156,-0.03197704,-0.01190167,0.04907046,-0.01439038,-0.03454206,-
0.05463716,-0.22485232,-0.11342744,0.06627996,0.47566068,-
0.00029592,0.00022396,0.00003761,0.00011024,-
0.00066321,0.00015788,0.00121786,0.00094677,0.00001520,0.00318454,-
0.00503731,0.00133413,-0.00073054,-
0.00055983,0.00008135,0.00050824,0.00008310,0.00013443,0.00001162,-
0.00004939,-0.00013814,-0.00016867,-0.00000599,-
0.00038965,0.01021965,0.02223954,0.01970093,-0.01653962,-0.01298546,-
0.01158339,0.02825485,-0.02403371,-0.02023850,-0.02574825,-0.12718493,-
0.16077116,-0.12740267,0.25808067,0.39288945,-0.00018570,0.00068510,-
0.00000558,-0.00194660,-0.00037119,-0.00120746,0.00168218,-
0.00467679,0.00011368,-0.03485507,0.00656392,0.01640242,-
0.00109709,0.00341449,0.00254373,-0.00030000,0.00059228,-0.00082632,-
0.00015801,0.00044583,0.00086607,-0.00032790,-0.00013371,0.00049967,-
0.21177039,0.02329625,0.06691930,0.04850587,0.04754696,0.02689064,-
0.05963374,0.00147611,0.01832378,0.00335104,-0.06107966,-0.04970676,-
0.31668319,-0.06604376,0.02786786,0.65175631,-0.00077978,0.00010134,-
0.00002399,-0.00024607,0.00071439,-0.00037312,-0.00317095,-0.00177471,-
0.00061750,-0.00508337,0.01279291,0.00801671,-0.00284588,-0.00196078,-
0.00049667,0.00084843,0.00027092,-
0.00047759,0.00007290,0.00012720,0.00006366,-0.00058546,-
0.00021415,0.00118854,0.06611287,-0.13604424,-0.07731918,0.01682061,-
0.01246262,-0.02336701,-0.00046534,-0.00331583,0.00275265,-0.02564280,-
0.01249872,-0.00811940,-0.11599846,-0.13671072,-
0.02768577,0.10705023,0.50069445,0.00007470,-
0.00036756,0.00012803,0.00016821,0.00046132,-0.00019856,-0.00320930,-
0.00023734,0.00091338,0.00373345,0.00295180,0.00516980,-
0.00041028,0.00000800,-0.00072863,-0.00024511,-0.00062024,-
0.00022032,0.00013121,-0.00025345,-0.00008627,0.00051192,-
0.00061196,0.00043422,0.10527055,-0.06404528,-0.14906562,0.00151240,-
0.03311770,-0.01980121,0.01643913,0.00233163,-0.00956607,-
0.02086710,0.00189988,0.01610562,-0.01288723,-0.04288651,-0.09172445,-
0.07627456,0.27900647,0.39111585,0.00017898,-0.00029339,-

0.00005284,0.00021030,0.00022459,0.00006491,-0.00033640,0.00013084,-
0.00001921,0.00124575,0.00111985,-0.00034143,-0.00017933,-
0.00011043,0.00011126,-0.00048462,-0.00013254,-0.00017843,0.00004025,-
0.00005163,0.00002263,-0.00061490,-
0.00051481,0.00089194,0.00784782,0.01824328,0.01377393,-
0.00324335,0.00054948,0.00356102,-0.00108428,0.00018029,0.00036620,-
0.00454117,-0.00229685,0.00202051,-0.00178774,-0.02400651,-0.01905949,-
0.06871567,-0.04505705,-0.02955957,0.07018516,-
0.00017245,0.00032228,0.00000518,-0.00025755,-0.00038866,-
0.00009599,0.00098220,-0.00001947,0.00004622,-0.00130042,-
0.00175335,0.00176461,-0.00080647,-
0.00003698,0.00077601,0.00040830,0.00013366,0.00011164,-
0.00003622,0.00002822,0.00001475,-0.00093893,-0.00005525,0.00033927,-
0.00261039,-0.00670460,-0.00716767,0.00141673,0.00247367,-
0.00326243,0.00038978,0.00035768,0.00032125,-0.00339273,0.00368350,-
0.00229691,-0.00114526,-0.00639465,-0.00701520,-0.04434580,-0.21612379,-
0.14209126,0.05111621,0.22657648,-0.00001597,-0.00009263,-0.00006662,-
0.00003394,0.00016245,0.00009849,-0.00025902,-0.00005711,0.00016990,-
0.00106679,0.00175709,-0.00143887,0.00083670,-0.00073496,0.00013100,-
0.00026684,0.00048407,0.00014473,-0.00008042,0.00006337,-
0.00005326,0.00040340,0.00101960,-0.00044212,-0.00561889,-0.01382224,-
0.00707271,0.00416634,-0.00283039,0.00219197,0.00022267,0.00041603,-
0.00008157,0.00135943,-
0.00271121,0.00477022,0.00095302,0.00076275,0.00294449,-0.02870561,-
0.14287427,-0.15042713,0.02940310,0.15661001,0.15174158,0.00001297,-
0.00001299,0.00003609,-0.00002590,-0.00005906,0.00003729,-0.00053736,-
0.00036093,-0.00069427,-0.00032482,0.00026175,-
0.00094720,0.00037261,0.00026898,-0.00000730,-
0.00010739,0.00005283,0.00001481,-0.00110376,-0.00037083,-0.00084212,-
0.00000971,0.00000883,-0.00002461,0.00775262,-0.00262953,-0.00391793,-
0.19995138,0.07463298,0.12106176,-0.01744862,0.01322756,0.01763381,-
0.00328680,-0.00459215,-0.00030397,0.00000047,-0.00058679,-
0.00049736,0.00065959,-0.00215302,-0.00028160,0.00043018,-0.00024845,-
0.00021953,0.21353686,-0.00013841,0.00048496,0.00004645,-0.00058287,-
0.00033113,0.00016673,0.00023987,0.00037444,0.00047602,0.00259984,-
0.00029475,0.00159035,-0.00058755,-0.00074411,-0.00000348,0.00054751,-
0.00006961,-0.00008930,0.00012219,0.00038964,-0.00027967,-
0.00000150,0.00007552,-0.00000171,0.01832284,-0.00674199,-
0.01398283,0.07400125,-0.09350675,-0.07435158,-
0.00973594,0.00822515,0.00831400,-0.00346420,0.00244591,-0.00324602,-
0.00080178,-0.00069149,-0.00007126,-0.00301758,-0.00123401,-
0.00504234,0.00102527,-0.00027647,-0.00090812,-
0.07918007,0.09280141,0.00007740,-0.00004444,-0.00006139,-
0.00017714,0.00051944,0.00015507,-0.00008978,-0.00111324,0.00009283,-
0.00042924,0.00204235,-0.00147129,0.00022143,0.00014003,0.00018486,-
0.00009156,0.00013932,0.00009666,-0.00101553,0.00051587,-
0.00045612,0.00001379,0.00010511,-0.00005334,0.01488795,-0.00583869,-
0.00701874,0.12109025,-0.07549953,-0.14175547,-
0.00248527,0.00057000,0.00379277,0.00052575,-0.00311363,0.00463780,-
0.00049087,0.00010415,-0.00014977,-0.00103358,-
0.00513498,0.00189599,0.00085186,-0.00049563,-0.00015578,-

0.13078017,0.08431039,0.14257454,-0.00002637,-0.00000804,-0.00000211,-
0.00001618,0.00001328,0.00002207,-0.00000717,-
0.00005758,0.00004754,0.00039629,-0.00097028,-0.00080734,-0.00039883,-
0.00002764,-0.00003828,0.00001832,-0.00009656,0.00007183,-
0.00002156,0.00001991,-0.00004512,0.00000975,-0.00000504,-0.00002692,-
0.00451145,-0.00237091,0.00185899,-0.00139030,-0.02342026,-0.01820945,-
0.06930535,-0.04767371,-0.02967390,0.00925962,0.01881521,0.01360618,-
0.00362941,0.00073082,0.00355130,-
0.00089418,0.00021235,0.00030306,0.00003532,0.00013019,-
0.00024610,0.00074847,0.00100514,-0.00071857,0.06993889,-0.00002535,-
0.00001521,-0.00000330,0.00000811,0.00003012,-0.00001682,0.00000509,-
0.00006918,0.00005421,0.00030317,-0.00032495,-0.00029277,-
0.00016116,0.00003912,0.00005619,0.00005009,-0.00004103,-
0.00000431,0.00003746,0.00003364,0.00000277,0.00002752,-
0.00000851,0.00000143,-0.00291900,0.00344650,-0.00262724,-0.00068016,-
0.00451110,-0.00656329,-0.04616000,-0.22735180,-0.14317988,-0.00288765,-
0.00646077,-0.00688931,0.00161837,0.00278125,-0.00306939,-
0.00004717,0.00026917,0.00048913,0.00014182,-
0.00050695,0.00051966,0.00066646,-
0.00106533,0.00234175,0.05239518,0.23554020,-0.00001042,-
0.00000312,0.00000403,-0.00000463,-0.00002718,0.00003085,-0.00006704,-
0.00000532,-0.00007565,0.00005142,0.00010260,0.00010682,-0.00001619,-
0.00000869,-0.00009404,-0.00004755,0.00001953,0.00001919,-0.00003183,-
0.00004160,-0.00000547,-0.00001875,0.00002001,-0.00001698,0.00173132,-
0.00266646,0.00497267,0.00094357,0.00047416,0.00361346,-0.02802892,-
0.14287662,-0.14823573,-0.00473979,-0.01329978,-0.00668339,0.00395434,-
0.00297076,0.00205727,0.00034478,0.00046895,-0.00003365,-
0.00028092,0.00052401,-0.00065158,-0.00111703,0.00228993,-
0.00188787,0.02998468,0.15785635,0.14831387,-0.00003371,-
0.00001109,0.00000177,-0.00003449,0.00009647,-0.00004119,-0.00024818,-
0.00027670,-0.00002356,-0.00033708,0.00092724,0.00072422,-
0.00006419,0.00006893,-0.00006690,0.00001762,-0.00002786,-
0.00000470,0.00000878,0.00002455,0.00001755,0.00003016,-
0.00002375,0.00003138,-0.00300960,-0.00404904,0.00012747,-0.00015824,-
0.00071352,-0.00060777,0.00058865,-0.00242945,-0.00036242,0.00906402,-
0.00174980,-0.00301989,-0.21097716,0.07934582,0.12136050,-
0.01524302,0.01189548,0.01619198,0.00076517,0.00069928,-0.00101575,-
0.00009402,0.00023593,-0.00015220,0.00053736,-0.00035476,-
0.00023270,0.22063951,-0.00000389,-0.00000788,-0.00000091,-
0.00003955,0.00000934,-0.00005645,-0.00016105,-0.00009497,0.00005834,-
0.00029005,0.00035651,0.00037534,-0.00001452,-0.00001075,0.00000920,-
0.00002103,-0.00000063,-0.00003329,-0.00000598,-0.00001263,0.00002023,-
0.00003847,-0.00002356,0.00003444,-0.00363219,0.00218013,-0.00343032,-
0.00045665,-0.00047603,0.00009339,-0.00335037,-0.00150756,-
0.00477994,0.01928761,-0.00568115,-0.01427148,0.07789383,-0.09532644,-
0.07186977,-0.01037923,0.00903669,0.00799990,0.00103997,-
0.00110872,0.00223576,0.00022199,-0.00035932,0.00052009,0.00102211,-
0.00012893,-0.00098351,-
0.08337667,0.09361500,0.00000161,0.00000271,0.00000377,0.00001530,0.000052
89,0.00002124,-0.00000567,-0.00002303,-0.00009990,-
0.00014969,0.00012472,0.00014195,0.00004566,0.00005281,-

0.00007506,0.00002335,-0.00001578,0.00003198,0.00000325,0.00002845,-
0.00001503,0.00005545,-0.00000544,-0.00000267,0.00016906,-
0.00343533,0.00488677,-0.00062040,0.00003729,-0.00013749,-0.00074914,-
0.00496988,0.00166821,0.01425550,-0.00699415,-0.00637520,0.12001179,-
0.07275082,-0.13849026,-0.00231674,0.00052945,0.00479045,-
0.00069723,0.00246829,-0.00194497,-0.00013462,0.00054525,-
0.00059805,0.00087350,-0.00055911,-0.00002447,-
0.13189152,0.08196122,0.13799794,0.00998261,0.02579113,0.00225811,-
0.00436217,0.00308698,0.00053226,-0.00098873,0.00000453,0.00012015,-
0.00539980,-0.00078536,0.00023193,-0.00094261,-0.03371146,-0.00199313,-
0.10045494,-0.01437817,-0.01313066,0.00009875,-
0.00000949,0.00006883,0.00078001,-0.00028106,-0.00013781,0.00038147,-
0.00129261,-0.00022832,-0.00039842,-0.00002271,0.00008292,0.00002990,-
0.00004273,-0.00013430,-0.00003653,-0.00001914,0.00000366,-
0.00000460,0.00003168,0.00001003,0.00001518,0.00000063,-0.00007375,-
0.00000200,0.00000956,0.00004898,0.00002004,-0.00002698,-0.00001008,-
0.00003485,-0.00001622,0.00000225,-0.00001080,-
0.00000405,0.00000018,0.48844052,0.00846086,-
0.03176276,0.00188688,0.00328812,0.00360437,0.00019248,0.00004507,-
0.00089686,-0.00001144,-0.00093102,0.00479241,-0.00053117,-0.01643075,-
0.02036614,-0.00517663,-0.01433976,-0.21633919,-0.00652427,-
0.00000731,0.00006280,0.00003519,0.00196931,0.00044333,0.00147117,-
0.00084422,-0.00021355,0.00002437,0.00044247,0.00021799,0.00007324,-
0.00019853,-0.00001665,-0.00000841,0.00052427,0.00022457,0.00002355,-
0.00013566,-0.00012823,-0.00003410,0.00039966,0.00013914,0.00007888,-
0.00007515,0.00002511,0.00001697,-0.00002890,-0.00008334,-
0.00006335,0.00002366,0.00002845,0.00001057,0.00004201,0.00002303,-
0.00001619,0.00570358,0.63930972,0.00114894,-
0.00125940,0.00345198,0.00002136,0.00055486,0.00650667,-
0.00050348,0.00006944,-0.00096274,-0.00038185,-0.00074772,0.00640455,-
0.00039731,-0.00211807,0.00298627,-0.01290544,-0.00652120,-
0.06976180,0.00005347,0.00003443,-0.00069866,0.00014943,0.00025189,-
0.00342699,0.00005988,-0.00017547,0.00033052,0.00002194,0.00009754,-
0.00005465,0.00004180,-0.00000649,-0.00002377,-0.00001382,-0.00000760,-
0.00000217,0.00003191,0.00000844,0.00003423,0.00000031,0.00003292,-
0.00007826,0.00005631,0.00003671,-0.00000730,0.00002335,-0.00000189,-
0.00003019,0.00000388,-0.00000618,0.00001019,0.00000293,-
0.00000034,0.00000184,0.23740927,-0.01461508,0.34327597,-
0.00204251,0.00350802,-0.00135524,-0.00051084,-0.00071481,-
0.00005195,0.00005774,0.00063371,0.00004032,0.00033745,-
0.00109190,0.00016760,0.00251853,0.00285289,-0.00040763,0.00024560,-
0.01518190,-0.00237594,-0.00002061,-0.00000996,-0.00003616,-0.00019519,-
0.00003276,-0.00037609,0.00006472,0.00010771,0.00008638,-0.00001085,-
0.00002757,-0.00001091,0.00002429,0.00001009,0.00001243,-0.00003518,-
0.00001228,0.00000381,0.00000428,0.00001203,-0.00000102,-0.00003333,-
0.00000403,-0.00001988,-0.00001255,-0.00000731,0.00001997,-
0.00000371,0.00000264,-0.00000028,0.00000200,0.00000035,-
0.00000151,0.00000099,-0.00000009,0.00000029,-0.20130909,-0.06989893,-
0.10487871,0.46605710,-0.00298052,-0.00093003,-0.00187029,-
0.00040948,0.00032572,0.00101813,-0.00079708,-0.00016540,0.00002204,-
0.00091299,-0.00161727,0.00114564,0.00408574,-0.00280189,-0.00026196,-

0.02744692,-0.02024930,-0.01939966,-0.00000783,-0.00003937,-0.00026149,-
0.00044447,0.00020293,-0.00074207,0.00056174,-0.00024756,0.00002091,-
0.00031322,-0.00010790,-0.00003075,0.00011182,0.00000893,-0.00003857,-
0.00029098,-0.00012381,-0.00001032,0.00007448,0.00006801,0.00002631,-
0.00019028,-0.00007085,-0.00003608,0.00004277,0.00000537,-
0.00002424,0.00002615,0.00004190,0.00004012,-0.00001874,-0.00001858,-
0.00000422,-0.00002185,-0.00000661,0.00000523,-0.02519703,-0.14771631,-
0.01845886,-0.01446388,0.71700705,0.00000600,0.00021354,-0.00081677,-
0.00044114,0.00059462,-0.00006942,-
0.00042276,0.00006890,0.00015188,0.00021913,-0.00045742,-
0.00015205,0.00169861,0.00248163,0.00110524,-0.00145514,-
0.00631528,0.00315418,-0.00007343,-0.00000300,-0.00002448,-
0.00032023,0.00003966,-0.00012662,0.00005626,0.00008807,-0.00005565,-
0.00003461,-0.00001995,-0.00001003,0.00002661,-0.00000175,0.00001959,-
0.00004206,-0.00001257,0.00000042,0.00000672,0.00001390,0.00000494,-
0.00003261,-0.00003549,-0.00001741,-0.00000342,0.00000431,0.00000098,-
0.00000209,0.00001154,0.00000724,-0.00000064,0.00000150,-0.00000221,-
0.00000507,-0.00000259,-0.00000061,-0.10872630,-0.05686346,-
0.14782841,0.27676504,-0.04417912,0.36077093,0.00050967,-0.00359929,-
0.00072112,0.00069673,0.00049442,-0.00005897,0.00009465,-0.00069497,-
0.00004568,-0.00017968,0.00089064,-0.00021354,-0.00210944,-0.00311238,-
0.00120871,0.00920904,0.00721495,0.00340311,-
0.00001630,0.00000097,0.00003234,-0.00003213,-
0.00051682,0.00101124,0.00002709,-0.00000044,0.00002683,-
0.00001777,0.00000090,-0.00002482,-0.00000434,0.00000466,-0.00001826,-
0.00002339,-0.00000755,0.00000402,-0.00001928,-0.00000731,-0.00002696,-
0.00001234,0.00005146,0.00002115,0.00002896,-
0.00001883,0.00001427,0.00001513,-0.00001147,0.00002102,-0.00000149,-
0.00000791,0.00000369,0.00000120,0.00000004,0.00000622,-
0.17270988,0.08631263,-0.08874677,-0.02736513,-0.00298474,-
0.02948317,0.48141959,-0.00502673,-0.00051433,0.00009473,0.00029419,-
0.00187614,-0.00134142,0.00077339,-0.00019560,-
0.00005982,0.00062048,0.00015566,-0.00093295,0.00323235,-
0.00109194,0.00218750,0.01952562,-0.03166113,0.01593187,0.00000623,-
0.00007500,0.00025623,-0.00000781,-
0.00050723,0.00073306,0.00040491,0.00013346,-0.00000013,-0.00019469,-
0.00009004,-0.00003735,0.00007724,0.00001856,-0.00000491,-0.00026820,-
0.00011697,-0.00001343,0.00005910,0.00006345,-0.00000035,-0.00020786,-
0.00006037,0.00000038,0.00005086,-0.00002560,-
0.00001271,0.00002531,0.00003321,0.00005334,-0.00001101,-0.00001697,-
0.00000309,-0.00002133,-0.00001346,0.00001348,0.04173831,-
0.18813128,0.04103502,0.02897409,0.06030725,0.02094271,0.04158512,0.692916
25,0.00049270,-0.00305634,0.00082750,0.00038156,0.00022016,-0.00024199,-
0.00035475,-0.00000154,0.00014349,-0.00051092,-0.00043663,-
0.00022761,0.00026624,-0.00025650,-
0.00063396,0.00232094,0.00283999,0.00558194,-
0.00006961,0.00001687,0.00000385,-
0.00069346,0.00032017,0.00046846,0.00010041,-0.00004516,-0.00008640,-
0.00003301,-0.00002943,-0.00001684,0.00001154,-
0.00000058,0.00000097,0.00001810,0.00002122,0.00001278,-0.00003810,-
0.00000398,-0.00000717,-0.00001330,0.00001771,-0.00004287,0.00000479,-

0.00001552,0.00005776,-0.00000217,0.00000547,0.00001241,-
0.00000170,0.00000020,0.00000255,-0.00000687,-0.00000773,0.00000583,-
0.08797898,0.07984734,-0.13733867,-0.03096257,-0.00484854,-
0.01669910,0.28762195,0.00848045,0.36657034,0.00066777,-
0.00060618,0.00016974,0.00022025,0.00045766,0.00071658,-
0.00034192,0.00006595,-0.00000606,0.00023413,-0.00017805,0.00034387,-
0.00068360,0.00045401,-0.00004076,-0.00002692,0.00283084,-
0.00537166,0.00001328,0.00000738,-0.00012073,0.00028994,-0.00003490,-
0.00040202,-
0.00009060,0.00000526,0.00000451,0.00004133,0.00002218,0.00001070,-
0.00001304,-0.00000663,0.00000484,0.00004341,0.00001647,-
0.00000082,0.00000030,-
0.00001176,0.00000680,0.00004375,0.00001005,0.00000502,-
0.00001482,0.00001260,-0.00001159,-0.00000644,-0.00000518,-
0.00001482,0.00000237,0.00000311,0.00000024,0.00000557,0.00000412,-
0.00000243,0.03143599,0.01273061,0.01984617,-0.01829994,-0.02562848,-
0.00808686,-0.10507552,-0.00312791,-0.03728280,0.48375162,0.00082048,-
0.00185178,0.00023083,0.00037092,0.00057713,0.00019822,-
0.00025282,0.00005438,0.00010828,-0.00035569,0.00001198,0.00052582,-
0.00044526,0.00058962,-0.00101882,0.00266525,0.00374337,0.00158103,-
0.00003808,0.00004489,-0.00020598,-0.00045832,-0.00025237,-0.00047005,-
0.00016303,-0.00004613,0.00003604,0.00009008,0.00003474,0.00000865,-
0.00003875,0.00000119,0.00000798,0.00013137,0.00005586,0.00000815,-
0.00003847,-0.00001493,0.00000510,0.00007505,0.00000350,-0.00004209,-
0.00003224,-0.00000090,0.00003667,-0.00001260,-0.00001543,-
0.00001472,0.00000486,0.00000825,0.00000273,0.00000503,0.00000260,-
0.00000571,0.05033014,-0.04207541,0.04183690,-0.02357211,-0.04223763,-
0.01736537,-0.05575847,-0.34601934,-0.02988023,-
0.01706783,0.71460863,0.00056912,0.00138989,0.00003546,-0.00066760,-
0.00008569,0.00016530,0.00031023,-0.00023927,0.00001858,-
0.00007134,0.00052821,0.00015030,-0.00031215,-0.00087367,0.00004415,-
0.00555409,0.00162127,0.00191536,0.00001786,0.00002436,-0.00011122,-
0.00001147,-0.00015841,-0.00038987,-0.00002943,-
0.00000055,0.00003708,0.00002475,0.00000464,0.00000380,-
0.00001250,0.00001086,0.00000126,0.00000567,-0.00000219,-
0.00000179,0.00000308,0.00000894,0.00000013,0.00000374,-0.00002773,-
0.00000997,-0.00000338,-0.00000245,0.00000588,-
0.00000294,0.00000206,0.00000504,0.00000156,0.00000164,0.00000006,-
0.00000207,-0.00000147,-0.00000024,0.01966754,0.01299101,0.01976982,-
0.00849771,-0.01913003,-0.01198919,-0.03525282,0.01200615,-
0.08832528,0.28202354,-0.04339170,0.36582769,-
0.00015388,0.00067118,0.00015316,-0.00013521,-0.00024017,-
0.00030083,0.00022107,-0.00008450,-0.00007170,0.00004236,0.00022805,-
0.00034536,-0.00005844,-0.00067928,0.00028334,-0.00101143,-
0.00014098,0.00020045,0.00001389,-
0.00002014,0.00016439,0.00021373,0.00012257,0.00031055,0.00005503,0.000012
66,-0.00000601,-0.00002969,-0.00000960,0.00000082,0.00001090,0.00000176,-
0.00000432,-0.00004527,-0.00001994,-0.00000266,0.00001208,0.00000625,-
0.00000364,-0.00002873,-0.00000936,0.00001625,0.00001338,-0.00000338,-
0.00001326,0.00000638,0.00000527,0.00000582,-0.00000190,-0.00000266,-
0.00000106,-0.00000273,-0.00000078,0.00000104,-0.04014889,0.00738656,-

0.02664415,0.03009066,0.04682411,0.01556623,0.01121258,-
0.06205865,0.00518965,-0.20983307,-0.03076440,-0.11620528,0.49005508,-
0.00121812,-0.00014353,-0.00091838,-0.00048717,-0.00015738,0.00044116,-
0.00010708,-0.00055677,-0.00002115,0.00043255,-0.00030696,-
0.00034934,0.00120937,-0.00032239,0.00080559,-0.00009747,-0.00190233,-
0.00009325,-0.00001323,-0.00007808,-
0.00003426,0.00006262,0.00007601,0.00000283,0.00055529,0.00012049,-
0.00003124,-0.00030131,-0.00012701,-0.00003979,0.00013435,0.00001935,-
0.00000647,-0.00042668,-0.00018512,-
0.00002169,0.00011591,0.00008264,0.00000659,-0.00026423,-
0.00010429,0.00002712,0.00007628,-0.00001445,-
0.00006035,0.00003199,0.00005962,0.00006373,-0.00001544,-0.00002214,-
0.00001053,-0.00002372,-
0.00000841,0.00000932,0.00734307,0.00363557,0.00528819,0.00990137,-
0.04308105,0.01177956,-0.02471942,-0.01771032,-0.01984081,-0.07382669,-
0.14775398,-0.05489385,0.00646264,0.64333555,-
0.00020546,0.00050034,0.00025209,0.00002140,-0.00016160,-
0.00025486,0.00013524,-0.00002426,-0.00005266,0.00009243,0.00014339,-
0.00026080,-0.00021892,-0.00046555,0.00029037,-0.00028010,-0.00005134,-
0.00071920,0.00002113,-
0.00001396,0.00014053,0.00025690,0.00006131,0.00023803,0.00001679,0.000007
56,-0.00000347,-0.00000967,0.00000197,0.00000156,0.00000335,-0.00000160,-
0.00000266,-0.00001614,-0.00000719,-0.00000211,0.00000691,0.00000106,-
0.00000167,-0.00001012,0.00000068,0.00001176,0.00000841,-0.00000020,-
0.00001136,0.00000328,0.00000016,0.00000026,-0.00000114,-0.00000116,-
0.00000030,-0.00000133,-0.00000071,0.00000042,-0.02764251,0.00553020,-
0.02862813,0.01823975,0.04045094,0.01985154,0.00436354,-
0.04921530,0.01328935,-0.11381247,-0.01945539,-0.15268457,0.27833195,-
0.01857332,0.36406445,-0.00061852,-
0.00018095,0.00017933,0.00034579,0.00006752,0.00022175,-
0.00027175,0.00001831,-0.00003606,0.00003306,-
0.00038904,0.00060481,0.00046920,-0.00000585,0.00011394,-0.00101630,-
0.00098855,-0.00579006,0.00002335,0.00000500,-0.00006458,-
0.00001824,0.00004552,-0.00033837,0.00005471,-0.00004252,-0.00002199,-
0.00002732,-0.00000404,-0.00000738,0.00001477,-0.00000591,-0.00000313,-
0.00002062,-0.00000821,-0.00000247,0.00001290,0.00000126,0.00001346,-
0.00000623,0.00000261,-0.00000334,0.00000333,0.00000786,-
0.00001376,0.00000323,0.00000785,-0.00000109,-0.00000187,-0.00000184,-
0.00000024,-0.00000026,0.00000075,-0.00000079,0.01147444,-
0.02743688,0.00594188,-0.10547573,-0.05494908,-0.03499115,-
0.00789215,0.01709802,-0.00172654,-0.02955317,-0.00371698,-0.03042954,-
0.17782198,0.09264763,-
0.09458080,0.49665058,0.00077851,0.00030679,0.00103704,0.00028841,0.000022
66,-0.00061297,0.00029814,-0.00002503,-0.00010249,-0.00016052,0.00076621,-
0.00029607,-0.00143988,-0.00144131,-0.00044022,-
0.00081371,0.00491404,0.00008376,0.00003819,0.00001025,0.00024304,0.000365
63,0.00004752,0.00036020,-0.00018485,-
0.00003359,0.00004488,0.00009874,0.00005476,0.00002283,-0.00004951,-
0.00000242,-0.00000422,0.00011808,0.00004778,0.00000337,-0.00002771,-
0.00002254,-0.00000962,0.00007181,0.00001879,0.00001008,-
0.00001273,0.00000092,0.00000180,-0.00000417,-0.00002193,-

0.00001887,0.00000455,0.00000542,0.00000367,0.00000577,0.00000131,-
0.00000206,-0.06468948,-0.01494968,-0.04993968,-0.00308700,-
0.35073303,0.01049796,0.01461044,-
0.05620477,0.01417789,0.03030460,0.05966297,0.02209285,0.04930075,-
0.18938431,0.04413718,0.04148930,0.70650470,-
0.00002787,0.00093824,0.00024475,-0.00016635,-
0.00049415,0.00003823,0.00037964,0.00013955,0.00003029,-
0.00064091,0.00029996,0.00015537,0.00007312,-0.00165258,-0.00016788,-
0.00596037,0.00008618,0.00174947,0.00001723,-0.00001491,-0.00009002,-
0.00002222,0.00002911,-0.00009273,0.00002338,-0.00012956,-0.00002647,-
0.00003802,0.00000128,0.00002173,-0.00000480,-0.00000301,-0.00001744,-
0.00000402,-0.00000616,-
0.00000280,0.00000555,0.00000664,0.00000051,0.00000061,-
0.00001850,0.00000095,-0.00000056,0.00000116,-0.00000444,0.00000026,-
0.00000615,-0.00000271,-0.00000351,-0.00000144,-0.00000022,-
0.00000156,0.00000037,-0.00000175,0.00902926,-0.02189037,0.01300352,-
0.03782686,-0.03063346,-0.08896213,-0.00184764,0.01641510,-0.00841998,-
0.03205548,-0.00567742,-0.01704118,-0.09295350,0.07991104,-
0.14264523,0.28795327,0.00742849,0.37107353,0.00029101,0.00039718,0.000624
74,-0.00011319,-0.00062383,0.00014132,0.00048567,-0.00000392,0.00001020,-
0.00022243,0.00051289,-0.00005837,-0.00057446,-0.00083443,0.00000599,-
0.00072714,0.00241212,0.00190885,0.00002955,0.00000710,-
0.00005938,0.00007125,0.00002053,0.00000011,-0.00006424,-0.00005048,-
0.00000854,0.00003380,0.00001305,0.00001143,-0.00002248,0.00000054,-
0.00000551,0.00004818,0.00001894,-0.00000003,-0.00000980,-
0.00000707,0.00000239,0.00002941,-0.00000635,0.00000515,-
0.00000895,0.00000523,-0.00000637,-0.00000431,-0.00000616,-
0.00000304,0.00000089,0.00000276,0.00000054,0.00000034,0.00000160,-
0.00000327,-0.01063773,0.01429160,-0.01186688,-0.14837200,0.09817374,-
0.10038142,-0.00013746,-0.00308367,-0.00479054,-0.00023344,-
0.00088767,0.00002684,0.00386295,-0.00132234,-0.00309415,0.00577531,-
0.00347120,0.00307503,0.15185501,-0.00011984,-0.00046829,-0.00054617,-
0.00011036,-
0.00021604,0.00007209,0.00012409,0.00000453,0.00004543,0.00001311,0.000052
97,-0.00001178,0.00000089,0.00020362,-0.00000392,-0.00011168,0.00043246,-
0.00041507,0.00000681,0.00001343,-0.00000458,0.00002545,-0.00000218,-
0.00002338,-0.00004847,-
0.00000206,0.00002412,0.00003492,0.00000742,0.00000003,-
0.00001080,0.00000203,-0.00000128,0.00004042,0.00002021,0.00000404,-
0.00001281,-0.00001090,-0.00000271,0.00002675,0.00001877,-0.00000128,-
0.00000597,-0.00000122,0.00000643,-0.00000345,-0.00000677,-
0.00000395,0.00000244,0.00000223,0.00000084,0.00000327,0.00000073,-
0.00000040,-0.00752813,0.01055724,-0.00663954,0.09904221,-
0.16028283,0.09187491,-0.00219673,-0.00064556,-0.00163490,-0.00069891,-
0.00040411,-0.00048058,-0.00243970,-0.00485525,-0.00134405,0.02033480,-
0.01525403,0.01773921,-0.10689180,0.17060657,-0.00108302,-
0.00045599,0.00005689,0.00055429,-0.00001993,0.00012518,-
0.00008105,0.00006664,-0.00005515,0.00017070,-
0.00007323,0.00008900,0.00010782,0.00028501,0.00018919,0.00202846,0.000137
22,-0.00159236,0.00005343,0.00002762,0.00001132,0.00009272,0.00004176,-
0.00005623,-0.00005916,-0.00002420,-

0.00002772,0.00003943,0.00001084,0.00000603,-0.00001783,-0.00000657,-
0.00000259,0.00005842,0.00002473,0.00000163,-0.00000981,-
0.00001872,0.00000370,0.00004569,0.00003552,0.00001181,-
0.00000730,0.00000238,-0.00000384,-0.00000326,-0.00000384,-
0.00000794,0.00000156,0.00000244,0.00000086,0.00000608,0.00000309,-
0.00000145,-0.00971464,0.01272397,-0.00586954,-0.10142977,0.09153484,-
0.12682233,-0.00465845,-0.00233786,0.00221057,0.00019330,-0.00039495,-
0.00016864,-0.00321336,-0.00060394,0.00489883,0.00183244,-
0.00263748,0.00422591,0.11229432,-0.09791793,0.12613997,-0.00004555,-
0.00000092,0.00004063,0.00002330,0.00001850,-0.00002462,-0.00000792,-
0.00001111,-0.00000223,-0.00002574,0.00003545,-0.00002350,0.00005790,-
0.00001863,0.00005525,-0.00008727,0.00015312,-0.00001580,0.00000094,-
0.00000185,0.00000999,-0.00001411,0.00001860,0.00001062,0.00001482,-
0.00000070,-0.00000721,-0.00000852,-
0.00000258,0.00000210,0.00000249,0.00000005,-0.00000022,-0.00000966,-
0.00000435,-0.00000054,0.00000378,0.00000292,0.00000094,-0.00000776,-
0.00000916,-0.00000024,0.00000225,0.00000098,-
0.00000360,0.00000042,0.00000195,0.00000139,-0.00000052,-0.00000017,-
0.00000042,-0.00000164,-0.00000052,-0.00000063,0.00398714,-0.00022133,-
0.00303503,0.00233385,0.00039805,-
0.00104811,0.00013608,0.00028848,0.00040525,0.00123737,0.00324013,-
0.00367743,-0.01047517,-0.00786677,-0.01147749,-0.20108741,-0.08051016,-
0.12789142,-0.00122551,-0.00003107,0.00243217,0.20740571,-0.00007824,-
0.00002670,0.00006030,0.00002635,-0.00000840,0.00001610,-0.00000404,-
0.00003320,-0.00000411,-0.00010180,-0.00000103,0.00006592,0.00010199,-
0.00039771,-0.00003830,-0.00110941,0.00017343,-0.00077202,0.00000188,-
0.00000529,-0.00000447,-0.00001348,0.00001498,-0.00002198,0.00003681,-
0.00002795,-0.00000027,-0.00002107,-0.00000419,0.00000077,0.00000537,-
0.00000023,-0.00000362,-0.00001646,-0.00000754,-
0.00000148,0.00000490,0.00000438,0.00000214,-0.00001225,-0.00000747,-
0.00000148,0.00000362,0.00000072,-
0.00000361,0.00000228,0.00000219,0.00000191,-0.00000184,-0.00000122,-
0.00000035,-0.00000214,-0.00000046,-0.00000012,0.00019698,-
0.00523857,0.00072387,-0.02273455,-0.00711240,-0.01822946,0.00054418,-
0.00082113,0.00040486,0.00231402,-
0.00288712,0.00222035,0.01390280,0.01274489,0.01087513,-0.08199911,-
0.10310037,-
0.06353474,0.00034376,0.00111507,0.00001119,0.08753419,0.10503044,0.000015
25,-0.00006165,-0.00007799,-0.00002264,-0.00001657,0.00003411,0.00000177,-
0.00000560,0.00000317,0.00003128,-0.00004269,0.00002267,-0.00001443,-
0.00000857,-0.00010306,0.00009831,0.00006567,0.00007530,-0.00000130,-
0.00000146,-0.00001461,0.00002180,-0.00001520,-
0.00001970,0.00000456,0.00000391,0.00001074,-0.00000059,-0.00000133,-
0.00000222,0.00000215,0.00000091,-0.00000010,-0.00000475,-0.00000199,-
0.00000012,0.00000068,0.00000004,-0.00000002,-
0.00000112,0.00000372,0.00000187,-0.00000054,-0.00000090,-
0.00000022,0.00000040,0.00000008,0.00000041,0.00000001,-0.00000050,-
0.00000027,0.00000102,0.00000058,0.00000012,-
0.00302518,0.00070566,0.00530419,-
0.00021772,0.00071811,0.00336851,0.00037895,0.00044625,-0.00008198,-
0.00372871,0.00262547,0.00240313,-0.01182698,-0.00697315,-0.00528648,-

0.12827633,-0.06196872,-0.14074796,0.00250956,-0.00036369,-
0.00218447,0.14235744,0.06763709,0.14001961,-
0.00032374,0.00097644,0.00010289,-0.00033662,-0.00040583,-
0.00010477,0.00043185,-0.00010595,-
0.00000535,0.00004593,0.00054170,0.00017805,0.00018867,-
0.00069992,0.00069700,-0.00132920,-0.00212758,0.00172474,0.00002507,-
0.00000303,-0.00005731,0.00029772,-
0.00065381,0.00000388,0.00004018,0.00002262,0.00001873,-0.00000629,-
0.00000530,0.00000509,0.00000303,0.00000731,0.00000153,-0.00003277,-
0.00001802,-0.00000362,0.00000489,0.00001758,-0.00000017,-0.00003063,-
0.00003447,-0.00001359,0.00000837,-0.00000489,0.00001238,-
0.00000118,0.00000700,0.00000445,-0.00000072,-0.00000032,-0.00000101,-
0.00000648,-0.00000435,0.00000268,-0.01063772,-0.00748030,-
0.01077084,0.00118500,0.00301359,-0.00394880,-0.19352069,-0.07454686,-
0.12979852,0.00030762,-0.00031219,-0.00152314,0.00429409,-0.00076522,-
0.00272033,0.00010419,0.00074914,0.00024682,-0.00049079,-0.00048374,-
0.00070873,-0.00045248,-0.00005650,0.00053115,0.20123595,0.00012847,-
0.00001472,-0.00001468,0.00006591,0.00015566,0.00003554,-
0.00022274,0.00007207,-0.00003461,-0.00001801,-0.00035406,-
0.00015983,0.00004931,-
0.00041522,0.00028670,0.00027118,0.00095131,0.00020693,-
0.00001500,0.00002002,0.00002029,-0.00015705,-0.00092642,0.00089464,-
0.00006014,-0.00002323,0.00006513,0.00004677,-0.00000113,-0.00000371,-
0.00001393,0.00000142,0.,0.00006456,0.00003305,0.00000936,-0.00002767,-
0.00001849,-0.00000440,0.00003462,0.00003381,-0.00000104,-
0.00001747,0.00000137,0.00001346,-0.00000439,-0.00000798,-
0.00000622,0.00000316,0.00000459,0.,0.00000623,0.00000453,-
0.00000385,0.01427268,0.01068832,0.01148678,0.00210376,-
0.00253609,0.00230113,-0.07521263,-0.09817542,-0.06162930,-0.02423674,-
0.00800545,-0.01956862,0.00040968,-0.00552449,0.00100108,0.00052564,-
0.00083593,0.00039119,0.00084008,0.00057225,0.00067479,-
0.00004213,0.00005714,-0.00009761,0.08224341,0.10336421,0.00004180,-
0.00031775,0.00015627,0.00018908,0.00002964,0.00009847,-0.00011470,-
0.00002935,-0.00006492,0.00054101,-0.00016912,0.00010461,-
0.00093740,0.00061867,0.00017661,0.00201877,-0.00036826,-
0.00162986,0.00004353,-0.00003921,0.00000897,0.00078165,0.00060586,-
0.00043923,-0.00000269,0.00005142,-0.00000402,-
0.00002675,0.00001200,0.00000174,0.00000556,-0.00001244,-0.00000469,-
0.00005066,-0.00002814,-0.00001181,0.00003514,-0.00000340,0.00000443,-
0.00000824,0.00001148,0.00005922,0.00000593,0.00001846,-
0.00006507,0.00000748,-0.00000483,-0.00000504,-0.00000215,-0.00000554,-
0.00000134,0.00000469,0.00000570,-0.00000123,-0.01112295,-0.00813671,-
0.00606888,-0.00373810,0.00296664,0.00267817,-0.13092539,-0.06105543,-
0.14345592,-0.00018136,0.00088176,0.00228833,-
0.00292862,0.00025642,0.00502399,0.00036952,0.00028501,-0.00010397,-
0.00077426,-0.00041206,-0.00008357,0.00052001,-0.00006905,-
0.00065628,0.14384007,0.06508398,0.14402880,0.00001377,-
0.00008057,0.00005696,-0.00000589,-0.00003112,-0.00004169,-
0.00000361,0.00001258,-0.00000047,0.00000764,-0.00001989,-0.00002650,-
0.00002934,0.00000926,0.00002254,0.00015241,-
0.00017304,0.00013456,0.00000062,0.00000044,0.00001049,-0.00000496,-

0.00004679,0.00002076,0.00000239,0.00000031,-
0.00000119,0.00000388,0.00000112,-0.00000053,-
0.00000258,0.00000036,0.00000092,0.00000529,0.00000258,0.00000029,-
0.00000117,0.00000126,-0.00000076,-0.00000317,-0.00000073,-
0.00000573,0.00000199,-0.00000117,0.00000466,-
0.00000105,0.00000059,0.00000049,0.00000028,0.00000059,0.00000062,-
0.00000191,-0.00000252,0.00000157,0.00351026,-0.00158304,-0.00329924,-
0.00007609,-0.00045905,0.00023525,0.00682907,-0.00430335,0.00295845,-
0.15505046,0.10347559,-0.10050280,-0.01021611,0.01508642,-0.01143861,-
0.00016477,-0.00348530,-0.00466146,-0.00044682,0.00011124,0.00057750,-
0.00030991,0.00090898,-0.00083659,-
0.00116386,0.00034213,0.00240952,0.15752251,-0.00020817,-0.00037160,-
0.00001130,0.00010440,-0.00001378,-0.00006690,-0.00000518,-0.00003898,0.,-
0.00002643,0.00000412,-0.00001217,0.00006623,-0.00004475,-
0.00006791,0.00113623,-0.00013299,0.00078473,-0.00000328,-
0.00000651,0.00000308,-
0.00002595,0.00001022,0.00003990,0.00005234,0.00000266,0.00000033,-
0.00002502,-0.00000611,-0.00000482,0.00000696,0.00000176,-0.00000161,-
0.00002735,-0.00001199,-0.00000188,0.00000517,0.00000861,-0.00000163,-
0.00002606,-0.00000623,-0.00000709,0.00000982,-
0.00000530,0.00000426,0.00000359,0.00000197,0.00000682,-0.00000177,-
0.00000276,0.00000074,-0.00000396,-0.00000415,0.00000353,-0.00213134,-
0.00471476,-0.00105256,-0.00071101,-0.00044279,-0.00051601,0.01935684,-
0.01397420,0.01686987,0.10510133,-0.16530753,0.09177952,-
0.00750457,0.01188647,-0.00651704,-0.00258437,-0.00071000,-
0.00169895,0.00012063,0.00001886,0.00000762,-0.00051956,0.00063730,-
0.00040435,-0.00004486,0.00107099,-0.00028985,-0.11163044,0.17395201,-
0.00002118,-0.00000202,-
0.00009540,0.00005053,0.00002959,0.00001637,0.00000253,0.00000656,0.000004
85,-0.00002255,0.00001587,0.00003325,0.00003295,0.00006286,-
0.00007837,0.00015863,-0.00010990,0.00015737,-0.00000090,0.00000360,-
0.00001361,-0.00002210,0.00005120,0.00000202,-0.00002084,-
0.00000560,0.00000236,0.00000771,0.00000290,-0.00000043,-0.00000337,-
0.00000146,-0.00000176,0.00001305,0.00000569,0.00000075,-0.00000633,-
0.00000499,-0.00000032,0.00001448,0.00001028,0.00000530,-
0.00000548,0.00000175,-0.00000037,0.00000016,-0.00000381,-
0.00000212,0.00000032,-0.00000028,0.00000028,0.00000310,0.00000290,-
0.00000067,-0.00331316,-0.00094035,0.00510894,0.00017666,-0.00054715,-
0.00013861,0.00147977,-0.00240077,0.00484728,-0.10098196,0.09026288,-
0.12469842,-0.00968034,0.01174909,-0.00557789,-0.00470683,-
0.00206990,0.00208800,0.00059933,0.00003661,-0.00069596,-
0.00077191,0.00071490,0.00005703,0.00252322,0.00006445,-
0.00211538,0.11232904,-0.09835751,0.12261200,0.00604636,0.01262666,-
0.00251922,-0.17465051,0.05722202,0.00988638,-
0.03615911,0.01792817,0.00350526,-0.00111219,-0.00515895,-0.00026780,-
0.00093194,-0.00004188,-0.00007457,0.00295169,-0.00382923,-
0.00079009,0.00144952,0.00127327,-0.00062609,0.00007727,0.00001881,-
0.00003028,-
0.00099844,0.00050352,0.00008360,0.00028326,0.00014509,0.00002011,-
0.00012363,-0.00002514,0.00000449,0.00034844,0.00015662,0.00001615,-
0.00011405,-0.00005734,0.00009181,0.00013074,-0.00007144,-0.00009963,-

0.00006250,0.00004992,0.00005234,-0.00001629,-0.00005518,-
0.00004801,0.00002462,0.00001183,0.00001741,-0.00000129,-0.00000796,-
0.00000707,0.00085782,0.00055652,0.00016519,-0.00006044,-
0.00036098,0.00007411,-0.00011412,-0.00060664,-
0.00002769,0.00010086,0.00015191,0.00006843,-0.00005888,-0.00050990,-
0.00002281,-0.00005397,0.00016412,-0.00001734,0.00004608,0.00004538,-
0.00000796,-0.00000258,-0.00004587,-0.00001808,-0.00003583,0.00006302,-
0.00004436,0.00000936,-0.00004974,0.00000384,0.58700263,0.02976216,-
0.02764487,0.00054132,0.05722341,-
0.14336367,0.00600538,0.00081460,0.01459766,-0.00191699,-
0.00501485,0.00049113,-0.00089681,-0.00005875,-0.00097975,-0.00009777,-
0.00400710,-0.00361330,-0.00066360,-0.00107109,-
0.00028904,0.00113349,0.00002066,0.00008870,-
0.00000896,0.00004208,0.00115697,0.00020201,-0.00026236,-
0.00013057,0.00009182,0.00014641,-0.00000836,-0.00002062,-0.00038683,-
0.00017441,-0.00002357,0.00008741,0.00012694,0.00010098,-0.00052326,-
0.00030466,-0.00004266,0.00005401,-0.00000208,-
0.00003424,0.00002958,0.00004384,-0.00002234,-0.00003815,-0.00001885,-
0.00001898,-0.00004651,-0.00001077,-0.00000208,0.00103626,-
0.00069879,0.00005743,0.00004375,0.00021460,0.00004850,-0.00014916,-
0.00004939,-0.00010227,0.00000929,-
0.00009269,0.00011444,0.00003165,0.00024380,0.00000490,-0.00000324,-
0.00007681,-0.00000109,-0.00003987,-0.00001380,-0.00005027,-
0.00000069,0.00001342,0.00000899,0.00006039,-0.00003133,0.00003793,-
0.00000891,-0.00001513,-0.00000899,-0.06263375,0.56416930,0.00085693,-
0.00175468,0.00332351,0.00974193,0.00586207,-0.06829367,-0.00013922,-
0.00003291,0.00316451,0.00031484,-
0.00063010,0.00639332,0.00029853,0.00039798,-0.00094387,-0.00067584,-
0.00003559,0.00645425,-0.00003508,0.00020889,-0.00341029,-
0.00002435,0.00000481,-0.00070563,0.00002055,-0.00011600,0.00034090,-
0.00001316,0.00002814,-0.00007681,-0.00003112,-
0.00001877,0.00003275,0.00001976,0.00000600,-0.00000186,-0.00002625,-
0.00003309,-0.00001779,-0.00009217,0.00004081,-0.00006345,-0.00001423,-
0.00001455,-0.00002968,-0.00006463,-0.00001533,-0.00001314,-0.00000136,-
0.00000224,0.00000173,-0.00000046,-0.00000827,0.00000992,-0.00010878,-
0.00007579,0.00033975,0.00003179,0.00002303,-0.00007896,0.00006260,-
0.00004584,-0.00007124,-0.00003026,-0.00004466,-
0.00001767,0.00000518,0.00005648,0.00000051,-0.00000581,-
0.00004784,0.00003217,-0.00000228,-0.00006927,-0.00001741,-
0.00000164,0.00000046,0.00000318,-0.00000785,-0.00002186,-0.00002627,-
0.00000810,-0.00000067,0.00000944,-0.14602643,-0.17686683,0.31980427,-
0.00442558,-0.00015844,0.00017243,0.00263549,0.02708156,-0.01323698,-
0.00146361,0.00268189,-0.00067913,0.00066257,-0.00030972,0.00085599,-
0.00002663,0.00086213,0.00007953,-0.00024690,0.00117508,0.00101700,-
0.00047699,0.00050493,-0.00128291,-0.00003328,0.00003193,-
0.00020582,0.00028291,0.00015950,0.00000403,-0.00011502,-0.00011058,-
0.00002069,0.00004703,-0.00000937,0.00002472,-0.00020184,-0.00009104,-
0.00001419,0.00004424,0.00002575,0.00001996,-0.00015008,-
0.00006340,0.00003479,0.00003324,0.00001046,-
0.00005184,0.00001648,0.00006372,-0.00000379,-0.00001691,-0.00000576,-
0.00001787,-0.00001657,0.00000413,-0.00000343,-0.00045791,-0.00026017,-

0.00003329,0.00002164,0.00016039,-0.00000900,0.00006296,0.00027252,-
0.00000059,-0.00004203,-0.00007209,-
0.00004359,0.00002968,0.00025481,0.00000979,0.00001591,-
0.00008240,0.00001602,-0.00001526,-0.00003939,-
0.00003365,0.00000489,0.00001928,0.00000584,0.00001973,-
0.00004155,0.00003095,-0.00000545,0.00002313,-0.00000077,-0.11853444,-
0.02024254,0.03368566,0.65426070,0.00106491,0.00436593,0.00073000,0.014161
77,-0.02446366,0.00874873,-0.00376949,-0.00170550,0.00216917,-0.00000965,-
0.00075097,-0.00052055,-0.00060406,-0.00009382,-0.00004474,0.00145271,-
0.00094719,-0.00083847,-0.00002142,-0.00002978,-0.00021133,0.00001636,-
0.00005285,0.00015869,-0.00024201,-0.00011721,-
0.00003246,0.00014957,0.00004259,-0.00000535,-0.00007439,-
0.00000633,0.00001320,0.00016582,0.00007078,0.00000446,-0.00004227,-
0.00003193,0.00000499,0.00012273,0.00004147,0.00000075,-
0.00002713,0.00001585,0.00002065,-0.00000580,-0.00001256,-
0.00001893,0.00001692,0.00000718,0.00000839,0.00000705,0.00000176,-
0.00000242,0.00018518,0.00028955,-0.00006183,-0.00002551,-
0.00013272,0.00002633,-0.00001427,-
0.00018634,0.00001027,0.00002856,0.00007409,0.00001198,-0.00002588,-
0.00019054,-0.00001361,-0.00001930,0.00005913,-
0.00000692,0.00000774,0.00000741,-0.00000503,-0.00000212,-0.00001055,-
0.00000359,-0.00001684,0.00002187,-0.00001793,0.00000400,-
0.00001130,0.00000254,0.02616217,-0.24818246,0.09028683,-
0.09209731,0.55281598,0.00160013,-0.00240962,0.00088105,-
0.00348016,0.00099354,0.00496247,-0.00013478,-0.00028708,-
0.00061361,0.00070914,0.00003967,-
0.00016722,0.00022511,0.00027934,0.00016492,-0.00037519,-0.00007580,-
0.00023878,0.00022775,0.00073512,0.00041923,0.00003596,0.00006159,0.000001
19,-0.00002967,-0.00009784,-0.00009001,-0.00000295,0.00001817,-
0.00004388,0.00003128,0.00002519,-0.00000375,-
0.00003084,0.00000005,0.00000960,-0.00000791,-
0.00000893,0.00000146,0.00004324,0.00000152,-0.00001281,-
0.00000236,0.00000606,0.00001083,0.00000528,-
0.00001315,0.00005748,0.00000914,-
0.00000156,0.00000541,0.00000089,0.00000077,0.00000162,0.00007912,-
0.00005277,-0.00007030,-0.00001807,0.00002956,-0.00001015,-
0.00000386,0.00001450,-0.00000560,-0.00001028,-
0.00001481,0.00002077,0.00000689,0.00001664,0.00000212,0.00000676,-
0.00000496,0.00000251,0.00000695,0.00000267,0.00000163,-
0.00000017,0.00000399,-0.00000074,0.00000914,-
0.00000108,0.00000382,0.00000201,-0.00000176,-
0.00000208,0.00650252,0.11451607,-0.13024239,-0.19594012,-
0.19783041,0.33684886,-0.00040870,0.00196772,-0.00025963,0.00442925,-
0.00227158,0.00267809,-0.00012579,-0.00111588,0.00072478,-
0.00008834,0.00001382,-0.00063786,-0.00023235,-0.00033602,-
0.00009044,0.00082620,-0.00022910,-0.00060732,-
0.00025676,0.00005659,0.00063289,0.00000285,-0.00004271,0.00023663,-
0.00014184,-0.00010982,-0.00004126,0.00006294,0.00005498,0.00003051,-
0.00003173,-0.00000665,-0.00001153,0.00011794,0.00005488,0.00000633,-
0.00002477,-0.00002326,-0.00001165,0.00009129,0.00003849,-0.00000768,-
0.00002041,0.,0.00001979,-0.00001610,-0.00002915,-

0.00001795,0.00000843,0.00000400,0.00000826,0.00000783,-0.00000073,-
0.00000008,0.00008420,0.00016331,0.00006037,-0.00001982,-
0.00006420,0.00000578,-0.00000515,-
0.00011129,0.00001496,0.00002583,0.00004351,0.00000003,-0.00001687,-
0.00011985,-0.00000556,-0.00000267,0.00003731,-
0.00001179,0.00000392,0.00001182,0.00000919,-0.00000198,-0.00000464,-
0.00000185,-0.00001811,0.00001732,-0.00001381,0.00000251,-
0.00000712,0.00000254,0.02901980,0.05710985,-0.04193684,-
0.26351890,0.09305965,0.04111193,0.60822091,0.00060610,-
0.00070438,0.00005226,-0.00210465,-0.00120567,0.00479983,-0.00017089,-
0.00003022,-0.00068891,0.00019575,0.00037211,0.00013452,-0.00003691,-
0.00004430,0.00007714,-0.00013072,-0.00002495,-
0.00038826,0.00047099,0.00026910,-0.00000397,0.00001140,0.00004400,-
0.00005330,0.00005342,0.00000600,0.00002416,-0.00000971,-0.00001699,-
0.00003248,0.00002019,0.00001793,0.00000051,-0.00005509,-
0.00002308,0.00000077,0.00000549,0.00001429,0.00000299,-0.00002334,-
0.00001588,-0.00000405,0.00000735,-
0.00000181,0.00000080,0.00001296,0.00000084,0.00003282,0.00000068,-
0.00000134,-0.00000301,-0.00000254,0.00000050,0.00000050,0.00003516,-
0.00006899,-0.00000795,0.00000649,0.00003108,0.00000013,-
0.00000988,0.00003159,-0.00000406,-0.00000027,-
0.00001453,0.00000500,0.00000641,0.00004301,0.00000426,0.00000715,-
0.00000589,-0.00000759,-0.00000760,-
0.00000058,0.00001176,0.00000008,0.00000262,0.00000210,0.00000473,-
0.00000256,0.00001027,-0.00000080,0.00000090,-0.00000148,0.01866496,-
0.03637414,0.01456868,0.14686829,-0.19657822,0.01024713,-
0.10679841,0.62710713,-0.00140930,0.00063888,-
0.00003242,0.00278526,0.00494925,0.00242922,0.00082745,-
0.00042542,0.00008872,-0.00032413,0.00041752,0.00012099,-0.00006237,-
0.00039270,0.00000867,0.00054611,0.00039956,0.00015500,0.00013412,-
0.00008847,-0.00036047,-0.00002417,0.00000570,-
0.00010316,0.00001859,0.00001720,0.00003680,0.00001722,-0.00002104,-
0.00000891,-0.00000824,0.00000438,-0.00000057,-0.00000114,-0.00000479,-
0.00000184,0.00000070,0.00001602,0.00000116,-0.00001923,-
0.00001331,0.00000198,0.00000032,0.00000297,0.00000527,0.00000395,0.000000
08,0.00000625,0.00000239,0.00000033,0.00000004,-0.00000203,-0.00000027,-
0.00000017,-0.00012526,0.00003472,-0.00002113,-
0.00001502,0.00001355,0.00000222,0.00002031,0.00003724,0.00002305,-
0.00000171,0.00000433,-0.00001644,-0.00000231,0.00001475,-
0.00000164,0.00000365,-0.00001005,-0.00000060,0.00000040,-0.00000019,-
0.00000378,0.00000111,0.00000165,-0.00000148,-0.00000452,0.00000225,-
0.00000091,0.00000041,0.00000410,-0.00000009,-0.02244083,-
0.00336310,0.01687236,0.01157893,0.03516259,-0.08492632,-0.15588187,-
0.22707577,0.33863840,-0.00047269,-0.00099655,0.00049963,-
0.00158220,0.00045002,-0.00010813,0.00007929,0.00114479,-
0.00076849,0.00017805,0.00027003,0.00054159,-
0.00028014,0.00039030,0.00005956,-0.00051148,-0.00011153,-
0.00012907,0.00025550,0.00004718,-0.00022814,-0.00004500,0.00005348,-
0.00010069,0.00037785,0.00025557,0.00005398,-0.00021134,-0.00009903,-
0.00004752,0.00011678,0.00002575,0.00000755,-0.00034474,-0.00015230,-
0.00001615,0.00007803,0.00006440,0.00001665,-0.00023476,-

0.00010354,0.00001109,0.00005580,-0.00001236,-
0.00004875,0.00003417,0.00005454,0.00005667,-0.00001780,-0.00001537,-
0.00001365,-0.00001743,-0.00000013,0.00000321,-0.00014253,-0.00043916,-
0.00005716,0.00003376,0.00022667,0.00003400,0.00003075,0.00026855,-
0.00001308,-0.00004208,-0.00010372,-
0.00001083,0.00004283,0.00037653,0.00001304,0.00001230,-0.00011628,-
0.00000298,-0.00004098,-0.00004217,-
0.00004840,0.00000396,0.00001944,0.00000920,0.00003214,-
0.00005003,0.00004709,-0.00000948,0.00002035,-0.00000650,-0.01241817,-
0.02332996,0.01528602,-0.04824933,0.03400584,0.00786873,-0.24076458,-
0.06043660,0.11399864,0.58828509,0.00092241,0.00012440,-
0.00069495,0.00045281,-0.00134402,-0.00019704,-0.00084752,-
0.00040249,0.00029721,0.00008552,-0.00046605,0.00002129,0.00038313,-
0.00004852,0.00005940,0.00009493,0.00021118,0.00051120,0.00011391,0.000054
33,-0.00022566,0.00004779,-0.00001030,-0.00014108,-0.00028287,-0.00018750,-
0.00003828,0.00015763,0.00007073,0.00001837,-0.00008862,-
0.00001726,0.,0.00025331,0.00011233,0.00001215,-0.00006101,-0.00005043,-
0.00000727,0.00017805,0.00007483,-0.00001476,-
0.00003955,0.00001288,0.00003710,-0.00002238,-0.00003364,-
0.00003290,0.00001307,0.00001096,0.00000999,0.00001233,0.,-
0.00000254,0.00010074,0.00031364,0.00004425,-0.00002760,-0.00016247,-
0.00004158,-0.00002557,-
0.00018880,0.00000357,0.00002892,0.00007082,0.00001210,-0.00003064,-
0.00027305,-0.00000883,-
0.00000596,0.00008221,0.00000731,0.00003536,0.00003483,0.00004419,-
0.00000319,-0.00001426,-0.00000674,-0.00002009,0.00003872,-
0.00003273,0.00000706,-0.00001528,0.00000464,-0.02345855,-
0.02673789,0.02150008,-0.00421067,0.04122742,-0.01738280,-0.01531567,-
0.12851106,0.04264817,-0.06307220,0.57288493,-
0.00018742,0.00044508,0.00025732,0.00016285,0.00019629,-
0.00071563,0.00047139,-0.00017100,0.00027823,-0.00016075,0.00003629,-
0.00026108,-0.00004867,-0.00012129,-0.00005537,0.00009813,-0.00010699,-
0.00022751,-0.00021526,-0.00013747,0.00021843,-0.00001161,-
0.00002648,0.00013143,-0.00002262,-0.00000832,-
0.00000379,0.00000919,0.00000908,0.00001277,-0.00000684,-0.00000454,-
0.00000210,0.00002043,0.00000867,-0.00000025,-0.00000191,-0.00000384,-
0.00000277,0.00000745,0.00000829,0.00000090,-0.00000345,-
0.00000190,0.00000136,-0.00000537,-0.00000645,-
0.00001208,0.00000148,0.00000107,0.00000087,0.00000148,0.00000029,0.000000
03,0.00001211,0.00002545,0.00000112,0.00000238,-
0.00001493,0.00000731,0.00000327,-
0.00001914,0.00000277,0.00000091,0.00000817,-0.00000128,-0.00000249,-
0.00002000,-0.00000186,-0.00000367,0.00000504,-0.00000145,-0.00000034,-
0.00000145,-0.00000619,-0.00000024,-0.00000041,-0.00000029,-
0.00000442,0.00000227,-0.00000449,0.00000046,-
0.00000046,0.00000042,0.01598903,0.02223333,-0.02603648,0.02804177,-
0.03523513,0.01393772,0.08797866,0.06362721,-0.14059660,-0.16926693,-
0.21146709,0.33621689,0.00021637,-0.00000476,-0.00087158,0.00072572,-
0.00314040,0.00383330,-0.00127998,0.00026975,0.00018791,0.00013327,-
0.00028348,-
0.00021054,0.00002892,0.00002329,0.00009898,0.00032413,0.00028692,0.000303

51,0.00022326,0.00013040,-0.00000578,0.00004598,0.00002639,-0.00012198,-
0.00011278,-0.00003004,-0.00002273,0.00004793,0.00002653,-0.00000091,-
0.00002117,0.00000172,-0.00000436,0.00007564,0.00003570,0.00000683,-
0.00002278,-0.00001571,0.00000351,0.00006495,0.00001292,-0.00000845,-
0.00000960,0.00000656,0.00001476,-0.00000121,-
0.00001033,0.00000741,0.00000365,0.00000300,0.00000351,0.00000270,0.000000
15,-0.00000165,0.00001257,0.00010243,0.00003855,-0.00002568,-0.00003259,-
0.00004134,-0.00001233,-0.00005993,-
0.00000234,0.00001806,0.00002106,0.00000575,-0.00001121,-0.00010025,-
0.00000077,0.00001077,0.00003210,0.00000334,0.00000566,0.00001805,0.000039
25,-0.00000079,-0.00000110,-0.00000388,-0.00000561,0.00001249,-
0.00000395,0.00000348,-0.00000568,0.00000171,-0.04990290,-
0.00616381,0.02831650,-0.01594862,0.02321660,-0.00887766,0.02748781,-
0.02846322,0.00714214,-0.11619369,-0.02075992,0.03700828,0.65888168,-
0.00092800,-0.00047185,0.00053003,-
0.00332527,0.00257503,0.00405067,0.00165058,0.00026257,-0.00030292,-
0.00045656,0.00067516,-0.00065975,-0.00025428,-0.00030854,-
0.00006782,0.00008125,0.00001637,-0.00057460,-0.00020998,-
0.00019809,0.00048732,-0.00002114,-
0.00000843,0.00021722,0.00011023,0.00012748,0.00005729,-0.00004816,-
0.00003366,0.00000488,0.00003503,0.00001117,-0.00001361,-0.00009717,-
0.00004574,-0.00000494,0.00002801,0.00003438,0.00000274,-0.00009146,-
0.00003582,0.00001478,0.00001920,-0.00001077,-
0.00001177,0.00001105,0.00000456,0.00001239,-0.00000318,-0.00000458,-
0.00000262,-0.00000563,-0.00000067,0.00000114,-0.00006564,-0.00014620,-
0.00003509,0.00003129,0.00006730,0.00002195,0.00000885,0.00009056,-
0.00000650,-0.00001248,-0.00002866,-
0.00001033,0.00001534,0.00010978,0.00000563,0.00000499,-0.00002353,-
0.00001072,-0.00002386,-0.00001918,-0.00000941,-
0.00000025,0.00000813,0.00000581,0.00000506,-0.00001797,0.00001799,-
0.00000371,0.00000751,-0.00000076,0.03293928,0.04572501,-
0.03687189,0.02532594,-0.04792325,0.01149964,-0.06383179,-
0.00034055,0.03444383,0.02397742,-0.25981292,0.09801760,-
0.08232324,0.56392735,-
0.00068988,0.00066039,0.00020676,0.00395250,0.00417777,0.00234528,0.001114
66,-0.00117339,-0.00013587,0.00022229,0.00067614,0.00015713,-0.00038642,-
0.00017888,0.00000955,0.00049442,-
0.00022327,0.00005402,0.00000365,0.00003767,-0.00007983,0.00000445,-
0.00001960,-0.00009041,0.00007838,-0.00010234,-0.00002461,0.00001212,-
0.00001458,0.00000111,-0.00000780,0.00000115,-0.00000056,0.00000440,-
0.00000195,-0.00000230,0.00000707,0.00000254,-
0.00001683,0.00002263,0.00002636,0.00002399,0.00000503,-0.00000440,-
0.00000274,0.00000051,0.00000147,-0.00000331,0.00000097,0.00000158,-
0.00000154,0.00000307,0.00000152,0.00000013,0.00000932,0.00000576,0.000034
21,-0.00002266,0.00001874,-0.00000467,-0.00000703,-
0.00001379,0.00000156,0.00001400,0.00000190,0.,-0.00000300,0.00000997,-
0.00000141,0.00000639,-0.00001041,-0.00000167,-
0.00000261,0.00000141,0.00000399,-
0.00000040,0.00000327,0.00000051,0.00000404,-
0.00000095,0.00000682,0.00000015,-0.00000129,-0.00000029,0.00682892,-
0.02106711,0.01335857,-0.01002422,0.01280839,-

0.00866565,0.02724589,0.01798860,-0.01406269,0.01145874,0.11944703,-
0.13427242,-0.19697294,-0.20264323,0.34124415,-0.00131362,-
0.00385410,0.00218713,-
0.03252864,0.00354889,0.01379692,0.00339207,0.00330910,0.00064487,-
0.00161935,0.00109600,-0.00098797,-0.00015190,-0.00061532,-0.00007181,-
0.00060972,-0.00030494,-0.00071334,-0.00027940,-0.00043610,0.00078128,-
0.00005359,0.00000491,0.00020917,0.00025662,0.00039782,-0.00004867,-
0.00016100,-0.00008277,0.00002802,0.00008629,0.00000380,-0.00001148,-
0.00023924,-0.00011087,-0.00001863,0.00007530,0.00006269,0.00002562,-
0.00023498,-0.00010178,0.00001466,0.00003674,-0.00001083,-
0.00002833,0.00001487,0.00001744,0.00000525,-0.00001393,-0.00001098,-
0.00000793,-0.00001550,-0.00000156,0.00000060,-0.00007168,-0.00035011,-
0.00004414,0.00001990,0.00018969,0.00000355,0.00001072,0.00018012,-
0.00000555,-0.00002288,-0.00006736,-
0.00000009,0.00002584,0.00023918,0.00000631,0.00002155,-
0.00008146,0.00000529,-0.00002682,-0.00001815,-0.00001686,-
0.00000035,0.00002187,0.00001071,0.00002420,-0.00003250,0.00003271,-
0.00000511,0.00001341,-0.00000323,-0.23272451,-
0.00920570,0.08284981,0.02874503,-0.06189126,0.02591555,-
0.05678586,0.00997780,0.01783438,0.02477086,0.05684752,-0.04038960,-
0.25953792,0.09091363,0.04220949,0.59426492,0.00272227,-0.00177621,-
0.00023407,0.01648434,0.01182948,-0.01091024,0.00216202,-
0.00358321,0.00011544,0.00083705,0.00037147,0.00074054,0.00082132,0.000024
57,0.00002844,-0.00062351,0.00043386,0.00073024,0.00000968,0.00023843,-
0.00024404,0.00001527,-0.00001875,-0.00015844,-0.00006149,-0.00042500,-
0.00006016,0.00011153,0.00004869,-0.00000267,-0.00006475,-
0.00001039,0.00001448,0.00017903,0.00007906,0.00000650,-0.00003634,-
0.00004366,-0.00004078,0.00018430,0.00011242,-0.00000071,-
0.00003541,0.00000361,0.00002667,-0.00003284,-0.00002096,-
0.00003268,0.00001397,0.00001111,0.00000691,0.00001705,0.00000259,0.000001
53,0.00006229,0.00023365,0.00000232,-0.00007207,-0.00009002,-0.00001954,-
0.00001541,-0.00012587,0.00003149,0.00000698,0.00003570,0.00002096,-
0.00002161,-0.00016202,-0.00001116,-
0.00001607,0.00001807,0.00002659,0.00005070,0.00003030,-
0.00001053,0.00000081,-0.00001130,-0.00001354,0.00000506,0.00002931,-
0.00004367,0.00000962,-0.00001330,-0.00000339,-0.05576309,-
0.12953417,0.05935097,-0.02868458,0.00133197,0.01787251,0.01258517,-
0.00510334,-0.00669756,0.01809095,-0.03523889,0.01569559,0.14557832,-
0.19534549,0.00986919,-0.10788093,0.61238869,-0.00010059,-0.00006882,-
0.00072708,0.00413302,-0.00301905,0.00377306,-
0.00280457,0.00058608,0.00101950,0.00012305,-0.00042713,-
0.00017882,0.00026163,0.00031965,0.00016967,-0.00016351,0.00073295,-
0.00007588,0.00018673,0.00024301,-0.00008339,0.00005331,0.00004995,-
0.00001482,-0.00009283,0.00002894,-0.00006585,0.00004542,-0.00000261,-
0.00001312,-
0.00001323,0.00000408,0.00000394,0.00003460,0.00002042,0.00000446,-
0.00001601,-0.00001931,0.00001781,0.00003420,0.00000974,-0.00000697,-
0.00000677,0.00000907,0.00000691,-0.00000133,0.00000562,-
0.00000004,0.00000474,0.00000228,0.00000010,-0.00000112,0.00000174,-
0.00000207,-0.00008468,-0.00002351,-0.00008667,0.00001324,-0.00000862,-
0.00004545,-0.00001158,0.00002688,-0.00000980,-0.00000472,-

0.00000418,0.00000227,0.00000696,-
0.00005075,0.00000894,0.00001335,0.00004039,-0.00000257,-
0.00000807,0.00000209,0.00005871,-
0.00000304,0.00000743,0.00000511,0.00000582,0.00000196,0.00000709,0.000002
22,-0.00000059,0.00000250,0.11179918,0.03757169,-
0.13589205,0.00727155,0.03312047,-0.01381583,0.01605904,-0.00559220,-
0.01062662,-0.01862485,-0.00093942,0.01726595,0.01169060,0.03445647,-
0.08446347,-0.15462747,-0.22822823,0.33422397,0.00002041,0.00012437,-
0.00002919,0.00113307,-0.00173642,-0.00094437,-0.00057918,0.00002100,-
0.00003804,0.00018023,-0.00037555,0.00004766,0.00030215,0.00030266,-
0.00001342,-0.00050242,0.00032629,-
0.00017661,0.00006232,0.00004095,0.00002433,0.00002858,0.00000828,0.000046
23,-0.00008457,-0.00003212,-0.00001534,0.00003784,0.00002306,-0.00000040,-
0.00002198,-0.00000274,-0.00000195,0.00006557,0.00002842,0.00000396,-
0.00001529,-0.00001860,0.00000321,0.00004519,0.00002591,-0.00000477,-
0.00001094,0.00000239,0.00000504,-0.00000556,-0.00000985,-
0.00000024,0.00000314,0.00000165,0.00000365,0.00000371,-0.00000014,-
0.00000011,0.00001208,0.00007335,0.00005284,0.00001835,-
0.00006240,0.00000192,-0.00001959,-0.00003751,-
0.00000815,0.00000435,0.00001326,0.,-0.00000329,-0.00007993,0.00000286,-
0.00000916,0.00003431,-
0.00000043,0.00001364,0.00001423,0.00000421,0.00000089,-0.00001033,-
0.00000566,-0.00000819,0.00000865,-0.00000665,0.00000189,-
0.00000458,0.,0.00287767,-0.02254516,0.01301657,-
0.00351959,0.00047368,0.00424201,-0.00114117,0.00015982,0.00026780,-
0.00277195,0.00359587,0.00283140,0.00413768,0.02303329,-0.01426925,-
0.05650441,0.00078439,0.00860559,0.05567636,0.00067903,-0.00022234,-
0.00080803,0.00076083,-0.00127613,-0.00160520,-0.00080886,0.00022509,-
0.00004772,0.00011651,-
0.00034916,0.00002999,0.00017842,0.00018679,0.00003752,-
0.00020121,0.00021339,-0.00005937,0.00003840,0.00001289,-
0.00001285,0.00000737,0.00001418,0.00003990,-
0.00003581,0.00001490,0.00002266,-0.00000926,0.00001461,-
0.00000506,0.00000287,-0.00000026,-
0.00000376,0.00000467,0.00000539,0.00000339,-0.00000733,-
0.00000558,0.00000274,0.00000390,-0.00000263,-0.00000812,-0.00000037,-
0.00000062,-0.00000037,0.00000135,-0.00000524,0.00000899,-0.00000086,-
0.00000198,0.00000201,-0.00000029,-0.00000100,0.00000017,-
0.00000967,0.00001535,-0.00004686,0.00000568,-
0.00001594,0.00000568,0.00001113,0.00001162,-0.00000248,-0.00000714,-
0.00000032,-0.00000054,0.00000118,-0.00000166,-0.00000081,-0.00000832,-
0.00000267,0.00000222,0.00000039,-0.00000078,-0.00001220,-0.00000025,-
0.00000225,-0.00000041,-0.00000256,-0.00000138,-0.00000045,-
0.00000089,0.00000128,0.00000113,0.00021039,-0.00407590,0.00381665,-
0.00045543,0.00211261,0.00225495,-0.00004636,0.00049265,-
0.00035191,0.00479633,0.00136739,0.00136992,-0.00189083,-
0.01316637,0.00975017,0.00003964,-0.25912140,0.13309502,-
0.00320450,0.27504931,0.00110287,0.00045173,0.00011440,-0.00138814,-
0.00145897,-0.00182154,-0.00034190,0.00012619,0.00017393,-0.00006092,-
0.00020563,0.00008611,0.00003457,0.00012612,-0.00005193,-0.00041629,-
0.00038894,0.00008738,-0.00008850,-0.00003364,-0.00006161,-0.00005628,-

0.00001814,0.00001152,0.00005411,0.00002424,-0.00002414,-0.00005617,-
0.00000669,0.00000825,0.00001886,-0.00000611,0.00000537,-0.00005423,-
0.00002472,-0.00000447,0.00001572,0.00000717,-0.00000074,-0.00003231,-
0.00001951,0.00000289,0.00000477,0.,-0.00000741,0.00000327,0.00000636,-
0.00000370,-0.00000596,-0.00000293,-0.00000236,-
0.00000220,0.00000014,0.00000032,0.00004451,-0.00002452,-
0.00001362,0.00001637,-
0.00000282,0.00005954,0.00002567,0.00001973,0.00000250,-
0.00001463,0.00000130,-0.00000419,0.00000076,0.00006189,-0.00000703,-
0.00002095,-0.00003080,0.00000291,0.00000975,-0.00000914,-
0.00006829,0.00000254,-0.00000659,-0.00000167,-0.00000821,-0.00000403,-
0.00000484,-0.00000395,0.00000506,-0.00000070,-0.00179423,0.01475285,-
0.00492039,0.00463733,0.00171053,0.00272411,0.00009560,-0.00041623,-
0.00018767,0.00238962,0.00195580,0.00520105,0.00061871,-
0.00246856,0.00360803,0.00952147,0.13371240,-0.11959893,-0.01271989,-
0.14549511,0.11809804,-0.00007426,-0.00003696,-0.00007376,-0.00046335,-
0.00082702,0.00056349,-0.00015135,0.00030548,-0.00000961,-0.00004491,-
0.00008150,-0.00004223,-
0.00003280,0.00002203,0.00000361,0.00002588,0.00001669,0.00000457,0.000005
71,-0.00002908,0.00001130,-0.00000345,0.00000585,-
0.00000241,0.00001615,0.00004514,0.00000708,-0.00002111,-0.00000377,-
0.00000095,0.00001034,0.00000158,-0.00000144,-0.00002528,-0.00001080,-
0.00000061,0.00000485,0.00000544,0.00000365,-0.00002161,-0.00001364,-
0.00000296,0.00000430,-0.00000069,-
0.00000281,0.00000410,0.00000293,0.00000638,-0.00000244,-0.00000216,-
0.00000027,-0.00000200,-0.00000061,0.00000031,-0.00001995,-0.00003867,-
0.00000167,0.00000610,0.00002025,-0.00000168,0.00000650,0.00002335,-
0.00000216,-0.00000259,-0.00000670,-
0.00000245,0.00000295,0.00002816,0.00000053,0.00000330,-0.00000742,-
0.00000182,-0.00000704,-0.00000448,0.00000130,-
0.00000063,0.00000301,0.00000257,-0.00000051,-0.00000424,0.00000530,-
0.00000150,0.00000277,0.00000063,-
0.00098887,0.00477663,0.00137959,0.00011570,0.00062700,-
0.00051846,0.00210619,0.00140193,0.00098546,0.00363279,-0.00007233,-
0.00027136,-0.24374507,-0.05871090,0.12930028,-0.01488789,-
0.00780320,0.01318781,0.00027792,-0.00078174,-
0.00160912,0.25625869,0.00001362,0.00000299,0.00002122,0.00053009,0.000546
05,-0.00049085,0.00017168,-0.00020314,-
0.00005974,0.00005502,0.00002278,0.00006769,0.00000402,-0.00000869,-
0.00000094,0.00000871,-0.00001639,0.00002432,0.00000464,-0.00000081,-
0.00002255,0.00000124,-0.00000147,-0.00000959,0.00000601,-
0.00002245,0.00000434,-0.00000028,0.00000256,-0.00000160,-
0.00000023,0.00000042,0.00000060,0.00000154,0.00000061,-0.00000025,-
0.00000032,-0.00000094,-0.00000241,0.00000490,0.00000484,-0.00000043,-
0.00000051,-0.00000102,-0.00000005,-0.00000025,-
0.00000064,0.00000092,0.00000023,0.,0.00000039,0.00000097,0.00000018,0.0000
0034,0.00001222,0.00002314,0.00000225,-0.00000435,-0.00001462,0.00000692,-
0.00000246,-0.00000834,0.00000221,-0.00000045,0.00000128,0.00000026,-
0.00000110,-0.00000841,-0.00000007,-
0.00000373,0.00000313,0.00000194,0.00000595,0.00000401,-
0.00000607,0.00000169,-0.00000462,-0.00000347,-0.00000066,0.00000162,-

0.00000347,0.00000041,-0.00000088,-0.00000103,0.00422016,-
0.00067190,0.00272359,0.00037724,-0.00068070,-0.00000854,0.00231911,-
0.00375921,0.00429383,-0.02356813,-0.00278410,0.01422592,-0.05704963,-
0.07545124,0.04801374,0.01678706,0.00914146,-0.01117160,-0.00117861,-
0.00013106,-0.00172690,0.05982672,0.07412685,0.00003947,-0.00005210,-
0.00008047,-0.00010671,-0.00001956,0.00009350,0.00002565,-0.00000053,-
0.00009948,0.00000755,-0.00005549,0.00001862,0.00000622,-
0.00000721,0.00000405,0.00002618,0.00000674,0.00003528,-0.00000613,-
0.00002674,-0.00002060,0.00000330,-0.00000016,-0.00001459,-0.00000947,-
0.00000324,0.00001014,-0.00000052,0.00000459,0.00000278,-0.00000187,-
0.00000104,-0.00000025,0.00000833,0.00000374,0.00000047,-0.00000290,-
0.00000171,-0.00000042,0.00000376,0.00000051,-0.00000219,-0.00000080,-
0.00000010,0.00000069,0.00000084,-0.00000069,-0.00000095,-0.00000108,-
0.00000001,0.00000025,0.00000052,-0.00000022,-
0.00000024,0.00000747,0.00002411,0.00000422,0.00000134,-
0.00002014,0.00000446,-0.00000182,-0.00000964,-
0.00000082,0.00000046,0.00000421,-0.00000130,-0.00000179,-0.00001600,-
0.00000039,-0.00000168,0.00000854,0.00000059,0.00000513,0.00000438,-
0.00000084,0.00000114,-0.00000464,-0.00000147,-0.00000351,0.00000224,-
0.00000234,-0.00000028,-
0.00000037,0.00000017,0.00143215,0.00266260,0.00556402,-
0.00054162,0.00005602,-
0.00009309,0.00077187,0.00454835,0.00286651,0.01267739,0.00276006,-
0.00455830,0.12838190,0.04926038,-0.12582223,-
0.00062287,0.00056655,0.00347845,-0.00140736,-0.00201995,-0.00236465,-
0.14139655,-0.05503349,0.12339894,0.00037444,-0.00054910,-0.00006036,-
0.00095548,0.00017131,-0.00129379,-0.00044656,0.00064325,-
0.00074181,0.00011476,-0.00004263,-
0.00001164,0.00007685,0.00011193,0.00003876,-0.00024561,-
0.00000739,0.00003071,-0.00069942,0.00090768,-0.00066626,0.00001219,-
0.00000077,0.00002531,-0.00000589,-0.00001099,-
0.00005989,0.00000220,0.00000145,0.00000758,-0.00001140,-
0.00000995,0.00000233,0.00002089,0.00000876,-0.00000153,-0.00000067,-
0.00000925,-0.00000110,0.00001541,0.00002227,0.00000049,-0.00000342,-
0.00000173,0.00000132,-0.00000532,-0.00000244,-
0.00001551,0.00000040,0.00000006,0.00000109,0.00000273,-
0.00000051,0.00000116,0.00002640,-0.00000282,0.00002290,0.00001274,-
0.00001869,-0.00000532,-0.00000734,-0.00001257,-0.00000627,0.00000053,-
0.00000175,0.00000134,0.00000135,-0.00001613,0.00000224,-
0.00000252,0.00000918,-0.00000255,-0.00000234,0.00000189,0.00000886,-
0.00000146,-0.00000254,0.00000107,-0.00000214,0.00000244,-0.00000191,-
0.00000037,-0.00000178,0.00000029,0.00286463,0.00012034,-0.00015057,-
0.23349068,-0.05477887,0.12911989,-0.01688939,-0.00830334,0.01388537,-
0.00098771,0.00530937,0.00105926,0.00029807,0.00040663,-
0.00040904,0.00180957,0.00118888,0.00110859,0.00018568,0.00012015,0.000030
73,-0.00021786,-0.00024650,-0.00030786,0.24863665,0.00027101,-0.00071605,-
0.00009404,-0.00226866,0.00081226,-0.00112520,-0.00008253,0.00018479,-
0.00027002,0.00050232,-0.00039335,-
0.00022452,0.00023269,0.00041355,0.00000242,-
0.00048453,0.00007819,0.00007513,0.00045228,0.00007601,0.00070985,0.000005
84,0.00003206,0.00005569,0.00007172,0.00002732,0.00003723,-0.00006711,-

0.00000022,0.00000348,0.00003542,-0.00000044,-0.00000025,-0.00007497,-
0.00002686,0.00000212,0.00001004,0.00000584,0.00000029,-0.00002671,-
0.00002533,-0.00000859,0.00000979,-0.00000542,-
0.00000701,0.00001030,0.00001569,0.00000285,-0.00001008,-0.00000324,-
0.00000649,-0.00000353,-0.00000001,-0.00000020,-0.00000187,-0.00009673,-
0.00001007,0.00001505,0.00004063,-0.00000186,0.00001086,0.00005206,-
0.00000636,-0.00001380,-
0.00002288,0.00000428,0.00000865,0.00007212,0.00000378,0.00000354,-
0.00002012,-0.00000288,-0.00000919,-0.00000805,0.00000234,-
0.00000041,0.00000282,0.00000346,0.00000521,-0.00000992,0.00000431,-
0.00000219,0.00000377,-0.00000057,-0.02254587,-0.00382802,0.01453312,-
0.05405361,-0.07416726,0.04805702,0.01651898,0.00888277,-
0.01166012,0.00414286,-0.00044124,0.00272825,0.00061347,-
0.00097749,0.00012289,0.00212176,-0.00339951,0.00440278,-0.00125702,-
0.00017628,0.00091513,-0.00025529,-0.00014366,-
0.00048030,0.05635283,0.07335398,0.00021138,-0.00029700,0.00015563,-
0.00115271,-0.00161048,-0.00179172,0.00018275,0.00113266,0.00017289,-
0.00024866,-0.00051737,0.00007476,0.00011539,0.00007497,-0.00005652,-
0.00018054,-0.00011284,0.00009040,-0.00105194,-0.00002597,-0.00052553,-
0.00000225,-0.00005830,0.00001053,-0.00003355,0.00003627,-0.00000752,-
0.00000473,0.00001471,0.00006046,-0.00002418,-
0.00002935,0.00000236,0.00005506,0.00001782,-0.00000658,0.00000628,-
0.00001268,-0.00000522,0.00000992,0.00002861,0.00000303,-0.00000147,-
0.00000904,-0.00000500,-0.00001465,0.00001029,-0.00006652,-
0.00000688,0.00000175,-0.00000100,0.00000483,-
0.00000135,0.00000002,0.00000651,0.00005713,-0.00002423,0.00000923,-
0.00005404,0.00000663,-0.00000887,-
0.00003507,0.00000268,0.00000195,0.00001705,-0.00000073,-0.00000712,-
0.00005617,-0.00000327,-
0.00001088,0.00001533,0.00000548,0.00000488,0.00000465,-0.00000376,-
0.00000034,-0.00000635,-0.00000172,-0.00000144,0.00000447,-
0.00000697,0.00000124,-0.00000229,0.00000056,0.01354182,0.00224646,-
0.00496075,0.12949387,0.04919901,-0.12796719,-
0.00098010,0.00044029,0.00277920,0.00166763,0.00243119,0.00533281,-
0.00040039,-0.00005875,-
0.00012599,0.00050277,0.00466791,0.00301925,0.00081953,0.00026663,-
0.00001157,-0.00032294,-0.00044768,-0.00076270,-0.14071106,-
0.05554405,0.12689701,-0.00032234,0.00012112,-
0.00004228,0.00049936,0.00081107,-
0.00060655,0.00000181,0.00004774,0.00003369,-0.00001902,-
0.00000297,0.00002789,-0.00002052,0.00001140,-
0.00000073,0.00002232,0.00006806,0.00007481,-0.00003229,-0.00000023,-
0.00004711,-0.00000410,0.00000170,-
0.00000907,0.00003018,0.00002760,0.00000263,-0.00001858,-
0.00001229,0.00000784,0.00000685,-0.00000334,0.00000250,-0.00001714,-
0.00000761,-0.00000026,0.00000363,0.00000145,0.00000084,-0.00001421,-
0.00000884,0.00000332,0.00000293,0.00000035,-
0.00000550,0.00000297,0.00000975,-0.00000629,-0.00000596,0.00000029,-
0.00000440,-0.00000190,0.00000049,-0.00000107,-0.00003268,-
0.00000987,0.00000416,0.00000184,0.00000682,-
0.00000060,0.00000491,0.00001332,0.00000050,-0.00000202,-0.00000265,-

0.00000319,0.00000093,0.00001056,-0.00000016,0.00000085,-
0.00000335,0.00000105,-0.00000100,-0.00000165,-
0.00000145,0.00000044,0.00000092,0.00000031,-0.00000005,-
0.00000207,0.00000081,-0.00000020,0.00000165,0.00000004,-
0.00276362,0.00363620,0.00276173,0.00439248,0.02249786,-0.01335895,-
0.05666858,0.00334606,0.00629101,0.00480407,-0.02313966,0.01274589,-
0.00397965,0.00056889,0.00405090,-0.00093015,0.00004400,0.00018272,-
0.00005161,-0.00017189,-0.00035718,0.00027068,-
0.00127251,0.00088845,0.00028497,-0.00121574,-
0.00140692,0.05413215,0.00023397,-0.00001884,-0.00003011,-0.00048792,-
0.00045585,0.00036827,-0.00002371,-0.00007049,-
0.00006338,0.00000724,0.00003097,0.00001282,0.00001878,-
0.00002078,0.00000140,-0.00005833,-0.00004608,-0.00001629,-
0.00001402,0.00003874,0.00001238,0.00000426,-0.00000026,-0.00000544,-
0.00001931,-0.00001953,-0.00000004,0.00001081,0.00000698,-0.00000138,-
0.00000463,0.00000099,-0.00000108,0.00001598,0.00000688,0.00000067,-
0.00000360,-0.00000275,-0.00000192,0.00001349,0.00000885,-0.00000160,-
0.00000226,-0.00000172,0.00000364,-0.00000112,-
0.00000502,0.00000081,0.00000162,0.00000027,0.00000142,0.00000202,-
0.00000028,0.00000058,0.00002799,0.00001977,0.00000598,0.00000336,-
0.00001648,-0.00000065,-0.00000519,-0.00001745,-
0.00000322,0.00000269,0.00000421,0.00000247,-0.00000182,-0.00001861,-
0.00000043,-0.00000146,0.00000740,-
0.00000118,0.00000117,0.00000331,0.00000516,-0.00000062,-
0.00000246,0.00000033,-0.00000164,0.00000286,-0.00000192,0.00000006,-
0.00000162,0.00000038,0.00405704,0.00132868,0.00177323,-0.00158427,-
0.01040818,0.00881162,0.00175359,-0.27121022,0.13100070,-0.00046985,-
0.00378079,0.00467662,-
0.00038535,0.00225135,0.00209090,0.00029136,0.00035328,-0.00052080,-
0.00018366,-0.00029834,-0.00037840,0.00021185,-0.00001875,0.00030059,-
0.00082419,-0.00016331,-0.00191022,-
0.00409920,0.28438216,0.00002831,0.00001977,-0.00010324,-0.00005016,-
0.00019321,0.00011887,-0.00006654,0.00001633,-0.00008075,-
0.00000034,0.00002780,0.00003669,-0.00000311,-0.00000111,0.00000360,-
0.00005837,-0.00001434,0.00002010,-0.00002561,0.00005386,-0.00000143,-
0.00000058,0.00000301,-0.00001451,0.00001433,0.00000815,0.00000334,-
0.00001524,-0.00000188,0.00000521,0.00000636,0.00000058,0.00000012,-
0.00000848,-0.00000379,-0.00000039,0.00000224,0.00000046,-0.00000157,-
0.00000442,-0.00000210,-0.00000100,0.00000210,-0.00000267,-
0.00000158,0.00000211,0.00000450,-0.00000097,-0.00000401,-0.00000033,-
0.00000107,0.00000039,-0.00000045,0.00000014,0.00000521,-
0.00000489,0.00001077,0.00000411,-0.00000285,0.00000153,-0.00000157,-
0.00000059,-0.00000249,-0.00000005,-0.00000187,-
0.00000008,0.00000050,0.00000321,0.00000039,-0.00000040,0.00000016,-
0.00000026,-0.00000048,0.00000133,0.00000036,-0.00000004,-
0.00000137,0.00000011,-0.00000030,-0.00000009,0.00000022,-
0.00000046,0.00000006,-
0.00000018,0.00277750,0.00178925,0.00548957,0.00056068,-
0.00219933,0.00458942,0.00761291,0.13041791,-0.11693875,-
0.00050086,0.01471006,-
0.00413350,0.00434311,0.00190360,0.00247357,0.00025077,-0.00051160,-

0.00018981,-0.00038897,-0.00037478,-
0.00070120,0.00007946,0.00096501,0.00013273,-0.00173159,-0.00176855,-
0.00242346,-0.00994022,-0.14424251,0.11395663,0.00126416,-0.00003630,-
0.00052652,-0.00077525,0.00022765,-0.00027447,0.00010490,-
0.00099764,0.00044012,-0.00020261,-0.00041463,-0.00032348,0.00074487,-
0.00043735,-0.00000268,0.00010645,0.00034801,0.00032146,-
0.00028704,0.00000362,0.00003557,0.00011150,-0.00007745,-0.00000231,-
0.00060114,-0.00042437,-0.00008085,0.00032189,0.00016902,0.00012379,-
0.00020494,-0.00006639,-0.00000982,0.00063148,0.00027799,0.00003055,-
0.00014011,-0.00012908,-0.00003216,0.00040904,0.00017400,-0.00001126,-
0.00009312,0.00002303,0.00006716,-0.00006065,-0.00007031,-
0.00012367,0.00000693,0.00003043,0.00001322,0.00002643,0.00000123,-
0.00001154,0.00025850,0.00075693,0.00007363,-0.00008871,-0.00039043,-
0.00010891,-0.00006400,-
0.00047759,0.00001456,0.00008126,0.00019038,0.00003386,-0.00008137,-
0.00073762,-0.00002450,-
0.00000687,0.00022612,0.00002515,0.00008317,0.00008874,0.00010791,-
0.00000710,-0.00002418,-0.00001939,-0.00004792,0.00008953,-
0.00007712,0.00002115,-0.00003531,0.00001274,-0.00282966,-
0.00011646,0.00092005,0.00013437,-0.00427334,0.00548704,-
0.03278964,0.01597855,0.00650816,-
0.16369208,0.05610673,0.00874326,0.00599868,0.01276730,-
0.01031766,0.00728836,-0.00346692,0.00227589,0.00107399,-
0.00099088,0.00046932,-0.00065080,-
0.00397732,0.00067555,0.00009798,0.00056866,-0.00012442,-
0.00043060,0.00019779,-0.00171061,0.55252953,-0.00054027,-
0.00032272,0.00034691,-0.00017702,-0.00041259,-0.00030068,-
0.00023012,0.00092365,-0.00037621,0.00021364,0.00010669,0.00022347,-
0.00035899,0.00042937,0.00003746,-0.00036268,-0.00017365,-
0.00021643,0.00019988,-0.00000026,-0.00002412,-
0.00006692,0.00006140,0.00000118,0.00045799,0.00031920,0.00004568,-
0.00025871,-0.00014157,-0.00006341,0.00016090,0.00004069,0.00002109,-
0.00046368,-0.00020420,-0.00002212,0.00010010,0.00008422,0.00002640,-
0.00028954,-0.00013506,0.00001712,0.00006466,-0.00001161,-
0.00005989,0.00004772,0.00007566,0.00006543,-0.00002062,-0.00001478,-
0.00002379,-0.00002040,0.00000346,0.00000186,-0.00015457,-0.00044400,-
0.00006526,0.00004297,0.00021184,0.00008643,0.00002982,0.00029843,-
0.00000067,-0.00005754,-0.00011933,-
0.00001676,0.00004881,0.00043899,0.00001597,-0.00000383,-0.00012672,-
0.00000194,-0.00003001,-0.00004416,-0.00008713,0.00001394,0.00000427,-
0.00000171,0.00003493,-0.00005576,0.00003700,-0.00000864,0.00002132,-
0.00001365,0.00047499,-0.00351597,0.00117894,-
0.00598992,0.00543468,0.00390975,0.00956455,0.01164514,-
0.01037095,0.04635935,-0.12834358,0.00783911,0.02269618,-
0.02165956,0.00101329,-0.00281427,-
0.00145093,0.00629238,0.00080657,0.00004697,-0.00003399,-0.00090577,-
0.00064371,-0.00143353,-0.00119602,0.00093623,0.00045846,-0.00416585,-
0.00218368,0.00094083,-0.09439519,0.82832704,0.00003441,-
0.00002328,0.00003101,0.00010923,-0.00031117,-0.00082338,-
0.00023668,0.00017778,0.00003102,0.00009162,-0.00020321,-
0.00001289,0.00005321,0.00020626,-0.00001942,-0.00020179,-0.00002420,-

0.00004346,-0.00002932,-0.00003477,0.00004793,-
0.00001263,0.00000107,0.00004258,0.00002563,0.00002372,-0.00000767,-
0.00003522,0.00000763,-0.00001105,0.00001470,0.00000525,-0.00000437,-
0.00003878,-0.00001619,-0.00000204,0.00000988,0.00000458,-0.00000076,-
0.00002227,-0.00000241,-0.00000580,0.00000606,-0.00000622,-
0.00000158,0.00000428,-0.00000388,0.00001633,0.00000130,-
0.00000698,0.00000708,0.00000065,-0.00000267,0.00000435,-0.00002492,-
0.00008575,-
0.00001876,0.00003088,0.00002832,0.00001137,0.00001401,0.00005471,-
0.00000821,-0.00001283,-0.00002018,-
0.00000695,0.00000969,0.00008211,0.00000215,0.00000017,-0.00002186,-
0.00000737,-0.00001614,-0.00001056,-0.00000441,-
0.00000344,0.00000094,0.00000975,-0.00000026,-0.00001050,0.00001030,-
0.00000574,0.00000527,0.00000177,0.00062895,0.00156213,-
0.00217812,0.00663365,0.00259733,0.00253822,0.00994556,-
0.01307119,0.00392169,0.01418694,0.00342673,-0.08485851,-
0.01565941,0.00549219,0.00321073,0.00148339,0.00669904,0.00332439,-
0.00060274,0.00090258,0.00044147,-0.00107769,-0.00006912,-
0.00348047,0.00097754,-0.00038882,0.00037330,0.00076857,-0.00109317,-
0.00359469,-0.10771730,-0.30464049,0.41920615,-
0.00041437,0.00013890,0.00014901,0.00050517,-0.00018298,-0.00003078,-
0.00014072,0.00026628,-0.00009769,0.00009076,0.00009180,0.00006452,-
0.00024493,0.00015416,-0.00000030,0.00000198,-0.00012077,-
0.00006638,0.00011433,0.00000442,0.00001814,-
0.00004093,0.00002819,0.00000372,0.00019742,0.00013867,0.00002338,-
0.00010514,-0.00005658,-0.00004937,0.00007107,0.00002475,0.00000356,-
0.00022182,-0.00009693,-0.00001036,0.00004858,0.00004599,0.00001214,-
0.00013909,-0.00006046,0.00000260,0.00003116,-0.00000658,-
0.00002094,0.00001978,0.00002153,0.00004604,0.00000117,-0.00001051,-
0.00000368,-0.00000894,-0.00000016,0.00000431,-0.00008703,-0.00025514,-
0.00002351,0.00002791,0.00013521,0.00004164,0.00002263,0.00016365,-
0.00000330,-0.00002932,-0.00006681,-
0.00001115,0.00002829,0.00026011,0.00000838,0.00000085,-0.00008090,-
0.00000811,-0.00002670,-0.00003055,-
0.00004128,0.00000345,0.00000706,0.00000505,0.00001690,-
0.00003057,0.00002435,-0.00000704,0.00001166,-
0.00000529,0.00060986,0.00083176,-0.00071694,0.00132944,0.00030137,-
0.00110929,-0.00208313,0.00152898,0.00025261,0.00718183,0.03304273,-
0.01304097,-0.00510246,-0.00008383,0.00378672,-0.00121556,0.00196970,-
0.00121787,-0.00055975,0.00038951,-0.00016284,0.00020577,0.00081261,-
0.00025339,-0.00012073,-0.00005698,0.00010075,-0.00002631,0.00168762,-
0.00105760,-0.11358900,0.03272870,-
0.00038813,0.09872336,0.00039944,0.00007413,-
0.00026953,0.00004187,0.00024394,-0.00005447,0.00007111,-
0.00056401,0.00021129,-0.00008843,-0.00013346,-0.00012043,0.00028773,-
0.00021588,-0.00001982,0.00015183,0.00015752,0.00013954,-
0.00011432,0.00001279,0.00001313,0.00004973,-0.00003816,-0.00000016,-
0.00031835,-0.00022008,-0.00003418,0.00017939,0.00010036,0.00004039,-
0.00011191,-0.00002762,-0.00001544,0.00032110,0.00014209,0.00001584,-
0.00006982,-0.00005957,-0.00001701,0.00020020,0.00009428,-0.00001394,-
0.00004448,0.00000841,0.00004122,-0.00003314,-0.00005379,-

0.00004194,0.00001529,0.00000951,0.00001762,0.00001394,-0.00000279,-
0.00000111,0.00009984,0.00028732,0.00003956,-0.00002426,-0.00014040,-
0.00006339,-0.00002084,-0.00019664,-
0.00000281,0.00003828,0.00007944,0.00001166,-0.00003169,-0.00029626,-
0.00000981,0.00000375,0.00008694,0.00000075,0.00001859,0.00002850,0.000063
14,-0.00001020,-0.00000144,0.00000249,-0.00002312,0.00003699,-
0.00002296,0.00000554,-0.00001430,0.00000992,-
0.00159717,0.00052158,0.00047864,0.00124593,-0.00143531,-0.00019636,-
0.00694665,-0.00210215,0.00443753,0.02110888,-
0.04145744,0.02380705,0.00510373,0.00380102,-0.00275259,0.00233129,-
0.00201925,-0.00065769,0.00046502,-0.00058339,-0.00006768,-
0.00019345,0.00077662,-0.00003492,0.00038211,-0.00012851,-
0.00012932,0.00189006,0.00019427,-0.00116617,0.03267630,-
0.58117332,0.26591714,-0.02064002,0.65897732,-0.00009260,-
0.00009376,0.00011051,-
0.00025915,0.00003465,0.00020588,0.00010049,0.00014179,-0.00007448,-
0.00001486,0.00010225,0.00004454,-0.00008757,0.00000702,0.00001026,-
0.00001489,-0.00004529,-0.00003970,0.00002258,-0.00000691,-0.00003034,-
0.00000963,0.00000761,-0.00001147,0.00007001,0.00005009,0.00000964,-
0.00002406,-0.00002982,-0.00000922,0.00001899,0.00000295,0.00000489,-
0.00006706,-0.00002983,-0.00000312,0.00001480,0.00001465,0.00000654,-
0.00004685,-0.00002523,0.00000763,0.00000909,0.00000279,-
0.00001072,0.00000465,0.00001349,0.00000450,-0.00000145,0.00000015,-
0.00000686,-0.00000521,0.00000198,-0.00000155,-0.00001904,-0.00006238,-
0.00000779,-0.00000787,0.00004091,0.00001039,-
0.00000019,0.00003724,0.00000341,-0.00000547,-
0.00001569,0.00000084,0.00000631,0.00005663,0.00000231,-0.00000065,-
0.00002042,0.00000317,0.00000032,-0.00000663,-
0.00001728,0.00000377,0.00000348,-0.00000426,0.00000919,-
0.00000740,0.00000408,0.00000089,0.00000181,-0.00000329,0.00066601,-
0.00064623,0.00018487,-
0.00158435,0.00037905,0.00004716,0.00493650,0.00054826,-0.00244403,-
0.00641530,0.01836289,0.01158873,0.00085281,-0.00036174,0.00280673,-
0.00135848,-0.00060418,-0.00063079,-0.00017429,-0.00009001,-
0.00023077,0.00031512,-0.00043871,0.00077550,-
0.00015983,0.00009911,0.00003764,-0.00122843,-0.00106148,0.00080787,-
0.00029942,0.26586688,-0.22187077,-0.01882988,-0.33792917,0.22823043,-
0.00055412,0.00002174,0.00019816,0.00045609,-0.00019087,-0.00005103,-
0.00018934,0.00042977,-0.00021024,0.00014694,0.00013198,0.00012362,-
0.00031102,0.00025535,-0.00000021,-0.00008350,-0.00014208,-
0.00010940,0.00018102,0.00002511,0.00002186,-
0.00005372,0.00004062,0.00000419,0.00026663,0.00018844,0.00003447,-
0.00014565,-0.00007004,-0.00006845,0.00009570,0.00003437,0.00000166,-
0.00029643,-0.00012929,-0.00001406,0.00006525,0.00006167,0.00001539,-
0.00018847,-0.00007917,0.00000011,0.00004350,-0.00001146,-
0.00002700,0.00002708,0.00002627,0.00006734,0.00000138,-0.00001609,-
0.00000211,-0.00001190,-0.00000166,0.00000734,-0.00012761,-0.00038413,-
0.00003662,0.00005064,0.00019948,0.00004925,0.00003497,0.00023914,-
0.00001168,-0.00004164,-0.00009652,-
0.00001760,0.00004219,0.00037523,0.00001334,0.00000535,-0.00011638,-
0.00001624,-0.00004678,-0.00004670,-

0.00004769,0.00000057,0.00001406,0.00001378,0.00002266,-
0.00004436,0.00004013,-0.00001222,0.00001759,-0.00000500,0.00025971,-
0.00098588,0.00044757,-
0.00196147,0.00115872,0.00016149,0.00058083,0.00572472,-0.00266258,-
0.03953224,-0.00255305,0.02340636,-0.00338398,-0.00079765,0.00235176,-
0.00121318,0.00009881,0.00015829,-0.00017959,0.00033324,-0.00014187,-
0.00174226,0.00186119,-0.00004177,-
0.00064122,0.00032849,0.00004188,0.00102950,-0.00032484,-0.00025677,-
0.21104268,-0.03631366,0.08845431,0.00665592,-
0.03617395,0.03006475,0.49361753,-0.00000784,-0.00003893,0.00000497,-
0.00006086,0.00023923,-0.00002676,0.00010756,-0.00007595,0.00000731,-
0.00002390,0.00002686,0.00000647,0.00002634,-0.00002953,-
0.00001019,0.00000664,0.00001518,-0.00000381,-0.00002503,-0.00000409,-
0.00000859,0.00000525,-0.00000736,0.00000334,-0.00004946,-0.00003295,-
0.00000147,0.00003170,0.00001719,0.00000199,-0.00002015,-0.00000373,-
0.00000454,0.00004978,0.00002207,0.00000255,-0.00000984,-0.00000764,-
0.00000309,0.00002671,0.00001527,-0.00000300,-
0.00000576,0.00000045,0.00000708,-0.00000598,-0.00001171,-
0.00000368,0.00000495,0.00000035,0.00000475,0.00000204,-
0.00000134,0.00000079,0.00000467,0.00001021,0.00000155,0.00000072,0.000001
25,-0.00001403,-0.00000017,-0.00001539,-
0.00000301,0.00000541,0.00000729,0.00000171,-0.00000193,-0.00002235,-
0.00000101,0.00000249,0.00000325,-0.00000312,-0.00000469,-
0.00000185,0.00001229,-0.00000379,0.00000497,0.00000418,-
0.00000114,0.00000270,0.00000165,0.00000005,-
0.00000137,0.00000252,0.00049010,0.00039814,-
0.00042429,0.00150453,0.00047175,-0.00108096,0.00243735,-
0.00338942,0.00176294,0.01856166,0.01257187,-0.01059185,0.00205845,-
0.00320481,0.00139357,-0.00065659,0.00091163,-0.00068272,-
0.00008174,0.00001842,0.00001061,0.00240276,-0.00032798,-
0.00066913,0.00030382,-0.00029408,-0.00010539,0.00099402,0.00005149,-
0.00041518,-0.00186554,-0.12410330,0.03578697,-0.05234765,-
0.02915852,0.05591404,-0.16915525,0.40978624,0.00021613,0.00002613,-
0.00008522,-0.00012420,-0.00001274,0.00014356,0.00004751,-
0.00018981,0.00011058,-0.00005604,-0.00004381,-0.00005925,0.00011121,-
0.00011074,0.00000640,0.00006240,0.00004840,0.00005126,-
0.00005997,0.00000072,-0.00002108,0.00002110,-0.00001550,-0.00000902,-
0.00010863,-0.00007557,-0.00001472,0.00007158,0.00002039,0.00002398,-
0.00004046,-0.00001438,0.00000215,0.00011583,0.00005097,0.00000601,-
0.00002602,-0.00002270,-0.00000327,0.00007362,0.00002737,0.00000182,-
0.00001802,0.00000842,0.00001044,-0.00001293,-0.00000742,-
0.00002998,0.00000172,0.00000883,-0.00000215,0.00000317,0.00000172,-
0.00000413,0.00005060,0.00014532,0.00001476,-0.00002978,-0.00006257,-
0.00002290,-0.00001696,-
0.00009748,0.00000784,0.00001825,0.00003761,0.00001019,-0.00001597,-
0.00014548,-0.00000451,-
0.00000247,0.00003923,0.00000721,0.00001875,0.00001528,0.00001642,-
0.00000012,-0.00000113,-0.00000744,-0.00000390,0.00001796,-
0.00001655,0.00000668,-0.00000840,0.00000076,-
0.00036727,0.00025461,0.00010995,-0.00007470,-0.00088870,0.00024365,-
0.00081683,0.00021607,0.00190931,0.01165952,-

0.00097232,0.00399872,0.00081634,0.00268899,-0.00094523,0.00059652,-
0.00098387,-0.00044649,0.00011373,-0.00018061,0.00003681,-0.00034698,-
0.00040160,0.00083696,0.00001438,-0.00010621,-0.00015384,-
0.00095326,0.00019500,0.00048871,0.06926856,0.05145712,-
0.12122462,0.03908422,0.04854249,-0.01595707,-0.11385663,-
0.12913043,0.16107351,0.00085833,0.00029117,0.00002698,-
0.00010730,0.00051289,-0.00030323,0.00034656,0.00103137,0.00064208,-
0.00054331,0.00004431,-0.00012827,0.00123756,-0.00037367,-
0.00062949,0.00012180,-0.00042617,0.00031641,-
0.00030816,0.00007279,0.00004060,-0.00016207,-0.00026845,-0.00005488,-
0.00343557,-0.00019112,0.00059527,0.00383319,0.00444672,0.00469425,-
0.02931124,-0.00203139,0.00716432,-0.18153670,-0.03687701,0.00298949,-
0.01167237,-0.02139117,-
0.01355978,0.00852784,0.00080429,0.00047969,0.00098395,0.00060857,0.000490
42,0.00041689,-0.00037941,-0.00029799,0.00144685,-0.00012664,-0.00180057,-
0.00001463,0.00377650,0.00186438,0.00007805,-
0.00085265,0.00000346,0.00005241,0.00049346,0.00006109,0.00000910,0.000446
73,-0.00007982,-0.00006116,-
0.00024555,0.00000461,0.00008448,0.00078046,0.00002919,0.00004180,-
0.00020717,0.00001955,-0.00007362,-0.00007045,-
0.00008930,0.00001780,0.00002502,0.00000914,0.00005562,-
0.00012193,0.00010699,-0.00001469,0.00003465,-0.00002369,-
0.00053447,0.00061591,-0.00001959,0.00029162,-0.00027130,0.00009567,-
0.00019398,0.00011215,0.00000198,0.00061060,-0.00044671,-0.00003515,-
0.00012819,0.00017983,-0.00001108,0.00040286,-0.00029680,-0.00004948,-
0.00010535,-0.00000674,0.00007454,0.00003983,-0.00000056,-0.00001965,-
0.00004284,0.00011159,-0.00011836,0.00000630,-0.00002195,0.00000653,-
0.00121984,0.00083369,0.00008854,0.00044233,-
0.00057230,0.00011912,0.00059302,-0.00007823,-
0.00022703,0.82387872,0.00036061,0.00033518,0.00000120,-
0.00024270,0.00002749,-0.00015605,0.00059208,0.00059437,0.00030886,-
0.00032564,-0.00070486,0.00041189,0.00030527,-0.00021315,-
0.00022836,0.00025523,-0.00022756,0.00014161,-
0.00015274,0.00003901,0.00000021,-0.00007675,-0.00012900,-
0.00003144,0.00035500,-0.00260762,-0.00144101,0.00501618,0.00326173,-
0.00531395,0.00719941,0.01268309,0.00901575,-0.04584826,-0.10814619,-
0.01169652,-0.02733791,-0.01037535,-0.00065251,-0.00075709,-0.00174298,-
0.00705961,-0.00101576,0.00010548,-0.00010491,0.00127352,0.00076600,-
0.00041261,0.00269362,-0.00286295,0.00006668,-0.00028202,-
0.00269304,0.00105724,0.00003551,-
0.00035638,0.00001614,0.00003179,0.00022034,0.00001923,0.00000439,0.000171
10,-0.00006031,-0.00001634,-0.00010127,-
0.00000971,0.00003652,0.00033902,0.00001220,0.00002547,-
0.00009640,0.00000577,-0.00003674,-0.00003228,-
0.00002955,0.00000443,0.00001152,0.00000951,0.00000880,-
0.00004230,0.00006928,-0.00001794,0.00000553,0.00000095,-
0.00029704,0.00033786,0.00001183,0.00017382,-0.00016359,0.00002962,-
0.00011740,0.00005969,-0.00001080,0.00033447,-0.00024399,-0.00002043,-
0.00006995,0.00008767,-0.00000994,0.00020454,-0.00016128,-0.00003367,-
0.00005238,-0.00000095,0.00004944,0.00002320,0.00000239,-0.00000493,-
0.00002054,0.00007916,-0.00004215,0.00001695,-0.00001024,0.00001530,-

0.00060551,0.00048245,0.00003330,0.00021056,-
0.00033770,0.00006070,0.00027582,-0.00006169,-0.00011358,-
0.04308298,0.49556454,0.00015028,-0.00012870,-
0.00003920,0.00006133,0.00017324,-0.00003177,0.00000199,-
0.00000815,0.00003281,-0.00036390,0.00006914,-
0.00076403,0.00025360,0.00010983,0.00003453,-0.00020555,0.00001922,-
0.00000204,-0.00007501,0.00005578,0.00005745,-
0.00001401,0.00003774,0.00005169,0.00111889,-0.00135288,-
0.00248381,0.00549674,-
0.00480087,0.00206814,0.01455884,0.01176517,0.00419014,-0.00434704,-
0.01458701,-0.08701272,-0.01818216,-0.00256293,0.00485285,-0.00136777,-
0.00745941,0.00131815,-0.00094232,-
0.00070181,0.00031944,0.00120066,0.00011657,0.00038087,0.00050465,0.001040
76,-0.00334141,-0.00149601,-0.00020581,-0.00353988,0.00000773,-0.00003162,-
0.00000940,0.00000945,0.00002099,-0.00000535,0.00002412,0.00002559,-
0.00001187,-0.00000208,-0.00001463,-
0.00000681,0.00000524,0.00004159,0.00000126,-0.00000015,-0.00001118,-
0.00000187,-0.00000895,-0.00000218,-0.00000049,-
0.00000290,0.00000038,0.00000501,-0.00000634,-0.00000215,0.00001838,-
0.00000747,0.00000092,0.00000866,-0.00006774,0.00007143,-
0.00000874,0.00001339,-0.00004567,0.00000619,-0.00002203,0.00001230,-
0.00000679,0.00007219,-0.00005344,-0.00000503,-0.00001249,0.00002236,-
0.00000503,0.00004249,-0.00004126,-0.00001432,-
0.00000904,0.00000466,0.00001255,0.00000777,0.00000237,0.00000218,-
0.00000136,0.00002233,-0.00000177,0.00000408,0.00000169,0.00001095,-
0.00012338,0.00010448,0.00001904,0.00003859,-0.00007512,-
0.00000188,0.00005530,-0.00001733,-0.00003538,-
0.23012182,0.25366615,0.48041088,-0.00041225,-0.00018660,-
0.00001784,0.00010934,-0.00022081,0.00012997,-0.00029473,-0.00044437,-
0.00033121,0.00037540,0.00004863,-0.00003497,-
0.00059134,0.00016737,0.00026810,-0.00008009,0.00019572,-
0.00011619,0.00019404,-
0.00008097,0.00001241,0.00008508,0.00013008,0.00000960,0.00086860,0.001362
49,0.00086980,-0.00367052,-0.00085592,0.00008661,-0.00079737,-0.00732662,-
0.00480179,-
0.05083998,0.01080766,0.03065746,0.00030470,0.00508102,0.00407100,-
0.00135062,0.00034641,0.00047180,-0.00026719,-0.00027925,-0.00017539,-
0.00094583,-0.00023644,0.00003082,0.00034786,0.00008872,0.00012166,-
0.00142276,-0.00099447,-0.00046502,-0.00003924,0.00045217,-0.00000044,-
0.00002513,-0.00026203,-0.00003490,-0.00000221,-
0.00024055,0.00003898,0.00003406,0.00013298,-0.00000508,-0.00004520,-
0.00041942,-0.00001550,-0.00002257,0.00011099,-
0.00001014,0.00003785,0.00003709,0.00004774,-0.00001024,-0.00001278,-
0.00000389,-0.00003219,0.00006633,-0.00005239,0.00000592,-
0.00001897,0.00001512,0.00026054,-0.00030081,0.00000939,-
0.00014717,0.00013017,-0.00005879,0.00009815,-0.00005989,-0.00000379,-
0.00031255,0.00022711,0.00001862,0.00006467,-0.00009265,0.00000513,-
0.00020229,0.00014939,0.00002010,0.00005357,0.00000378,-0.00003364,-
0.00001946,0.00000076,0.00001192,0.00002254,-
0.00005170,0.00006937,0.00000115,0.00001132,0.00000039,0.00064557,-
0.00042274,-0.00004859,-0.00023729,0.00028829,-0.00006338,-

0.00031924,0.00003523,0.00011884,-
0.47379643,0.15946898,0.25512700,0.51369780,-0.00006092,-
0.00005356,0.00000690,0.00006343,0.00005109,-0.00000641,-0.00021236,-
0.00007102,-0.00000238,0.00024742,0.00035548,0.00005752,-0.00000702,-
0.00008438,-0.00002098,-0.00000230,0.00003964,0.00000365,0.00002298,-
0.00000322,0.00001367,0.00001302,0.00004441,-0.00001632,-
0.00088579,0.00024462,0.00042268,-0.00050769,0.00018635,0.00147767,-
0.00252946,-0.00048256,-0.00210451,-0.00031403,0.01884336,0.00010511,-
0.00278677,-0.00382210,-
0.00245831,0.00121756,0.00110808,0.00110809,0.00012542,0.00001240,-
0.00003027,-0.00016045,-0.00022923,0.00018167,-
0.00084447,0.00060189,0.00025482,-0.00074734,0.00134472,0.00163194,-
0.00000376,0.00002393,-0.00000192,-0.00000561,-
0.00002565,0.00000213,0.00000027,-0.00000625,0.00002296,-
0.00000290,0.00001021,0.00000520,-0.00000528,-0.00004108,-0.00000183,-
0.00000579,0.00001336,-0.00000138,0.00000386,0.00000445,-
0.00000125,0.00000044,-0.00000149,-0.00000320,0.00000578,-0.00000092,-
0.00001977,0.00000782,0.00000558,-0.00000483,0.00005842,-0.00006483,-
0.00000676,-0.00003826,0.00003541,-0.00000088,0.00002616,-
0.00001249,0.00000436,-0.00006701,0.00004911,0.00000411,0.00001375,-
0.00001591,0.00000188,-0.00003597,0.00003089,0.00000816,0.00000908,0.,-
0.00001042,-0.00000429,-0.00000126,-0.00000006,0.00000433,-
0.00001887,0.00000410,-0.00000597,0.00000177,-0.00000492,0.00010888,-
0.00010571,-0.00000035,-0.00003638,0.00007546,-0.00001368,-
0.00004432,0.00001720,0.00001870,0.15946221,-0.17844681,-0.13458332,-
0.19866588,0.18765178,0.00006107,0.00007424,0.00001619,-
0.00003204,0.00005182,-0.00004126,-
0.00003761,0.00012845,0.00013548,0.00017499,0.00021842,0.00022468,0.000092
21,-0.00016509,-0.00010344,0.00010103,-0.00004610,0.00004257,-
0.00002780,0.00000174,-0.00002564,-0.00002065,-0.00001472,-0.00003298,-
0.00099901,-0.00030598,0.00020005,0.00023229,0.00158542,-0.00037991,-
0.00082565,-0.00056410,0.00278956,0.02160657,-0.00335839,0.00934846,-
0.00235571,-0.00487930,-
0.00314445,0.00128478,0.00126547,0.00018938,0.00019007,0.00009808,0.000043
49,0.00002338,0.00020558,-0.00019925,-0.00059641,-0.00007209,0.00080998,-
0.00026665,0.00170152,0.00114367,0.00000996,-
0.00010193,0.00000167,0.00000242,0.00006069,0.00001381,-
0.00001416,0.00004533,-0.00000510,-0.00000832,-
0.00002255,0.00000441,0.00000895,0.00008584,0.00000329,0.00000577,-
0.00002382,0.00000487,-0.00000363,-0.00000940,-
0.00001343,0.00000474,0.00000406,-0.00000156,0.00001133,-
0.00001380,0.00000348,0.00000107,0.00000297,-0.00000786,-
0.00005007,0.00005447,-0.00000298,0.00004224,-0.00001648,0.00001011,-
0.00001887,0.00001168,0.00000528,0.00005251,-0.00003841,-0.00000268,-
0.00001218,0.00001470,0.00000232,0.00003736,-0.00002172,0.00000056,-
0.00001223,-0.00000482,0.00000451,0.00000149,-0.00000149,-0.00000403,-
0.00000708,0.00000489,-0.00001917,0.00000042,-0.00000494,-0.00000520,-
0.00010871,0.00007248,-0.00000344,0.00004250,-
0.00004862,0.00002233,0.00005247,-0.00000445,-0.00001042,0.25504854,-
0.13462703,-0.26440447,-0.31280438,0.18890456,0.28449520,0.00007618,-
0.00000687,-0.00003404,-0.00005838,0.00004914,-0.00001364,0.00002698,-

0.00007017,0.00002040,-0.00002214,-0.00001716,-0.00001502,0.00004905,-
0.00004402,-0.00000023,0.00001618,0.00003127,0.00001875,-
0.00002318,0.00000190,0.00000131,0.00000985,-0.00000585,-0.00000084,-
0.00004213,-0.00002961,-0.00000427,0.00001815,0.00001322,0.00001410,-
0.00001436,-0.00000539,-0.00000032,0.00005122,0.00002230,0.00000253,-
0.00001166,-0.00001164,-0.00000338,0.00003286,0.00001302,-0.00000098,-
0.00000723,0.00000103,0.00000406,-0.00000309,-0.00000382,-0.00001098,-
0.00000251,0.00000245,0.00000037,0.00000215,0.00000004,-
0.00000145,0.00002024,0.00006640,0.00000683,-0.00000892,-0.00003424,-
0.00001110,-0.00000429,-
0.00004139,0.00000200,0.00000813,0.00001791,0.00000306,-0.00000792,-
0.00006924,-0.00000269,-
0.00000044,0.00002153,0.00000228,0.00000721,0.00000775,0.00001011,-
0.00000046,-0.00000150,-0.00000183,-0.00000493,0.00000766,-
0.00000644,0.00000224,-0.00000263,0.00000157,0.00000451,-
0.00001129,0.00002294,-0.00031217,-0.00062422,0.00031588,-
0.00085075,0.00140474,-0.00016096,-0.00161122,-
0.00362379,0.00512746,0.00049565,-
0.00086544,0.00005682,0.00034906,0.00011884,-0.00016618,0.00001058,-
0.00002920,0.00002355,0.00009875,0.00026577,-0.00030461,-
0.00008587,0.00008386,-0.00004848,-0.00015818,-0.00001190,0.00016124,-
0.04325568,0.03453274,0.00259559,0.00815720,-0.00050883,-0.00643040,-
0.24200027,0.19531936,-0.00537182,-0.00011065,-0.00004380,-
0.00000401,0.00006224,0.00000553,-0.00001297,0.27907974,0.00000298,-
0.00000293,-0.00000259,-0.00001034,0.00000476,-
0.00001754,0.00000745,0.00000199,-0.00000668,-
0.00000138,0.00000773,0.00000836,-0.00000376,-
0.00000744,0.00000162,0.00000194,0.00000167,0.00000082,0.00000099,-
0.00000015,-0.00000276,0.00000084,0.00000107,-
0.00000171,0.00000971,0.00000413,0.00000072,-0.00000735,-
0.00000285,0.00000127,0.00000409,0.00000063,0.00000077,-0.00000758,-
0.00000367,-0.00000060,0.00000150,0.00000077,-0.00000006,-0.00000338,-
0.00000137,0.00000097,0.00000089,-0.00000073,-
0.00000147,0.00000116,0.00000243,0.00000006,-0.00000172,0.00000007,-
0.00000095,0.,0.00000040,-0.00000012,0.00000250,0.00000961,0.00000127,-
0.00000029,-0.00000952,0.00000255,-0.00000105,-0.00000239,0.00000052,-
0.00000059,0.00000050,-0.00000050,-0.00000091,-0.00000580,-0.00000019,-
0.00000018,0.00000449,0.00000178,0.00000242,0.00000291,-
0.00000110,0.00000126,-0.00000279,-0.00000152,-0.00000107,0.00000041,-
0.00000143,0.00000005,0.00000018,-0.00000063,0.00003729,-
0.00011643,0.00007633,-0.00021523,0.00005391,-0.00016257,-
0.00010146,0.00040952,0.00013110,-0.00153986,0.00068150,0.00321540,-
0.00017407,-0.00031806,0.00000143,0.00010490,0.00011407,-
0.00003416,0.00001815,-0.00001131,0.00000851,0.00006742,0.00027386,-
0.00031891,-0.00004390,-0.00000489,-
0.00001990,0.00000848,0.00006741,0.00005532,-0.00797976,0.01616118,-
0.00281741,-0.00047043,-0.00585486,-0.00018395,0.23629587,-
0.26387369,0.02957653,0.00000902,0.00001414,0.00000700,-0.00000206,-
0.00000494,-0.00000195,-0.22600755,0.25240882,-
0.00002869,0.00002489,0.00000474,0.00002156,-0.00007280,-0.00000731,-
0.00001973,0.00003609,-0.00001412,-0.00000081,0.00001316,0.00001204,-

0.00002915,0.00000480,-0.00000216,0.00000842,-0.00001513,-
0.00000325,0.00001094,0.00000056,0.00000201,-
0.00000441,0.00000335,0.00000029,0.00002554,0.00001786,0.00000440,-
0.00002026,-0.00000447,-0.00000336,0.00001055,0.00000363,-0.00000031,-
0.00002608,-0.00001176,-0.00000154,0.00000556,0.00000471,-0.00000011,-
0.00001546,-0.00000532,-0.00000053,0.00000393,-0.00000299,-
0.00000226,0.00000404,0.00000229,0.00000605,-0.00000182,-
0.00000226,0.00000047,-0.00000009,-0.00000049,0.00000104,-0.00000679,-
0.00001765,-
0.00000055,0.00000761,0.00000171,0.00000777,0.00000334,0.00001662,-
0.00000153,-0.00000390,-0.00000655,-
0.00000318,0.00000217,0.00002432,0.00000045,0.00000010,-0.00000380,-
0.00000108,-0.00000203,-0.00000031,-0.00000364,0.00000058,-
0.00000293,0.00000123,-0.00000131,-0.00000305,0.00000208,-
0.00000179,0.00000187,-0.00000016,-
0.00000464,0.00011216,0.00000183,0.00007035,0.00002739,-
0.00048339,0.00069352,-
0.00055516,0.00056217,0.00398736,0.00415886,0.00109791,-
0.00033532,0.00031612,-0.00050748,-0.00014675,-0.00003327,0.00024583,-
0.00000539,0.00002838,0.00000199,-0.00019144,-
0.00041371,0.00008671,0.00002171,-0.00008182,-
0.00003908,0.00014914,0.00006813,0.00007105,0.02618698,-
0.02217478,0.00491018,-0.00646988,-0.00016889,-0.00353667,-
0.02810758,0.04824154,-0.03008395,0.00004632,0.00003143,0.00001484,-
0.00002180,-0.00000682,-0.00000215,0.00419820,-0.02951001,0.02767428,-
0.29761271,-0.10006214,-0.00781842,-0.00061505,-0.00064764,-
0.00009138,0.00148351,-
0.00019919,0.00007383,0.00030789,0.00054596,0.00005790,-
0.00189149,0.00417399,0.00016804,-0.01964888,-0.01103661,-0.00073719,-
0.00004270,0.00005511,0.00008937,-0.00101877,-0.00061832,-
0.00011964,0.00006886,-0.00001180,-0.00001332,-0.00004430,-
0.00002000,0.00001617,0.00001111,0.00000043,-0.00002302,-0.00006581,-
0.00003635,-0.00001307,0.00004184,0.00000942,0.00001253,-0.00005839,-
0.00001120,0.00003679,0.00002419,-0.00000382,-0.00005039,0.00001210,-
0.00000110,0.00001219,-0.00000538,-0.00000441,-0.00000249,-0.00000380,-
0.00000161,0.00000242,0.00028832,-0.00192321,-0.00008509,0.00009285,-
0.00006961,-0.00076239,-0.00004796,0.00052910,-0.00026326,-0.00010199,-
0.00031470,-0.00005969,0.00014468,-
0.00010654,0.00021705,0.00038143,0.00026835,0.00007248,0.00046978,-
0.00020021,0.00060632,0.00001602,0.00003242,-0.00003056,0.00005427,-
0.00004968,0.00007671,-0.00001218,0.00001696,0.00002806,-
0.00015660,0.00083752,0.00007983,0.00030098,-0.00024709,0.00015771,-
0.00012737,0.00002997,0.00004063,0.00002149,-0.00002124,-
0.00007048,0.00008482,0.00018297,-0.00013512,-
0.00015366,0.00049616,0.00070000,-
0.00015550,0.00096568,0.00019947,0.00004050,0.00001635,0.00005590,-
0.00000514,0.00002390,-0.00001026,0.00000377,-0.00002758,-0.00002743,-
0.00008003,0.00005655,-0.00001435,0.00002114,-
0.00002023,0.00001350,0.00004328,-0.00000219,-
0.00001906,0.00013688,0.00006217,0.00000886,-0.00007225,-
0.00001102,0.00001294,-0.00000671,0.00000046,0.00000216,0.31786952,-

0.10020776,-0.10130510,-0.00331329,-0.02943022,-0.00809258,-
0.00085211,0.00082088,-0.00573090,-0.00077896,0.00055717,-0.00079430,-
0.00004118,0.00313186,-0.00234138,-
0.00063316,0.01798683,0.01074958,0.00054932,-0.00158066,-0.00046883,-
0.00003377,0.00102254,0.00051006,-0.00003285,-
0.00001221,0.00008694,0.00008845,-0.00000721,-0.00002114,-
0.00007124,0.00002496,0.00003348,0.00001576,-0.00003130,-
0.00000765,0.00001225,-0.00002111,0.00001270,0.00002903,-0.00001972,-
0.00002037,-0.00006296,-
0.00001479,0.00002074,0.00003241,0.00000421,0.00003175,0.00005942,0.000000
19,-0.00000033,-0.00000154,-0.00000450,-
0.00000146,0.00000036,0.00041715,0.00103294,0.00022739,0.00023617,-
0.00049280,-0.00027225,0.00011651,-
0.00003294,0.00005394,0.00004104,0.00017170,0.00000519,-
0.00017595,0.00015294,-0.00013603,-0.00009905,-
0.00041020,0.00015348,0.00030798,-0.00083476,-0.00082883,0.00004334,-
0.00000112,-0.00004687,-0.00003539,0.00001720,-0.00005675,-
0.00001497,0.00001215,0.00000943,-
0.00152358,0.00146657,0.00023468,0.00020057,-0.00038509,0.00022761,-
0.00033115,0.00016348,0.00003863,0.00004644,0.00030568,-
0.00024780,0.00058866,-0.00011457,0.00009038,0.00008809,-
0.00027435,0.00039772,0.00041111,-0.00034790,-0.00104949,-0.00000063,-
0.00002901,-0.00000877,0.00001656,0.00008120,-0.00009025,0.00000387,-
0.00000378,-0.00001020,0.00028965,-0.00018179,-0.00009516,-
0.00012018,0.00017076,-0.00001648,-
0.00012981,0.00002717,0.00005501,0.00005358,0.00003596,0.00000775,-
0.00003154,-0.00000675,0.00000633,0.00002626,0.00000140,-
0.00001603,0.10739283,0.10688543,-0.00741937,-0.00420772,-0.03585999,-
0.00287041,0.00010434,0.00291126,0.00058578,-0.00096394,0.00636526,-
0.00015913,0.00048548,-0.00081324,-0.00007204,-
0.00106008,0.00636147,0.00118336,0.00171622,0.00306756,-
0.00009548,0.00004895,0.00031006,-0.00001526,0.00007350,0.00030546,-
0.00002336,0.00009981,-0.00069531,-0.00024227,-0.00010262,-
0.00002134,0.00024792,0.00000835,-0.00009807,-
0.00006652,0.00014681,0.00014948,-0.00017268,-0.00016898,-
0.00010999,0.00024171,0.00009839,-0.00000595,0.00003349,-
0.00004860,0.00000915,0.00001153,-0.00005799,0.00001039,-
0.00000748,0.00000830,-0.00001373,-0.00000086,0.00001181,-0.00001374,-
0.00044893,-0.00090499,-0.00350594,0.00080596,-0.00110367,0.00032571,-
0.00016869,0.00083610,-0.00004481,-0.00041913,-0.00024497,-
0.00012652,0.00029163,-0.00009710,0.00023303,-0.00027812,0.00056393,-
0.00032005,-0.00015301,-0.00091114,-0.00062158,0.00001740,-0.00004474,-
0.00000962,0.00000181,0.00003074,-0.00005902,0.00001316,0.00002491,-
0.00001576,0.00109980,-0.00020090,-0.00347261,-0.00045145,0.00068468,-
0.00003189,0.00045635,0.00013595,-0.00009712,-0.00014217,-
0.00026564,0.00020043,-0.00017420,0.00059976,-0.00031931,0.00017587,-
0.00133685,0.00033466,0.00072621,-0.00051034,-0.00056730,0.00002284,-
0.00004141,-0.00000520,-0.00002081,0.00002340,-0.00006195,-
0.00002487,0.00000386,-0.00001862,-0.00000617,0.00002028,0.00005097,-
0.00002457,0.00001759,-0.00002691,-0.00000550,0.00001594,-
0.00002001,0.00000279,-0.00000157,0.00004295,-0.00000216,-0.00000239,-

0.00001174,0.00000381,0.00000165,0.00000296,0.00756203,0.00644397,0.026291
24,0.00018223,0.00028547,0.00013645,-0.00006345,-0.00013725,-
0.00004537,0.00017641,0.00002815,-0.00001786,-
0.00011817,0.00024424,0.00004093,-0.00017231,-0.00028822,-0.00007089,-
0.00051907,0.00029814,0.00043937,0.00001107,0.00001002,-0.00000087,-
0.00001101,-0.00001447,-0.00001895,-0.00001393,-
0.00000184,0.00002003,0.00001741,0.00001639,-0.00000412,-
0.00001305,0.00000407,-0.00000237,0.00001523,0.00000632,-0.00000019,-
0.00000116,0.00000758,-0.00001005,-0.00001015,0.00000126,-
0.00002145,0.00001080,-0.00001158,0.00002143,-0.00000074,-
0.00000795,0.00000523,0.00000207,-0.00000216,0.00000529,-0.00000361,-
0.00001208,0.00000952,-0.00300017,-0.00054928,-
0.00157830,0.00036691,0.00590347,-0.00583709,-0.00119341,-0.00188868,-
0.00701691,0.00019876,-0.02502714,0.00225924,-0.09151967,-0.00520048,-
0.01197163,0.01086098,0.01464237,0.00725427,0.00048320,0.00135976,-
0.00022686,-0.00350201,0.00235096,0.00018578,0.00026543,-0.00121751,-
0.00030798,-0.00284629,-0.00030273,0.00094167,0.00010538,-
0.00005182,0.00000331,-0.00004927,0.00002683,-0.00000951,0.00001647,-
0.00001043,-0.00000630,-0.00007765,0.00005656,0.00000383,0.00002137,-
0.00003039,-0.00001436,-0.00006470,0.00003802,-
0.00000707,0.00003147,0.00000493,0.00000647,-
0.00000603,0.00001243,0.00001419,0.00000492,-
0.00000925,0.00001785,0.00000053,0.00000547,0.00000419,0.00016095,-
0.00004130,-0.00002347,-0.00005454,0.00002237,-0.00000994,-0.00009636,-
0.00002017,0.00002241,-0.00003724,-0.00007103,-
0.00001961,0.00001070,0.00003744,-
0.00000421,0.00001795,0.00001443,0.00000724,0.00001379,-0.00002427,-
0.00000054,0.63931771,0.00100889,0.00081237,0.00068740,0.00050797,-
0.00002472,-0.00036537,0.00011585,0.00101050,0.00006258,-
0.00049907,0.00002551,0.00036575,-0.00075579,0.00116055,-0.00058809,-
0.00007012,-0.00072323,-0.00008476,0.00001854,0.00016215,0.00000805,-
0.00025193,-0.00025429,-0.00005336,-0.00091788,-
0.00018190,0.00003210,0.00052277,0.00021658,0.00005898,-0.00024620,-
0.00002816,0.00002267,0.00079377,0.00034709,0.00004078,-0.00022433,-
0.00013405,0.00000101,0.00044212,0.00014361,-0.00010291,-
0.00013765,0.00001640,0.00013089,-0.00005743,-0.00009678,-
0.00009663,0.00002338,0.00003921,0.00002185,0.00002736,0.00000148,-
0.00001521,0.00003096,-
0.00314575,0.00013590,0.00424117,0.00529728,0.00328795,-
0.00130165,0.00805702,-0.00086974,-0.01546018,-0.01409015,-0.01169397,-
0.01450602,-0.19950268,-0.00580387,0.00846887,-0.03456747,0.00842704,-
0.00033108,0.00052193,-0.00034397,-0.00186562,0.00086089,-
0.00169932,0.00050907,0.00093874,0.00049254,0.00263732,0.00140215,0.001843
41,0.00083512,-0.00037836,-0.00007498,-0.00043632,0.00032489,-
0.00001727,0.00022079,-0.00006907,-0.00003365,-
0.00068830,0.00050092,0.00003750,0.00019758,-0.00018297,-0.00002227,-
0.00039961,0.00023569,0.00013642,0.00013101,-0.00000655,-0.00013449,-
0.00003862,-0.00000477,0.00002019,0.00002909,-0.00011775,0.00008067,-
0.00001548,0.00002952,-0.00001000,0.00140913,-0.00088338,-0.00014925,-
0.00050569,0.00060465,-0.00011206,-0.00070563,0.00006447,0.00027973,-
0.00152426,-0.00068988,-0.00009655,0.00081801,0.00010327,-

0.00015622,0.00013615,0.00000293,-0.00005574,0.00016634,-
0.00030551,0.00002624,0.13767601,0.69139596,0.00004871,-
0.00021897,0.00006611,0.00016563,0.00017191,-0.00002140,-
0.00018544,0.00002242,-0.00003172,0.00008027,-0.00014351,-0.00001899,-
0.00006518,0.00010927,-0.00001395,0.00022829,0.00027574,-
0.00077123,0.00000504,-
0.00000207,0.00004252,0.00006722,0.00001111,0.00005340,0.00001797,0.000008
30,-0.00000877,-0.00001377,0.00000283,-0.00000815,0.00000493,-0.00000241,-
0.00000402,-0.00001567,-0.00000620,-0.00000155,0.00000229,0.00000188,-
0.00000599,-0.00000809,0.00002271,0.00000120,0.00000779,-
0.00000773,0.00000700,0.00000432,-0.00000356,0.00000416,0.00000012,-
0.00000476,0.00000314,0.00000231,-0.00000474,0.00000887,-0.00171832,-
0.00033509,-0.00237702,-0.00539906,0.00502978,0.00243933,-0.00692563,-
0.00180612,0.00229961,0.00174805,-0.01914061,0.00143050,-
0.01160027,0.00169533,-0.08575333,0.00741226,0.01315770,0.00650793,-
0.00017216,0.00113598,0.00040428,0.00044229,0.00167201,-0.00358853,-
0.00045329,-0.00100891,0.00035886,0.00084334,-0.00055478,-
0.00332580,0.00003577,-0.00002058,-0.00001401,-0.00002211,0.00000838,-
0.00000843,0.00000871,0.00000051,-0.00000376,-
0.00002254,0.00001544,0.00000096,0.00000410,-0.00000496,-0.00000679,-
0.00002186,-0.00001388,-
0.00000339,0.00001202,0.00000425,0.00000717,0.00000180,0.00000447,0.000010
16,0.00000502,-
0.00000080,0.00000628,0.00000062,0.00000413,0.00000416,0.00004058,-
0.00000988,0.00000728,-0.00001624,0.00000492,-0.00001445,-0.00002402,-
0.00001002,-0.00000938,0.00002460,-0.00000274,0.00001062,-
0.00001514,0.00001147,-
0.00001149,0.00000596,0.00000839,0.00001158,0.00005576,-
0.00003628,0.00005164,0.34171575,0.08633830,0.46883869,-0.00029298,-
0.00015970,-0.00015917,-0.00007456,-0.00000874,0.00006493,-0.00003462,-
0.00016045,0.00000281,0.00013315,-0.00007074,-0.00008355,0.00016809,-
0.00006373,0.00015780,0.00023880,-0.00017865,0.00005106,-0.00000476,-
0.00002823,-
0.00000361,0.00007193,0.00004836,0.00000625,0.00012349,0.00002356,-
0.00001339,-0.00007791,-0.00003702,-0.00000316,0.00003901,0.00000174,-
0.00000228,-0.00011287,-0.00005010,-
0.00000598,0.00003075,0.00001322,0.00000594,-0.00005013,-
0.00002152,0.00002908,0.00001102,0.00000463,-
0.00003207,0.00000814,0.00001705,0.00000922,-0.00000459,-0.00000392,-
0.00000628,-0.00000103,0.00000752,-
0.00000396,0.00019929,0.00131199,0.00004354,-0.00014223,-
0.00200938,0.00056443,-0.00009745,-0.00163784,0.00109030,0.00372572,-
0.00008222,0.00054090,-0.00416773,-0.02650021,-0.01706751,-
0.00402401,0.00563026,-0.00366968,0.00001126,-
0.00029688,0.00003765,0.00147263,-0.00102614,0.00157003,-0.00022531,-
0.00026489,0.00012110,0.00087956,0.00055166,0.00020728,-
0.00018935,0.00008917,0.00001367,0.00009288,-0.00006329,0.00001062,-
0.00004197,0.00001628,0.00000695,0.00014478,-0.00010426,-0.00000809,-
0.00004048,0.00004551,0.00001267,0.00010038,-0.00005849,-0.00000879,-
0.00004026,-0.00000234,0.00001058,0.00001007,-0.00000944,-0.00001318,-
0.00000627,0.00002330,-0.00002311,0.00000169,-0.00000748,-0.00000149,-

0.00029449,0.00013647,0.00003966,0.00010243,-
0.00008853,0.00001867,0.00016079,0.00000818,-
0.00005357,0.00022327,0.00013774,0.00002736,-0.00011327,-
0.00003840,0.00002179,-0.00003030,-0.00001117,0.00000238,-
0.00000835,0.00004300,-0.00001606,-0.40804125,-0.19200999,-
0.25599651,0.46447279,-0.00033854,-0.00037636,-0.00023251,-
0.00018693,0.00003069,0.00010472,-0.00003380,-0.00042063,-
0.00002450,0.00019706,0.00003551,-0.00011097,0.00018231,-
0.00054008,0.00022504,0.00002636,0.00051893,0.00002516,-0.00000718,-
0.00007298,-0.00000201,0.00014972,0.00011862,-
0.00001058,0.00037352,0.00007360,-0.00001276,-0.00021883,-0.00009067,-
0.00002076,0.00010558,0.00001074,-0.00000897,-0.00033809,-0.00014881,-
0.00001791,0.00009747,0.00005489,0.00000181,-0.00018061,-
0.00006396,0.00005195,0.00005447,-0.00000312,-
0.00006310,0.00002379,0.00004143,0.00003672,-0.00000976,-0.00001554,-
0.00001096,-0.00000973,0.00000322,0.00000321,-0.00102559,0.00091037,-
0.00086656,-0.00108865,0.00016209,-0.00092573,-0.00128509,-0.00313288,-
0.00099799,0.00490438,-0.00544171,0.00433798,-0.03804282,-0.02510514,-
0.02819393,-0.00253917,0.00007708,-0.00193619,0.00012634,-
0.00025853,0.00013723,-0.00078582,-0.00140415,-0.00058936,-0.00019094,-
0.00091422,-0.00017067,-0.00041537,0.00006424,-0.00027978,-
0.00035842,0.00016480,0.00002920,0.00019131,-0.00013926,0.00000865,-
0.00009771,0.00003008,0.00001522,0.00030549,-0.00022268,-0.00001654,-
0.00008847,0.00008179,0.00001195,0.00017610,-0.00010106,-0.00006052,-
0.00005833,0.00000202,0.00005746,0.00001574,0.00000216,-0.00001041,-
0.00001339,0.00004975,-0.00003473,0.00000598,-0.00001322,0.00000380,-
0.00063121,0.00039166,0.00006566,0.00022719,-
0.00026787,0.00005156,0.00031749,-0.00002662,-
0.00012256,0.00065924,0.00031255,0.00004455,-0.00035178,-
0.00005472,0.00006881,-0.00006229,-0.00000289,0.00002280,-
0.00007630,0.00013352,0.00001718,-0.19204584,-0.23692398,-
0.14500272,0.21927064,0.23165602,-0.00021170,-0.00001547,-0.00011004,-
0.00010220,-0.00

1\1\GINC-ACID10\Freq\RB3LYP\6-31G(d)\C24H15N3O6\WINKLER\13-Sep-
2016\0\\#N Geom=AllCheck Guess=TCheck SCRF=Check GenChk RB3LYP/6-
31G(d) Freq\\Title\\0,1\N,-
1.1401903877,0.7577732869,0.0144617401\C,0.0826720638,1.3094158965,0.0143
672534\N,1.2261866429,0.608207938,0.0139165625\C,1.092513972,-
0.7266172174,0.0135180922\N,-0.0865112887,-1.3663533129,0.0135674073\C,-
1.1756840739,-0.5831834696,0.014062193\C,2.3284296408,-
1.5484450986,0.012919009\C,3.5896133783,-
0.9318426151,0.0131400316\C,4.7488770009,-
1.6995803864,0.0125841997\C,4.662984125,-
3.0989243532,0.0117735747\C,3.4057109758,-
3.7183505391,0.0115475627\C,2.2482616759,-
2.9506941969,0.0121261525\H,1.2724924368,-
3.4219046119,0.0119727392\H,3.6454627214,0.150323436,0.0137592061\H,5.721
5124362,-1.2206940972,0.012772477\H,3.3587886788,-
4.8021754951,0.010916616\C,-2.5053637508,-1.2426068299,0.0141250296\C,-
3.6796776068,-0.4720637117,0.0145208426\C,-2.6020892048,-

2.6431064378,0.0137975795\C,-3.8466351731,-3.2631476504,0.0138667694\C,-
5.0155556488,-2.4891041075,0.0142592757\C,-4.9232500296,-
1.0905654,0.0145853889\H,-3.5999529373,0.6085848988,0.0147695643\H,-
5.8383092278,-0.5078432348,0.0148957946\H,-1.6929648434,-
3.2327680685,0.0135025055\H,-3.9181455502,-
4.344924074,0.0136126733\C,0.1766135797,2.7906499402,0.0147616084\C,1.431
1330907,3.4222270386,0.0145904612\C,1.5174236803,4.8084132122,0.014930077
\C,0.3525028282,5.5877730851,0.015457089\C,-
0.9023745657,4.9626204397,0.0156335346\C,-
0.9878215806,3.574781762,0.0152833484\H,-
1.9530418665,3.0822856712,0.015399103\H,-
1.803450182,5.5654879992,0.0160356863\H,2.3271184832,2.8128174366,0.01418
16237\H,2.4796510128,5.3094018123,0.0147898087\C,0.5036221955,7.068404954
9,0.0157950049\O,1.5658812784,7.6572063633,0.0155856007\O,-
0.6853021335,7.7231128823,0.0163394529\C,5.8700371511,-
3.9696517089,0.0111345348\O,5.849362635,-5.1839991961,0.0103906983\H,-
0.463908874,8.6727906505,0.0164876452\C,-6.3731260866,-
3.0991589712,0.0143503532\O,-7.4145149093,-2.4742097096,0.0146787463\O,-
6.3448330076,-4.4561548059,0.0140134191\H,-7.2777858606,-
4.7398863844,0.0141027738\O,7.0311450898,-
3.2667551767,0.0114906088\H,7.7432820157,-
3.9329178437,0.0110185815\\Version=EM64L-G09RevD.01\State=1-A\HF=-
1539.2795012\RMSD=3.353e-09\RMSF=2.425e-
05\ZeroPoint=0.353128\Thermal=0.3798692\Dipole=0.000178,0.0001152,0.0002695
\DipoleDeriv=-1.210724,-0.6152929,0.0000576,-0.6220184,-1.7277905,-
0.0003074,0.0000229,-0.0003112,-0.2255822,1.0180206,0.0907227,-
0.0001646,0.1001259,2.5006834,0.0006468,-0.0001254,0.0006066,0.2069222,-
1.062974,0.5364137,0.0003587,0.5299413,-1.8762089,-0.0006257,0.0003583,-
0.0006043,-0.2255565,2.047764,-0.6945062,-0.0006813,-
0.6850411,1.4710996,0.0005511,-0.0007075,0.0005846,0.2069031,-
2.1347291,0.088729,0.0004829,0.0822808,-0.8041025,-0.0001821,0.0005029,-
0.0002203,-0.2256463,2.2120155,0.589633,-
0.0002788,0.5993992,1.3058623,0.0001989,-0.0002735,0.0002351,0.2069536,-
0.4290899,0.2965724,0.0002084,0.3051353,-0.190102,-0.0001275,0.000227,-
0.0001288,-0.0056582,-0.1222136,-0.1219315,-
0.000069,0.1283589,0.0349678,0.000068,0.0000272,0.0001133,-
0.0909517,0.1598189,0.1068629,0.0000459,-0.0449429,-0.0837249,0.0000097,-
0.0000449,-0.0000086,-0.0952467,-0.5986359,0.3961427,0.0002833,0.3683137,-
0.2265807,-0.0001771,0.0002785,-0.0001959,0.027497,-0.0851579,-0.130016,-
0.0000842,0.0381956,0.1260025,0.0001198,0.0000207,0.0001451,-
0.08905,0.0251101,0.180297,0.0000913,-0.095603,-0.0834763,0.0000092,-
0.0000234,-0.0000565,-0.0917801,0.0321306,-0.0680369,-0.000034,-
0.0909183,0.0993617,0.0000042,-0.0000595,0.0000185,0.1071221,0.145912,-
0.0444874,-0.0000218,-0.0200635,-0.0146226,-0.0000714,0.0000075,-
0.0000783,0.1072515,-0.0118374,-0.0351772,-0.0000123,-0.05966,0.0685454,-
0.0000223,-0.0000182,-0.0000261,0.1149721,0.0970967,-0.0041579,-0.0000003,-
0.0066672,-0.0134523,-0.0000712,-0.0000066,-0.0000719,0.1137622,-0.5104198,-
0.2581996,0.0000303,-0.2499225,-0.1089535,0.0000199,0.0000279,0.0000205,-
0.0056369,-0.0925183,0.1639194,0.0000392,-0.1119051,0.0348409,0.0000517,-
0.000048,-0.0000095,-0.0917511,-0.0072548,-0.1948991,-0.0000538,0.0555051,-
0.0800769,-0.0000092,0.0000164,0.0000397,-0.0910096,-

0.0491455,0.1660016,0.0000282,0.0141455,0.1259005,0.0000531,-
0.0000055,0.0000319,-0.0952089,-0.6508306,-0.3382924,0.0000462,-0.3661068,-
0.1745914,0.0000162,0.0000293,0.0000061,0.0275291,0.1127207,-0.1527594,-
0.0000724,0.015485,-0.0721267,0.0000062,-0.0000328,0.0000366,-
0.0891309,0.151298,0.0219987,-0.0000043,-0.0010644,-0.0201463,-0.0000302,-
0.000001,-
0.0000329,0.1071566,0.0188141,0.0517559,0.0000303,0.0493616,0.0648186,-
0.0000209,0.0000325,-
0.0000208,0.1137735,0.0532242,0.0734116,0.0000255,0.0980289,0.0779427,-
0.0000283,0.0000213,-0.0000261,0.1072531,0.0894919,0.0011523,0.0000061,-
0.0235541,-0.0329952,-0.0000326,-0.0000028,-0.0000396,0.1149961,0.0108377,-
0.0515807,-0.0000204,-0.0428106,-0.629742,-0.0001688,-0.0000167,-0.0001473,-
0.0056357,-0.0196233,0.0698423,-0.0000019,-0.2061233,-0.0386422,0.0000738,-
0.0000741,0.0000063,-0.0917948,0.0332483,0.0304038,-
0.000022,0.1984513,0.0068945,-0.0000349,0.0000153,0.0000148,-
0.0890611,0.0116123,-0.0165581,0.0000002,-0.0441625,-0.8364513,-0.0002282,-
0.0000043,-0.000223,0.0275511,0.0040178,-0.044901,-0.0000416,-
0.1967427,0.0722823,0.0001005,-0.0000809,0.0000469,-0.0952317,-0.0015851,-
0.0584971,-0.0000433,0.1915343,-0.0862048,-0.000055,0.0000321,0.0000029,-
0.0910056,-0.0024941,-0.0656787,0.0000131,-
0.0410995,0.133812,0.0000226,0.0000151,0.000032,0.1073014,0.0073656,0.07077
12,0.0000492,0.0463324,0.0491937,-0.0000323,0.0000403,-
0.0000341,0.1149561,0.0136625,0.0802656,0.000048,0.0574902,0.1177264,-
0.0000102,0.0000398,-0.0000232,0.1071045,0.0096054,-0.0438552,0.0000183,-
0.0463208,0.0742836,0.0000034,0.000017,-0.0000018,0.1137501,1.4105558,-
0.2218204,-0.0004236,0.0130127,2.5942913,0.0006288,-
0.0003555,0.0006666,0.3089096,-0.8608325,-0.2881756,0.0000832,-0.3184617,-
1.2868034,-0.0001541,0.0000887,-0.0001453,-0.3589523,-
0.8709257,0.67363,0.0003496,0.406261,-1.0838923,-0.0003359,0.0002639,-
0.0004018,-0.373668,2.3890847,-0.5778251,-0.0004804,-
0.34244,1.6159015,0.0008467,-0.0003653,0.0008601,0.3090049,-
0.9177588,0.3514626,0.0002487,0.3210609,-1.2298294,-0.0005693,0.0002365,-
0.0005649,-0.3589607,0.2672122,-0.172352,-0.0000202,-
0.0587129,0.3208331,0.0000175,0.0000078,0.0000546,0.3444294,2.2074495,0.446
8997,-0.0002339,0.6822151,1.7970065,0.0002279,-
0.0001643,0.0002803,0.3089555,-1.4429043,-0.0176935,0.00019,-0.0480756,-
0.7045125,-0.0000719,0.0001738,-0.0000782,-0.3589447,-0.5625888,-0.2278773,-
0.0000187,-0.495374,-1.3919023,-0.0001499,-0.0000858,-0.0002054,-
0.3736968,0.2071159,0.0240365,0.0000287,0.1375968,0.3807331,-
0.0000168,0.0000565,0.0000023,0.3443992,-1.4982691,-0.0442795,0.0000348,-
0.3119162,-0.4566652,-0.0000354,-0.0001199,-0.0000493,-0.3736976,0.4073195,-
0.0220735,-0.0000149,0.0914759,0.1806054,-0.0001079,0.0000564,-
0.0001001,0.3443955\Polar=446.9067805,0.0180639,446.892825,-
0.0762888,0.1165874,93.4008988\PG=C01
[X(C24H15N3O6)]\NImag=0\\0.50472257,0.10594955,0.59404347,-
0.00005752,0.00013413,0.08014146,-0.28447892,-
0.05522986,0.00002640,0.66977273,-0.07956120,-0.15801472,-
0.00000716,0.00134191,0.69078305,0.00001959,-0.00001322,-0.07744945,-
0.00009882,0.00014218,0.20394599,-0.08822505,-0.02232739,0.00002039,-
0.26608376,0.09330199,0.00006681,0.48034977,0.04298793,0.07421023,0.000000
12,0.06896013,-0.17699441,-0.00004189,-

0.09166652,0.61893349,0.00004086,0.00001689,0.03929774,0.00005864,-
0.00004830,-0.07744917,-0.00011064,0.00018725,0.08015024,-0.02028028,-
0.04909587,-0.00001258,0.04248898,0.04538520,0.00000409,-0.13110533,-
0.00885621,0.00000937,0.68429911,-0.04911448,-0.06149620,-
0.00000322,0.10133129,-0.06867569,-0.00004539,-0.03317610,-0.31127145,-
0.00006728,-0.01006770,0.67586867,-0.00001453,-0.00000517,-
0.01282255,0.00002419,-0.00003291,0.00467300,0.00000019,-0.00007455,-
0.07736810,-
0.00010871,0.00016771,0.20390491,0.04244888,0.09793553,0.00002952,-
0.09366841,0.00654633,0.00001997,0.02464323,-0.10817000,-0.00003158,-
0.26976714,-0.06674502,0.00002126,0.66447971,0.03258743,-0.05658676,-
0.00003725,0.00657799,0.01182836,0.00000612,-0.04287126,-0.03869911,-
0.00001616,-0.09120283,-0.17327571,-0.00001318,-
0.01420185,0.43526463,0.00001039,-
0.00005024,0.03928892,0.00001890,0.00000626,-0.01277543,-0.00001050,-
0.00000095,0.03926689,0.00001232,-0.00001796,-0.07740056,-
0.00013589,0.00012044,0.08009833,-0.12879909,-
0.01436921,0.00000596,0.02249222,-0.05683987,-0.00002148,-
0.00868660,0.04238709,0.00001344,-0.10407406,-0.01645948,0.00001949,-
0.24840967,0.10053918,0.00007086,0.68660416,0.01005159,-0.31382538,-
0.00007383,-0.11279424,-0.04854265,0.00000861,0.04241640,-0.07311857,-
0.00002864,0.03950513,0.07825541,0.00001618,0.07608466,-0.19442149,-
0.00005705,0.00885214,0.67336294,0.00001339,-0.00006897,-0.07735138,-
0.00003859,-0.00000450,0.00464462,0.00001198,-0.00002794,-
0.01279760,0.00003578,0.00002574,0.00457868,0.00006323,-0.00005988,-
0.07743266,-0.00010110,0.00014291,0.20385939,-
0.00231208,0.00377708,0.00000038,0.00447125,-
0.00598299,0.00000032,0.00443171,0.01710057,0.00000546,-
0.17479266,0.04939993,0.00003885,-
0.05398864,0.01105341,0.00001824,0.00288934,-0.00818489,-
0.00000138,0.65980684,0.00377814,0.00087873,0.00000140,-0.00751646,-
0.00306656,-0.00000296,0.03559490,-0.04211203,-0.00002396,0.04946328,-
0.13343469,-0.00003868,-0.00754467,0.01630379,0.00000562,-0.00664485,-
0.00150608,-0.00000183,0.03100666,0.68435844,0.00000046,0.00000022,-
0.00487077,-0.00000096,-0.00000286,0.00906214,0.00001223,-
0.00001871,0.00425049,0.00004048,-0.00003972,-
0.07237937,0.00001145,0.00000202,0.00426433,-0.00000187,-
0.00000097,0.00907243,-0.00004288,0.00028754,0.15612996,-0.00044331,-
0.00180402,-0.00000078,0.00029476,-0.00151887,-0.00000012,-0.00522793,-
0.00280152,-0.00000114,-
0.03608624,0.00025974,0.00000363,0.00417674,0.00207003,-0.00000039,-
0.00326640,0.00287397,0.00000249,-0.27072475,-0.05275255,-
0.00000879,0.67374464,0.00064955,-0.00058027,0.00000009,-
0.00092359,0.00162242,0.00000040,0.00239316,-0.00420794,-
0.00000266,0.01268942,0.01949976,0.00000793,0.00665112,-0.00304397,-
0.00000391,0.00018451,0.00077303,-0.00000039,-0.10775867,-0.17034806,-
0.00004695,-0.01473369,0.75501598,-0.00000004,-0.00000019,-0.00005650,-
0.00000002,0.00000061,-0.00025560,0.00000018,-
0.00000162,0.00096430,0.00001262,0.00000882,0.00227790,0.00000373,-
0.00000297,0.00218861,0.00000090,-0.00000008,0.00037680,-0.00004220,-
0.00005296,-0.06663741,-

0.00006210,0.00035777,0.13614129,0.00086688,0.00101972,0.00000036,-
0.00117500,0.00084744,0.00000006,0.00200367,-0.00096880,-0.00000013,-
0.00090230,-0.00593641,-0.00000275,-
0.00321558,0.00118126,0.00000125,0.00041174,-0.00162424,-0.00000083,-
0.05649595,-0.01357326,-0.00000088,-
0.28479659,0.13457501,0.00009892,0.74955756,-0.00008225,-0.00116931,-
0.00000072,0.00087049,0.00025864,0.00000010,0.00013965,-
0.00028789,0.00000040,-0.00584927,-0.00001959,-
0.00000315,0.00208748,0.00054843,-0.00000033,-
0.00175609,0.00152237,0.00000173,0.03212390,0.05858816,0.00002551,0.067463
05,-0.19825672,-0.00008485,0.02499406,0.67766176,0.00000007,-0.00000055,-
0.00004380,0.00000016,0.00000010,0.00023127,0.00000027,-0.00000018,-
0.00058427,-0.00000245,-
0.00000314,0.00660698,0.00000092,0.00000033,0.00031106,-
0.00000120,0.00000094,-
0.00077374,0.00002593,0.00003234,0.00672893,0.00005989,-0.00009310,-
0.06043954,-0.00004404,0.00031481,0.13454634,-
0.00039683,0.00133487,0.00000071,-0.00047625,-0.00158277,-0.00000041,-
0.00067182,0.00075620,0.00000020,0.00185633,-0.00257998,-0.00000196,-
0.00333764,0.00124615,0.00000171,0.00243111,-0.00073055,-0.00000122,-
0.01898149,-0.02752946,-0.00001396,0.03512804,0.02470543,0.00001131,-
0.12860277,0.00805387,0.00001097,0.65967448,0.00126886,0.00074105,0.000000
28,-0.00186806,0.00137883,0.00000032,0.00228070,-0.00234534,-0.00000087,-
0.00229113,-0.00051604,0.00000008,-0.00051600,0.00016877,0.00000011,-
0.00025819,-0.00144783,-0.00000058,-0.02790483,-0.04159458,-
0.00001561,0.07096998,-0.03355737,-0.00003222,-0.04969830,-0.31951878,-
0.00014599,0.03305792,0.69197501,0.00000079,0.00000032,0.00004667,-
0.00000103,0.00000103,0.00001897,0.00000149,-0.00000148,-0.00013177,-
0.00000145,0.00000015,-0.00041659,-0.00000008,0.00000009,-0.00013446,-
0.00000034,-0.00000074,0.00002079,-0.00001541,-0.00001706,-
0.00762496,0.00003822,-0.00002625,0.00756297,-0.00002243,-0.00015162,-
0.06253867,-0.00003435,0.00031945,0.14358170,-0.00120272,-0.00095131,-
0.00000060,0.00256940,-0.00085041,-0.00000025,-
0.00119337,0.00294558,0.00000125,0.00406472,-0.00371969,-
0.00000139,0.00036572,-0.00055437,-0.00000050,-
0.00057042,0.00113129,0.00000108,0.03467783,0.07044273,0.00003618,-
0.06561335,0.00543967,0.00000937,0.02711818,-0.07726950,-0.00004646,-
0.27549719,-0.05430138,-
0.00000833,0.67955284,0.00016361,0.00087006,0.00000005,-0.00072252,-
0.00060484,0.00000078,0.00207067,-0.00153364,-0.00000125,-0.00391750,-
0.00499281,-0.00000612,-0.00159085,0.00134627,0.00000177,0.00105181,-
0.00033099,-0.00000040,0.02548414,-0.03246095,-0.00002598,0.00427859,-
0.00117979,0.00000244,-0.03358856,-0.01958232,-0.00001281,-0.11223718,-
0.17049537,-0.00005026,-0.01535241,0.75575876,0.00000041,0.00000094,-
0.00004117,-0.00000131,-0.00000014,-0.00077420,0.00000197,-
0.00000197,0.00030073,-0.00000234,-0.00000586,0.00663696,-
0.00000160,0.00000147,-0.00058963,0.00000104,-
0.00000092,0.00022760,0.00001250,-
0.00002955,0.00675946,0.00000886,0.00000246,-0.00646243,-0.00002167,-
0.00000807,0.00847085,-0.00004221,-0.00005607,-0.06477429,-
0.00006948,0.00036423,0.13699355,-0.00014638,0.00075485,0.00000075,-

0.00092013,0.00117675,-0.00000024,-
0.00506729,0.00275303,0.00000274,0.00656883,0.03058842,0.00001657,-
0.00420035,0.00279242,0.00000249,0.00227919,-0.00007596,-0.00000106,-
0.12817041,-0.04792567,-0.00001826,0.02676805,-0.03374203,-0.00002139,-
0.01459393,0.02714234,0.00001662,-0.05646499,-0.01352152,-0.00000085,-
0.28785022,0.13596992,0.00010509,0.75695781,-0.00169254,-
0.00086515,0.00000005,0.00386133,-0.00158809,-0.00000094,-
0.00185719,0.00623448,0.00000082,0.01819550,-0.02309030,-0.00001636,-
0.00241702,-0.00519564,-0.00000333,-0.00064241,-
0.00036162,0.00000025,0.00700186,-0.31160735,-0.00014141,-0.07465839,-
0.01903420,-0.00000798,0.02656517,-0.05208735,-
0.00002915,0.03189280,0.05842217,0.00002657,0.06990704,-0.20024857,-
0.00008997,0.01738360,0.67334860,-0.00000084,-0.00000100,-
0.00005392,0.00000214,-0.00000087,0.00037775,-
0.00000009,0.00000223,0.00221123,0.00000793,-
0.00001705,0.00229479,0.00000082,-0.00000476,0.00094220,-
0.00000142,0.00000027,-0.00025722,0.00001371,-0.00013631,-0.06655188,-
0.00004526,-0.00001247,0.00875702,0.00001605,-0.00002930,-
0.00653974,0.00002509,0.00003050,0.00720945,0.00006771,-0.00009652,-
0.06084295,-0.00006324,0.00030745,0.13682679,-0.00002101,-0.00008411,-
0.00000004,-0.00011531,0.00009083,0.00000012,0.00014213,-0.00041869,-
0.00000016,-0.00135443,-0.00031366,-0.00000028,-
0.00156133,0.00331510,0.00000208,-0.00002065,0.00015111,-
0.00000004,0.00245127,-0.00187076,-
0.00000085,0.00126893,0.00045896,0.00000096,0.00028938,0.00075930,0.000000
34,-0.00193464,0.00367884,0.00000346,-0.01771184,-0.01212501,-0.00000446,-
0.29134998,-0.11585555,-0.00003756,0.30983666,-0.00001817,0.00086287,-
0.00000012,-0.00038157,-0.00025370,-
0.00000011,0.00037663,0.00017423,0.00000013,-
0.00223228,0.00045006,0.00000251,-0.00026993,-
0.00031500,0.00000046,0.00140840,0.00022437,0.00000033,-0.02986815,-
0.00925507,-0.00000385,0.00153267,-0.00591212,-0.00000689,0.00056359,-
0.00071752,-0.00000031,0.00306155,-0.00180011,-
0.00000584,0.01789018,0.01225926,0.00000295,-0.11372026,-0.11564456,-
0.00003008,0.12287981,0.11862577,-
0.00000012,0.00000029,0.00031197,0.00000019,-0.00000029,0.00024265,-
0.00000002,0.00000086,-0.00016191,-0.00000083,0.00000176,-
0.00344027,0.00000040,-0.00000081,-0.00004794,0.00000058,0.00000071,-
0.00044879,-0.00001801,-0.00000626,0.00351880,0.00000147,-
0.00000685,0.00578099,0.00000015,-0.00000035,-0.00032218,0.00000282,-
0.00000596,0.00771258,0.00001266,0.00000640,0.00349047,-0.00003562,-
0.00003048,-
0.03885329,0.00003724,0.00003743,0.02680383,0.00076764,0.00030953,-
0.00000019,-0.00036989,0.00126379,0.00000064,-0.00152196,-
0.00032896,0.00000049,0.00109926,-0.00124186,-
0.00000113,0.00019086,0.00039881,0.00000012,-0.00013027,-0.00038580,-
0.00000008,0.00368763,-0.02931343,-0.00001703,-0.05994673,-0.01641227,-
0.00000637,0.00556715,0.02774507,0.00001574,-
0.00428783,0.00208586,0.00000231,-0.00104124,-0.00000141,0.00000005,-
0.00556450,-0.00133844,0.00000035,0.00012246,-0.00167492,-
0.00000093,0.05968985,0.00024452,0.00007478,-

0.00000029,0.00000229,0.00056545,0.00000047,0.00327645,-0.00024581,-
0.00000017,0.00077604,-0.00196206,0.00000102,-
0.00040024,0.00012932,0.00000034,0.00008416,-0.00023859,-0.00000024,-
0.00133019,-0.01071911,-0.00000785,-0.01853823,-0.34709033,-0.00017688,-
0.00237427,-0.01106362,-0.00000807,0.00274559,0.00049095,-
0.00000430,0.00017913,0.00061317,0.00000052,-0.00239660,0.00085892,-
0.00000243,0.00017691,-0.00050703,-
0.00000071,0.01766345,0.36895932,0.00000013,-
0.00000022,0.00031336,0.00000036,0.00000037,-
0.00045177,0.00000141,0.00000014,-0.00006751,-0.00000036,0.00000142,-
0.00351825,0.00000035,0.00000019,-0.00016131,-
0.00000007,0.00000005,0.00024719,0.00000021,-0.00000558,0.00351073,-
0.00000850,-0.00017663,-0.03866347,-0.00000156,-
0.00001142,0.00341260,0.00000278,-
0.00000430,0.00767610,0.00000006,0.00000050,-0.00037121,-0.00000030,-
0.00000270,0.00572773,0.00000021,-
0.00000058,0.00068397,0.00000609,0.00019539,0.02682112,-
0.00004059,0.00012081,-0.00000002,0.00004458,-0.00016756,0.00000006,-
0.00005356,0.00026974,0.00000005,-0.00014842,-0.00131751,-0.00000055,-
0.00061946,0.00028124,0.00000013,0.00008491,-0.00014744,0.00000009,-
0.00205570,0.00383505,0.00000299,-0.01811316,-0.01256472,-0.00000535,-
0.29033594,-0.11620789,-0.00004411,0.00248169,-0.00027593,-
0.00000011,0.00145268,0.00051597,0.00000062,0.00027347,0.00076323,0.000000
40,-0.00006206,-0.00004606,-0.00000012,0.00106150,0.00011470,-
0.00000041,0.30787928,-0.00004334,-0.00005767,-
0.00000001,0.00003997,0.00002093,0.00000005,-0.00008232,-0.00001053,-
0.00000011,0.00054057,0.00078458,0.00000030,0.00035211,-0.00017855,-
0.00000001,0.00005535,0.00009925,0.00000006,0.00311903,-0.00188852,-
0.00000592,0.01787988,0.01234285,0.00000364,-0.11386472,-0.11721880,-
0.00003583,-0.02956029,-0.00940121,-0.00000428,0.00147845,-0.00595504,-
0.00000709,0.00058836,-0.00064833,-0.00000025,-
0.00003431,0.00009326,0.00000074,-
0.00044750,0.00086918,0.00000304,0.12240650,0.12042887,-0.00000005,-
0.00000003,-0.00002958,0.,-0.00000002,0.00002133,-
0.00000012,0.00000005,0.00006806,0.00000040,0.00000043,0.00028605,0.000000
17,-0.00000003,-0.00030379,-0.00000003,0.00000012,-0.00000282,0.00000282,-
0.00000592,0.00771276,0.00001249,0.00000655,0.00334077,-0.00004298,-
0.00003588,-0.03788074,-0.00001709,-0.00000696,0.00276096,0.00000131,-
0.00000691,0.00583695,0.00000042,-0.00000028,-0.00028281,-
0.00000018,0.00000075,-0.00118378,-0.00000072,0.00000288,-
0.00426835,0.00004512,0.00004365,0.02627578,-0.00009321,-
0.00006149,0.00000002,0.00014156,0.00006267,-0.00000005,-
0.00047723,0.00042032,0.00000022,0.00094369,0.00095509,0.00000047,-
0.00010744,-0.00009642,-0.00000006,0.00008550,0.00005148,0.,-
0.00439922,0.00213702,0.00000270,-0.00102827,0.00002908,0.00000011,-
0.00557758,-0.00130895,0.00000043,0.00350070,-0.02927733,-0.00001714,-
0.06041513,-0.01356919,-
0.00000574,0.00578676,0.02757785,0.00001594,0.00103542,-0.00049086,-
0.00000090,0.00008632,0.00004086,-0.00000012,0.00014408,-0.00167154,-
0.00000091,0.05931846,0.00008207,-0.00000577,0.00000004,-
0.00010726,0.00002873,-0.00000005,0.00029061,-0.00026297,0.00000007,-

0.00076094,-0.00028306,-0.00000028,0.00020149,0.00002623,-0.00000002,-
0.00011511,-0.00000655,-0.00000008,0.00264902,0.00052729,-
0.00000456,0.00021429,0.00061326,0.00000053,-0.00232885,0.00084790,-
0.00000254,-0.00105329,-0.01083912,-0.00000854,-0.01577527,-0.34577952,-
0.00017786,-0.00222842,-0.01072294,-
0.00000804,0.00007708,0.00086946,0.00000305,0.00002840,-
0.00006982,0.00000060,0.00013104,-0.00043899,-
0.00000070,0.01571447,0.36652661,0.00000005,0.00000001,-0.00003263,-
0.00000001,0.,0.00000290,0.00000013,-0.00000011,-0.00030701,-0.00000045,-
0.00000038,0.00024067,0.00000025,-0.00000005,0.00007514,-0.00000010,-
0.00000004,0.00002574,0.00000277,-
0.00000462,0.00777194,0.00000030,0.00000063,-0.00034463,-0.00000007,-
0.00000259,0.00560235,-0.00000100,-0.00000527,0.00381650,-0.00000670,-
0.00017823,-0.03909957,-0.00000169,-0.00001105,0.00335993,-
0.00000068,0.00000307,-0.00432984,-0.00000008,0.00000058,-
0.00109609,0.00000014,-
0.00000047,0.00072727,0.00000593,0.00019697,0.02747184,-0.00270861,-
0.02196040,-0.00000553,0.00602571,0.00483489,0.00000235,-0.00318689,-
0.00326788,-0.00000156,0.00467534,0.00741158,0.00000323,-0.05325384,-
0.00223428,0.00001170,-0.18635752,-0.04269921,0.00000921,-0.00081029,-
0.00052594,-0.00000012,0.00009829,0.00023890,0.00000008,-
0.00009215,0.00004062,0.,-0.00027657,0.00008357,0.00000004,0.00007199,-
0.00004387,-0.00000002,-0.00007062,0.00003514,0.00000013,0.00006245,-
0.00005377,-0.00000009,0.00009495,-0.00002772,0.00000002,-
0.00003890,0.00001352,0.00000004,-0.00001024,0.00001622,0.,0.65102192,-
0.04057912,-0.03490320,-0.00000381,0.00636922,-0.00463293,-0.00000555,-
0.00326899,0.00174763,0.00000269,0.00589723,-0.00325681,-
0.00000569,0.01629163,0.01565319,0.00000040,-0.04266977,-0.12152211,-
0.00000501,0.00077406,0.00111798,0.00000027,0.00026044,-0.00036363,-
0.00000018,0.00009503,0.00014361,0.00000011,-0.00003384,0.00001055,0.,-
0.00000354,0.00000545,0.,-0.00009674,-
0.00004957,0.,0.00005088,0.00000440,0.00000002,0.00006752,0.00001647,0.0000
0001,0.00003135,-0.00002483,-0.00000004,0.00000248,-0.00000272,-0.00000001,-
0.02611821,0.69238871,-0.00001104,-0.00000719,0.00425342,0.00000293,-
0.00000516,0.00907073,-0.00000147,0.00000287,-0.00486917,0.00000282,-
0.00000558,0.00905987,0.00001775,0.00000399,0.00426002,0.00000870,-
0.00000569,-0.07233799,0.00000030,0.00000045,0.00008833,0.00000007,-
0.00000009,-0.00014447,0.00000014,0.00000013,-0.00002947,0.00000009,-
0.00000001,-0.00000681,-0.00000007,-0.00000009,0.00005686,0.00000005,-
0.00000010,0.00001900,0.00000003,0.,-0.00003126,0.,0.,-0.00001427,-
0.00000006,-0.00000007,0.00003559,0.,0.00000004,0.00000553,-
0.00009441,0.00013994,0.15621884,-
0.00509724,0.00294782,0.00000170,0.00060936,0.00160747,0.00000029,-
0.00028019,0.00176917,0.00000062,-0.00360406,-
0.00231211,0.00000037,0.00301970,-0.00281329,-0.00000134,-
0.03681778,0.00680296,0.00000884,0.00003685,-0.00030823,-0.00000016,-
0.00023193,0.00009718,0.00000009,0.00004024,-0.00012948,-
0.00000006,0.00003155,0.00007132,0.00000003,-
0.00006316,0.00001262,0.00000005,0.00010378,0.00003909,-
0.00000002,0.00000143,-0.00000040,-0.00000002,-0.00000220,0.00000133,-
0.00000002,-0.00000778,0.00000296,-0.00000001,0.00000785,-0.00000189,-

0.00000001,-0.24842566,0.06209935,0.00004626,0.67998163,-0.00225664,-
0.00426592,-0.00000099,0.00103930,0.00130677,0.00000033,-0.00067642,-
0.00073347,-0.00000009,0.00037212,0.00109474,0.00000033,-0.00742064,-
0.00185406,0.00000041,-0.00554344,0.02025385,0.00000537,-0.00028806,-
0.00029302,-0.00000001,-0.00004779,0.00019656,0.00000012,-0.00001995,-
0.00000943,-0.00000001,-0.00014744,0.00006763,0.00000004,0.00003862,-
0.00001450,-0.00000002,-0.00014175,-0.00002254,0.00000003,-0.00000924,-
0.00008495,-0.00000003,0.00005377,-0.00000146,0.,-0.00002280,0.00000903,0.,-
0.00001815,0.00001319,0.,0.11701131,-0.19178597,-
0.00005185,0.02749566,0.75118620,-0.00000001,-
0.00000213,0.00093282,0.00000034,0.00000023,-0.00025754,-0.00000028,-
0.00000042,-0.00005361,0.00000096,0.00000059,0.00037578,-0.00000199,-
0.00000063,0.00221158,0.00000581,0.00000373,0.00230032,-
0.00000015,0.00000002,-0.00015019,0.00000002,0.00000008,-0.00004855,0.,-
0.00000002,0.00004007,-0.00000005,0.00000002,-0.00001692,0.00000003,-
0.00000003,0.00003446,-0.00000006,0.,-0.00002040,0.00000001,-0.00000002,-
0.00000893,0.00000002,0.,-0.00000596,0.,0.,-0.00000129,0.,0.,-
0.00000251,0.00006007,-0.00004221,-0.06661381,-
0.00008809,0.00014561,0.13668228,-0.00501968,-0.00135374,0.00000102,-
0.00155749,-0.00116881,-0.00000012,-0.00004665,-0.00087656,-
0.00000012,0.00234298,-0.00025771,-0.00000085,-0.00428356,-
0.00294388,0.00000029,-0.00000632,-0.03353297,-0.00000782,-
0.00006518,0.00009490,0.00000007,0.00011138,0.00012661,0.00000003,0.000009
52,0.00015104,0.00000007,-0.00017731,-
0.00001012,0.00000003,0.00017069,0.00006017,-0.00000002,-0.00058681,-
0.00023383,-0.00000015,-
0.00018521,0.00000105,0.00000028,0.00009474,0.00001341,0.00000003,-
0.00000602,0.00000609,0.00000002,-0.00006194,0.00003154,0.00000004,-
0.12591021,0.02418667,0.00001661,0.03920149,0.02657173,0.00000118,0.747295
18,0.00322895,0.00613518,0.00000074,-0.00385521,-
0.00093259,0.00000032,0.00157476,-0.00097531,-0.00000062,0.00032848,-
0.00042715,-0.00000012,0.00227418,-0.00515238,-0.00000197,-0.02108680,-
0.01659566,-0.00000068,-0.00004050,-0.00005488,-0.00000005,-0.00005679,-
0.00003075,0.,-0.00001804,-0.00005221,-
0.00000004,0.00003950,0.00001131,0.00000002,-0.00007940,-0.00006383,-
0.00000003,0.00032651,0.00012793,0.00000009,0.00019994,-0.00009150,-
0.00000021,-0.00005671,-0.00000815,0.,0.00000018,-0.00000188,0.,0.00001657,-
0.00001114,0.,-0.03085751,-0.31506331,-0.00005573,0.06755052,-0.03163383,-
0.00002176,-0.02792439,0.68084758,0.00000201,0.00000113,0.00218588,-
0.00000065,-0.00000022,0.00037780,0.00000042,0.00000006,-0.00005568,-
0.00000058,-0.00000007,-0.00025618,0.00000197,-0.00000059,0.00097467,-
0.00000514,0.00000088,0.00227666,0.00000007,-0.00000003,0.00001855,-
0.00000005,-0.00000002,-0.00002203,0.,-0.00000006,0.00001305,0.00000005,0.,-
0.00000708,0.,0.,-0.00000018,0.00000004,-0.00000002,0.00006271,-
0.00000002,0.00000008,-0.00010703,-0.00000002,0.,0.00000051,0.,0.,-
0.00000241,0.,0.,-0.00000528,0.00000327,-0.00006487,-0.06663866,0.00001129,-
0.00001446,0.00879455,-0.00011208,0.00013667,0.13628343,-0.00180409,-
0.00289142,-0.00000041,0.00270793,0.00042888,-0.00000050,-
0.00106572,0.00119851,0.00000044,-0.00084454,-
0.00106121,0.00000007,0.00065014,0.00064132,-
0.00000005,0.00485985,0.00250782,0.00000097,0.00007979,-0.00000428,-

0.00000004,-0.00006799,-0.00003269,0.00000002,0.00001788,-0.00006735,-
0.00000004,0.00005598,0.00002621,-0.00000001,-
0.00006429,0.00000329,0.00000004,0.00016506,0.00004640,0.00000003,0.000071
36,0.00000401,-0.00000010,-0.00000762,-0.00000271,-0.00000001,0.00000104,-
0.00000258,0.,0.00001698,-0.00000730,-0.00000002,0.02171040,-0.07735195,-
0.00002189,-0.06590065,0.00237108,0.00001100,-0.30749063,-
0.05431202,0.00003065,0.67476847,-0.00199223,-
0.00085790,0.00000015,0.00029732,-0.00077432,-
0.00000029,0.00009647,0.00076339,0.00000036,-0.00103870,-
0.00007260,0.00000009,0.00175057,0.00107043,-0.00000011,0.00260039,-
0.00578836,-0.00000359,0.00004013,-0.00000205,0.00000005,-0.00000412,-
0.00001175,-0.00000002,0.00000916,0.00000060,0.,0.00007182,-0.00004424,-
0.00000001,0.00001380,0.00005906,0.00000001,-0.00008958,-0.00006081,-
0.00000006,-0.00010669,0.00007346,0.00000012,-
0.00000740,0.00000388,0.,0.00000576,0.00000086,0.,0.00000273,-0.00000073,0.,-
0.03164939,-0.01968943,-0.00000085,0.00295863,-0.00087995,0.00000087,-
0.12147326,-0.17537691,-
0.00000650,0.01864594,0.75335349,0.00000010,0.00000034,0.00031243,-
0.00000068,-0.00000001,-0.00077287,0.00000023,-0.00000009,-
0.00004371,0.00000003,0.00000024,0.00023139,0.00000006,0.00000019,-
0.00058272,0.00000086,-0.00000353,0.00660673,0.,0.,0.00005966,0.00000004,-
0.00000002,0.00003518,0.00000002,0.00000005,-0.00001142,0.,-
0.00000002,0.00000032,0.00000002,-0.00000002,0.00000794,-0.00000007,-
0.00000001,-0.00000038,-0.00000005,0.00000002,-0.00001608,0.,0.,-0.00000366,-
0.00000002,0.,-0.00000368,0.,0.00000001,-0.00000487,-
0.00001031,0.00000680,0.00675632,0.00001102,0.00000105,-
0.00646948,0.00001396,-0.00001814,-0.06041273,-
0.00009044,0.00014820,0.13441151,-0.00102570,-0.00082188,-0.00000012,-
0.00004872,0.00169117,0.00000053,-0.00067107,-0.00111073,-
0.00000016,0.00240983,0.00020174,-0.00000066,-0.00324234,-
0.00079659,0.00000062,0.00218713,0.00210138,0.00000005,-
0.00026851,0.00007006,0.00000011,0.00003321,0.00014648,0.00000004,0.000009
47,0.00006649,0.00000004,-0.00031253,0.00014015,0.00000011,0.00003801,-
0.00007412,-0.00000007,-0.00017072,-0.00001989,0.00000005,0.00005801,-
0.00012317,-0.00000008,0.00009148,-0.00000157,0.00000001,-0.00002270,-
0.00000148,0.,-0.00001908,0.00001951,0.00000001,-
0.01195222,0.02388387,0.00000686,0.02165219,-0.03166628,-0.00001032,-
0.05758400,-0.01723202,0.00000689,-
0.25419635,0.12183614,0.00006337,0.65521330,-0.00258035,-0.00213904,-
0.00000016,0.00216311,0.00103327,-0.00000007,-
0.00117650,0.00101486,0.00000047,-0.00008323,-0.00150612,-
0.00000041,0.00072880,0.00022302,-0.00000002,0.00238990,-0.00084929,-
0.00000042,-0.00006280,0.00001985,0.00000002,-
0.00006942,0.00006698,0.00000005,0.00003280,-0.00003548,-0.00000002,-
0.00011139,0.00010807,0.00000007,-0.00003643,-
0.00003092,0.,0.00002166,0.00000590,0.00000002,0.00006032,-0.00005969,-
0.00000009,0.00004689,-0.00000125,0.,-0.00001035,-0.00000269,0.,-
0.00000043,0.00000483,0.,0.02350455,-0.04861067,-0.00001450,-0.07709624,-
0.01978903,0.00000703,0.02903118,0.05938535,0.00000755,0.06407743,-
0.19444219,-0.00004314,-0.03046012,0.69608426,-0.00000044,-0.00000031,-
0.00013405,0.00000051,0.00000001,0.00002089,-

0.00000015,0.00000040,0.00004651,-0.00000040,-
0.00000043,0.00001897,0.00000066,0.00000021,-0.00013193,0.00000010,-
0.00000047,-0.00041763,0.00000004,-0.00000003,-0.00001317,-0.00000003,0.,-
0.00001532,-0.00000004,-0.00000006,0.00000515,0.,0.,-
0.00000056,0.,0.00000004,0.00000027,0.00000004,0.,-
0.00000587,0.00000003,0.,0.00001182,0.,0.,0.00000286,0.00000004,0.00000002,0.
00000031,0.,-0.00000003,0.00000035,0.00000648,-0.00001407,-0.00761652,-
0.00002120,-
0.00000113,0.00723990,0.00001862,0.00001544,0.00748043,0.00004939,-
0.00005324,-0.06261220,-
0.00009743,0.00014026,0.14366610,0.00203116,0.00062649,-0.00000020,-
0.00133514,-0.00060879,0.00000015,0.00069253,-0.00125836,-
0.00000052,0.00087186,0.00170379,0.00000031,-0.00362179,-
0.00066743,0.00000049,0.00059569,0.00594119,0.00000256,-0.00011294,-
0.00006327,-0.00000004,0.00005766,0.00001489,0.,-0.00003701,0.00003085,0.,-
0.00001027,-0.00003196,0.00000003,0.00002468,-0.00001427,-0.00000005,-
0.00000885,0.00001276,0.00000001,0.00000237,-0.00000943,0.,-0.00001716,-
0.00000425,0.00000001,-0.00000219,0.00000282,0.00000002,-
0.00000114,0.00000044,0.00000002,-0.05698234,0.02764606,0.00001800,-
0.31142146,-0.12234744,0.00001364,-0.02158331,-0.02993151,-
0.00000472,0.03995274,0.02619157,0.00000099,-
0.12458296,0.02514024,0.00001672,0.74982787,-0.00040850,-
0.00032347,0.00000009,-0.00068775,0.00043521,0.00000010,-0.00014386,-
0.00102501,-0.00000019,0.00183200,0.00109457,-0.00000015,-
0.00154452,0.00089461,0.00000062,0.00573635,-0.00149981,-0.00000313,-
0.00000760,0.00015497,0.00000010,0.00012016,-0.00002764,-0.00000005,-
0.00001350,0.00009809,0.00000006,-0.00007217,-0.00002695,-
0.00000005,0.00006532,-0.00000631,0.,-0.00015554,-0.00003320,0.00000002,-
0.00002498,-
0.00000429,0.00000003,0.00002298,0.00000503,0.00000002,0.00000154,-
0.00000042,0.,-0.00001526,0.00000733,0.,-0.01730750,0.05943149,0.00001660,-
0.05623959,-0.17648147,-0.00001893,-0.03109022,-0.04508910,-
0.00000404,0.06992652,-0.03256558,-0.00002236,-0.03279237,-0.32126020,-
0.00005713,-0.02543638,0.68475717,-0.00000040,0.00000003,-0.00059286,-
0.00000004,0.00000010,0.00022730,-0.00000010,-0.00000010,-
0.00004115,0.00000016,0.00000035,-
0.00077478,0.00000006,0.00000018,0.00029913,0.00000266,-
0.00000297,0.00663650,-0.00000001,0.00000008,-0.00002420,0.00000002,-
0.00000003,0.00003839,0.00000002,0.00000005,-0.00000739,0.,0.,0.00000537,-
0.00000001,-0.00000002,-0.00001156,-0.00000004,-0.00000001,0.00001291,-
0.00000002,0.,-0.00000291,0.,0.,-0.00000276,-0.00000001,0.,-0.00000372,0.,0.,-
0.00000164,0.00000690,0.00000802,0.00667923,0.00003042,-0.00000719,-
0.06080833,-0.00000504,-0.00000424,-0.00654493,0.00001150,-
0.00001462,0.00850815,0.00000270,-0.00006717,-0.06476056,-
0.00011543,0.00013974,0.13711262,-0.00198215,0.00047758,0.00000053,-
0.00051333,-0.00112301,-0.00000020,0.00068431,-0.00038911,-0.00000029,-
0.00009252,0.00036737,0.00000022,0.00018102,-0.00039740,-
0.00000018,0.00103831,0.00088298,-0.00000062,0.00008829,-0.00007580,-
0.00000005,-0.00000709,-0.00005284,-0.00000002,-0.00002090,-0.00002063,-
0.00000002,0.00008806,-0.00005071,-0.00000003,-
0.00000635,0.00000704,0.,0.00009812,0.00004419,0.,0.00001001,0.00001250,0.,-

0.00002970,-0.00000185,0.,0.00000524,0.00000074,0.,0.00000898,-
0.00000724,0.,0.00762218,0.02687569,0.00000587,-0.06018852,-0.01967239,-
0.00000080,-0.00498035,0.00207558,0.00000233,-
0.00103830,0.00020315,0.00000017,-0.00475469,-
0.00143540,0.00000182,0.00226955,-0.02943767,-0.00000704,0.05996352,-
0.00309505,0.00008453,0.00000050,0.00013826,0.00071775,0.00000029,-
0.00032647,0.00015859,0.00000005,-0.00010493,-0.00027855,-
0.00000011,0.00040119,0.00012965,-0.00000004,-0.00111692,-
0.00203247,0.00000053,0.00001843,0.00002294,-0.00000001,-
0.00000042,0.00000542,0.,0.00000744,0.00000734,0.,-
0.00000974,0.00000506,0.00000001,0.00000414,0.00000271,0.,-0.00002764,-
0.00001209,0.,-
0.00001000,0.00000264,0.00000001,0.00000638,0.00000484,0.,0.00000022,-
0.00000005,0.,-0.00000306,0.00000139,0.,-0.00107002,-0.01423881,-0.00000420,-
0.01756799,-0.34699492,-0.00007179,0.00314526,0.00033646,-
0.00000189,0.00000985,0.00059904,0.00000023,-0.00205493,0.00101322,-
0.00000127,0.00060822,-0.00771208,-0.00000288,0.02138576,0.36866639,-
0.00000060,0.,-0.00002388,0.00000012,0.00000050,-0.00044675,-
0.00000013,0.00000005,0.00031211,-0.00000002,-
0.00000027,0.00024236,0.00000024,0.00000009,-0.00016255,-
0.00000122,0.00000003,-0.00344717,0.00000001,0.,-0.00001377,0.,0.00000001,-
0.00000556,0.,0.,-0.00000322,-0.00000002,0.,0.00000313,0.,0.00000001,-
0.00000342,-0.00000002,-
0.00000001,0.00000029,0.,0.,0.00000161,0.,0.,0.00000517,0.,0.,0.00000179,0.,0.,0.
00000281,-0.00000066,-0.00000904,0.00349569,-0.00000053,-0.00007124,-
0.03884440,0.00000262,-0.00000169,0.00577877,0.00000013,0.00000020,-
0.00031983,0.00000167,-
0.00000139,0.00771287,0.00000032,0.00000231,0.00347927,-
0.00000066,0.00007957,0.02678659,-0.00005267,-
0.00023266,0.,0.00004429,0.00013935,-0.00000005,-0.00003663,-
0.00011472,0.00000003,0.00007636,0.00014492,-0.00000005,-0.00062500,-
0.00020587,0.00000006,-0.00005972,0.00134094,0.00000030,-0.00003832,-
0.00002847,0.00000007,-0.00000501,0.00002131,0.00000001,-0.00000262,-
0.00000125,0.00000001,-0.00001680,0.00000824,-0.00000002,0.00000068,-
0.00000372,0.,-0.00000561,0.00000010,0.,0.00000254,-0.00000845,-
0.00000001,0.00000391,-0.00000070,0.,-0.00000273,0.00000102,-0.00000001,-
0.00000086,0.00000126,-0.00000001,-0.00277276,-0.00358740,0.00000104,-
0.01760099,0.01574225,0.00000761,0.00010832,-0.00086344,-
0.00000030,0.00080355,-0.00135706,0.00000056,0.00589978,-0.00030416,-
0.00000047,-0.26149429,0.13194388,0.00007284,0.00107954,-0.00011873,-
0.00000101,0.27592181,0.00006491,-0.00002819,-0.00000004,-
0.00002754,0.00003485,0.00000004,0.00002872,-0.00006251,-0.00000004,-
0.00002507,0.00009400,0.00000009,-0.00033522,-0.00011561,0.00000008,-
0.00037558,0.00072079,0.00000021,-0.00001557,-0.00001913,-0.00000004,-
0.00000135,0.00000876,0.,-0.00000240,0.00000028,0.,-0.00000038,-
0.00000168,0.00000002,0.00000253,-0.00000015,0.,-0.00000595,-0.00000091,0.,-
0.00000256,-0.00000241,0.,-0.00000028,0.00000006,0.,-
0.00000085,0.00000092,0.,-0.00000166,0.00000051,0.,-0.00307831,-0.00109587,-
0.00000173,-0.01409985,0.01264709,0.00000479,-0.00068589,-
0.00051314,0.00000007,-0.00237703,-0.00554944,-0.00000236,0.02791101,-
0.01307292,-0.00000911,0.12978518,-0.14455015,-

0.00004951,0.00045160,0.00080857,0.00000121,-
0.14085915,0.14970111,0.,0.,0.00007541,-
0.00000002,0.,0.00002580,0.,0.00000001,-0.00003267,-0.00000004,-
0.00000003,0.00000284,-0.00000002,0.00000012,-0.00030696,-0.00000005,-
0.00000017,0.00024091,0.00000002,-0.00000002,0.00003586,0.,0.,-0.00000134,-
0.00000002,0.,-0.00000381,0.,0.,0.00000037,0.,0.00000002,-0.00000352,0.,0.,-
0.00000266,0.,0.,0.00000486,0.,0.,0.00000177,0.00000002,0.,0.00000113,0.,0.,0.00
000117,0.00000107,-0.00000153,0.00777201,0.00000008,-
0.00000050,0.00338100,-0.00000021,0.00000014,-0.00034653,0.00000022,-
0.00000250,0.00560508,0.00000650,-0.00000410,0.00384591,0.00007258,-
0.00004999,-0.03910894,-0.00000082,0.00000129,-0.00432511,-
0.00007970,0.00005566,0.02737320,0.00014661,0.00042702,0.00000013,-
0.00008113,-0.00011163,0.,0.00000635,0.00019233,0.00000014,-0.00021570,-
0.00009409,-0.00000014,-0.00199895,-0.00312682,-0.00000048,-
0.00101105,0.00044136,-0.00000031,0.00006425,-
0.00005732,0.00000001,0.00000425,-
0.00000170,0.00000002,0.00000520,0.00002406,0.,0.00006422,-0.00007288,-
0.00000004,0.00008834,0.00010200,0.00000004,-0.00020782,-0.00018090,-
0.00000020,-
0.00026329,0.00019688,0.00000042,0.00000987,0.00000925,0.,0.00000527,0.0000
0295,0.,-0.00000789,0.00000201,-0.00000001,0.00613823,-0.00002954,-
0.00000032,0.00085537,-0.00131251,0.00000053,-
0.25993644,0.13407331,0.00006925,-0.01792601,0.01594810,0.00000754,-
0.00279198,-0.00361001,0.00000094,0.00013335,-0.00084956,-
0.00000029,0.00029901,-0.00027854,0.,-0.00006052,0.00004380,-
0.00000018,0.27630066,-0.00037198,0.00017385,0.00000013,0.00035924,-
0.00028871,-0.00000018,0.00012925,0.00083648,0.00000019,-
0.00135565,0.00041664,0.00000049,0.00046472,0.00004177,-
0.00000028,0.00242866,0.00005978,0.00000041,0.00004682,0.00000519,0.,-
0.00000757,-0.00008494,-0.00000005,0.00000629,-0.00000863,0.,0.00011006,-
0.00005893,-0.00000005,-0.00000447,0.00005760,0.00000005,0.00001649,-
0.00006466,-0.00000005,-0.00014090,0.00023087,0.00000011,-
0.00001337,0.00000298,0.,0.00000952,-0.00000242,0.,0.00001976,-0.00000864,-
0.00000002,0.02793900,-0.01295855,-0.00000879,-0.00237224,-0.00557779,-
0.00000232,0.13203353,-0.14697052,-0.00004857,-
0.01421038,0.01240708,0.00000459,-0.00295108,-0.00100211,-0.00000154,-
0.00067726,-0.00055076,0.00000008,0.00157236,-0.00068387,-
0.00000061,0.00005631,0.00007735,0.00000027,-0.14272837,0.15228564,-
0.00000022,-0.00000014,-0.00016007,0.00000025,-
0.00000011,0.00024784,0.00000006,0.00000010,0.00031428,-
0.00000051,0.00000012,-0.00045389,0.00000056,0.00000070,-
0.00006040,0.00000032,0.00000086,-0.00349409,-0.00000007,0.,-0.00003379,-
0.00000002,-0.00000003,-0.00000832,-0.00000001,-0.00000003,-
0.00000315,0.00000002,0.00000002,0.00001226,-0.00000012,-0.00000002,-
0.00001590,0.00000031,0.00000019,-0.00010714,0.00000031,-
0.00000017,0.00024584,-0.00000002,0.,0.00000137,0.,0.,0.00000472,0.00000003,-
0.00000002,0.00000659,0.00000612,-0.00000422,0.00351535,0.00000034,-
0.00000256,0.00573060,0.00006845,-0.00004865,-0.03868957,0.00000050,-
0.00000057,0.00343790,0.00000112,-0.00000144,0.00767625,-
0.00000029,0.00000008,-0.00037329,0.00000044,-0.00000029,0.00068433,-
0.00000018,0.00000029,-0.00109567,-0.00007652,0.00005466,0.02679007,-

0.00056260,-0.00038441,-0.00000005,0.00013551,-0.00008458,-0.00000007,-
0.00008687,0.00007008,0.00000004,0.00008197,-0.00006148,-0.00000004,-
0.00010201,0.00011037,0.00000005,0.00088724,-0.00113955,-0.00000037,-
0.00001159,-0.00000190,0.,0.00000965,0.00001801,0.,0.00000061,0.00001535,0.,-
0.00001942,0.00000206,0.,0.00001685,-0.00000493,0.,-0.00006426,-
0.00000819,0.,-0.00001013,-0.00001887,0.00000001,0.00000939,0.00000180,0.,-
0.00000075,0.00000197,0.,-0.00001336,0.00000520,0.,-0.00494295,-
0.00145206,0.00000185,-0.00100201,0.00014942,0.00000017,0.00243017,-
0.02974376,-0.00000709,-0.06087221,-0.01852139,-
0.00000059,0.00660266,0.02711389,0.00000588,-
0.00496807,0.00222711,0.00000247,0.00008898,-0.00005332,-
0.00000024,0.00037384,0.00153881,0.00000043,0.00105004,0.00044067,-
0.00000081,0.06133413,-0.00031423,-
0.00023534,0.00000002,0.00011850,0.00004893,0.00000007,-0.00009404,-
0.00001125,-0.00000002,0.00014615,-0.00001658,0.00000004,-
0.00024209,0.00003771,0.,0.00071953,-0.00025210,-0.00000019,-
0.00002174,0.00000035,-
0.00000004,0.00000242,0.00001443,0.00000001,0.00000095,0.00000599,0.,-
0.00002472,0.00001034,0.00000003,0.00000567,-0.00000309,-0.00000001,-
0.00002748,-0.00000610,0.,0.,-0.00000896,0.,0.00000826,0.00000033,0.,-
0.00000196,0.00000020,0.,-0.00000391,0.00000324,0.,-0.00217154,0.00099421,-
0.00000133,-0.00002371,0.00061654,0.00000021,0.00072505,-0.00818730,-
0.00000294,-0.01626077,-0.34685080,-0.00007320,-0.00208689,-0.01346307,-
0.00000357,0.00318678,0.00046334,-0.00000189,-0.00004161,-
0.00005862,0.00000029,-0.00026341,-0.00066952,-0.00000029,-
0.00012547,0.00086261,0.00000129,0.02001871,0.36731553,-
0.00000004,0.00000010,-0.00030348,0.00000002,0.00000006,-0.00000272,0.,-
0.00000001,-0.00002962,0.00000002,0.,0.00002133,0.00000002,-
0.00000002,0.00006826,0.00000006,0.00000006,0.00028592,0.,0.,0.00000631,0.,0.
,-0.00000273,-0.00000002,-0.00000001,-
0.00000171,0.,0.,0.00000050,0.,0.00000002,-0.00000481,0.,0.,-
0.00000551,0.,0.,0.00000692,0.,0.,0.00000289,0.00000002,0.,0.00000118,0.,-
0.00000001,0.00000564,0.00000172,-
0.00000141,0.00771213,0.00000009,0.00000016,-
0.00028020,0.00000044,0.00000260,0.00332879,-0.00000013,-0.00007306,-
0.03787170,-0.00000130,-0.00000870,0.00273000,0.00000268,-
0.00000169,0.00583567,-0.00000022,0.00000029,-0.00118377,0.,-
0.00000059,0.00072793,-0.00000097,0.00000114,-0.00426114,-
0.00000109,0.00008036,0.02631717,-0.00769107,-0.04260865,-0.00000979,-
0.10087717,-0.00683452,0.00000522,0.00012182,0.03886120,0.00001195,-
0.00682336,0.00104154,0.00000365,0.00334977,-0.00050593,-0.00000194,-
0.00703160,0.00087664,0.00000369,0.00074313,0.00142315,0.00000040,-
0.00003371,-0.00007674,-0.00000003,0.00003371,-0.00002205,0.,-
0.00004898,0.00005654,0.00000003,0.00005738,0.00016149,0.00000012,-
0.00046880,0.00001646,0.00000006,0.00001407,0.00003332,0.,0.00001332,0.0000
3087,0.00000004,-0.00001288,0.00000121,0.,-
0.00004296,0.00002572,0.00000002,0.00052776,-0.00154729,-0.00000058,-
0.00002776,0.00007306,0.00000004,-
0.00046487,0.00006454,0.00000008,0.00002588,-0.00016317,-0.00000005,-
0.00008324,-0.00007834,-
0.00000002,0.00004278,0.00002053,0.00000002,0.00002309,-0.00002450,-

0.00000001,-0.00001279,-0.00000105,0.,0.00001908,-0.00002355,-0.00000002,-
0.00004777,-0.00002622,0.,0.70555399,-0.02408679,-0.02995788,-0.00000460,-
0.00677533,-0.20728151,-0.00003739,0.02035207,-0.03791886,-
0.00001643,0.00257579,0.00820018,-0.00000059,-0.00049704,-0.00478941,-
0.00000007,-0.00065453,0.00845330,0.00000045,0.00012301,-0.00043723,-
0.00000024,0.00005796,-0.00008497,0.00000007,-0.00006703,0.00004296,-
0.00000002,0.00018994,-0.00019959,-0.00000015,0.00010567,-0.00001462,-
0.00000003,-0.00000384,0.00021217,0.00000014,-
0.00006080,0.00009708,0.00000010,-
0.00007330,0.00005275,0.,0.00002128,0.00000169,-0.00000001,0.00001282,-
0.00001451,-0.00000002,-0.00024622,-0.00022126,-0.00000003,-0.00005978,-
0.00009149,0.,0.00008572,0.00019905,0.00000010,-
0.00010862,0.00002520,0.00000003,-0.00019527,-0.00018316,-
0.00000005,0.00006053,0.00003489,0.00000002,0.00007774,0.00004702,-
0.00000001,-0.00001493,-0.00000023,-0.00000001,0.00007149,0.00009273,0.,-
0.00000835,-0.00001588,-0.00000002,-0.00460014,0.63904214,-0.00000407,-
0.00000066,0.00420373,0.00000481,-0.00003594,-0.07237217,0.00000712,-
0.00002034,0.00424164,0.00000395,-0.00000096,0.00907341,-
0.00000192,0.00000040,-0.00486985,0.00000316,-0.00000067,0.00906018,-
0.00000004,-0.00000042,0.00008836,0.,-
0.00000003,0.00001858,0.00000008,0.00000007,0.00005961,0.00000010,-
0.00000007,-0.00001314,-0.00000006,-0.00000008,-
0.00002409,0.00000008,0.00000013,-0.00015037,-0.00000005,0.00000003,-
0.00001389,-0.00000006,0.00000005,-0.00003269,-0.00000007,-
0.00000004,0.00000633,0.00000001,0.00000003,0.00003584,-
0.00000016,0.00000033,0.00008835,0.,-
0.00000006,0.00001868,0.00000006,0.00000004,-0.00014459,-
0.00000002,0.00000009,-0.00002941,-0.00000002,-0.00000002,-0.00000685,-
0.00000006,0.00000004,0.00005677,-0.00000001,0.,-0.00003108,0.00000005,-
0.00000003,0.00000554,0.00000003,0.00000002,-0.00001452,0.00000002,-
0.00000006,0.00003558,-
0.00014570,0.00013429,0.15621698,0.00379597,0.00697807,0.00000188,0.005463
12,-0.01881983,-0.00000603,-0.00476988,0.00202702,0.00000209,-0.00001145,-
0.00068153,-0.00000020,-0.00108999,0.00114828,0.00000057,0.00075978,-
0.00289011,-0.00000102,-0.00008172,-0.00004196,-0.00000002,-
0.00001089,0.00056843,0.00000046,-0.00002302,-0.00015542,-0.00000007,-
0.00003742,0.00009686,0.00000004,-0.00008899,-0.00005900,0.,-0.00003578,-
0.00011980,-0.00000006,0.00002280,-0.00008793,-0.00000007,-
0.00017330,0.00020396,-0.00000018,-0.00003601,0.00002446,0.,-
0.00000461,0.00000052,0.,0.00004854,0.00028205,0.00000006,0.00005351,0.0001
7137,0.00000004,0.00006809,-0.00012548,-
0.00000005,0.00006777,0.00000388,0.00000002,0.00009174,0.00011286,-
0.00000001,-0.00004881,-0.00004673,0.00000001,-
0.00005869,0.00004471,0.00000002,0.00002061,0.00001082,-0.00000001,-
0.00002551,-0.00004042,-0.00000001,0.00001339,0.00001055,-0.00000001,-
0.28347229,-0.09675731,0.00003253,0.70891003,0.00236167,-0.00262857,-
0.00000173,-0.03120939,-0.02199475,0.00000176,-0.00311731,-0.00460683,-
0.00000060,-0.00125150,0.00193253,0.00000081,-
0.00129983,0.00007715,0.00000043,-0.00020629,-0.00326752,-
0.00000141,0.00009060,-0.00003889,0.00000007,0.00000680,-0.00045026,-
0.00000036,-0.00001908,0.00012780,0.00000005,0.00005530,-0.00012756,-

0.00000008,0.00010925,0.00004685,0.,0.00006156,0.00011693,0.00000005,-
0.00001600,0.00006352,0.00000005,0.00000712,-
0.00010345,0.00000017,0.00004383,-0.00003430,-0.00000002,0.00000649,-
0.00000199,0.,0.00030133,-0.00030439,-0.00000005,-0.00001007,0.00002733,0.,-
0.00027061,-0.00010327,0.00000003,0.00011325,-0.00003648,-0.00000004,-
0.00010609,0.00000744,0.00000002,-0.00002073,-0.00002913,-
0.00000002,0.00003556,-0.00002406,0.,-
0.00000539,0.00000046,0.,0.00001234,0.00002197,0.00000001,-0.00001520,-
0.00001203,0.,-0.04176006,-0.15633141,-0.00001271,-
0.04477081,0.72086276,0.00000010,-0.00000331,0.00220914,-0.00000946,-
0.00000206,0.00229766,0.00000071,-0.00000176,0.00094544,-
0.00000041,0.00000064,-0.00025688,-0.00000002,-0.00000021,-0.00005336,-
0.00000019,-0.00000031,0.00037681,0.00000005,0.00000003,0.00001912,0.,-
0.00000043,0.00006282,0.,0.00000009,-0.00000036,0.00000001,-0.00000005,-
0.00000588,0.00000005,0.00000003,0.00001304,0.00000004,0.00000008,-
0.00002040,0.,0.00000004,0.00000034,0.00000008,-0.00000008,-
0.00010677,0.00000003,-0.00000002,-0.00000550,0.,0.,-0.00000268,0.00000008,-
0.00000008,-0.00015035,-0.00000002,-0.00000002,-0.00002045,-0.00000009,0.,-
0.00004851,0.00000005,-0.00000004,0.00004010,-0.00000008,-0.00000002,-
0.00001695,0.,0.00000002,0.00003442,0.00000002,-0.00000002,-0.00000890,0.,0.,-
0.00000253,0.,0.00000002,-0.00000592,0.,-0.00000002,-
0.00000130,0.00004777,0.00000268,-0.06659301,-
0.00016824,0.00016702,0.13678402,0.00072153,-0.00096465,-0.00000044,-
0.00603524,-
0.00191767,0.00000315,0.00017200,0.00148438,0.00000011,0.00055379,-
0.00040330,-0.00000010,0.00001109,0.00053702,0.00000010,-0.00049241,-
0.00104531,-0.00000044,0.00000163,0.00000357,-0.00000002,-0.00001335,-
0.00016738,-0.00000015,0.00003601,0.00005485,0.00000003,-
0.00006180,0.00001666,0.00000003,0.00002355,0.00001338,0.,-
0.00000430,0.00002150,0.00000003,0.00000517,-0.00000032,0.,0.00010776,-
0.00009138,0.00000003,0.00000203,-0.00000358,0.,-
0.00000046,0.00000308,0.,0.00011891,-0.00012587,-
0.00000011,0.00003472,0.00013053,0.,-0.00006501,-0.00004963,0.,0.00005691,-
0.00004063,-0.00000002,0.00002237,0.00005340,0.00000005,-0.00002069,-
0.00003915,-
0.00000004,0.00001215,0.00001918,0.00000003,0.00001169,0.00000788,0.000000
01,-0.00000852,-0.00002802,0.,-0.00000276,-0.00000124,0.,0.02576756,-
0.03058836,-0.00001334,-0.13279240,-0.04680709,0.00000736,0.72320425,-
0.00183580,-0.00344961,-0.00000078,-
0.00211065,0.00512841,0.00000032,0.00044292,0.00153386,0.00000042,-
0.00048177,-0.00145419,-0.00000028,0.00165152,-0.00034394,-0.00000059,-
0.00091676,0.00245822,0.00000140,-0.00003685,0.00007573,-0.00000007,-
0.00002705,0.00012073,0.00000010,0.00004375,-0.00004093,-0.00000002,-
0.00002081,0.00006857,0.00000005,-0.00006599,-0.00000516,0.00000002,-
0.00000477,-0.00007317,-0.00000003,0.00000255,-0.00000886,-
0.00000002,0.00001566,0.00003836,-0.00000004,-0.00001410,0.00001157,0.,-
0.00000319,0.00000094,0.,-0.00007024,-0.00007646,-0.00000008,-0.00003782,-
0.00007681,0.,0.00005576,0.00009480,0.00000002,-
0.00008493,0.00000411,0.,0.00001314,-
0.00005952,0.,0.00004036,0.00003917,0.,0.00000368,-0.00001420,-0.00000001,-
0.00000788,-0.00000542,0.,-0.00000073,-0.00000346,0.,-

0.00000248,0.00000012,0.00000001,-0.07553802,-
0.02342522,0.00001226,0.01926259,-0.35493953,-
0.00008200,0.04094786,0.71165472,-0.00000059,-
0.00000053,0.00029959,0.00000292,-0.00000014,0.00663746,-0.00000014,0.,-
0.00059166,-0.00000014,-0.00000024,0.00022775,0.00000035,-0.00000023,-
0.00004122,-0.00000015,0.00000112,-0.00077426,-
0.00000003,0.00000002,0.00005683,0.00000001,0.00000007,-
0.00000016,0.00000001,0.00000002,0.00000790,0.00000002,0.,0.00000033,-
0.00000003,-0.00000003,-0.00001155,0.,-0.00000003,0.00003443,0.,0.,-
0.00000342,-0.00000003,0.00000002,-0.00001564,-0.00000001,0.,-
0.00000479,0.,0.,-0.00000350,-0.00000010,0.00000003,-0.00002428,0.,-
0.00000005,0.00001294,0.00000002,0.00000002,0.00003838,-
0.00000005,0.00000004,-0.00000738,0.,-0.00000003,0.00000537,0.,0.,-
0.00001158,0.,0.,-0.00000281,0.00000001,0.,-0.00000162,0.00000002,0.,-
0.00000278,0.,0.,-0.00000371,-0.00002578,0.00000052,0.00672919,0.00002539,-
0.00006375,-0.06078570,-0.00015238,0.00013842,0.13717158,-0.00060809,-
0.00224444,-0.00000059,-0.00203387,0.00005272,0.00000035,-
0.00039060,0.00221406,0.00000069,-0.00090647,-0.00166798,-
0.00000033,0.00158363,-0.00012522,-0.00000039,-
0.00057853,0.00180877,0.00000076,-0.00003959,0.00005331,0.00000003,-
0.00002317,0.00009934,0.00000006,0.00002327,-0.00004522,-
0.00000001,0.00001543,0.00004919,0.00000001,-0.00006784,-
0.00000131,0.00000002,0.00002567,-0.00007473,-0.00000004,-0.00000041,-
0.00000033,-0.00000001,-0.00001234,0.00002924,-0.00000002,-
0.00000695,0.00000515,0.,-0.00000205,0.00000031,0.,-0.00005524,-
0.00006005,0.,-0.00003909,-0.00009789,-
0.00000001,0.00002517,0.00007403,0.00000002,-
0.00006713,0.00001151,0.00000003,-0.00000959,-0.00006355,-
0.00000004,0.00003416,0.00003878,0.00000002,-0.00000299,-0.00002503,-
0.00000002,-0.00000942,-0.00000517,0.,0.00000225,0.00000463,0.,-0.00000257,-
0.00000135,0.,-
0.05991004,0.00409027,0.00001507,0.03754646,0.06796736,0.00001015,-
0.26904717,0.05798680,0.00007199,0.71311514,-0.00072013,-0.00240476,-
0.00000052,0.00033685,0.00336268,0.00000095,0.00045378,-0.00277838,-
0.00000089,-0.00119636,0.00188987,0.00000091,-0.00019080,-0.00123925,-
0.00000038,0.00152236,0.00148086,0.00000015,0.00017016,-0.00022680,-
0.00000012,0.00004961,-0.00014279,-0.00000004,-0.00009040,-0.00001163,-
0.00000001,0.00030062,-0.00021979,-
0.00000016,0.00003877,0.00003068,0.00000001,0.00014424,0.00007353,0.,-
0.00004145,0.00009348,0.00000007,-0.00015381,0.00001735,0.,0.00003186,-
0.00000226,0.,0.00000889,-0.00001637,0.,-0.00019313,-0.00019355,-0.00000002,-
0.00005594,-0.00012536,-0.00000003,-0.00014197,0.00007497,0.00000006,-
0.00002193,0.00004130,0.00000003,-0.00031499,-0.00019475,0.,0.00007615,-
0.00002732,-0.00000002,0.00015798,0.00000097,-0.00000002,-0.00002505,-
0.00000485,0.,0.00005307,0.00008796,0.,-0.00001013,-0.00002279,-
0.00000001,0.00370991,-0.00075980,0.00000094,0.02253673,-0.03579202,-
0.00001787,0.11593561,-0.17716732,-0.00006129,-0.00228012,0.63925472,-
0.00000003,0.00000007,-0.00013079,0.00000048,0.00000104,-
0.00041664,0.00000022,-0.00000133,-0.00013339,-
0.00000006,0.00000095,0.00002137,-0.00000049,-
0.00000031,0.00004639,0.00000051,-0.00000013,0.00001954,0.00000008,-

0.00000010,-0.00000682,0.00000002,-0.00000005,-0.00000705,-0.00000007,-
0.00000004,0.00000032,0.00000005,-0.00000007,-
0.00000054,0.00000006,0.00000006,0.00000533,0.00000002,0.00000004,-
0.00001688,0.,0.00000002,0.00000315,-
0.00000004,0.00000001,0.00001257,0.00000004,0.00000001,0.00000053,0.,-
0.00000003,0.00000040,0.,-0.00000005,-0.00001315,-0.00000003,-0.00000002,-
0.00000586,-0.00000002,0.00000003,-0.00001527,0.,-0.00000002,0.00000513,-
0.00000007,-0.00000004,-0.00000051,0.00000004,-
0.00000001,0.00000031,0.00000005,0.,0.00001206,-
0.00000004,0.00000001,0.00000036,0.,0.00000004,0.00000288,0.,0.00000001,0.00
000032,0.00001551,0.00000066,-0.00755152,-0.00000253,-
0.00002999,0.00727553,0.00008701,-0.00004534,-0.06483992,-
0.00015933,0.00012994,0.14363227,-0.00007573,-0.00133653,-0.00000059,-
0.00533760,0.00328723,0.00000405,0.00102175,0.00035834,-0.00000019,-
0.00021791,0.00139272,0.00000030,-0.00025513,-0.00056499,-
0.00000012,0.00064358,0.00017925,0.00000002,0.00014311,0.00009521,-
0.00000006,0.00002079,-0.00014424,-0.00000005,-0.00002067,0.00004527,-
0.00000001,0.00001895,-0.00006126,0.,0.00007171,0.00003214,-0.00000002,-
0.00006184,0.00007043,0.00000005,-
0.00000536,0.00002960,0.00000003,0.00000802,-
0.00002261,0.00000002,0.00001197,-0.00000884,0.,-
0.00000202,0.00000154,0.00000001,0.00000179,0.00000367,-
0.00000005,0.00001437,0.00017691,0.00000005,-0.00000989,-
0.00002933,0.,0.00002964,-0.00001592,-0.00000004,-0.00006156,-
0.00000396,0.00000004,0.00002099,-0.00006350,-
0.00000002,0.00011719,0.00007971,0.00000002,0.00000570,0.00000377,0.,0.0000
0549,-0.00000015,0.,-0.00000046,-
0.00000471,0.00000001,0.03774334,0.02243157,-0.00000207,-0.01951792,-
0.02995369,-0.00000442,-0.05585210,0.02930284,0.00002533,-0.28982517,-
0.10121913,0.00003535,0.71693036,-
0.00023206,0.00178669,0.00000082,0.00337714,0.00440725,-
0.00000140,0.00126379,-0.00368404,-
0.00000143,0.00125996,0.00215281,0.00000057,-0.00166664,-
0.00004813,0.00000040,0.00020199,-0.00156005,-0.00000071,0.00004092,-
0.00009204,-0.00000002,0.00002457,-0.00006338,-0.00000002,-
0.00003136,0.00003854,0.,-0.00002745,-0.00004470,-0.00000002,0.00007637,-
0.00001053,-0.00000003,-0.00003902,0.00009242,0.00000004,0.00000159,-
0.00000817,0.,-0.00000458,-0.00001137,0.,0.00000548,-
0.00000508,0.,0.00000272,-
0.00000032,0.,0.00004897,0.00007455,0.,0.00004055,0.00008999,0.00000002,-
0.00000075,-0.00006982,-0.00000003,0.00006035,-0.00001181,-
0.00000002,0.00004148,0.00007319,0.00000002,-0.00005243,-0.00002584,0.,-
0.00003035,0.00002755,0.00000003,0.00001371,0.00001058,0.,-0.00000483,-
0.00000898,0.,0.00000344,0.00000226,0.,0.06815460,-0.03584275,-0.00002979,-
0.02937482,-0.04715438,-0.00000281,-0.01437665,0.06346432,0.00001840,-
0.04338689,-0.15833942,-0.00001374,-0.04348678,0.70974212,-
0.00000020,0.00000106,-0.00058325,0.00000414,-
0.00000153,0.00660592,0.00000009,-
0.00000131,0.00031154,0.00000029,0.00000046,-0.00077337,-
0.00000039,0.00000017,-0.00004334,-0.00000002,-0.00000056,0.00023090,-
0.00000007,-0.00000002,-

0.00002942,0.,0.00000002,0.00001310,0.00000002,0.00000003,-
0.00001140,0.,0.,0.00000511,-0.00000002,-0.00000004,-0.00000734,0.00000002,-
0.00000002,0.00004008,0.,0.,-0.00000325,0.,-0.00000002,-0.00000311,-
0.00000001,0.,-0.00000173,0.,0.,-
0.00000384,0.,0.00000001,0.00005958,0.00000003,0.,-0.00000033,0.,-
0.00000004,0.00003513,0.00000002,0.00000002,-
0.00001136,0.00000002,0.00000003,0.00000026,-0.00000004,0.,0.00000793,-
0.00000005,-0.00000001,-0.00001594,0.00000002,0.,-0.00000487,0.,-0.00000001,-
0.00000368,0.,0.,-0.00000370,0.00000963,-0.00001731,0.00678914,-0.00000435,-
0.00000262,-
0.00654617,0.00001368,0.00000654,0.00844383,0.00005007,0.00000194,-
0.06257871,-0.00016888,0.00016533,0.13451516,-0.00461823,-
0.00209529,0.00000079,0.01118660,0.01462093,0.00000201,0.00253161,-
0.00759205,-0.00000214,0.00108725,0.00232747,0.00000065,-0.00104415,-
0.00099921,-0.00000017,0.00023544,0.00088754,0.00000026,-0.00003247,-
0.00031406,-0.00000019,0.00003527,-0.00017112,-0.00000009,-
0.00004168,0.00004787,0.,0.00009191,-0.00011252,-0.00000004,0.00005205,-
0.00001801,-0.00000004,0.00011088,0.00010058,0.00000001,-
0.00002074,0.00004500,0.00000005,-0.00006655,-
0.00003924,0.00000002,0.00002216,-0.00001084,0.00000001,0.00001397,-
0.00001037,0.00000001,-0.00008061,0.00004873,-0.00000004,-0.00009173,-
0.00061603,-0.00000009,-0.00002542,0.00013594,0.00000003,-
0.00009427,0.00007790,0.00000002,-0.00004848,-
0.00011379,0.00000001,0.00000290,0.00017651,0.00000003,-0.00020332,-
0.00018986,-0.00000012,-0.00004478,-
0.00002655,0.00000002,0.00003621,0.00009062,0.00000002,-0.00000562,-
0.00000045,0.00000002,-0.26520568,0.11095214,0.00008235,-0.05446240,-
0.01322720,0.00001369,-0.01282816,0.02489636,0.00000829,0.02493989,-
0.03053466,-0.00001298,-0.13219754,-
0.04681797,0.00000670,0.72179227,0.00311645,-0.00479968,-
0.00000257,0.02704938,-0.02778668,-0.00001553,-0.00300517,-0.00141847,-
0.00000031,-0.00036018,-0.00357786,-0.00000101,0.00145688,0.00002177,-
0.00000038,0.00148016,0.00168350,0.00000046,-0.00033555,-0.00023357,-
0.00000001,0.00001263,0.00004583,0.00000002,0.00001930,-0.00003776,-
0.00000002,0.00010360,0.00000811,-0.00000002,-0.00012360,-
0.00002236,0.,0.00024563,-0.00014630,-0.00000009,-0.00000743,0.00001910,0.,-
0.00003314,-0.00001439,-0.00000001,0.00000492,0.00000207,0.,0.00001231,-
0.00001034,-0.00000001,-0.00008467,-0.00003890,-0.00000002,-0.00005280,-
0.00036942,-0.00000007,-0.00004786,0.00010618,0.00000005,-
0.00009094,0.00005139,0.00000002,-0.00006435,-0.00011748,-
0.00000002,0.00003646,0.00010403,0.00000003,0.00000921,-0.00007337,-
0.00000004,-0.00004160,-0.00002840,0.,0.00002029,0.00005047,0.,-0.00000631,-
0.00000224,0.,0.05593212,-0.17617095,-
0.00004489,0.02769773,0.06227679,0.00000687,0.02605046,-0.05386723,-
0.00001973,-0.07679322,-0.02324878,0.00001274,0.02030618,-0.35040813,-
0.00008363,0.04281069,0.70645841,0.00000230,-
0.00000153,0.00097706,0.00000522,-0.00001187,0.00229189,-
0.00000094,0.00000094,0.00218522,-0.00000032,-
0.00000156,0.00037646,0.00000067,0.00000018,-
0.00005567,0.00000037,0.00000048,-0.00025599,-0.00000010,0.00000002,-
0.00014444,-0.00000002,0.00000006,-0.00002216,0.00000001,-

0.00000006,0.00003513,0.,0.00000004,-0.00001528,-0.00000003,-
0.00000002,0.00003839,0.00000005,-0.00000001,-0.00004854,0.,0.,-
0.00000552,0.,0.00000003,-0.00000821,-0.00000001,0.,-0.00000272,0.,0.,-
0.00000132,-0.00000001,-
0.00000005,0.00001874,0.00000002,0.00000010,0.00006262,0.,0.,-
0.00002206,0.,0.,0.00001312,-0.00000003,0.,-0.00000702,0.00000001,-
0.00000003,-0.00000022,0.00000006,0.00000007,-0.00010658,0.,0.,-
0.00000524,0.,0.,0.00000059,0.,0.,-0.00000240,0.00006758,-0.00005979,-
0.06669978,0.00002441,0.00001811,0.00872673,0.00000872,-0.00001934,-
0.00653273,-0.00002479,-0.00000012,0.00752804,0.00002413,-0.00006581,-
0.06038495,-0.00014263,0.00014374,0.13634814,0.00065427,-0.00191573,-
0.00000076,-0.00140594,-0.00220305,-
0.00000125,0.00014240,0.00037320,0.00000004,-0.00034251,-
0.00020578,0.00000004,0.00048622,-0.00040519,-
0.00000013,0.00088237,0.00071980,-
0.00000015,0.00005340,0.00000372,0.00000004,-0.00000111,-0.00006904,-
0.00000006,-0.00000327,0.00000514,0.00000001,0.00004140,-0.00002721,-
0.00000003,0.00000769,0.00001854,0.00000001,0.00002238,0.00001288,-
0.00000001,-0.00000198,0.00001791,0.00000001,0.00000279,-
0.00001312,0.,0.00000686,-0.00000325,0.,0.00000060,-0.00000113,0.,0.00002448,-
0.00002329,0.00000003,-0.00002868,-0.00011920,-0.00000007,-
0.00005849,0.00004374,0.,-0.00001814,0.00000737,0.00000002,-0.00004417,-
0.00004729,-0.00000002,0.00002326,0.00004237,0.00000001,0.00006973,-
0.00006907,0.,-0.00001786,-0.00001394,0.,0.00000644,0.00001527,0.,-
0.00000588,-0.00000311,0.,-0.02030939,-0.01264636,0.00000287,-
0.00235586,0.00424683,0.00000321,0.00028281,0.00058641,0.,0.00138633,0.0005
2647,0.00000172,0.00407279,0.00151088,0.00000023,-0.29046389,-
0.11437454,0.00003436,0.30694732,0.00168991,-0.00255153,-0.00000108,-
0.00022203,0.00054720,0.00000116,-
0.00042578,0.00017568,0.00000020,0.00026357,-0.00002653,-0.00000017,-
0.00046951,0.00035353,0.00000014,-0.00054494,-0.00068416,0.00000003,-
0.00009156,0.00005802,0.00000007,0.00000132,0.00008789,0.00000003,0.000009
83,-0.00000035,0.00000001,-0.00007579,0.00004870,0.00000003,-0.00000865,-
0.00001979,-0.00000001,-0.00003972,-0.00002588,0.,0.00000972,-0.00002299,-
0.00000002,0.00000935,0.00000991,0.,-0.00000970,0.00000277,0.,-
0.00000164,0.00000338,0.,0.00008244,0.00004096,0.,0.00007617,-0.00024874,-
0.00000001,0.00003789,-0.00002230,-0.00000002,-
0.00001077,0.00001465,0.00000001,0.00013554,0.00004935,-0.00000003,-
0.00006464,0.00012303,0.00000005,-0.00042378,-0.00012758,-
0.00000011,0.00000319,0.00000098,0.,-
0.00000724,0.00000628,0.,0.00000348,0.00000876,0.,0.01534100,0.01342576,-
0.00000136,0.00318622,-0.00234645,-0.00000310,0.00076590,-0.00069718,-
0.00000029,0.00119247,-0.00518603,-0.00000387,-0.02861365,-
0.00957082,0.00000398,-0.11645268,-
0.11654675,0.00000934,0.12490020,0.12164721,0.00000038,-0.00000032,-
0.00006058,-0.00000075,0.00000159,-0.00353481,-0.00000024,0.00000011,-
0.00016152,0.00000020,-0.00000008,0.00024715,-
0.00000017,0.00000011,0.00031356,-0.00000052,-0.00000020,-0.00045155,-
0.00000005,0.00000003,-0.00001457,0.,0.00000006,0.00000048,0.,0.,-0.00000367,-
0.00000004,0.00000002,0.00000287,-0.00000001,0.,-0.00000277,-0.00000002,-
0.00000002,-0.00000598,0.,-

0.00000002,0.00000515,0.,0.,0.00000147,0.,0.,0.00000290,0.,0.,0.00000178,0.0000
0003,0.00000004,-0.00003286,0.,-0.00000013,-0.00010610,0.00000002,0.,-
0.00000819,0.,0.,-0.00000291,0.00000007,0.00000002,0.00001245,-
0.00000002,0.00000007,-0.00001565,-0.00000015,-
0.00000014,0.00026301,0.,0.,0.00000691,0.,0.,0.00000150,0.,0.,0.00000472,0.0000
1032,0.00000630,0.00347901,0.00000311,-0.00000331,0.00572961,0.00000008,-
0.00000026,-0.00037367,0.00000198,-0.00000357,0.00767757,-0.00000777,-
0.00000374,0.00342158,0.00003360,0.00000832,-0.03874238,-0.00003878,-
0.00000674,0.02690598,0.00006011,0.00014765,0.00000008,0.00021462,-
0.00033032,-0.00000015,-0.00001449,-0.00000292,-0.00000004,0.00005562,-
0.00007199,-0.00000012,-0.00001977,0.00005530,0.00000007,-0.00002850,-
0.00008213,-0.00000007,-0.00000878,0.00000367,0.00000005,-0.00000029,-
0.00000260,0.,0.00000212,0.00000318,0.,-0.00001269,0.00000711,-
0.00000002,0.00000102,-0.00000078,0.,-0.00000383,-0.00000070,0.,0.00000174,-
0.00000280,0.,0.00000490,-0.00000321,0.,0.00000002,-
0.00000153,0.,0.00000007,0.00000057,0.,0.00000800,0.00001072,0.00000004,-
0.00000519,-0.00000665,0.,0.00000447,0.00000064,0.,-
0.00000224,0.00000045,0.,0.00001260,0.00000609,-
0.00000002,0.00000144,0.00000345,0.,-0.00000095,-0.00000530,0.,-0.00000157,-
0.00000294,0.,-0.00000184,-0.00000172,0.,-
0.00000004,0.00000149,0.,0.00106922,-0.00130128,0.00000147,0.00016682,-
0.00064831,-0.00000029,-0.00323315,-0.00418535,0.00000135,-
0.01936143,0.01695121,0.00001047,-
0.25988826,0.13131491,0.00009458,0.00703907,-0.00337501,-
0.00000190,0.00077416,0.00028143,-0.00000127,0.27331525,-0.00020358,-
0.00012504,-0.00000002,0.00152745,0.00042088,-0.00000027,0.00006822,-
0.00078355,-0.00000019,0.00013057,0.00012866,0.00000009,-0.00010879,-
0.00007851,-0.00000004,0.00012531,0.00009410,0.00000004,-0.00001412,-
0.00005478,-0.00000005,0.00000323,-0.00000777,0.,-0.00000495,-
0.00000034,0.,0.00001601,-0.00001256,0.,0.00000031,-
0.00000373,0.,0.00002517,0.00000505,0.,-0.00000229,0.00000351,0.,-0.00000973,-
0.00000202,0.,0.00000234,-0.00000053,0.,0.00000248,-0.00000187,0.,-
0.00000939,-0.00001900,-0.00000003,-0.00002593,-0.00006507,-0.00000001,-
0.00001504,0.00001969,0.,-0.00001368,0.00000920,0.,-0.00002062,-
0.00002175,0.00000001,0.00001385,0.00001197,0.,0.00001385,-0.00001769,-
0.00000001,-0.00001201,-0.00000867,0.,0.00000468,0.00001145,0.,-0.00000238,-
0.00000047,0.,-0.00201673,-0.00502980,-0.00000296,-0.00082614,-
0.00054514,0.00000014,-0.00322907,-0.00126573,-0.00000097,-
0.01224758,0.01237553,0.00000589,0.13377425,-0.14741723,-
0.00006499,0.02706979,-0.01279609,-0.00001158,-
0.00027241,0.00114764,0.00000153,-0.14240959,0.15504638,-0.00000006,-
0.00000011,0.00006816,0.00000040,0.00000015,0.00028655,-0.00000004,-
0.00000012,-0.00030367,0.00000002,0.00000003,-0.00000269,-0.00000003,-
0.00000003,-0.00002961,0.00000003,0.00000004,0.00002140,0.00000002,-
0.00000003,0.00003557,0.,0.,-0.00000242,-0.00000002,-0.00000001,-
0.00000368,0.,0.,0.00000032,0.00000001,0.00000002,-0.00000374,0.,0.,-
0.00000127,0.,0.,0.00000179,0.,0.,0.00000474,0.00000001,0.,0.00000118,0.,-
0.00000001,0.00000114,0.,-0.00000002,0.00000633,-0.00000002,-0.00000003,-
0.00000546,0.,0.00000002,-0.00000271,-0.00000001,0.,-0.00000170,0.,-
0.00000001,0.00000053,0.00000002,0.,-0.00000479,0.00000001,0.,0.00000719,-
0.00000001,0.,0.00000566,0.,0.,0.00000290,0.,0.,0.00000118,0.00000132,-

0.00000314,0.00771664,-0.00000041,0.00000007,-0.00028285,0.00000161,-
0.00000073,0.00583460,0.00000281,-0.00000177,0.00272832,0.00009500,-
0.00006449,-0.03791202,0.00000621,-0.00000329,0.00334026,-
0.00000136,0.00000142,-0.00424996,-
0.00010427,0.00007248,0.02634267,0.00014864,-0.00037430,-0.00000020,-
0.00108106,0.00241754,-
0.00000004,0.00050717,0.00152270,0.00000023,0.00083560,-0.00091159,-
0.00000073,0.00059784,0.00037462,0.00000015,-
0.00034416,0.00025031,0.00000020,0.00001835,0.00002872,0.00000002,-
0.00002852,0.00009017,0.00000002,0.00002844,-0.00002861,-0.00000001,-
0.00005736,0.00005636,0.00000003,-0.00001748,-0.00000200,0.,-0.00006769,-
0.00003945,-0.00000002,0.00000558,-0.00001494,-
0.00000001,0.00013272,0.00003594,0.00000001,-0.00001727,0.00001467,0.,-
0.00000596,0.00000346,0.,0.00006403,-0.00000436,-
0.00000001,0.00000832,0.00007941,0.00000002,0.00002517,-0.00001829,-
0.00000001,0.00000575,-0.00002283,-0.00000002,0.00005203,0.00003055,0.,-
0.00000433,-0.00000545,0.,0.00000347,0.00001385,0.,0.00000779,0.00000373,0.,-
0.00000557,-0.00002016,0.,0.00000086,0.00000241,0.,-
0.02011271,0.01689658,0.00001098,-
0.25871591,0.13244413,0.00009579,0.00727589,-0.00348770,-
0.00000198,0.00107308,-0.00131128,0.00000149,0.00010645,-0.00066729,-
0.00000029,-0.00324850,-0.00414106,0.00000132,-
0.00099865,0.00102857,0.00000074,0.00001957,0.00009346,-
0.00000031,0.27277586,0.00042654,0.00017092,0.00000004,0.00042613,0.000193
27,0.00000089,-0.00217033,-0.00250393,-0.00000023,0.00034968,-0.00063082,-
0.00000017,0.00043979,0.00024529,-0.00000010,-0.00022212,-0.00002448,0.,-
0.00007540,0.00005036,0.00000006,-0.00010950,-0.00024878,-
0.00000023,0.00008137,0.00011691,0.00000007,-
0.00012713,0.00005570,0.00000007,0.00001371,0.00001531,0.,-0.00003065,-
0.00001505,0.00000001,0.00000743,0.00000724,0.,0.00039073,-
0.00019577,0.00000004,-0.00000451,-0.00000147,-0.00000002,-
0.00000234,0.00000612,0.,0.00009034,0.00004721,-
0.00000002,0.00000766,0.00007796,0.00000002,0.00003415,-0.00002710,-
0.00000001,0.00000515,-0.00001965,0.,0.00007168,0.00004122,0.,-
0.00000811,0.00000082,0.,-0.00000862,0.00000924,0.,0.00000798,0.00000263,0.,-
0.00001190,-0.00001913,0.,0.00000178,0.00000432,0.,-
0.01108980,0.01325712,0.00000568,0.13471960,-0.14796935,-
0.00006625,0.02653530,-0.01271514,-0.00001160,-0.00192557,-0.00482488,-
0.00000273,-0.00086671,-0.00053883,0.00000017,-0.00307688,-0.00138998,-
0.00000108,-
0.00082350,0.00061369,0.00000022,0.00008166,0.00001096,0.00000029,-
0.14429961,0.15556730,-0.00000003,0.00000009,-0.00016480,-
0.00000052,0.00000062,-0.00348046,-0.00000070,-0.00000107,-0.00005395,-
0.00000030,0.00000012,-0.00045006,0.00000009,-
0.00000017,0.00031180,0.00000003,-0.00000007,0.00024151,-0.00000003,-
0.00000003,-0.00003161,-0.00000003,0.00000020,-0.00010717,0.00000001,-
0.00000003,-0.00001618,0.,0.,0.00001190,0.,0.,-0.00000272,-0.00000001,0.,-
0.00000904,0.,0.,0.00000163,-
0.00000002,0.,0.00025761,0.,0.,0.00000697,0.,0.,0.00000484,0.,0.,-0.00001415,0.,-
0.00000001,0.00000026,0.,0.,-0.00000555,-0.00000002,0.,-
0.00000326,0.00000001,0.,0.00000315,0.00000001,0.,-

0.00000345,0.,0.,0.00000167,0.,0.,0.00000281,0.,0.,0.00000514,0.,0.,0.00000179,0.
00000347,-0.00000231,0.00351535,0.00009667,-0.00006569,-
0.03887262,0.00000598,-0.00000330,0.00349638,0.00000133,-
0.00000289,0.00771691,-0.00000032,0.00000008,-0.00032193,0.00000159,-
0.00000075,0.00577757,0.00000024,-0.00000032,0.00068364,-
0.00000031,0.00000030,-0.00118334,-0.00010600,0.00007397,0.02684248,-
0.00000879,-0.00002032,-
0.00000005,0.00010771,0.00027842,0.00000006,0.00003836,-
0.00011706,0.00000003,-0.00001113,0.00005920,-0.00000003,-0.00001873,-
0.00003895,0.00000002,0.00003764,0.00004747,-0.00000006,0.00000359,-
0.00000841,0.00000004,-0.00000300,0.00000240,-0.00000001,0.00000182,-
0.00000156,0.,0.00000699,-0.00000431,-0.00000002,-0.00000111,-
0.00000050,0.,0.00000329,-0.00000068,0.,-
0.00000122,0.00000139,0.,0.00000097,0.00000322,0.,-
0.00000044,0.00000231,0.,0.,-0.00000074,0.,-0.00000481,-
0.00000373,0.00000005,0.00000046,0.00000249,0.,-
0.00000330,0.00000036,0.,0.00000119,0.00000021,0.,-0.00000889,-0.00000418,-
0.00000001,0.00000061,-
0.00000246,0.,0.00000355,0.00000196,0.,0.00000035,0.00000097,0.,0.00000144,0.
00000169,0.,-0.00000006,-
0.00000069,0.,0.00136839,0.00067614,0.00000205,0.00437388,0.00141004,0.0000
0009,-0.28717990,-0.11493747,0.00003972,-0.02030926,-0.01273987,0.00000342,-
0.00232553,0.00420325,0.00000335,0.00031444,0.00056432,-0.00000005,-
0.00000032,-0.00007852,-0.00000033,-0.00095941,-
0.00076724,0.00000025,0.00073415,-0.00024383,-0.00000150,0.30336354,-
0.00014956,-0.00073139,-0.00000009,-
0.00143734,0.00055300,0.00000047,0.00017978,-0.00011853,-0.00000008,-
0.00010722,0.00009002,0.00000003,0.00010445,-0.00008018,-0.00000004,-
0.00012273,0.00013250,0.00000004,0.00000549,-0.00001661,0.,0.00001752,-
0.00007011,-0.00000006,-0.00001146,0.00001449,0.,0.00001546,-0.00002131,-
0.00000002,0.00001527,0.00000738,0.,0.00001552,0.00001796,0.,-
0.00000285,0.00001154,0.,-0.00001429,-0.00001766,0.00000002,0.00001135,-
0.00000980,0.,0.00000216,-0.00000033,0.,0.00000918,-0.00005254,-0.00000001,-
0.00000358,-0.00000692,0.,-0.00002229,0.00000706,0.,-0.00000090,-
0.00000345,0.,-0.00001280,-0.00000959,0.,0.00000380,-
0.00000006,0.,0.00000780,-0.00000347,0.,-0.00000195,-
0.00000069,0.,0.00000214,0.00000251,0.,-0.00000256,-0.00000174,0.,0.00119091,-
0.00524096,-0.00000371,-0.02839937,-0.00931107,0.00000463,-0.11715183,-
0.11900957,0.00001195,0.01542039,0.01305207,-0.00000188,0.00318254,-
0.00240057,-0.00000295,0.00074691,-0.00071793,-0.00000030,-
0.00009099,0.00001732,0.00000031,0.00103382,0.00066496,-
0.00000027,0.00031651,0.00116268,0.00000133,0.12497905,0.12247027,-
0.00000009,-0.00000009,-0.00030696,-
0.00000035,0.00000002,0.00024024,0.00000006,0.,0.00007550,-
0.00000002,0.,0.00002582,0.00000003,0.,-0.00003264,-
0.00000006,0.00000003,0.00000287,0.00000001,-0.00000002,0.00000565,0.,-
0.00000001,-0.00000521,-0.00000002,-0.00000001,-
0.00000484,0.,0.,0.00000032,0.00000001,0.00000002,-0.00000162,0.,0.,-
0.00000250,0.,0.,0.00000281,0.,0.,0.00000690,0.00000002,0.,0.00000565,0.,0.,0.00
000117,0.00000002,-0.00000004,0.00003589,-0.00000001,-0.00000001,-
0.00000268,0.,0.00000001,-0.00000132,0.,0.,-

0.00000380,0.,0.,0.00000037,0.00000002,0.,-0.00000350,0.,0.,0.00000486,-
0.00000001,0.,0.00000116,0.,0.,0.00000177,0.,0.,0.00000112,0.00000204,-
0.00000365,0.00777483,-0.00000776,-
0.00000378,0.00336808,0.00003951,0.00001160,-
0.03916141,0.00001065,0.00000581,0.00379849,0.00000298,-
0.00000328,0.00560026,-0.00000003,-0.00000027,-0.00034738,-
0.00000031,0.00000033,-0.00109562,0.00000072,0.0000019,0.00072706,-
0.00000132,0.00000151,-0.00431312,-0.00004502,-0.00001023,0.02749337,-
0.00044091,-0.00066622,-0.00000002,-0.00026546,0.00073539,-
0.00000009,0.00000920,0.00016838,0.00000011,0.00018992,-0.00006800,-
0.00000012,-0.00012499,-0.00010565,0.00000006,0.00026906,0.00040536,-
0.00000004,-0.00000858,0.00001862,0.00000005,-
0.00003637,0.00007065,0.00000002,0.00004138,-0.00001987,0.,-
0.00001994,0.00003108,0.00000003,-0.00003446,-0.00000719,-
0.00000001,0.00000523,-0.00003323,-0.00000003,0.00000132,-
0.00001143,0.,0.00003854,-0.00000266,0.,-0.00001808,0.00001922,0.,0.00000055,-
0.00000228,0.,-0.00004156,-0.00004607,0.00000003,-0.00005201,-0.00007575,-
0.00000005,-0.00000721,0.00004526,0.,-0.00003566,0.00001559,0.00000002,-
0.00006978,-0.00005807,0.,0.00005578,0.00001132,-0.00000001,0.00005633,-
0.00000216,0.,-0.00002111,-0.00001750,0.,0.00000951,0.00002176,0.,-
0.00000107,-0.00000083,0.,-0.00407002,-0.00093591,0.00000064,-
0.00525606,0.00720311,0.00000536,0.01394755,0.02292255,0.00000263,-
0.09923572,0.00470466,0.00000695,0.00614753,-0.03004355,-0.00000950,-
0.00638883,-0.00361374,0.00000289,0.00010801,-0.00151591,-0.00000034,-
0.00233830,-0.00085391,-0.00000068,0.00024492,0.00161524,0.00000047,-
0.00320383,0.00257650,0.00000054,0.88843111,0.00241941,0.00524157,0.000001
02,-0.00062378,-0.00953667,-0.00000219,-
0.00171712,0.00547320,0.00000195,0.00159301,-0.00363613,-
0.00000152,0.00017171,0.00228513,0.00000069,-0.00210391,-0.00342024,-
0.00000072,-0.00035779,0.00049090,0.00000031,-0.00006575,0.00016475,-
0.00000003,0.00016820,0.00006084,0.00000005,-
0.00063203,0.00042229,0.00000031,-0.00003346,-0.00004789,-0.00000002,-
0.00029206,-0.00012995,0.00000001,0.00007787,-0.00016164,-
0.00000013,0.00027690,-0.00004351,0.00000002,-0.00003828,-0.00001915,-
0.00000001,-
0.00001275,0.00003415,0.00000002,0.00042659,0.00043918,0.00000011,0.000074
92,0.00016725,0.00000003,0.00028281,-0.00015569,-0.00000011,0.00002294,-
0.00006959,0.,0.00066584,0.00039348,-0.00000003,-
0.00014892,0.00007876,0.00000004,-0.00029065,-
0.00000831,0.00000004,0.00002644,-0.00000805,-0.00000002,-0.00009911,-
0.00015420,-
0.00000001,0.00001550,0.00004923,0.,0.00045151,0.00358006,0.00000095,0.0028
5321,0.00363330,-0.00000158,0.01431367,-0.02974656,-0.00001241,-0.00737226,-
0.19828495,-0.00002975,-0.01673373,-0.01520082,0.00000098,-
0.00103607,0.00497252,-0.00000025,0.00043200,0.00062098,-
0.00000004,0.00287634,0.00156769,0.00000054,-
0.00035233,0.00035821,0.00000011,-
0.00275215,0.00082176,0.00000198,0.17324129,0.71251044,0.00000078,0.000001
51,0.00008892,-0.00000038,-0.00000246,-0.00120939,-
0.00000043,0.00000141,0.00009213,0.00000034,-0.00000096,-
0.00007523,0.00000015,0.00000059,0.00015187,-0.00000064,-0.00000095,-

0.00009613,-0.00000014,0.00000018,-0.00001717,0.,0.00000004,-
0.00000488,0.00000007,0.00000007,-0.00000456,-
0.00000015,0.00000010,0.00000062,-0.00000004,-0.00000007,0.00000024,-
0.00000007,-0.00000003,-0.00000560,0.00000002,-
0.00000004,0.00000260,0.00000006,-0.00000003,0.00001135,-0.00000002,-
0.00000002,0.00000509,0.,0.00000002,0.00000240,0.00000009,0.00000015,-
0.00001693,0.00000005,0.00000007,-0.00000643,0.00000006,-0.00000007,-
0.00000663,0.00000003,0.,0.00000042,0.00000019,0.00000012,0.00000052,-
0.00000007,0.00000002,-0.00000413,-0.00000009,-
0.00000001,0.00001216,0.00000003,0.,0.00000515,-0.00000002,-
0.00000006,0.00000283,0.,0.,0.00000248,0.00000117,0.00000160,-
0.00077927,0.00000420,-0.00000320,0.00714716,-0.00000009,-
0.00001412,0.00202980,0.00000354,-0.00003214,-0.07777884,-
0.00000587,0.00000409,0.00005915,0.00000350,0.00000017,0.00787069,0.000000
18,0.00000048,0.00035390,0.00000037,0.00000173,-0.00398996,-0.00000006,-
0.00000049,0.00033607,-0.00000076,0.00000042,-0.00387460,-
0.00018183,0.00006899,0.20049405,-0.00012832,-0.00069444,-
0.00000023,0.00017576,0.00120334,0.00000038,0.00034800,-0.00094523,-
0.00000038,-0.00045738,0.00058892,0.00000034,0.00017620,-0.00037041,-
0.00000021,0.00022894,0.00050041,0.00000021,0.00006992,-0.00009565,-
0.00000010,0.00003548,-0.00009387,-0.00000003,-
0.00005430,0.00000662,0.,0.00012619,-0.00009624,-
0.00000006,0.00002896,0.00001617,0.,0.00005233,0.00004419,0.00000001,-
0.00001542,0.00003986,0.00000003,-0.00006871,0.00000193,0.,0.00002000,-
0.00001039,0.,0.00000218,-0.00000452,0.,-0.00005288,-0.00005723,-
0.00000005,0.00001724,0.00002893,0.00000002,-
0.00004633,0.00000205,0.00000002,0.00001643,0.00000126,-0.00000001,-
0.00008158,-0.00003864,0.00000002,-0.00000281,-
0.00002560,0.,0.00002902,0.00000925,0.,0.00000822,0.00001238,0.,0.00001207,0.
00001346,0.,-0.00000251,-
0.00000885,0.,0.00012346,0.00042640,0.00000009,0.00101863,-0.00201069,-
0.00000105,-0.00682692,0.00605960,0.00000343,-0.01138244,-
0.03095004,0.00000241,0.00327986,0.00041074,-0.00000010,0.00067081,-
0.00134304,-0.00000112,-0.00001162,-0.00018896,-
0.00000012,0.00105277,0.00080374,0.00000024,0.00001465,-0.00032645,-
0.00000008,0.00279381,-0.00086145,-0.00000114,-0.57292077,-
0.26482102,0.00010037,0.68497356,-0.00104920,-0.00245627,-
0.00000053,0.00012401,0.00408458,0.00000109,0.00080919,-0.00240839,-
0.00000086,-0.00075567,0.00155985,0.00000069,0.00004231,-0.00102330,-
0.00000037,0.00085099,0.00156742,0.00000036,0.00016980,-0.00022878,-
0.00000014,0.00003135,-0.00006858,0.00000001,-0.00008106,-0.00003171,-
0.00000002,0.00029774,-0.00019478,-
0.00000015,0.00001334,0.00002216,0.00000001,0.00013369,0.00005838,0.,-
0.00003554,0.00007312,0.00000006,-
0.00013005,0.00002407,0.,0.00001782,0.00000864,0.,0.00000482,-0.00001533,-
0.00000001,-0.00019244,-0.00020225,-0.00000004,-0.00001843,-0.00003557,-
0.00000001,-0.00012841,0.00006046,0.00000004,0.00000059,0.00002547,0.,-
0.00029807,-0.00016925,0.00000002,0.00005794,-0.00004722,-
0.00000002,0.00012918,0.00001294,-0.00000001,-
0.00000504,0.00000986,0.,0.00004263,0.00006201,0.,-0.00000646,-0.00002316,0.,-
0.00148690,-0.00160186,-0.00000004,-0.00074581,0.00077189,0.00000050,-

0.00330652,-0.00058893,0.00000095,-0.04624044,-
0.02985116,0.00000291,0.00681414,-0.00561183,-0.00000395,-0.00194992,-
0.00253885,0.00000005,-0.00005193,-0.00075424,-0.00000013,-
0.00040620,0.00008433,0.00000006,0.00012867,-0.00012945,-0.00000006,-
0.00071152,-0.00158895,-0.00000003,-0.26456910,-
0.27201761,0.00003942,0.30620440,0.27180732,-0.00000023,-0.00000040,-
0.00000512,0.,0.00000069,0.00013949,0.00000011,-0.00000034,0.00002650,-
0.00000007,0.00000023,-0.00000592,-0.00000005,-0.00000014,-
0.00002970,0.00000013,0.00000022,0.00003275,0.00000005,-
0.00000006,0.00000205,0.,0.,0.00000764,-0.00000003,-
0.00000005,0.00000564,0.00000003,-0.00000002,-
0.00000062,0.00000002,0.00000004,0.00000166,0.,0.,0.00000298,0.,0.,-
0.00000310,-0.00000001,0.00000002,-0.00001306,0.00000001,0.00000001,-
0.00000650,0.,-0.00000002,-0.00000217,0.,-0.00000005,0.00000477,-0.00000002,-
0.00000002,0.00000532,0.,0.00000002,0.00000368,-0.00000001,-
0.00000002,0.00000116,-0.00000005,-0.00000003,-
0.00000046,0.00000003,0.,0.00000329,0.00000002,0.00000001,-0.00000845,-
0.00000002,0.,-0.00000364,0.,0.00000002,-0.00000296,0.,0.00000001,-
0.00000196,-0.00000045,-0.00000065,0.00005914,-0.00000060,0.00000103,-
0.00056261,0.00000140,-0.00000226,0.00014289,-0.00000146,-
0.00000261,0.01900377,0.00000125,-0.00000197,0.00264420,-
0.00000096,0.00000019,-0.00156159,-0.00000009,-0.00000004,-0.00026610,-
0.00000027,-0.00000042,0.00064001,0.00000003,0.00000007,0.00003991,-
0.00000111,-0.00000009,-0.00033061,0.00010045,0.00004004,-0.07428025,-
0.00014091,-0.00004245,0.03212480,0.00072484,0.00129756,0.00000022,-
0.00009180,-0.00214575,-0.00000058,-
0.00026866,0.00116342,0.00000040,0.00021824,-0.00080935,-
0.00000033,0.00022949,0.00052337,0.00000014,-0.00064298,-0.00081303,-
0.00000014,-0.00007031,0.00009960,0.00000007,0.00000624,-0.00001938,-
0.00000004,0.00001431,0.00002862,0.00000002,-
0.00012407,0.00007239,0.00000003,0.00001335,-0.00000396,0.00000001,-
0.00006546,-0.00000814,0.00000002,0.00001564,-0.00002582,-
0.00000002,0.00004401,-0.00001462,0.00000001,0.00000299,-0.00001585,0.,-
0.00000323,0.00000885,0.,0.00011501,0.00011640,0.00000003,0.00004705,0.0000
9003,0.00000003,0.00006696,-0.00005730,-0.00000002,0.00002449,-
0.00002595,0.,0.00018209,0.00011587,-0.00000001,-
0.00006269,0.00000807,0.00000001,-
0.00008927,0.00000361,0.00000001,0.00001863,0.00000945,0.,-0.00002702,-
0.00004722,0.,0.00000374,0.00001063,0.,0.00021366,0.00082585,0.00000018,0.00
111496,0.00001715,-0.00000037,0.00138213,-0.00165638,-
0.00000042,0.00045956,0.01274853,0.00000563,-0.00412677,-
0.00109596,0.00000118,0.00074188,0.00107665,-
0.00000009,0.00007706,0.00025579,0.00000005,0.00137157,-0.00071559,-
0.00000054,-0.00001936,-0.00001468,-0.00000005,0.00098251,-0.00157165,-
0.00000056,-0.22707037,0.04516937,0.00006514,-
0.10381876,0.01433799,0.00004699,0.40395626,-0.00099429,-0.00196476,-
0.00000031,0.00035003,0.00348688,0.00000090,0.00058873,-0.00215493,-
0.00000091,-0.00048503,0.00139964,0.00000063,-0.00020547,-0.00085006,-
0.00000018,0.00084138,0.00123660,0.00000018,0.00013119,-0.00018707,-
0.00000013,0.00001347,-0.00001514,0.00000002,-0.00005478,-0.00003563,-
0.00000003,0.00023448,-0.00014903,-0.00000010,-

0.00000117,0.00001211,0.,0.00010999,0.00003756,-0.00000002,-
0.00002788,0.00005233,0.00000005,-
0.00010125,0.00002532,0.,0.00000815,0.00001458,0.00000002,0.00000478,-
0.00001369,0.,-0.00017137,-0.00017383,-0.00000003,-0.00003577,-0.00007028,-
0.00000002,-0.00010911,0.00006385,0.00000004,-
0.00001070,0.00002837,0.00000001,-0.00026839,-0.00015721,0.,0.00006648,-
0.00003051,-0.00000002,0.00011941,0.00000411,-0.00000001,-
0.00001356,0.00000017,0.,0.00003850,0.00006107,0.,-0.00000539,-
0.00001865,0.,0.00093474,-0.00155692,-0.00000067,0.00150140,-0.00191900,-
0.00000078,-0.00595611,-0.00468378,0.00000002,0.03938141,-0.03207981,-
0.00002274,0.00127945,-0.00464897,-
0.00000201,0.00041714,0.00043673,0.00000028,-0.00013400,-
0.00013180,0.,0.00019924,-0.00335272,-0.00000114,-0.00004213,-0.00064787,-
0.00000012,0.00003851,0.00047851,0.00000004,0.08712007,-0.17169499,-
0.00005544,-0.00539673,0.03641692,0.00000359,0.01218649,0.62625797,-
0.00000049,-
0.00000095,0.00003281,0.00000019,0.00000153,0.00007034,0.00000022,-
0.00000094,0.00000755,-0.00000023,0.00000065,-0.00000821,-0.00000013,-
0.00000038,-0.00000599,0.00000046,0.00000058,-0.00000620,0.00000006,-
0.00000008,0.00000308,0.,-
0.00000003,0.00000513,0.00000002,0.,0.00000322,0.00000012,-0.00000006,-
0.00000060,-0.00000002,-
0.00000003,0.00000154,0.00000007,0.00000003,0.00000318,-
0.00000002,0.00000002,-0.00000297,-0.00000004,0.00000002,-0.00000740,-
0.00000004,-0.00000001,-0.00000363,0.,0.00000002,-0.00000205,-0.00000011,-
0.00000005,0.00000177,0.,-0.00000003,0.00000877,-
0.00000006,0.,0.00000284,0.,0.00000005,0.00000212,-0.00000014,-0.00000007,-
0.00000096,0.,-0.00000001,0.00000552,0.00000005,0.,-0.00001380,0.00000003,-
0.00000002,-0.00000674,0.00000003,0.00000002,-0.00000325,0.,-0.00000003,-
0.00000233,0.00000024,-0.00000071,0.00008307,-0.00000005,-0.00000044,-
0.00025428,-0.00000158,-0.00000097,0.00156301,0.00001265,-
0.00001469,0.00861217,0.00000171,-0.00000109,0.00091420,-
0.00000032,0.00000007,-0.00077838,-0.00000006,-0.00000009,-0.00001117,-
0.00000023,-0.00000089,0.00044259,-0.00000004,-0.00000012,-0.00017213,-
0.00000025,0.00000061,0.00013882,0.00007429,-0.00004130,-
0.06111108,0.00004231,-0.00000268,0.02876062,-
0.00011718,0.00013442,0.03815367,0.00165431,-0.00119952,-0.00000064,-
0.00176281,0.00327871,0.00000100,0.00306242,-0.00444536,-0.00000197,-
0.00727084,0.00466795,0.00000319,0.00478017,-0.00103572,-0.00000156,-
0.00333978,0.00044426,0.00000099,0.00189045,-0.00388260,-
0.00000243,0.00412683,-0.00504103,-
0.00000239,0.01044076,0.01427705,0.00000751,-
0.17237338,0.04956866,0.00003947,-0.03493859,0.01392340,0.00001194,-
0.00292822,-0.00419768,-0.00000162,-
0.00021559,0.00061865,0.00000044,0.00096317,-0.00092430,-0.00000063,-
0.00028095,-0.00403383,-0.00000288,-
0.00010511,0.00096533,0.00000038,0.00051293,0.00005748,-
0.00000002,0.00004425,0.00026948,0.00000007,0.00011287,-0.00002040,-
0.00000007,-0.00000867,-0.00013942,-0.00000001,0.00057217,0.00029020,-
0.00000006,-0.00002651,0.00002926,0.00000003,-
0.00014970,0.00000936,0.00000002,0.00003230,-0.00000555,-0.00000002,-

0.00014150,-0.00017310,0.00000002,-0.00001066,0.00003397,0.,0.00015366,-
0.00053923,-0.00000011,0.00011303,-0.00017053,-
0.00000008,0.00013272,0.00010465,0.,0.00001423,-0.00071454,-0.00000023,-
0.00007773,0.00000118,0.00000003,-0.00026043,-0.00013660,0.,-
0.00007966,0.00015071,0.00000007,0.00003023,-0.00003368,-
0.00000002,0.00013444,0.00024594,0.00000003,-0.00001515,-0.00002976,-
0.00000001,0.00008264,0.00151916,0.00000041,-0.00032254,-0.00071961,-
0.00000011,0.00027790,-0.00055221,-0.00000022,0.60632986,-
0.00092059,0.00050564,0.00000023,0.00076677,-0.00138753,-0.00000035,-
0.00135419,0.00173756,0.00000078,0.00330362,-0.00253302,-0.00000112,-
0.00291979,0.00070376,0.00000103,0.00210277,-0.00010548,-0.00000059,-
0.00250100,-0.00236980,-0.00000058,-0.00245397,-0.00556962,-
0.00000777,0.02761281,-0.01945458,-0.00001444,0.03740815,-0.12516364,-
0.00003214,0.00530223,0.01917284,0.00000973,-0.00854655,0.00131696,-
0.00000239,-0.00134872,0.00081963,0.00000039,0.00102365,-0.00023372,-
0.00000041,-0.00035858,-0.00053474,0.00000201,-0.00433378,-
0.00228046,0.00000140,-0.00031858,-0.00003092,0.00000007,0.00001074,-
0.00016927,-0.00000007,-
0.00015705,0.00001624,0.00000005,0.00004903,0.00010974,0.00000002,-
0.00037246,-0.00016927,0.00000002,0.00000230,-0.00005511,-
0.00000003,0.00009853,-0.00001068,0.,-
0.00001604,0.00000241,0.,0.00010649,0.00013681,0.,-0.00002348,-0.00002655,0.,-
0.00006631,0.00024333,0.00000008,-0.00001965,0.00000252,-0.00000001,-
0.00005611,0.00000217,0.,0.00000965,0.00030851,0.00000010,0.00000905,-
0.00002737,-0.00000004,0.00010083,0.00009767,0.00000003,0.00002942,-
0.00006486,-0.00000003,-0.00001717,0.00001785,0.00000002,-0.00004277,-
0.00008675,0.,0.00001404,-0.00001408,0.,0.00010003,-0.00073701,-
0.00000027,0.00006448,0.00033234,0.00000009,-
0.00021903,0.00030730,0.00000016,-0.01069708,0.99411581,-
0.00000074,0.00000026,0.00015197,0.00000082,-0.00000115,-0.00009640,-
0.00000124,0.00000154,0.00008920,0.00000250,-0.00000138,-0.00120877,-
0.00000199,0.00000038,0.00009204,0.00000149,0.00000011,-0.00007536,-
0.00000189,-0.00000070,-0.00077923,-0.00000089,-
0.00000732,0.00787024,0.00001505,-0.00001251,0.00006057,0.00003134,-
0.00003248,-0.07780658,0.00000743,0.00000841,0.00201443,-0.00000396,-
0.00000279,0.00714705,-0.00000077,0.00000025,0.00033613,0.00000056,-
0.00000027,0.00035356,-0.00000052,0.00000231,-0.00395536,-
0.00000316,0.00000076,-0.00389003,-0.00000029,0.,-0.00001710,0.,-0.00000013,-
0.00000557,-0.00000010,0.,-0.00000490,0.00000001,0.00000013,-0.00000454,-
0.00000027,-0.00000012,0.00000059,0.,-
0.00000005,0.00000022,0.00000007,0.,0.00000259,0.00000001,0.,0.00000240,0.00
000009,0.00000008,0.00001132,0.,-0.00000003,0.00000508,-
0.00000008,0.00000019,-0.00001691,0.,0.00000004,-0.00000641,-0.00000005,-
0.00000005,-0.00000413,0.,0.00000027,0.00000049,-0.00000002,-
0.00000002,0.00000047,0.00000007,0.00000006,-0.00000663,0.00000005,-
0.00000005,0.00000281,0.,0.00000001,0.00000245,-0.00000004,-
0.00000008,0.00001214,0.00000003,0.,0.00000515,0.00000007,-
0.00000054,0.00000620,0.00000005,0.00000026,-0.00000775,-
0.00000015,0.00000023,-0.00000538,-0.00003878,0.00048956,0.20029332,-
0.00058179,0.00039511,0.00000024,0.00064805,-0.00109359,-0.00000039,-
0.00111986,0.00162270,0.00000071,0.00253430,-0.00148536,-0.00000104,-

0.00166107,0.00035021,0.00000055,0.00112773,-0.00015913,-0.00000033,-
0.00070989,0.00197181,0.00000118,-0.00031420,0.00251926,0.00000128,-
0.00652185,-0.00115762,0.00000030,0.00818945,0.03501561,0.00002243,-
0.00337012,0.00127585,0.00000128,0.00202917,0.00016048,-0.00000014,-
0.00000810,-0.00011576,-0.00000006,-
0.00046494,0.00031308,0.00000021,0.00015282,0.00092637,0.00000060,0.000239
88,0.00222965,0.00000114,-0.00018619,-0.00002212,0.00000002,-0.00001999,-
0.00009184,-0.00000003,-0.00001565,-0.00000186,0.00000002,-
0.00000488,0.00005270,0.00000001,-0.00020197,-
0.00010841,0.00000002,0.00001097,-0.00000304,0.,0.00004913,-0.00000178,0.,-
0.00001113,0.00000286,0.,0.00005623,0.00005797,-0.00000001,0.00000825,-
0.00001111,0.,-0.00005654,0.00019484,0.00000004,-
0.00002708,0.00004414,0.00000001,-0.00005030,-0.00003060,0.,-
0.00000144,0.00025184,0.00000008,0.00002257,-
0.00000392,0.,0.00008850,0.00005069,0.,0.00002513,-0.00005027,-0.00000002,-
0.00001132,0.00001159,0.,-0.00004286,-0.00008783,0.,0.00000622,0.00000610,0.,-
0.00001627,-0.00054758,-0.00000014,0.00010688,0.00025846,0.00000003,-
0.00010848,0.00020368,0.00000008,-
0.11798624,0.00221771,0.00000518,0.10983313,0.00080755,-0.00026559,-
0.00000021,-0.00074187,0.00114850,0.00000032,0.00126584,-0.00146521,-
0.00000062,-0.00256283,0.00172726,0.00000116,0.00210425,-0.00039965,-
0.00000071,-0.00150260,-0.00002568,0.00000039,0.00005523,-0.00076413,-
0.00000044,0.00190611,-0.00155473,-
0.00000003,0.00524131,0.00418230,0.00000036,0.01968379,-0.04930621,-
0.00004346,-0.00811228,-0.00405353,-0.00000167,0.00143256,-
0.00023825,0.00000005,0.00033899,-0.00010681,-0.00000012,0.00045040,-
0.00030105,-0.00000009,-
0.00028237,0.00098070,0.00000011,0.00243879,0.00105669,0.00000040,0.000242
85,0.00002762,-0.00000003,-0.00000253,0.00013070,0.00000005,0.00012355,-
0.00001776,-0.00000004,-0.00003544,-0.00008051,0.,0.00028557,0.00013306,-
0.00000002,-0.00000557,0.00003983,0.00000002,-
0.00007706,0.00000866,0.,0.00001222,-0.00000207,0.,-0.00007396,-
0.00010109,0.,0.00001714,0.00002043,0.,0.00005905,-0.00019833,-0.00000005,-
0.00000319,0.00000845,0.00000002,0.00005317,-0.00000008,0.,-0.00000771,-
0.00024890,-0.00000008,-0.00000488,0.00001936,0.00000002,-0.00007981,-
0.00007443,-0.00000002,-0.00002081,0.00004885,0.00000002,0.00001386,-
0.00001429,-0.00000002,0.00003213,0.00008515,0.,-0.00001121,0.00001187,0.,-
0.00007438,0.00059191,0.00000020,-0.00005476,-0.00026795,-
0.00000007,0.00017305,-0.00024682,-0.00000012,0.00248120,-0.72657658,-
0.00040187,0.02537120,0.84639118,0.00000054,-0.00000020,-0.00002977,-
0.00000055,0.00000083,0.00003293,0.00000091,-0.00000106,-0.00000527,-
0.00000175,0.00000112,0.00013942,0.00000138,-0.00000028,0.00002642,-
0.00000102,-0.00000003,-0.00000582,0.00000014,-
0.00000067,0.00005790,0.00000098,-0.00000032,-
0.00156079,0.00000410,0.00000103,0.00264535,0.00001309,-
0.00004501,0.01901177,-0.00000461,-
0.00000251,0.00013319,0.00000058,0.00000011,-0.00056290,0.00000022,-
0.00000008,0.00003919,0.00000029,-0.00000005,-0.00026624,-
0.00000012,0.00000016,0.00064217,0.00000142,0.00000067,-
0.00035062,0.00000022,0.00000001,0.00000196,0.,0.00000009,0.00000296,0.0000
0007,0.,0.00000765,0.,-0.00000010,0.00000561,0.00000019,0.00000009,-

0.00000059,0.,0.00000004,0.00000167,-0.00000005,0.,-0.00000309,-
0.00000001,0.,-0.00000216,-0.00000006,-0.00000005,-0.00001306,0.,0.00000003,-
0.00000649,0.00000006,-0.00000014,0.00000471,0.,-
0.00000002,0.00000531,0.00000005,0.00000003,0.00000328,-0.00000001,-
0.00000018,-0.00000041,0.00000001,0.00000001,0.00000111,-0.00000004,-
0.00000004,0.00000368,-0.00000003,0.00000003,-0.00000294,0.,0.,-
0.00000193,0.00000002,0.00000006,-0.00000844,-0.00000002,0.,-0.00000363,-
0.00000006,0.00000038,-0.00000549,-0.00000003,-
0.00000018,0.00000628,0.00000011,-0.00000017,0.00000474,0.00000534,-
0.00040148,-0.07412535,0.00000936,0.00050001,0.03200582,-0.00013848,-
0.00026124,-0.00000006,0.00006775,0.00042802,0.00000011,0.00006916,-
0.00024747,-0.00000008,-0.00003144,0.00015388,0.00000008,-0.00003764,-
0.00010292,-0.00000005,0.00012739,0.00017394,0.00000005,0.00001458,-
0.00002127,-0.00000002,-0.00000166,0.00000188,0.,-0.00000236,-
0.00000545,0.,0.00002563,-0.00001480,0.,-
0.00000294,0.00000130,0.,0.00001408,0.00000186,0.,-0.00000317,0.00000531,0.,-
0.00000789,0.00000177,0.,-0.00000069,0.00000317,0.,0.00000066,-0.00000181,0.,-
0.00002619,-0.00002558,-0.00000001,-0.00000732,-0.00000962,0.,-
0.00001381,0.00001058,0.,-0.00000329,0.00000429,0.,-0.00004043,-
0.00002357,0.,0.00001358,-0.00000497,0.,0.00001967,0.00000156,0.,-0.00000238,-
0.00000105,0.,0.00000532,0.00000867,0.,-0.00000043,-0.00000235,0.,-
0.00014151,-0.00034219,-0.00000005,-
0.00043832,0.00023441,0.00000003,0.00070405,0.00126476,0.00000030,-
0.00632435,0.00174235,0.00000418,-0.00063779,-0.00068971,-
0.00000016,0.00032781,-0.00012202,-0.00000011,-0.00001118,-
0.00001382,0.,0.00070457,0.00010669,-0.00000020,0.00003043,0.00011219,0.,-
0.00009874,0.00012610,0.00000012,0.01208091,0.05390491,0.00001032,0.000611
74,-0.00802894,-0.00000418,-0.07553728,-0.13154905,-0.00001146,-
0.00005667,0.00004739,0.00000003,0.00002233,-0.00003714,-
0.00000002,0.06868888,0.00014132,0.00030336,0.00000005,-0.00003992,-
0.00053081,-0.00000020,-0.00009697,0.00030752,0.00000022,0.00007974,-
0.00021452,-0.00000017,0.00001407,0.00013001,0.00000004,-0.00012577,-
0.00020482,-0.00000004,-0.00002295,0.00002900,0.00000004,-
0.00000076,0.00000354,0.,0.00000785,0.00000462,0.,-
0.00004034,0.00002537,0.00000001,0.00000154,-0.00000242,0.,-0.00001791,-
0.00000530,0.,0.00000464,-0.00000909,0.,0.00001435,-0.00000180,0.,-
0.00000111,-0.00000333,-0.00000001,-
0.00000043,0.00000227,0.,0.00002859,0.00002690,0.00000002,0.00000150,0.0000
0390,0.,0.00001794,-0.00000756,0.,-0.00000170,-
0.00000345,0.,0.00004493,0.00002471,0.,-0.00000801,0.00000647,0.,-0.00001715,-
0.00000092,0.,0.00000015,-0.00000211,0.,-0.00000614,-
0.00000899,0.,0.00000044,0.00000329,0.,-
0.00001015,0.00028956,0.00000010,0.00058676,0.00003932,-0.00000004,-
0.00058025,-0.00096714,-0.00000025,0.00417075,0.00113695,-
0.00000225,0.00026507,0.00083919,0.00000033,-
0.00013943,0.00005660,0.,0.00001239,-0.00000393,-0.00000001,-0.00013285,-
0.00000645,0.00000009,-0.00004844,-0.00008200,0.00000001,0.00015765,-
0.00010136,-0.00000011,0.00170012,-0.03867528,-0.00001098,-
0.00808541,0.00478110,0.00000539,-0.08103580,-0.44361189,-
0.00007242,0.00009665,-0.00005532,-0.00000004,-
0.00003629,0.00004444,0.00000003,0.08316101,0.47645160,0.00000008,0.000000

18,-0.00001985,-0.00000006,-0.00000026,-0.00000447,-0.00000005,0.00000017,-
0.00002519,0.00000005,-0.00000012,0.00003435,0.,0.00000007,-0.00001886,-
0.00000009,-0.00000012,0.00001746,0.,0.,-0.00000023,0.,0.00000001,-
0.00000405,-0.00000002,-0.00000001,-0.00000255,-
0.00000003,0.,0.00000037,0.00000002,0.00000003,-0.00000144,-0.00000002,-
0.00000001,-
0.00000205,0.,0.,0.00000225,0.,0.,0.00000552,0.00000002,0.,0.00000315,0.,-
0.00000001,0.00000157,0.00000003,0.,-0.00000108,-0.00000001,0.,-
0.00000539,0.00000002,0.00000001,-0.00000208,0.,-0.00000002,-
0.00000161,0.00000003,0.,0.00000055,0.00000001,0.,-
0.00000346,0.,0.,0.00000811,-0.00000002,0.,0.00000434,-
0.00000001,0.,0.00000231,0.,0.00000001,0.00000173,0.,0.00000017,-
0.00000590,0.00000022,0.00000009,-0.00042688,-0.00000019,-
0.00000090,0.00045835,0.00000482,-
0.00000170,0.00539267,0.00000011,0.00000056,-0.00045723,-0.00000004,-
0.00000005,0.00015826,0.00000001,0.,0.00002948,-0.00000027,0.00000005,-
0.00023496,-0.00000004,-0.00000004,-0.00005063,0.00000012,-
0.00000011,0.00017588,-0.00000180,-0.00002793,0.00459541,-
0.00000432,0.00000529,-0.00657053,0.00000023,-0.00005558,-
0.01642916,0.00000004,-0.00000002,0.00000457,-0.00000001,0.00000001,-
0.00000397,0.00000118,0.00008020,0.01337885,0.00343391,0.00369439,0.000000
48,-0.00201847,-0.00286647,-0.00000045,0.00171171,0.00088832,-0.00000012,-
0.00323267,0.00008202,0.00000091,0.00457922,0.00047702,-0.00000092,-
0.00716729,-0.00336063,0.00000017,0.00048333,-0.00012356,-
0.00000017,0.00002378,-0.00026507,-0.00000010,-0.00004454,-0.00002849,-
0.00000002,0.00054051,-0.00031311,-
0.00000019,0.00001337,0.00012485,0.00000005,0.00011248,0.00001959,-
0.00000009,-0.00011948,0.00018927,0.00000017,-
0.00014644,0.00000931,0.,0.00004298,0.00000987,-0.00000002,-0.00000749,-
0.00001829,0.,0.00146028,0.00274960,0.00000030,0.00576389,0.00351528,0.0000
0120,0.00011550,0.00478373,0.00000244,-0.03014857,-0.00419639,0.00000435,-
0.17452495,-0.03612067,0.00000845,-0.00267830,-0.02392235,-
0.00000513,0.00087640,0.00071821,0.00000007,-
0.00028605,0.00444861,0.00000049,0.00002293,-0.00048138,-
0.00000003,0.00141939,-0.00063497,-
0.00000105,0.00021910,0.00048900,0.00000006,-
0.00023762,0.00015295,0.00000005,-
0.00006194,0.00001794,0.00000006,0.00005161,0.00067270,0.00000015,0.000120
25,-0.00012280,-0.00000005,0.00011685,0.00015481,0.00000003,0.00009556,-
0.00024386,-0.00000010,-0.00002727,0.00003316,0.00000002,-0.00009158,-
0.00013210,0.,0.00001904,0.00002344,0.,0.00016886,-0.00146869,-
0.00000042,0.00017048,0.00065485,0.00000011,-
0.00041016,0.00059378,0.00000027,-
0.00123363,0.00065860,0.00000053,0.00045242,-0.00051118,-
0.00000038,0.00009444,-0.00010208,-
0.00000005,0.90663364,0.00181072,0.00204658,0.00000018,-0.00120813,-
0.00142781,-0.00000012,0.00116658,0.00044921,-0.00000021,-
0.00242940,0.00008269,0.00000074,0.00356798,0.00021968,-0.00000081,-
0.00472273,-0.00263337,0.00000055,0.00034914,-0.00008612,-0.00000015,-
0.00002764,-0.00018632,-0.00000007,-0.00002025,-0.00006083,-
0.00000004,0.00041083,-0.00021719,-0.00000017,-

0.00003615,0.00012106,0.00000010,0.00017101,0.00000316,-0.00000006,-
0.00009945,0.00016705,0.00000012,-
0.00011197,0.00000210,0.,0.00002623,0.00000511,0.,0.00002541,-0.00002175,-
0.00000002,0.00413226,-0.00194334,-0.00000108,-0.00084382,-0.00738690,-
0.00000332,0.00737735,-0.00153576,-
0.00000361,0.00910066,0.02107607,0.00000346,-0.04826663,-0.12270512,-
0.00000224,-0.03255439,-0.01310483,0.00000206,-0.00124995,-
0.00027290,0.00000007,-0.00086698,-
0.00219519,0.00000051,0.00146834,0.00070652,-0.00000014,0.00306975,-
0.00227596,-0.00000017,0.00011303,0.00026345,0.00000002,-
0.00010691,0.00011236,0.00000007,-0.00000891,-0.00002000,-
0.00000002,0.00001033,0.00035088,0.00000011,0.00006542,-0.00001805,-
0.00000003,0.00001775,0.00001195,0.00000002,0.00003575,-0.00010086,-
0.00000005,-0.00002442,-0.00001005,0.,-0.00004252,-
0.00006831,0.,0.00001304,0.00001541,0.,-0.00005055,-0.00082385,-
0.00000023,0.00018251,0.00040524,0.00000007,-
0.00014015,0.00029789,0.00000013,-
0.00075950,0.00057849,0.00000039,0.00027228,-0.00044232,-
0.00000026,0.00003341,-0.00006167,-0.00000003,-0.16277229,0.69393512,-
0.00000018,-0.00000020,0.00009202,0.00000011,0.00000020,-0.00007507,-
0.00000003,-0.00000004,0.00015179,-0.00000009,0.,-0.00009605,0.00000013,-
0.00000005,0.00008912,-0.00000008,0.00000029,-0.00120882,0.,0.,-
0.00001708,0.,-0.00000001,-
0.00000661,0.00000002,0.,0.00000041,0.00000002,0.,0.00000057,-0.00000003,-
0.00000001,-0.00000412,0.00000005,0.,-
0.00000645,0.,0.,0.00001218,0.,0.,0.00000283,-0.00000003,-
0.00000002,0.00000249,0.,0.,0.00000516,0.00000055,-0.00000078,-0.00077970,0.,-
0.00000421,0.00714702,0.00000318,-
0.00000319,0.00786978,0.00000740,0.00000596,0.00007243,0.00000539,-
0.00000471,-0.07779797,-0.00000711,0.00000055,0.00200856,-0.00000040,-
0.00000027,0.00033593,-0.00000084,-0.00000036,-
0.00382684,0.00000043,0.00000018,0.00035387,-0.00000022,0.00000054,-
0.00396614,-0.00000004,-0.00000009,-0.00001726,0.00000004,0.00000001,-
0.00000557,0.00000004,-0.00000005,0.00000018,0.,-0.00000003,0.00000066,-
0.00000006,0.,-0.00000455,-0.00000004,-0.00000004,-
0.00000491,0.00000003,0.00000003,0.00001134,0.,0.,0.00000511,0.,0.,0.00000261
,0.00000002,0.,0.00000244,0.,0.00000013,0.00000616,-0.00000002,-0.00000003,-
0.00000544,0.00000004,-0.00000003,-
0.00000802,0.00000005,0.00000008,0.00000617,-0.00000002,-0.00000005,-
0.00000772,-0.00000001,0.,0.00000572,-0.00016051,0.00014753,0.20031556,-
0.00177697,-0.00203613,-0.00000026,0.00098310,0.00158670,0.00000028,-
0.00086590,-0.00064402,-0.00000001,0.00181817,0.00006621,-0.00000044,-
0.00262907,-0.00039359,0.00000050,0.00368079,0.00189973,-0.00000013,-
0.00027307,0.00006568,0.00000008,-
0.00002111,0.00014319,0.00000005,0.00002405,0.00000422,0.,-
0.00029296,0.00017577,0.00000013,-0.00002220,-0.00006937,-0.00000003,-
0.00001816,-0.00000178,0.00000005,0.00007728,-0.00010146,-
0.00000010,0.00007365,-0.00000728,0.,-0.00002453,-
0.00000622,0.00000001,0.00001266,0.00000745,0.,-
0.00162735,0.00047529,0.00000041,-0.00035763,-0.00005504,-0.00000009,-
0.00316075,-0.00026205,0.00000025,-0.00026526,-0.00885014,-0.00000164,-

0.05860328,0.01892174,0.00001787,-0.00093687,0.00669140,0.00000184,-
0.00017911,-0.00024106,-0.00000002,-0.00119016,-0.00163386,-0.00000025,-
0.00067348,-0.00033001,-0.00000003,0.00049445,0.00008811,0.00000004,-
0.00012192,-0.00028029,-0.00000006,0.00013403,-0.00008688,-
0.00000003,0.00003646,-0.00000908,-0.00000003,-0.00002737,-0.00038623,-
0.00000008,-0.00006943,0.00007238,0.00000003,-0.00006968,-0.00009239,-
0.00000001,-0.00005458,0.00013814,0.00000006,0.00001477,-
0.00002096,0.,0.00005265,0.00007506,0.,-0.00001077,-0.00001230,0.,-
0.00011501,0.00083899,0.00000024,-0.00008595,-0.00036954,-
0.00000006,0.00024599,-0.00034472,-0.00000016,0.00070942,-0.00034408,-
0.00000028,-0.00026253,0.00026778,0.00000020,-
0.00005685,0.00005859,0.00000003,-
0.57684581,0.26235030,0.00014919,0.64069749,-0.00028274,-0.00028263,-
0.00000001,0.00024319,0.00011965,-0.00000003,-
0.00023116,0.00001826,0.00000008,0.00041778,-0.00002176,-0.00000013,-
0.00075059,0.00004456,0.00000018,0.00082171,0.00057884,-0.00000008,-
0.00006990,0.00001793,0.00000004,0.00001270,0.00003502,0.,0.00000299,0.0000
1787,0.00000001,-0.00008363,0.00004237,0.00000004,0.00001455,-0.00002791,-
0.00000003,-0.00004925,-0.00000052,0.,0.00001847,-0.00003499,-
0.00000002,0.00002220,0.00000031,0.,-0.00000390,-0.00000103,0.,-
0.00000983,0.00000550,0.,-
0.00143949,0.00015303,0.00000029,0.00122096,0.00215002,0.00000044,-
0.00087302,0.00129410,0.00000086,-0.00245849,-0.00207780,-
0.00000073,0.00352706,0.01750012,-0.00000097,-0.00264732,-0.00647037,-
0.00000121,0.00021455,0.00006407,-0.00000003,-
0.00141244,0.00252411,0.00000101,-0.00019339,-0.00009342,0.00000007,-
0.00112031,0.00063989,0.00000020,-0.00001521,-0.00003694,0.,0.00000287,-
0.00002303,-0.00000002,-0.00001172,0.00001446,0.00000002,0.00000758,-
0.00004142,-0.00000002,-0.00001539,-
0.00001637,0.,0.00003322,0.00003656,0.,0.00000613,0.00001001,0.,0.00000730,0.
00001393,0.,-0.00000032,0.00000483,0.,-0.00000300,-
0.00000233,0.,0.00005538,0.00012505,0.00000003,-0.00005668,-0.00007517,-
0.00000001,-0.00000860,-0.00003450,-0.00000001,0.00012862,-0.00013350,-
0.00000007,-
0.00004523,0.00010137,0.00000005,0.00000074,0.00001162,0.,0.26261238,-
0.26811904,-0.00009172,-
0.33180656,0.31587381,0.00000024,0.00000028,0.00002658,-0.00000013,-
0.00000026,-0.00000615,0.00000010,0.00000012,-0.00002956,-0.00000021,-
0.00000003,0.00003252,0.00000027,0.00000009,-0.00000507,-0.00000045,-
0.00000025,0.00013925,0.00000005,-0.00000002,0.00000495,-0.00000001,-
0.00000001,0.00000366,-0.00000002,-0.00000003,0.00000119,0.00000003,-
0.00000002,-0.00000053,0.00000002,0.00000003,0.00000330,-
0.00000003,0.,0.00000533,0.,0.00000001,-0.00000847,0.,0.,-
0.00000297,0.00000003,0.00000001,-0.00000198,0.,-0.00000001,-0.00000366,0.,-
0.00000005,0.00005792,0.00000025,0.00000067,-
0.00056257,0.00000005,0.00000077,-0.00156270,-
0.00000006,0.00000031,0.00264395,0.00001429,-0.00000358,0.01901802,-
0.00000045,-0.00000274,0.00012956,0.00000009,0.00000005,0.00003926,-
0.00000021,0.00000100,-0.00036554,0.00000002,0.00000010,-0.00026603,-
0.00000025,0.,0.00064228,0.00000004,0.00000007,0.00000218,-
0.00000003,0.,0.00000296,-

0.00000002,0.00000004,0.00000173,0.00000001,0.00000006,-
0.00000069,0.00000004,-
0.00000002,0.00000565,0.00000003,0.00000004,0.00000767,-0.00000002,-
0.00000003,-0.00001306,0.,0.00000001,-0.00000652,0.,-0.00000001,-0.00000312,-
0.00000002,0.,-0.00000222,0.,-0.00000016,-
0.00000769,0.00000002,0.00000006,0.00000622,-
0.00000005,0.00000006,0.00001014,-0.00000011,0.,-0.00000543,0.00000004,-
0.00000001,0.00000623,0.,0.,-0.00000713,0.00014905,-0.00009162,-0.07422543,-
0.00018423,0.00012532,0.03212932,-0.00092379,-0.00096606,-
0.00000013,0.00054349,0.00067200,0.00000012,-0.00044215,-
0.00018216,0.00000001,0.00077808,-0.00002061,-0.00000020,-0.00115957,-
0.00009446,0.00000028,0.00181287,0.00074958,-0.00000014,-
0.00012516,0.00003249,0.00000003,-
0.00000730,0.00006584,0.00000003,0.00001156,0.00000340,0.00000001,-
0.00013836,0.00008331,0.00000004,-0.00000618,-0.00003401,0.,-0.00001747,-
0.00000138,0.00000003,0.00003410,-0.00004811,-0.00000005,0.00003519,-
0.00000246,0.,-0.00001085,-0.00000302,0.,0.00000300,0.00000451,0.,-
0.00035271,-0.00126024,-0.00000023,-0.00050201,-0.00243817,-
0.00000058,0.00116310,-0.00017613,-0.00000038,-0.00443958,-
0.00148084,0.00000057,-0.00131838,-0.04056946,-0.00000811,-
0.00646485,0.00141531,0.00000177,-0.00051523,-0.00024454,0.00000001,-
0.00005689,-0.00063964,-0.00000013,-0.00002682,0.00007419,0.00000002,-
0.00239124,-0.00248470,-0.00000016,-0.00005799,-0.00012394,-
0.00000001,0.00005597,-0.00003978,-0.00000004,0.00001037,-0.00000078,0.,-
0.00000991,-0.00016819,-0.00000004,-0.00003457,0.00002662,0.,-0.00001249,-
0.00002443,-0.00000001,-0.00002017,0.00006415,0.00000003,0.00000830,-
0.00000505,0.,0.00001997,0.00003030,0.,-0.00000520,-0.00000586,0.,-
0.00003345,0.00037188,0.00000009,-0.00004930,-0.00016932,-
0.00000001,0.00009756,-0.00014904,-0.00000007,0.00031710,-0.00017342,-
0.00000015,-0.00011726,0.00013467,0.00000011,-
0.00002276,0.00002700,0.00000002,-0.12811658,-
0.02996174,0.00000430,0.00515752,0.06839737,0.00002071,0.58109817,-
0.00154192,-0.00149865,-0.00000014,0.00099759,0.00107423,0.00000012,-
0.00091169,-0.00017808,0.00000014,0.00163471,-0.00018563,-0.00000050,-
0.00238735,-0.00007841,0.00000056,0.00324689,0.00158108,-0.00000025,-
0.00025496,0.00006656,0.00000009,0.00001442,0.00013821,0.00000005,0.000018
18,0.00004147,0.00000003,-0.00030097,0.00016324,0.00000012,0.00002473,-
0.00008693,-0.00000007,-0.00012764,-0.00000576,0.00000005,0.00006785,-
0.00011923,-0.00000008,0.00008316,-0.00000128,0.,-0.00001897,-0.00000408,0.,-
0.00001850,0.00001579,0.00000001,-0.00115415,-0.00098750,-0.00000005,-
0.00094666,-0.00030225,0.00000011,-
0.00083854,0.00001873,0.00000037,0.00096735,-0.00435324,-0.00000147,-
0.01373214,-0.03011543,-0.00000719,-0.00287358,0.00316765,0.00000090,-
0.00027206,-0.00015241,0.00000006,0.00096367,0.00151510,0.00000019,-
0.00031642,-0.00002892,0.00000004,-0.00173289,0.00051537,0.00000041,-
0.00009249,-0.00019941,-0.00000001,0.00007839,-0.00008381,-
0.00000005,0.00000223,0.00001516,0.,-0.00000655,-0.00026311,-0.00000007,-
0.00005678,0.00001323,0.00000001,0.00000855,0.00000331,-0.00000002,-
0.00002252,0.00008953,0.00000005,0.00001894,0.00000919,0.,0.00002828,0.0000
4812,0.,-0.00001002,-0.00001173,0.,0.00003937,0.00061919,0.00000016,-
0.00013900,-0.00030617,-0.00000004,0.00010377,-0.00022376,-

0.00000010,0.00056030,-0.00042956,-0.00000030,-
0.00020121,0.00032890,0.00000021,-
0.00002503,0.00004672,0.00000003,0.01185393,-0.27035887,-
0.00005305,0.04867050,-0.07256750,-0.00003301,0.09066929,0.44881885,-
0.00000023,-0.00000021,0.00000733,0.00000018,0.00000003,-0.00000819,-
0.00000016,0.,-0.00000598,0.00000026,-0.00000005,-0.00000635,-
0.00000039,0.00000002,0.00003304,0.00000052,0.00000022,0.00007055,-
0.00000006,0.00000005,0.00000177,0.,0.00000002,0.00000286,0.00000006,0.0000
0005,0.00000211,-0.00000003,0.00000003,-0.00000091,-0.00000002,-
0.00000007,0.00000555,-0.00000003,0.,0.00000873,0.,-0.00000002,-
0.00001380,0.00000001,0.,-0.00000325,-0.00000004,-0.00000001,-
0.00000233,0.,0.00000003,-0.00000675,-0.00000021,0.,0.00008156,-
0.00000015,0.00000042,-0.00025394,-0.00000057,0.00000020,-
0.00078026,0.00000119,-0.00000101,0.00089131,-0.00000149,-
0.00000235,0.00864767,0.00000063,0.00000015,0.00156410,-
0.00000001,0.00000004,-0.00017210,0.00000030,0.00000043,0.00014023,-
0.00000007,-0.00000003,-
0.00001150,0.00000005,0.00000044,0.00041953,0.00000002,0.00000007,0.000003
09,0.00000002,0.,0.00000324,-0.00000007,0.,0.00000149,0.00000003,-
0.00000002,-0.00000066,0.,0.,0.00000328,-0.00000002,0.00000002,0.00000509,-
0.00000001,0.00000001,-0.00000743,0.00000003,-0.00000002,-
0.00000364,0.00000002,0.,-0.00000298,0.,0.,-0.00000203,0.,0.00000005,-
0.00000541,-0.00000002,-0.00000004,0.00000478,0.00000002,-
0.00000002,0.00000682,0.00000010,-0.00000012,-0.00000793,-
0.00000003,0.00000008,0.00001001,0.,0.00000002,-0.00000493,0.00001450,-
0.00004614,-0.06104248,0.00001582,-0.00003632,0.02875468,-
0.00007063,0.00008477,0.03812401,0.00009858,0.00011342,0.00000002,-
0.00006757,-0.00010037,-0.00000005,0.00004945,0.00003606,0.00000003,-
0.00010055,-0.00000589,0.,0.00014038,0.00002020,-0.00000003,-0.00021249,-
0.00012209,0.,0.00001486,-0.00000477,0.,0.00000307,-0.00000783,0.,-
0.00000229,0.00000221,0.,0.00001770,-0.00001141,-
0.00000001,0.00000254,0.00000171,0.,-0.00000244,0.00000126,0.,-
0.00000249,0.00000430,0.,-0.00000432,0.00000043,0.,0.00000160,0.00000073,0.,-
0.00000255,-0.00000027,0.,0.00002955,0.00010923,0.00000002,0.00027543,-
0.00017462,-0.00000015,0.00001181,-
0.00004402,0.00000002,0.00028662,0.00026879,-
0.00000006,0.00183828,0.00054557,0.00000071,-
0.00025189,0.00002799,0.00000013,-0.00002621,0.00001558,0.,0.00002287,-
0.00008747,0.,-0.00000661,-0.00000952,0.,0.00015473,-0.00018284,-
0.00000011,0.00000634,0.00001831,0.00000002,-
0.00000735,0.00000530,0.00000002,-0.00000156,-
0.00000053,0.,0.00000154,0.00002624,0.,0.00000406,-
0.00000417,0.,0.00000279,0.00000339,0.,0.00000362,-0.00000768,0.,-
0.00000167,0.00000044,0.,-0.00000342,-
0.00000466,0.,0.00000091,0.00000053,0.,0.00000623,-
0.00006015,0.,0.00000732,0.00002761,0.,-0.00001620,0.00002510,0.00000002,-
0.00005225,0.00001712,0.00000002,0.00002011,-0.00001396,-
0.00000002,0.00000386,-0.00000511,0.,-0.00185916,-0.00975120,-0.00000122,-
0.00327009,0.00586168,0.00000084,-0.44363599,-
0.13186066,0.00004054,0.44649913,0.00026454,0.00027775,0.00000003,-
0.00017480,-0.00017832,-0.00000004,0.00015335,0.00004267,0.,-

0.00030585,0.00002363,0.00000006,0.00042258,0.00002387,-0.00000008,-
0.00058994,-0.00024989,0.00000004,0.00004934,-0.00001424,0.,-0.00000080,-
0.00002451,0.,-0.00000367,-0.00000451,0.,0.00005713,-0.00003340,-0.00000003,-
0.00000140,0.00001746,0.00000002,0.00001252,-0.00000103,0.,-
0.00001444,0.00002127,0.00000001,-
0.00001423,0.00000062,0.,0.00000348,0.00000132,0.,0.00000094,-
0.00000192,0.,0.00044112,0.00011983,-0.00000004,0.00017807,-0.00067488,-
0.00000008,-0.00006070,0.00037337,0.00000006,0.00122378,-0.00008483,-
0.00000013,0.00297553,-0.00701917,-0.00000356,-0.00181756,-
0.00001074,0.00000021,-0.00014500,-0.00002529,0.00000003,-0.00005456,-
0.00022336,-0.00000009,0.00001665,-0.00000871,-0.00000001,-
0.00042198,0.00054265,0.00000027,0.00001649,0.00003742,0.00000001,-
0.00001426,0.00001613,0.00000002,-0.00000044,-
0.00000399,0.,0.00000090,0.00004939,0.,0.00000912,-0.00000165,0.,0.00000066,-
0.00000079,0.,0.00000489,-0.00001336,0.,-0.00000375,-0.00000226,0.,-
0.00000542,-0.00000896,0.,0.00000194,0.00000202,0.,-0.00001102,-0.00011878,-
0.00000003,0.00002845,0.00005970,0.,-0.00001779,0.00004238,0.00000003,-
0.00011819,0.00008516,0.00000006,0.00004373,-0.00006542,-
0.00000004,0.00000429,-0.00000950,0.,-0.06194511,-
0.02473112,0.00000342,0.00579766,0.00864682,0.00000275,-0.08135498,-
0.07577998,-0.00000071,0.13532330,0.09891251,0.00000006,0.00000006,-
0.00002512,-0.00000005,0.00000002,0.00003443,0.00000004,0.,-0.00001893,-
0.00000006,0.00000002,0.00001764,0.00000008,0.,-0.00002003,-0.00000013,-
0.00000003,-0.00000457,0.00000003,-0.00000002,-0.00000115,0.,0.,-0.00000209,-
0.00000003,-0.00000002,-0.00000162,0.,-
0.00000001,0.00000056,0.00000002,0.00000004,-0.00000349,0.,0.,-
0.00000537,0.,0.,0.00000811,0.,0.,0.00000232,0.00000002,0.,0.00000175,0.,-
0.00000001,0.00000436,0.00000011,0.,-0.00000661,-0.00000009,-0.00000003,-
0.00042619,0.00000002,0.00000008,0.00015873,0.00000017,0.00000003,-
0.00045735,0.00000138,-0.00000309,0.00539123,-0.00000029,-
0.00000012,0.00045881,-0.00000004,0.,-0.00005072,0.,-
0.00000007,0.00017705,0.,0.,0.00002949,-0.00000018,0.00000024,-0.00023059,0.,-
0.00000003,-0.00000030,-0.00000001,0.,-0.00000208,0.00000003,0.,-0.00000143,-
0.00000002,0.,0.00000044,0.,0.,-0.00000260,0.00000002,0.,-
0.00000403,0.,0.,0.00000554,-
0.00000001,0.,0.00000318,0.,0.,0.00000226,0.,0.,0.00000158,0.,-
0.00000001,0.00000456,0.,0.,-0.00000397,0.,0.,-0.00000586,-
0.00000003,0.00000004,0.00000565,0.,-0.00000003,-0.00000704,0.,0.,0.00000426,-
0.00001409,-0.00000548,0.00460698,0.00000088,0.00000272,-
0.00658886,0.00005288,0.00000787,-0.01645093,-0.00004071,-
0.00000212,0.01340270,-0.00071838,0.00075311,0.00000029,0.00075442,-
0.00164954,-0.00000048,-0.00142363,0.00223622,0.00000099,0.00337244,-
0.00234997,-0.00000160,-0.00244356,0.00066612,0.00000091,0.00166498,-
0.00035025,-0.00000049,-0.00187932,0.00027121,0.00000034,-
0.00013070,0.00008873,-0.00000002,-0.00464720,-0.00101685,-0.00000018,-
0.04647438,-0.01226253,-0.00000372,0.00013933,0.00667881,0.00000401,-
0.00182405,0.00019476,0.00000050,-0.00046659,0.00030025,0.00000017,-
0.00013341,0.00025511,0.00000017,-
0.00196015,0.00187066,0.00000132,0.00126267,-0.00020493,-0.00000034,-
0.00025782,-0.00002326,0.,-0.00003209,-0.00012910,-0.00000003,-0.00000750,-
0.00000568,0.00000002,-0.00001150,0.00006987,0.,-0.00027580,-

0.00014700,0.00000004,0.00001449,0.00000037,-0.00000002,0.00006641,-
0.00000151,0.,-0.00001706,0.00000322,0.00000002,0.00007996,0.00008052,-
0.00000002,0.00001502,-0.00001511,0.,-0.00007750,0.00028340,0.00000005,-
0.00006462,0.00010064,0.00000004,-0.00007128,-0.00006168,0.,-
0.00000637,0.00037719,0.00000012,0.00004380,0.00000023,-
0.00000002,0.00013667,0.00006911,-0.00000001,0.00004258,-0.00007893,-
0.00000003,-0.00001505,0.00001629,0.00000002,-0.00007221,-0.00012947,-
0.00000001,0.00000638,0.00002041,0.00000001,-0.00007618,-0.00078795,-
0.00000021,0.00018901,0.00037808,0.00000007,-
0.00012377,0.00027712,0.00000009,-0.24280090,-0.07792757,-0.00003549,-
0.00243493,-0.05305361,-0.00003063,0.00002458,-0.00004863,-
0.00000001,0.00063415,0.00034645,-0.00000006,-0.00037552,-
0.00004953,0.00000008,-0.00016311,-0.00025432,-
0.00000005,0.00003031,0.00005568,0.00000002,0.56052315,0.00002355,0.000097
61,0.00000003,0.00000660,-0.00016144,-0.00000003,-
0.00005816,0.00018547,0.00000004,0.00014935,0.00002398,0.,0.00008998,0.0000
1867,-0.00000004,-0.00002450,-
0.00004673,0.,0.00037876,0.00053198,0.00000021,-
0.00057113,0.00131356,0.00000125,0.00134961,-0.00417464,-
0.00000295,0.01463191,0.01502203,0.00000279,0.00239225,-0.00342419,-
0.00000318,0.00167651,0.00101738,0.00000051,0.00027261,-0.00020087,-
0.00000004,-0.00013590,0.00007802,0.00000006,0.00263246,0.00025271,-
0.00000014,0.00140149,0.00019216,-0.00000014,0.00000830,0.00000030,-
0.00000002,-0.00001103,0.00000417,0.00000002,0.00002726,-0.00000154,0.,-
0.00001367,-0.00000943,0.,0.00001492,0.00000256,0.,0.00000331,0.00001073,0.,-
0.00000344,0.00000163,0.,-0.00000088,0.00000001,0.,-0.00000621,-
0.00001076,0.,0.00000892,0.00000255,0.,-0.00000528,0.00001809,0.,-
0.00002551,0.00004107,0.00000003,-0.00000280,-0.00002257,-0.00000001,-
0.00000704,0.00003155,0.00000001,0.00001437,0.00000942,0.,0.00001700,-
0.00000596,0.,0.00000716,-0.00000876,0.,0.00000022,0.00000052,0.,-0.00001358,-
0.00001258,0.,-0.00000233,0.00001234,0.,-0.00005271,-
0.00003623,0.00000001,0.00004156,0.00002289,-0.00000001,0.00002409,-
0.00000210,-0.00000001,-0.03609556,-0.15585575,-0.00005630,-0.07278699,-
0.06493395,-0.00005280,-0.00000579,0.00000127,0.,0.00001381,-0.00004832,-
0.00000002,-0.00001905,0.00002006,0.00000001,-
0.00000139,0.00003675,0.00000002,0.00000349,-0.00000628,0.,-
0.10266039,0.46939449,0.00000005,-0.00000004,-0.00000623,-0.00000002,0.,-
0.00000623,0.00000008,-0.00000010,0.00003306,-
0.00000019,0.00000020,0.00007029,0.00000022,-0.00000003,0.00000757,-
0.00000011,0.,-0.00000812,0.00000037,0.00000025,0.00008173,-
0.00000043,0.00000121,-0.00077753,0.00000132,-
0.00000292,0.00089912,0.00001338,0.00000482,0.00863838,0.00000165,-
0.00000351,0.00156609,0.00000113,0.00000077,-0.00025542,0.00000018,-
0.00000005,-0.00017219,-0.00000007,0.00000002,-0.00001159,0.00000166,-
0.00000028,0.00042712,0.00000080,0.00000006,0.00013941,0.00000002,0.000000
03,0.00000309,0.00000002,0.00000002,0.00000321,-0.00000002,-
0.00000002,0.00000509,0.,-0.00000001,0.00000323,0.00000002,0.00000002,-
0.00000063,-0.00000005,0.00000002,0.00000150,0.,0.,-0.00000298,0.00000003,-
0.00000002,-0.00000204,0.,-0.00000002,-0.00000740,0.,-0.00000001,-
0.00000362,0.00000002,0.00000004,0.00000175,0.,0.00000003,0.00000878,-
0.00000003,0.,0.00000550,0.00000002,-0.00000002,-0.00000096,-

0.00000001,0.,0.00000216,-0.00000003,0.00000001,0.00000282,0.,0.,-
0.00000327,0.00000003,-0.00000002,-0.00000234,0.00000001,0.,-
0.00001380,0.,0.,-0.00000672,-0.00000002,0.00000002,-0.00000796,0.,-
0.00000001,0.00001005,0.00000002,-0.00000003,0.00000680,-0.00000948,-
0.00005340,-0.06105052,-0.00004267,-0.00005405,0.02874922,0.,0.00000002,-
0.00000581,-0.00000005,-0.00000005,-
0.00000546,0.,0.00000001,0.00000485,0.00000003,0.00000004,0.00000680,0.,0.,-
0.00000495,-0.00010379,0.00027745,0.03812291,0.00012683,-0.00010891,-
0.00000006,-0.00014250,0.00028194,0.00000008,0.00024436,-0.00036228,-
0.00000013,-0.00054921,0.00039596,0.00000026,0.00039675,-0.00009230,-
0.00000015,-0.00026956,0.00005812,0.00000009,0.00033535,-0.00026493,-
0.00000017,0.00023705,0.00019124,0.00000012,0.00065465,-0.00101226,-
0.00000065,-0.00328067,-0.00592141,-0.00000306,-
0.00084440,0.00147415,0.00000094,-0.00043563,-0.00058807,-0.00000032,-
0.00008165,0.00011305,0.00000008,-0.00000504,-0.00001562,-
0.00000001,0.00018153,0.00043522,0.00000022,-0.00022349,-
0.00008565,0.,0.00004454,0.00000562,0.,0.00000645,0.00002217,0.,0.00000181,-
0.00000111,0.,0.00000172,-0.00001034,0.,0.00004925,0.00002671,0.,-0.00000373,-
0.00000103,0.,-0.00001191,0.00000047,0.,0.00000288,-0.00000087,0.,-
0.00001026,-0.00001293,0.,-0.00000355,0.00000277,0.,0.00001211,-0.00005167,-
0.00000001,0.00000478,-0.00000863,0.,0.00001432,0.00000771,0.,-0.00000080,-
0.00006764,-0.00000002,-0.00000512,0.00000219,0.,-0.00002155,-0.00001366,0.,-
0.00000631,0.00001304,0.,0.00000303,-0.00000244,0.,0.00001078,0.00002228,0.,-
0.00000163,-0.00000039,0.,0.00000742,0.00015310,0.00000004,-0.00003138,-
0.00007345,-0.00000001,0.00002852,-0.00005658,-0.00000002,-
0.05005042,0.03413250,0.00002550,0.01068358,0.00227700,0.00000007,-
0.00000583,0.00001055,0.,-0.00011808,-
0.00006092,0.00000002,0.00007076,0.00000833,-
0.00000002,0.00003094,0.00004492,0.,-0.00000661,-0.00001031,0.,-
0.26001576,0.18731389,0.00013589,0.30297569,0.00000788,-0.00003420,0.,-
0.00001813,0.00006515,0.00000003,0.00004036,-0.00008046,-0.00000004,-
0.00007185,0.00008635,0.00000006,0.00005855,-0.00002042,0.,-
0.00001639,0.00002374,0.,0.00006733,-0.00018595,-
0.00000012,0.00017439,0.00014753,0.,-0.00005783,-0.00045045,0.,-0.00349938,-
0.00190885,-0.00000422,-0.00037124,0.00058350,0.00000011,-
0.00023622,0.00003566,0.00000033,-
0.00004757,0.00003002,0.00000005,0.00001031,-0.00001021,-
0.00000002,0.00019862,0.00051466,0.00000038,-0.00005376,0.00002381,-
0.00000010,0.00000400,-0.00000088,0.,0.00000253,0.00000241,0.,-
0.00000390,0.00000003,0.,0.00000309,0.00000049,0.,0.00000119,0.00000164,0.,-
0.00000082,-0.00000160,0.,-0.00000027,-
0.00000039,0.,0.00000052,0.00000014,0.,-0.00000038,0.00000056,0.,-0.00000153,-
0.00000046,0.,0.00000346,-0.00001089,0.,0.00000259,-
0.00000786,0.,0.00000443,0.00000630,0.,0.00000064,-0.00001505,0.,-0.00000304,-
0.00000203,0.,-0.00000606,0.00000019,0.,-0.00000199,0.00000280,0.,0.00000037,-
0.00000040,0.,0.00000361,0.00000773,0.,0.00000058,-
0.00000276,0.,0.00001681,0.00002518,0.,-0.00001542,-0.00001402,0.,-
0.00000462,-0.00000464,0.,-0.01805710,0.02346247,0.00001300,0.00222166,-
0.00533594,0.00000066,0.00000132,0.00000035,0.,-
0.00001191,0.00000580,0.,0.00000915,-0.00000392,0.,0.00000203,-0.00000467,0.,-
0.00000079,0.00000090,0.,0.23781425,-0.25938189,-0.00017046,-

0.21816948,0.24245054,0.00000002,0.00000003,-0.00001877,-
0.00000004,0.00000003,0.00001754,0.00000002,0.,-0.00002001,-
0.00000001,0.00000003,-0.00000461,0.00000001,0.,-0.00002520,-0.00000001,-
0.00000002,0.00003437,0.00000006,-0.00000008,-
0.00000651,0.00000006,0.,0.00015928,-0.00000011,0.00000002,-0.00045748,-
0.00000142,-0.00000416,0.00539442,-0.00000022,0.00000007,0.00045887,-
0.00000017,0.00000023,-0.00042703,-0.00000003,0.00000004,-0.00005080,0.,-
0.00000001,0.00002932,0.00000010,0.00000041,-0.00023247,0.,-
0.00000009,0.00017624,0.00000001,-0.00000002,-0.00000027,-0.00000001,0.,-
0.00000207,0.00000002,0.00000001,-0.00000403,0.,0.,-
0.00000256,0.,0.,0.00000041,0.00000002,0.,-0.00000143,0.,0.,0.00000226,-
0.00000002,0.,0.00000158,-0.00000001,0.,0.00000552,0.,0.,0.00000316,0.,-
0.00000003,-0.00000111,0.,0.,-0.00000539,0.00000002,0.,-0.00000346,-
0.00000001,0.,0.00000058,0.,0.,-0.00000165,0.00000002,0.,-
0.00000207,0.,0.,0.00000233,-
0.00000001,0.,0.00000175,0.,0.,0.00000811,0.,0.,0.00000434,0.,0.,0.00000568,0.,0.
,-0.00000708,0.,0.,-0.00000494,-
0.00000766,0.00000940,0.00460345,0.00000009,0.00000055,-
0.00657486,0.,0.,0.00000425,0.,0.,0.00000460,0.,0.,-0.00000402,0.,0.,-
0.00000583,0.,0.,0.00000427,0.00016793,-0.00016675,-0.01644473,-
0.00015866,0.00016036,0.01338606\\-0.00004672,0.00004302,-
0.00000081,0.00008463,-0.00002983,-0.00000058,-0.00001776,0.00000692,-
0.00000090,-0.00001911,-0.00001622,0.00000456,-0.00004706,0.00001581,-
0.00000168,0.00000010,-0.00001073,0.00000375,0.00005216,0.00001776,-
0.00000260,0.00007686,-0.00003255,0.,0.00001262,0.00001439,0.00000048,-
0.00006389,-0.00002589,0.00000043,-0.00006741,0.00003664,0.00000029,-
0.00001838,-0.00001027,-0.00000091,-0.00002190,0.00001204,0.00000054,-
0.00003098,-0.00000558,0.00000018,0.00001719,-
0.00001763,0.00000017,0.00002672,0.00000670,-0.00000014,0.00000039,-
0.00002213,-0.00000167,0.00005539,-0.00004358,0.00000023,0.00001476,-
0.00000225,-0.00000061,-
0.00006018,0.00004409,0.00000082,0.00001565,0.00002186,-0.00000060,-
0.00000924,0.00001076,-0.00000059,0.00001851,0.00000030,-0.00000023,-
0.00000273,-0.00000195,0.00000047,-0.00001248,-0.00001121,0.00000025,-
0.00001886,-0.00000060,-0.00000026,-0.00001040,0.00005847,0.00000026,-
0.00000879,-0.00000477,-0.00000014,0.00005071,0.00000827,-
0.00000016,0.00003468,-0.00003969,-0.00000092,0.00001917,0.00001209,-
0.00000011,-0.00007143,0.00000342,0.00000040,0.00004538,-0.00003481,-
0.00000053,-0.00001756,-0.00000938,-
0.00000011,0.00001399,0.00001188,0.00000014,-0.00000978,-0.00000392,-
0.00000025,-0.00000333,-0.00001219,0.00000363,-0.00002659,0.00001497,-
0.00000160,0.00001000,0.00002067,-0.00000060,0.00007494,-0.00000592,-
0.00000263,-0.00000588,-0.00000225,0.00000091,0.00000612,-0.00001053,-
0.00000030,-0.00003242,-0.00002074,0.00000122,-0.00000529,-0.00000465,-
0.00000047,0.00003165,-0.00000130,-0.00000029,-0.00000233,-0.00000542,-
0.00000006,-0.00004189,0.00002520,0.00000114,0.00001079,0.00000074,-
0.00000013\\\@ 3,0.00004634,0.00002374,0.00001733,-0.00000283,-0.00003482,-
0.00003998,0.00002369,0.00001148,0.00003239,0.00001212,-
0.00000320,0.00001292,-0.00002157,0.00001475,-0.00002025,-0.00002222,-
0.00001067,0.00003224,0.00000121,-0.00000962,-0.00001135\\\@

TATB (1,3,5-triazine-2,4,6,-tribenzoic acid)

The BTB molecule was optimized using Gaussian® 09 [7] with B3LYP//6-31G*.

Energy: -965912.4624218

N	-0.31172	1.33316	0.00010
C	0.95712	0.89757	0.00012
N	1.31008	-0.39655	0.00015
C	0.29846	-1.27761	0.00013
N	-0.99878	-0.93623	0.00011
C	-1.25600	0.38039	0.00011
C	0.63612	-2.72290	0.00008
C	1.97738	-3.13740	0.00040
C	2.29624	-4.49078	0.00035
C	1.27525	-5.45157	-0.00005
C	-0.06509	-5.04180	-0.00038
C	-0.38269	-3.68972	-0.00030
H	-1.41640	-3.36476	-0.00054
H	2.75925	-2.38714	0.00070
H	3.33293	-4.80800	0.00062
H	-0.84159	-5.79938	-0.00068
C	-2.67650	0.81061	0.00006
C	-3.00440	2.17634	-0.00005
C	-3.70617	-0.14360	0.00014
C	-5.03766	0.25700	0.00011
C	-5.35924	1.62159	-0.00001
C	-4.33413	2.57740	-0.00009
H	-2.20618	2.90914	-0.00012
H	-4.60177	3.62872	-0.00017
H	-3.44761	-1.19591	0.00024
H	-5.83068	-0.48225	0.00017
C	2.04007	1.91251	0.00008
C	3.38673	1.51348	0.00001
C	4.39900	2.46441	-0.00006
C	4.08397	3.83013	-0.00004
C	2.74146	4.23409	0.00003
C	1.72867	3.28138	0.00009
H	0.68806	3.58361	0.00013
H	2.49780	5.29051	0.00004
H	3.62219	0.45578	-0.00001
H	5.44324	2.17042	-0.00013
C	5.20817	4.80548	-0.00013
O	6.38544	4.50696	-0.00027

O	4.79028	6.09682	-0.00006
C	1.55839	-6.91272	-0.00014
O	0.71162	-7.78338	-0.00051
H	5.60203	6.63716	-0.00015
C	-6.76624	2.10688	-0.00004
O	-7.09701	3.27549	-0.00015
O	-7.67503	1.09874	0.00004
H	-8.54915	1.53098	0.00000
O	2.88583	-7.19581	0.00027
H	2.94847	-8.16894	0.00017

1\1\GINC-ACID10\Freq\RB3LYP\6-31G(d)\C24H15N3O6\WINKLER\13-Sep-2016\0\\#N Geom=AllCheck Guess=TCheck SCRF=Check GenChk RB3LYP/6-31G(d) Freq\\Title\\0,1\N,-1.1401903877,0.7577732869,0.0144617401\C,0.0826720638,1.3094158965,0.0143672534\N,1.2261866429,0.608207938,0.0139165625\C,1.092513972,-0.7266172174,0.0135180922\N,-0.0865112887,-1.3663533129,0.0135674073\C,-1.1756840739,-0.5831834696,0.014062193\C,2.3284296408,-1.5484450986,0.012919009\C,3.5896133783,-0.9318426151,0.0131400316\C,4.7488770009,-1.6995803864,0.0125841997\C,4.662984125,-3.0989243532,0.0117735747\C,3.4057109758,-3.7183505391,0.0115475627\C,2.2482616759,-2.9506941969,0.0121261525\H,1.2724924368,-3.4219046119,0.0119727392\H,3.6454627214,0.150323436,0.0137592061\H,5.7215124362,-1.2206940972,0.012772477\H,3.3587886788,-4.8021754951,0.010916616\C,-2.5053637508,-1.2426068299,0.0141250296\C,-3.6796776068,-0.4720637117,0.0145208426\C,-2.6020892048,-2.6431064378,0.0137975795\C,-3.8466351731,-3.2631476504,0.0138667694\C,-5.0155556488,-2.4891041075,0.0142592757\C,-4.9232500296,-1.0905654,0.0145853889\H,-3.5999529373,0.6085848988,0.0147695643\H,-5.8383092278,-0.5078432348,0.0148957946\H,-1.6929648434,-3.2327680685,0.0135025055\H,-3.9181455502,-4.344924074,0.0136126733\C,0.1766135797,2.7906499402,0.0147616084\C,1.4311330907,3.4222270386,0.0145904612\C,1.5174236803,4.8084132122,0.014930077\C,0.3525028282,5.5877730851,0.015457089\C,-0.9023745657,4.9626204397,0.0156335346\C,-0.9878215806,3.574781762,0.0152833484\H,-1.9530418665,3.0822856712,0.015399103\H,-1.803450182,5.5654879992,0.0160356863\H,2.3271184832,2.8128174366,0.0141816237\H,2.4796510128,5.3094018123,0.0147898087\C,0.5036221955,7.0684049549,0.0157950049\O,1.5658812784,7.6572063633,0.0155856007\O,-0.6853021335,7.7231128823,0.0163394529\C,5.8700371511,-3.9696517089,0.0111345348\O,5.849362635,-5.1839991961,0.0103906983\H,-0.463908874,8.6727906505,0.0164876452\C,-6.3731260866,-3.0991589712,0.0143503532\O,-7.4145149093,-2.4742097096,0.0146787463\O,-6.3448330076,-4.4561548059,0.0140134191\H,-7.2777858606,-4.7398863844,0.0141027738\O,7.0311450898,-3.2667551767,0.0114906088\H,7.7432820157,-

3.9329178437,0.0110185815\\Version=EM64L-G09RevD.01\State=1-A\HF=-
1539.2795012\RMSD=3.353e-09\RMSF=2.425e-
05\ZeroPoint=0.353128\Thermal=0.3798692\Dipole=0.000178,0.0001152,0.0002695
\DipoleDeriv=-1.210724,-0.6152929,0.0000576,-0.6220184,-1.7277905,-
0.0003074,0.0000229,-0.0003112,-0.2255822,1.0180206,0.0907227,-
0.0001646,0.1001259,2.5006834,0.0006468,-0.0001254,0.0006066,0.2069222,-
1.062974,0.5364137,0.0003587,0.5299413,-1.8762089,-0.0006257,0.0003583,-
0.0006043,-0.2255565,2.047764,-0.6945062,-0.0006813,-
0.6850411,1.4710996,0.0005511,-0.0007075,0.0005846,0.2069031,-
2.1347291,0.088729,0.0004829,0.0822808,-0.8041025,-0.0001821,0.0005029,-
0.0002203,-0.2256463,2.2120155,0.589633,-
0.0002788,0.5993992,1.3058623,0.0001989,-0.0002735,0.0002351,0.2069536,-
0.4290899,0.2965724,0.0002084,0.3051353,-0.190102,-0.0001275,0.000227,-
0.0001288,-0.0056582,-0.1222136,-0.1219315,-
0.000069,0.1283589,0.0349678,0.000068,0.0000272,0.0001133,-
0.0909517,0.1598189,0.1068629,0.0000459,-0.0449429,-0.0837249,0.0000097,-
0.0000449,-0.0000086,-0.0952467,-0.5986359,0.3961427,0.0002833,0.3683137,-
0.2265807,-0.0001771,0.0002785,-0.0001959,0.027497,-0.0851579,-0.130016,-
0.0000842,0.0381956,0.1260025,0.0001198,0.0000207,0.0001451,-
0.08905,0.0251101,0.180297,0.0000913,-0.095603,-0.0834763,0.0000092,-
0.0000234,-0.0000565,-0.0917801,0.0321306,-0.0680369,-0.000034,-
0.0909183,0.0993617,0.0000042,-0.0000595,0.0000185,0.1071221,0.145912,-
0.0444874,-0.0000218,-0.0200635,-0.0146226,-0.0000714,0.0000075,-
0.0000783,0.1072515,-0.0118374,-0.0351772,-0.0000123,-0.05966,0.0685454,-
0.0000223,-0.0000182,-0.0000261,0.1149721,0.0970967,-0.0041579,-0.0000003,-
0.0066672,-0.0134523,-0.0000712,-0.0000066,-0.0000719,0.1137622,-0.5104198,-
0.2581996,0.0000303,-0.2499225,-0.1089535,0.0000199,0.0000279,0.0000205,-
0.0056369,-0.0925183,0.1639194,0.0000392,-0.1119051,0.0348409,0.0000517,-
0.000048,-0.0000095,-0.0917511,-0.0072548,-0.1948991,-0.0000538,0.0555051,-
0.0800769,-0.0000092,0.0000164,0.0000397,-0.0910096,-
0.0491455,0.1660016,0.0000282,0.0141455,0.1259005,0.0000531,-
0.0000055,0.0000319,-0.0952089,-0.6508306,-0.3382924,0.0000462,-0.3661068,-
0.1745914,0.0000162,0.0000293,0.0000061,0.0275291,0.1127207,-0.1527594,-
0.0000724,0.015485,-0.0721267,0.0000062,-0.0000328,0.0000366,-
0.0891309,0.151298,0.0219987,-0.0000043,-0.0010644,-0.0201463,-0.0000302,-
0.000001,-
0.0000329,0.1071566,0.0188141,0.0517559,0.0000303,0.0493616,0.0648186,-
0.0000209,0.0000325,-
0.0000208,0.1137735,0.0532242,0.0734116,0.0000255,0.0980289,0.0779427,-
0.0000283,0.0000213,-0.0000261,0.1072531,0.0894919,0.0011523,0.0000061,-
0.0235541,-0.0329952,-0.0000326,-0.0000028,-0.0000396,0.1149961,0.0108377,-
0.0515807,-0.0000204,-0.0428106,-0.629742,-0.0001688,-0.0000167,-0.0001473,-
0.0056357,-0.0196233,0.0698423,-0.0000019,-0.2061233,-0.0386422,0.0000738,-
0.0000741,0.0000063,-0.0917948,0.0332483,0.0304038,-
0.000022,0.1984513,0.0068945,-0.0000349,0.0000153,0.0000148,-
0.0890611,0.0116123,-0.0165581,0.0000002,-0.0441625,-0.8364513,-0.0002282,-
0.0000043,-0.000223,0.0275511,0.0040178,-0.044901,-0.0000416,-
0.1967427,0.0722823,0.0001005,-0.0000809,0.0000469,-0.0952317,-0.0015851,-
0.0584971,-0.0000433,0.1915343,-0.0862048,-0.000055,0.0000321,0.0000029,-
0.0910056,-0.0024941,-0.0656787,0.0000131,-
0.0410995,0.133812,0.0000226,0.0000151,0.000032,0.1073014,0.0073656,0.07077

12,0.0000492,0.0463324,0.0491937,-0.0000323,0.0000403,-
0.0000341,0.1149561,0.0136625,0.0802656,0.000048,0.0574902,0.1177264,-
0.0000102,0.0000398,-0.0000232,0.1071045,0.0096054,-0.0438552,0.0000183,-
0.0463208,0.0742836,0.0000034,0.000017,-0.0000018,0.1137501,1.4105558,-
0.2218204,-0.0004236,0.0130127,2.5942913,0.0006288,-
0.0003555,0.0006666,0.3089096,-0.8608325,-0.2881756,0.0000832,-0.3184617,-
1.2868034,-0.0001541,0.0000887,-0.0001453,-0.3589523,-
0.8709257,0.67363,0.0003496,0.406261,-1.0838923,-0.0003359,0.0002639,-
0.0004018,-0.373668,2.3890847,-0.5778251,-0.0004804,-
0.34244,1.6159015,0.0008467,-0.0003653,0.0008601,0.3090049,-
0.9177588,0.3514626,0.0002487,0.3210609,-1.2298294,-0.0005693,0.0002365,-
0.0005649,-0.3589607,0.2672122,-0.172352,-0.0000202,-
0.0587129,0.3208331,0.0000175,0.0000078,0.0000546,0.3444294,2.2074495,0.446
8997,-0.0002339,0.6822151,1.7970065,0.0002279,-
0.0001643,0.0002803,0.3089555,-1.4429043,-0.0176935,0.00019,-0.0480756,-
0.7045125,-0.0000719,0.0001738,-0.0000782,-0.3589447,-0.5625888,-0.2278773,-
0.0000187,-0.495374,-1.3919023,-0.0001499,-0.0000858,-0.0002054,-
0.3736968,0.2071159,0.0240365,0.0000287,0.1375968,0.3807331,-
0.0000168,0.0000565,0.0000023,0.3443992,-1.4982691,-0.0442795,0.0000348,-
0.3119162,-0.4566652,-0.0000354,-0.0001199,-0.0000493,-0.3736976,0.4073195,-
0.0220735,-0.0000149,0.0914759,0.1806054,-0.0001079,0.0000564,-
0.0001001,0.3443955\Polar=446.9067805,0.0180639,446.892825,-
0.0762888,0.1165874,93.4008988\PG=C01
[X(C24H15N3O6)]\NImag=0\\0.50472257,0.10594955,0.59404347,-
0.00005752,0.00013413,0.08014146,-0.28447892,-
0.05522986,0.00002640,0.66977273,-0.07956120,-0.15801472,-
0.00000716,0.00134191,0.69078305,0.00001959,-0.00001322,-0.07744945,-
0.00009882,0.00014218,0.20394599,-0.08822505,-0.02232739,0.00002039,-
0.26608376,0.09330199,0.00006681,0.48034977,0.04298793,0.07421023,0.000000
12,0.06896013,-0.17699441,-0.00004189,-
0.09166652,0.61893349,0.00004086,0.00001689,0.03929774,0.00005864,-
0.00004830,-0.07744917,-0.00011064,0.00018725,0.08015024,-0.02028028,-
0.04909587,-0.00001258,0.04248898,0.04538520,0.00000409,-0.13110533,-
0.00885621,0.00000937,0.68429911,-0.04911448,-0.06149620,-
0.00000322,0.10133129,-0.06867569,-0.00004539,-0.03317610,-0.31127145,-
0.00006728,-0.01006770,0.67586867,-0.00001453,-0.00000517,-
0.01282255,0.00002419,-0.00003291,0.00467300,0.00000019,-0.00007455,-
0.07736810,-
0.00010871,0.00016771,0.20390491,0.04244888,0.09793553,0.00002952,-
0.09366841,0.00654633,0.00001997,0.02464323,-0.10817000,-0.00003158,-
0.26976714,-0.06674502,0.00002126,0.66447971,0.03258743,-0.05658676,-
0.00003725,0.00657799,0.01182836,0.00000612,-0.04287126,-0.03869911,-
0.00001616,-0.09120283,-0.17327571,-0.00001318,-
0.01420185,0.43526463,0.00001039,-
0.00005024,0.03928892,0.00001890,0.00000626,-0.01277543,-0.00001050,-
0.00000095,0.03926689,0.00001232,-0.00001796,-0.07740056,-
0.00013589,0.00012044,0.08009833,-0.12879909,-
0.01436921,0.00000596,0.02249222,-0.05683987,-0.00002148,-
0.00868660,0.04238709,0.00001344,-0.10407406,-0.01645948,0.00001949,-
0.24840967,0.10053918,0.00007086,0.68660416,0.01005159,-0.31382538,-
0.00007383,-0.11279424,-0.04854265,0.00000861,0.04241640,-0.07311857,-

0.00002864,0.03950513,0.07825541,0.00001618,0.07608466,-0.19442149,-
0.00005705,0.00885214,0.67336294,0.00001339,-0.00006897,-0.07735138,-
0.00003859,-0.00000450,0.00464462,0.00001198,-0.00002794,-
0.01279760,0.00003578,0.00002574,0.00457868,0.00006323,-0.00005988,-
0.07743266,-0.00010110,0.00014291,0.20385939,-
0.00231208,0.00377708,0.00000038,0.00447125,-
0.00598299,0.00000032,0.00443171,0.01710057,0.00000546,-
0.17479266,0.04939993,0.00003885,-
0.05398864,0.01105341,0.00001824,0.00288934,-0.00818489,-
0.00000138,0.65980684,0.00377814,0.00087873,0.00000140,-0.00751646,-
0.00306656,-0.00000296,0.03559490,-0.04211203,-0.00002396,0.04946328,-
0.13343469,-0.00003868,-0.00754467,0.01630379,0.00000562,-0.00664485,-
0.00150608,-0.00000183,0.03100666,0.68435844,0.00000046,0.00000022,-
0.00487077,-0.00000096,-0.00000286,0.00906214,0.00001223,-
0.00001871,0.00425049,0.00004048,-0.00003972,-
0.07237937,0.00001145,0.00000202,0.00426433,-0.00000187,-
0.00000097,0.00907243,-0.00004288,0.00028754,0.15612996,-0.00044331,-
0.00180402,-0.00000078,0.00029476,-0.00151887,-0.00000012,-0.00522793,-
0.00280152,-0.00000114,-
0.03608624,0.00025974,0.00000363,0.00417674,0.00207003,-0.00000039,-
0.00326640,0.00287397,0.00000249,-0.27072475,-0.05275255,-
0.00000879,0.67374464,0.00064955,-0.00058027,0.00000009,-
0.00092359,0.00162242,0.00000040,0.00239316,-0.00420794,-
0.00000266,0.01268942,0.01949976,0.00000793,0.00665112,-0.00304397,-
0.00000391,0.00018451,0.00077303,-0.00000039,-0.10775867,-0.17034806,-
0.00004695,-0.01473369,0.75501598,-0.00000004,-0.00000019,-0.00005650,-
0.00000002,0.00000061,-0.00025560,0.00000018,-
0.00000162,0.00096430,0.00001262,0.00000882,0.00227790,0.00000373,-
0.00000297,0.00218861,0.00000090,-0.00000008,0.00037680,-0.00004220,-
0.00005296,-0.06663741,-
0.00006210,0.00035777,0.13614129,0.00086688,0.00101972,0.00000036,-
0.00117500,0.00084744,0.00000006,0.00200367,-0.00096880,-0.00000013,-
0.00090230,-0.00593641,-0.00000275,-
0.00321558,0.00118126,0.00000125,0.00041174,-0.00162424,-0.00000083,-
0.05649595,-0.01357326,-0.00000088,-
0.28479659,0.13457501,0.00009892,0.74955756,-0.00008225,-0.00116931,-
0.00000072,0.00087049,0.00025864,0.00000010,0.00013965,-
0.00028789,0.00000040,-0.00584927,-0.00001959,-
0.00000315,0.00208748,0.00054843,-0.00000033,-
0.00175609,0.00152237,0.00000173,0.03212390,0.05858816,0.00002551,0.067463
05,-0.19825672,-0.00008485,0.02499406,0.67766176,0.00000007,-0.00000055,-
0.00004380,0.00000016,0.00000010,0.00023127,0.00000027,-0.00000018,-
0.00058427,-0.00000245,-
0.00000314,0.00660698,0.00000092,0.00000033,0.00031106,-
0.00000120,0.00000094,-
0.00077374,0.00002593,0.00003234,0.00672893,0.00005989,-0.00009310,-
0.06043954,-0.00004404,0.00031481,0.13454634,-
0.00039683,0.00133487,0.00000071,-0.00047625,-0.00158277,-0.00000041,-
0.00067182,0.00075620,0.00000020,0.00185633,-0.00257998,-0.00000196,-
0.00333764,0.00124615,0.00000171,0.00243111,-0.00073055,-0.00000122,-
0.01898149,-0.02752946,-0.00001396,0.03512804,0.02470543,0.00001131,-

0.12860277,0.00805387,0.00001097,0.65967448,0.00126886,0.00074105,0.000000
28,-0.00186806,0.00137883,0.00000032,0.00228070,-0.00234534,-0.00000087,-
0.00229113,-0.00051604,0.00000008,-0.00051600,0.00016877,0.00000011,-
0.00025819,-0.00144783,-0.00000058,-0.02790483,-0.04159458,-
0.00001561,0.07096998,-0.03355737,-0.00003222,-0.04969830,-0.31951878,-
0.00014599,0.03305792,0.69197501,0.00000079,0.00000032,0.00004667,-
0.00000103,0.00000103,0.00001897,0.00000149,-0.00000148,-0.00013177,-
0.00000145,0.00000015,-0.00041659,-0.00000008,0.00000009,-0.00013446,-
0.00000034,-0.00000074,0.00002079,-0.00001541,-0.00001706,-
0.00762496,0.00003822,-0.00002625,0.00756297,-0.00002243,-0.00015162,-
0.06253867,-0.00003435,0.00031945,0.14358170,-0.00120272,-0.00095131,-
0.00000060,0.00256940,-0.00085041,-0.00000025,-
0.00119337,0.00294558,0.00000125,0.00406472,-0.00371969,-
0.00000139,0.00036572,-0.00055437,-0.00000050,-
0.00057042,0.00113129,0.00000108,0.03467783,0.07044273,0.00003618,-
0.06561335,0.00543967,0.00000937,0.02711818,-0.07726950,-0.00004646,-
0.27549719,-0.05430138,-
0.00000833,0.67955284,0.00016361,0.00087006,0.00000005,-0.00072252,-
0.00060484,0.00000078,0.00207067,-0.00153364,-0.00000125,-0.00391750,-
0.00499281,-0.00000612,-0.00159085,0.00134627,0.00000177,0.00105181,-
0.00033099,-0.00000040,0.02548414,-0.03246095,-0.00002598,0.00427859,-
0.00117979,0.00000244,-0.03358856,-0.01958232,-0.00001281,-0.11223718,-
0.17049537,-0.00005026,-0.01535241,0.75575876,0.00000041,0.00000094,-
0.00004117,-0.00000131,-0.00000014,-0.00077420,0.00000197,-
0.00000197,0.00030073,-0.00000234,-0.00000586,0.00663696,-
0.00000160,0.00000147,-0.00058963,0.00000104,-
0.00000092,0.00022760,0.00001250,-
0.00002955,0.00675946,0.00000886,0.00000246,-0.00646243,-0.00002167,-
0.00000807,0.00847085,-0.00004221,-0.00005607,-0.06477429,-
0.00006948,0.00036423,0.13699355,-0.00014638,0.00075485,0.00000075,-
0.00092013,0.00117675,-0.00000024,-
0.00506729,0.00275303,0.00000274,0.00656883,0.03058842,0.00001657,-
0.00420035,0.00279242,0.00000249,0.00227919,-0.00007596,-0.00000106,-
0.12817041,-0.04792567,-0.00001826,0.02676805,-0.03374203,-0.00002139,-
0.01459393,0.02714234,0.00001662,-0.05646499,-0.01352152,-0.00000085,-
0.28785022,0.13596992,0.00010509,0.75695781,-0.00169254,-
0.00086515,0.00000005,0.00386133,-0.00158809,-0.00000094,-
0.00185719,0.00623448,0.00000082,0.01819550,-0.02309030,-0.00001636,-
0.00241702,-0.00519564,-0.00000333,-0.00064241,-
0.00036162,0.00000025,0.00700186,-0.31160735,-0.00014141,-0.07465839,-
0.01903420,-0.00000798,0.02656517,-0.05208735,-
0.00002915,0.03189280,0.05842217,0.00002657,0.06990704,-0.20024857,-
0.00008997,0.01738360,0.67334860,-0.00000084,-0.00000100,-
0.00005392,0.00000214,-0.00000087,0.00037775,-
0.00000009,0.00000223,0.00221123,0.00000793,-
0.00001705,0.00229479,0.00000082,-0.00000476,0.00094220,-
0.00000142,0.00000027,-0.00025722,0.00001371,-0.00013631,-0.06655188,-
0.00004526,-0.00001247,0.00875702,0.00001605,-0.00002930,-
0.00653974,0.00002509,0.00003050,0.00720945,0.00006771,-0.00009652,-
0.06084295,-0.00006324,0.00030745,0.13682679,-0.00002101,-0.00008411,-
0.00000004,-0.00011531,0.00009083,0.00000012,0.00014213,-0.00041869,-

0.00000016,-0.00135443,-0.00031366,-0.00000028,-
0.00156133,0.00331510,0.00000208,-0.00002065,0.00015111,-
0.00000004,0.00245127,-0.00187076,-
0.00000085,0.00126893,0.00045896,0.00000096,0.00028938,0.00075930,0.000000
34,-0.00193464,0.00367884,0.00000346,-0.01771184,-0.01212501,-0.00000446,-
0.29134998,-0.11585555,-0.00003756,0.30983666,-0.00001817,0.00086287,-
0.00000012,-0.00038157,-0.00025370,-
0.00000011,0.00037663,0.00017423,0.00000013,-
0.00223228,0.00045006,0.00000251,-0.00026993,-
0.00031500,0.00000046,0.00140840,0.00022437,0.00000033,-0.02986815,-
0.00925507,-0.00000385,0.00153267,-0.00591212,-0.00000689,0.00056359,-
0.00071752,-0.00000031,0.00306155,-0.00180011,-
0.00000584,0.01789018,0.01225926,0.00000295,-0.11372026,-0.11564456,-
0.00003008,0.12287981,0.11862577,-
0.00000012,0.00000029,0.00031197,0.00000019,-0.00000029,0.00024265,-
0.00000002,0.00000086,-0.00016191,-0.00000083,0.00000176,-
0.00344027,0.00000040,-0.00000081,-0.00004794,0.00000058,0.00000071,-
0.00044879,-0.00001801,-0.00000626,0.00351880,0.00000147,-
0.00000685,0.00578099,0.00000015,-0.00000035,-0.00032218,0.00000282,-
0.00000596,0.00771258,0.00001266,0.00000640,0.00349047,-0.00003562,-
0.00003048,-
0.03885329,0.00003724,0.00003743,0.02680383,0.00076764,0.00030953,-
0.00000019,-0.00036989,0.00126379,0.00000064,-0.00152196,-
0.00032896,0.00000049,0.00109926,-0.00124186,-
0.00000113,0.00019086,0.00039881,0.00000012,-0.00013027,-0.00038580,-
0.00000008,0.00368763,-0.02931343,-0.00001703,-0.05994673,-0.01641227,-
0.00000637,0.00556715,0.02774507,0.00001574,-
0.00428783,0.00208586,0.00000231,-0.00104124,-0.00000141,0.00000005,-
0.00556450,-0.00133844,0.00000035,0.00012246,-0.00167492,-
0.00000093,0.05968985,0.00024452,0.00007478,-
0.00000029,0.00000229,0.00056545,0.00000047,0.00327645,-0.00024581,-
0.00000017,0.00077604,-0.00196206,0.00000102,-
0.00040024,0.00012932,0.00000034,0.00008416,-0.00023859,-0.00000024,-
0.00133019,-0.01071911,-0.00000785,-0.01853823,-0.34709033,-0.00017688,-
0.00237427,-0.01106362,-0.00000807,0.00274559,0.00049095,-
0.00000430,0.00017913,0.00061317,0.00000052,-0.00239660,0.00085892,-
0.00000243,0.00017691,-0.00050703,-
0.00000071,0.01766345,0.36895932,0.00000013,-
0.00000022,0.00031336,0.00000036,0.00000037,-
0.00045177,0.00000141,0.00000014,-0.00006751,-0.00000036,0.00000142,-
0.00351825,0.00000035,0.00000019,-0.00016131,-
0.00000007,0.00000005,0.00024719,0.00000021,-0.00000558,0.00351073,-
0.00000850,-0.00017663,-0.03866347,-0.00000156,-
0.00001142,0.00341260,0.00000278,-
0.00000430,0.00767610,0.00000006,0.00000050,-0.00037121,-0.00000030,-
0.00000270,0.00572773,0.00000021,-
0.00000058,0.00068397,0.00000609,0.00019539,0.02682112,-
0.00004059,0.00012081,-0.00000002,0.00004458,-0.00016756,0.00000006,-
0.00005356,0.00026974,0.00000005,-0.00014842,-0.00131751,-0.00000055,-
0.00061946,0.00028124,0.00000013,0.00008491,-0.00014744,0.00000009,-
0.00205570,0.00383505,0.00000299,-0.01811316,-0.01256472,-0.00000535,-

0.29033594,-0.11620789,-0.00004411,0.00248169,-0.00027593,-
0.00000011,0.00145268,0.00051597,0.00000062,0.00027347,0.00076323,0.000000
40,-0.00006206,-0.00004606,-0.00000012,0.00106150,0.00011470,-
0.00000041,0.30787928,-0.00004334,-0.00005767,-
0.00000001,0.00003997,0.00002093,0.00000005,-0.00008232,-0.00001053,-
0.00000011,0.00054057,0.00078458,0.00000030,0.00035211,-0.00017855,-
0.00000001,0.00005535,0.00009925,0.00000006,0.00311903,-0.00188852,-
0.00000592,0.01787988,0.01234285,0.00000364,-0.11386472,-0.11721880,-
0.00003583,-0.02956029,-0.00940121,-0.00000428,0.00147845,-0.00595504,-
0.00000709,0.00058836,-0.00064833,-0.00000025,-
0.00003431,0.00009326,0.00000074,-
0.00044750,0.00086918,0.00000304,0.12240650,0.12042887,-0.00000005,-
0.00000003,-0.00002958,0.,-0.00000002,0.00002133,-
0.00000012,0.00000005,0.00006806,0.00000040,0.00000043,0.00028605,0.000000
17,-0.00000003,-0.00030379,-0.00000003,0.00000012,-0.00000282,0.00000282,-
0.00000592,0.00771276,0.00001249,0.00000655,0.00334077,-0.00004298,-
0.00003588,-0.03788074,-0.00001709,-0.00000696,0.00276096,0.00000131,-
0.00000691,0.00583695,0.00000042,-0.00000028,-0.00028281,-
0.00000018,0.00000075,-0.00118378,-0.00000072,0.00000288,-
0.00426835,0.00004512,0.00004365,0.02627578,-0.00009321,-
0.00006149,0.00000002,0.00014156,0.00006267,-0.00000005,-
0.00047723,0.00042032,0.00000022,0.00094369,0.00095509,0.00000047,-
0.00010744,-0.00009642,-0.00000006,0.00008550,0.00005148,0.,-
0.00439922,0.00213702,0.00000270,-0.00102827,0.00002908,0.00000011,-
0.00557758,-0.00130895,0.00000043,0.00350070,-0.02927733,-0.00001714,-
0.06041513,-0.01356919,-
0.00000574,0.00578676,0.02757785,0.00001594,0.00103542,-0.00049086,-
0.00000090,0.00008632,0.00004086,-0.00000012,0.00014408,-0.00167154,-
0.00000091,0.05931846,0.00008207,-0.00000577,0.00000004,-
0.00010726,0.00002873,-0.00000005,0.00029061,-0.00026297,0.00000007,-
0.00076094,-0.00028306,-0.00000028,0.00020149,0.00002623,-0.00000002,-
0.00011511,-0.00000655,-0.00000008,0.00264902,0.00052729,-
0.00000456,0.00021429,0.00061326,0.00000053,-0.00232885,0.00084790,-
0.00000254,-0.00105329,-0.01083912,-0.00000854,-0.01577527,-0.34577952,-
0.00017786,-0.00222842,-0.01072294,-
0.00000804,0.00007708,0.00086946,0.00000305,0.00002840,-
0.00006982,0.00000060,0.00013104,-0.00043899,-
0.00000070,0.01571447,0.36652661,0.00000005,0.00000001,-0.00003263,-
0.00000001,0.,0.00000290,0.00000013,-0.00000011,-0.00030701,-0.00000045,-
0.00000038,0.00024067,0.00000025,-0.00000005,0.00007514,-0.00000010,-
0.00000004,0.00002574,0.00000277,-
0.00000462,0.00777194,0.00000030,0.00000063,-0.00034463,-0.00000007,-
0.00000259,0.00560235,-0.00000100,-0.00000527,0.00381650,-0.00000670,-
0.00017823,-0.03909957,-0.00000169,-0.00001105,0.00335993,-
0.00000068,0.00000307,-0.00432984,-0.00000008,0.00000058,-
0.00109609,0.00000014,-
0.00000047,0.00072727,0.00000593,0.00019697,0.02747184,-0.00270861,-
0.02196040,-0.00000553,0.00602571,0.00483489,0.00000235,-0.00318689,-
0.00326788,-0.00000156,0.00467534,0.00741158,0.00000323,-0.05325384,-
0.00223428,0.00001170,-0.18635752,-0.04269921,0.00000921,-0.00081029,-
0.00052594,-0.00000012,0.00009829,0.00023890,0.00000008,-

0.00009215,0.00004062,0.,-0.00027657,0.00008357,0.00000004,0.00007199,-
0.00004387,-0.00000002,-0.00007062,0.00003514,0.00000013,0.00006245,-
0.00005377,-0.00000009,0.00009495,-0.00002772,0.00000002,-
0.00003890,0.00001352,0.00000004,-0.00001024,0.00001622,0.,0.65102192,-
0.04057912,-0.03490320,-0.00000381,0.00636922,-0.00463293,-0.00000555,-
0.00326899,0.00174763,0.00000269,0.00589723,-0.00325681,-
0.00000569,0.01629163,0.01565319,0.00000040,-0.04266977,-0.12152211,-
0.00000501,0.00077406,0.00111798,0.00000027,0.00026044,-0.00036363,-
0.00000018,0.00009503,0.00014361,0.00000011,-0.00003384,0.00001055,0.,-
0.00000354,0.00000545,0.,-0.00009674,-
0.00004957,0.,0.00005088,0.00000440,0.00000002,0.00006752,0.00001647,0.0000
0001,0.00003135,-0.00002483,-0.00000004,0.00000248,-0.00000272,-0.00000001,-
0.02611821,0.69238871,-0.00001104,-0.00000719,0.00425342,0.00000293,-
0.00000516,0.00907073,-0.00000147,0.00000287,-0.00486917,0.00000282,-
0.00000558,0.00905987,0.00001775,0.00000399,0.00426002,0.00000870,-
0.00000569,-0.07233799,0.00000030,0.00000045,0.00008833,0.00000007,-
0.00000009,-0.00014447,0.00000014,0.00000013,-0.00002947,0.00000009,-
0.00000001,-0.00000681,-0.00000007,-0.00000009,0.00005686,0.00000005,-
0.00000010,0.00001900,0.00000003,0.,-0.00003126,0.,0.,-0.00001427,-
0.00000006,-0.00000007,0.00003559,0.,0.00000004,0.00000553,-
0.00009441,0.00013994,0.15621884,-
0.00509724,0.00294782,0.00000170,0.00060936,0.00160747,0.00000029,-
0.00028019,0.00176917,0.00000062,-0.00360406,-
0.00231211,0.00000037,0.00301970,-0.00281329,-0.00000134,-
0.03681778,0.00680296,0.00000884,0.00003685,-0.00030823,-0.00000016,-
0.00023193,0.00009718,0.00000009,0.00004024,-0.00012948,-
0.00000006,0.00003155,0.00007132,0.00000003,-
0.00006316,0.00001262,0.00000005,0.00010378,0.00003909,-
0.00000002,0.00000143,-0.00000040,-0.00000002,-0.00000220,0.00000133,-
0.00000002,-0.00000778,0.00000296,-0.00000001,0.00000785,-0.00000189,-
0.00000001,-0.24842566,0.06209935,0.00004626,0.67998163,-0.00225664,-
0.00426592,-0.00000099,0.00103930,0.00130677,0.00000033,-0.00067642,-
0.00073347,-0.00000009,0.00037212,0.00109474,0.00000033,-0.00742064,-
0.00185406,0.00000041,-0.00554344,0.02025385,0.00000537,-0.00028806,-
0.00029302,-0.00000001,-0.00004779,0.00019656,0.00000012,-0.00001995,-
0.00000943,-0.00000001,-0.00014744,0.00006763,0.00000004,0.00003862,-
0.00001450,-0.00000002,-0.00014175,-0.00002254,0.00000003,-0.00000924,-
0.00008495,-0.00000003,0.00005377,-0.00000146,0.,-0.00002280,0.00000903,0.,-
0.00001815,0.00001319,0.,0.11701131,-0.19178597,-
0.00005185,0.02749566,0.75118620,-0.00000001,-
0.00000213,0.00093282,0.00000034,0.00000023,-0.00025754,-0.00000028,-
0.00000042,-0.00005361,0.00000096,0.00000059,0.00037578,-0.00000199,-
0.00000063,0.00221158,0.00000581,0.00000373,0.00230032,-
0.00000015,0.00000002,-0.00015019,0.00000002,0.00000008,-0.00004855,0.,-
0.00000002,0.00004007,-0.00000005,0.00000002,-0.00001692,0.00000003,-
0.00000003,0.00003446,-0.00000006,0.,-0.00002040,0.00000001,-0.00000002,-
0.00000893,0.00000002,0.,-0.00000596,0.,0.,-0.00000129,0.,0.,-
0.00000251,0.00006007,-0.00004221,-0.06661381,-
0.00008809,0.00014561,0.13668228,-0.00501968,-0.00135374,0.00000102,-
0.00155749,-0.00116881,-0.00000012,-0.00004665,-0.00087656,-
0.00000012,0.00234298,-0.00025771,-0.00000085,-0.00428356,-

0.00294388,0.00000029,-0.00000632,-0.03353297,-0.00000782,-
0.00006518,0.00009490,0.00000007,0.00011138,0.00012661,0.00000003,0.000009
52,0.00015104,0.00000007,-0.00017731,-
0.00001012,0.00000003,0.00017069,0.00006017,-0.00000002,-0.00058681,-
0.00023383,-0.00000015,-
0.00018521,0.00000105,0.00000028,0.00009474,0.00001341,0.00000003,-
0.00000602,0.00000609,0.00000002,-0.00006194,0.00003154,0.00000004,-
0.12591021,0.02418667,0.00001661,0.03920149,0.02657173,0.00000118,0.747295
18,0.00322895,0.00613518,0.00000074,-0.00385521,-
0.00093259,0.00000032,0.00157476,-0.00097531,-0.00000062,0.00032848,-
0.00042715,-0.00000012,0.00227418,-0.00515238,-0.00000197,-0.02108680,-
0.01659566,-0.00000068,-0.00004050,-0.00005488,-0.00000005,-0.00005679,-
0.00003075,0.,-0.00001804,-0.00005221,-
0.00000004,0.00003950,0.00001131,0.00000002,-0.00007940,-0.00006383,-
0.00000003,0.00032651,0.00012793,0.00000009,0.00019994,-0.00009150,-
0.00000021,-0.00005671,-0.00000815,0.,0.00000018,-0.00000188,0.,0.00001657,-
0.00001114,0.,-0.03085751,-0.31506331,-0.00005573,0.06755052,-0.03163383,-
0.00002176,-0.02792439,0.68084758,0.00000201,0.00000113,0.00218588,-
0.00000065,-0.00000022,0.00037780,0.00000042,0.00000006,-0.00005568,-
0.00000058,-0.00000007,-0.00025618,0.00000197,-0.00000059,0.00097467,-
0.00000514,0.00000088,0.00227666,0.00000007,-0.00000003,0.00001855,-
0.00000005,-0.00000002,-0.00002203,0.,-0.00000006,0.00001305,0.00000005,0.,-
0.00000708,0.,0.,-0.00000018,0.00000004,-0.00000002,0.00006271,-
0.00000002,0.00000008,-0.00010703,-0.00000002,0.,0.00000051,0.,0.,-
0.00000241,0.,0.,-0.00000528,0.00000327,-0.00006487,-0.06663866,0.00001129,-
0.00001446,0.00879455,-0.00011208,0.00013667,0.13628343,-0.00180409,-
0.00289142,-0.00000041,0.00270793,0.00042888,-0.00000050,-
0.00106572,0.00119851,0.00000044,-0.00084454,-
0.00106121,0.00000007,0.00065014,0.00064132,-
0.00000005,0.00485985,0.00250782,0.00000097,0.00007979,-0.00000428,-
0.00000004,-0.00006799,-0.00003269,0.00000002,0.00001788,-0.00006735,-
0.00000004,0.00005598,0.00002621,-0.00000001,-
0.00006429,0.00000329,0.00000004,0.00016506,0.00004640,0.00000003,0.000071
36,0.00000401,-0.00000010,-0.00000762,-0.00000271,-0.00000001,0.00000104,-
0.00000258,0.,0.00001698,-0.00000730,-0.00000002,0.02171040,-0.07735195,-
0.00002189,-0.06590065,0.00237108,0.00001100,-0.30749063,-
0.05431202,0.00003065,0.67476847,-0.00199223,-
0.00085790,0.00000015,0.00029732,-0.00077432,-
0.00000029,0.00009647,0.00076339,0.00000036,-0.00103870,-
0.00007260,0.00000009,0.00175057,0.00107043,-0.00000011,0.00260039,-
0.00578836,-0.00000359,0.00004013,-0.00000205,0.00000005,-0.00000412,-
0.00001175,-0.00000002,0.00000916,0.00000060,0.,0.00007182,-0.00004424,-
0.00000001,0.00001380,0.00005906,0.00000001,-0.00008958,-0.00006081,-
0.00000006,-0.00010669,0.00007346,0.00000012,-
0.00000740,0.00000388,0.,0.00000576,0.00000086,0.,0.00000273,-0.00000073,0.,-
0.03164939,-0.01968943,-0.00000085,0.00295863,-0.00087995,0.00000087,-
0.12147326,-0.17537691,-
0.00000650,0.01864594,0.75335349,0.00000010,0.00000034,0.00031243,-
0.00000068,-0.00000001,-0.00077287,0.00000023,-0.00000009,-
0.00004371,0.00000003,0.00000024,0.00023139,0.00000006,0.00000019,-
0.00058272,0.00000086,-0.00000353,0.00660673,0.,0.,0.00005966,0.00000004,-

0.00000002,0.00003518,0.00000002,0.00000005,-0.00001142,0.,-
0.00000002,0.00000032,0.00000002,-0.00000002,0.00000794,-0.00000007,-
0.00000001,-0.00000038,-0.00000005,0.00000002,-0.00001608,0.,0.,-0.00000366,-
0.00000002,0.,-0.00000368,0.,0.00000001,-0.00000487,-
0.00001031,0.00000680,0.00675632,0.00001102,0.00000105,-
0.00646948,0.00001396,-0.00001814,-0.06041273,-
0.00009044,0.00014820,0.13441151,-0.00102570,-0.00082188,-0.00000012,-
0.00004872,0.00169117,0.00000053,-0.00067107,-0.00111073,-
0.00000016,0.00240983,0.00020174,-0.00000066,-0.00324234,-
0.00079659,0.00000062,0.00218713,0.00210138,0.00000005,-
0.00026851,0.00007006,0.00000011,0.00003321,0.00014648,0.00000004,0.000009
47,0.00006649,0.00000004,-0.00031253,0.00014015,0.00000011,0.00003801,-
0.00007412,-0.00000007,-0.00017072,-0.00001989,0.00000005,0.00005801,-
0.00012317,-0.00000008,0.00009148,-0.00000157,0.00000001,-0.00002270,-
0.00000148,0.,-0.00001908,0.00001951,0.00000001,-
0.01195222,0.02388387,0.00000686,0.02165219,-0.03166628,-0.00001032,-
0.05758400,-0.01723202,0.00000689,-
0.25419635,0.12183614,0.00006337,0.65521330,-0.00258035,-0.00213904,-
0.00000016,0.00216311,0.00103327,-0.00000007,-
0.00117650,0.00101486,0.00000047,-0.00008323,-0.00150612,-
0.00000041,0.00072880,0.00022302,-0.00000002,0.00238990,-0.00084929,-
0.00000042,-0.00006280,0.00001985,0.00000002,-
0.00006942,0.00006698,0.00000005,0.00003280,-0.00003548,-0.00000002,-
0.00011139,0.00010807,0.00000007,-0.00003643,-
0.00003092,0.,0.00002166,0.00000590,0.00000002,0.00006032,-0.00005969,-
0.00000009,0.00004689,-0.00000125,0.,-0.00001035,-0.00000269,0.,-
0.00000043,0.00000483,0.,0.02350455,-0.04861067,-0.00001450,-0.07709624,-
0.01978903,0.00000703,0.02903118,0.05938535,0.00000755,0.06407743,-
0.19444219,-0.00004314,-0.03046012,0.69608426,-0.00000044,-0.00000031,-
0.00013405,0.00000051,0.00000001,0.00002089,-
0.00000015,0.00000040,0.00004651,-0.00000040,-
0.00000043,0.00001897,0.00000066,0.00000021,-0.00013193,0.00000010,-
0.00000047,-0.00041763,0.00000004,-0.00000003,-0.00001317,-0.00000003,0.,-
0.00001532,-0.00000004,-0.00000006,0.00000515,0.,0.,-
0.00000056,0.,0.00000004,0.00000027,0.00000004,0.,-
0.00000587,0.00000003,0.,0.00001182,0.,0.,0.00000286,0.00000004,0.00000002,0.
00000031,0.,-0.00000003,0.00000035,0.00000648,-0.00001407,-0.00761652,-
0.00002120,-
0.00000113,0.00723990,0.00001862,0.00001544,0.00748043,0.00004939,-
0.00005324,-0.06261220,-
0.00009743,0.00014026,0.14366610,0.00203116,0.00062649,-0.00000020,-
0.00133514,-0.00060879,0.00000015,0.00069253,-0.00125836,-
0.00000052,0.00087186,0.00170379,0.00000031,-0.00362179,-
0.00066743,0.00000049,0.00059569,0.00594119,0.00000256,-0.00011294,-
0.00006327,-0.00000004,0.00005766,0.00001489,0.,-0.00003701,0.00003085,0.,-
0.00001027,-0.00003196,0.00000003,0.00002468,-0.00001427,-0.00000005,-
0.00000885,0.00001276,0.00000001,0.00000237,-0.00000943,0.,-0.00001716,-
0.00000425,0.00000001,-0.00000219,0.00000282,0.00000002,-
0.00000114,0.00000044,0.00000002,-0.05698234,0.02764606,0.00001800,-
0.31142146,-0.12234744,0.00001364,-0.02158331,-0.02993151,-
0.00000472,0.03995274,0.02619157,0.00000099,-

0.12458296,0.02514024,0.00001672,0.74982787,-0.00040850,-
0.00032347,0.00000009,-0.00068775,0.00043521,0.00000010,-0.00014386,-
0.00102501,-0.00000019,0.00183200,0.00109457,-0.00000015,-
0.00154452,0.00089461,0.00000062,0.00573635,-0.00149981,-0.00000313,-
0.00000760,0.00015497,0.00000010,0.00012016,-0.00002764,-0.00000005,-
0.00001350,0.00009809,0.00000006,-0.00007217,-0.00002695,-
0.00000005,0.00006532,-0.00000631,0.,-0.00015554,-0.00003320,0.00000002,-
0.00002498,-
0.00000429,0.00000003,0.00002298,0.00000503,0.00000002,0.00000154,-
0.00000042,0.,-0.00001526,0.00000733,0.,-0.01730750,0.05943149,0.00001660,-
0.05623959,-0.17648147,-0.00001893,-0.03109022,-0.04508910,-
0.00000404,0.06992652,-0.03256558,-0.00002236,-0.03279237,-0.32126020,-
0.00005713,-0.02543638,0.68475717,-0.00000040,0.00000003,-0.00059286,-
0.00000004,0.00000010,0.00022730,-0.00000010,-0.00000010,-
0.00004115,0.00000016,0.00000035,-
0.00077478,0.00000006,0.00000018,0.00029913,0.00000266,-
0.00000297,0.00663650,-0.00000001,0.00000008,-0.00002420,0.00000002,-
0.00000003,0.00003839,0.00000002,0.00000005,-0.00000739,0.,0.,0.00000537,-
0.00000001,-0.00000002,-0.00001156,-0.00000004,-0.00000001,0.00001291,-
0.00000002,0.,-0.00000291,0.,0.,-0.00000276,-0.00000001,0.,-0.00000372,0.,0.,-
0.00000164,0.00000690,0.00000802,0.00667923,0.00003042,-0.00000719,-
0.06080833,-0.00000504,-0.00000424,-0.00654493,0.00001150,-
0.00001462,0.00850815,0.00000270,-0.00006717,-0.06476056,-
0.00011543,0.00013974,0.13711262,-0.00198215,0.00047758,0.00000053,-
0.00051333,-0.00112301,-0.00000020,0.00068431,-0.00038911,-0.00000029,-
0.00009252,0.00036737,0.00000022,0.00018102,-0.00039740,-
0.00000018,0.00103831,0.00088298,-0.00000062,0.00008829,-0.00007580,-
0.00000005,-0.00000709,-0.00005284,-0.00000002,-0.00002090,-0.00002063,-
0.00000002,0.00008806,-0.00005071,-0.00000003,-
0.00000635,0.00000704,0.,0.00009812,0.00004419,0.,0.00001001,0.00001250,0.,-
0.00002970,-0.00000185,0.,0.00000524,0.00000074,0.,0.00000898,-
0.00000724,0.,0.00762218,0.02687569,0.00000587,-0.06018852,-0.01967239,-
0.00000080,-0.00498035,0.00207558,0.00000233,-
0.00103830,0.00020315,0.00000017,-0.00475469,-
0.00143540,0.00000182,0.00226955,-0.02943767,-0.00000704,0.05996352,-
0.00309505,0.00008453,0.00000050,0.00013826,0.00071775,0.00000029,-
0.00032647,0.00015859,0.00000005,-0.00010493,-0.00027855,-
0.00000011,0.00040119,0.00012965,-0.00000004,-0.00111692,-
0.00203247,0.00000053,0.00001843,0.00002294,-0.00000001,-
0.00000042,0.00000542,0.,0.00000744,0.00000734,0.,-
0.00000974,0.00000506,0.00000001,0.00000414,0.00000271,0.,-0.00002764,-
0.00001209,0.,-
0.00001000,0.00000264,0.00000001,0.00000638,0.00000484,0.,0.00000022,-
0.00000005,0.,-0.00000306,0.00000139,0.,-0.00107002,-0.01423881,-0.00000420,-
0.01756799,-0.34699492,-0.00007179,0.00314526,0.00033646,-
0.00000189,0.00000985,0.00059904,0.00000023,-0.00205493,0.00101322,-
0.00000127,0.00060822,-0.00771208,-0.00000288,0.02138576,0.36866639,-
0.00000060,0.,-0.00002388,0.00000012,0.00000050,-0.00044675,-
0.00000013,0.00000005,0.00031211,-0.00000002,-
0.00000027,0.00024236,0.00000024,0.00000009,-0.00016255,-
0.00000122,0.00000003,-0.00344717,0.00000001,0.,-0.00001377,0.,0.00000001,-

0.00000556,0.,0.,-0.00000322,-0.00000002,0.,0.00000313,0.,0.00000001,-
0.00000342,-0.00000002,-
0.00000001,0.00000029,0.,0.,0.00000161,0.,0.,0.00000517,0.,0.,0.00000179,0.,0.,0.
00000281,-0.00000066,-0.00000904,0.00349569,-0.00000053,-0.00007124,-
0.03884440,0.00000262,-0.00000169,0.00577877,0.00000013,0.00000020,-
0.00031983,0.00000167,-
0.00000139,0.00771287,0.00000032,0.00000231,0.00347927,-
0.00000066,0.00007957,0.02678659,-0.00005267,-
0.00023266,0.,0.00004429,0.00013935,-0.00000005,-0.00003663,-
0.00011472,0.00000003,0.00007636,0.00014492,-0.00000005,-0.00062500,-
0.00020587,0.00000006,-0.00005972,0.00134094,0.00000030,-0.00003832,-
0.00002847,0.00000007,-0.00000501,0.00002131,0.00000001,-0.00000262,-
0.00000125,0.00000001,-0.00001680,0.00000824,-0.00000002,0.00000068,-
0.00000372,0.,-0.00000561,0.00000010,0.,0.00000254,-0.00000845,-
0.00000001,0.00000391,-0.00000070,0.,-0.00000273,0.00000102,-0.00000001,-
0.00000086,0.00000126,-0.00000001,-0.00277276,-0.00358740,0.00000104,-
0.01760099,0.01574225,0.00000761,0.00010832,-0.00086344,-
0.00000030,0.00080355,-0.00135706,0.00000056,0.00589978,-0.00030416,-
0.00000047,-0.26149429,0.13194388,0.00007284,0.00107954,-0.00011873,-
0.00000101,0.27592181,0.00006491,-0.00002819,-0.00000004,-
0.00002754,0.00003485,0.00000004,0.00002872,-0.00006251,-0.00000004,-
0.00002507,0.00009400,0.00000009,-0.00033522,-0.00011561,0.00000008,-
0.00037558,0.00072079,0.00000021,-0.00001557,-0.00001913,-0.00000004,-
0.00000135,0.00000876,0.,-0.00000240,0.00000028,0.,-0.00000038,-
0.00000168,0.00000002,0.00000253,-0.00000015,0.,-0.00000595,-0.00000091,0.,-
0.00000256,-0.00000241,0.,-0.00000028,0.00000006,0.,-
0.00000085,0.00000092,0.,-0.00000166,0.00000051,0.,-0.00307831,-0.00109587,-
0.00000173,-0.01409985,0.01264709,0.00000479,-0.00068589,-
0.00051314,0.00000007,-0.00237703,-0.00554944,-0.00000236,0.02791101,-
0.01307292,-0.00000911,0.12978518,-0.14455015,-
0.00004951,0.00045160,0.00080857,0.00000121,-
0.14085915,0.14970111,0.,0.,0.00007541,-
0.00000002,0.,0.00002580,0.,0.00000001,-0.00003267,-0.00000004,-
0.00000003,0.00000284,-0.00000002,0.00000012,-0.00030696,-0.00000005,-
0.00000017,0.00024091,0.00000002,-0.00000002,0.00003586,0.,0.,-0.00000134,-
0.00000002,0.,-0.00000381,0.,0.,0.00000037,0.,0.00000002,-0.00000352,0.,0.,-
0.00000266,0.,0.,0.00000486,0.,0.,0.00000177,0.00000002,0.,0.00000113,0.,0.,0.00
000117,0.00000107,-0.00000153,0.00777201,0.00000008,-
0.00000050,0.00338100,-0.00000021,0.00000014,-0.00034653,0.00000022,-
0.00000250,0.00560508,0.00000650,-0.00000410,0.00384591,0.00007258,-
0.00004999,-0.03910894,-0.00000082,0.00000129,-0.00432511,-
0.00007970,0.00005566,0.02737320,0.00014661,0.00042702,0.00000013,-
0.00008113,-0.00011163,0.,0.00000635,0.00019233,0.00000014,-0.00021570,-
0.00009409,-0.00000014,-0.00199895,-0.00312682,-0.00000048,-
0.00101105,0.00044136,-0.00000031,0.00006425,-
0.00005732,0.00000001,0.00000425,-
0.00000170,0.00000002,0.00000520,0.00002406,0.,0.00006422,-0.00007288,-
0.00000004,0.00008834,0.00010200,0.00000004,-0.00020782,-0.00018090,-
0.00000020,-
0.00026329,0.00019688,0.00000042,0.00000987,0.00000925,0.,0.00000527,0.0000
0295,0.,-0.00000789,0.00000201,-0.00000001,0.00613823,-0.00002954,-

0.00000032,0.00085537,-0.00131251,0.00000053,-
0.25993644,0.13407331,0.00006925,-0.01792601,0.01594810,0.00000754,-
0.00279198,-0.00361001,0.00000094,0.00013335,-0.00084956,-
0.00000029,0.00029901,-0.00027854,0.,-0.00006052,0.00004380,-
0.00000018,0.27630066,-0.00037198,0.00017385,0.00000013,0.00035924,-
0.00028871,-0.00000018,0.00012925,0.00083648,0.00000019,-
0.00135565,0.00041664,0.00000049,0.00046472,0.00004177,-
0.00000028,0.00242866,0.00005978,0.00000041,0.00004682,0.00000519,0.,-
0.00000757,-0.00008494,-0.00000005,0.00000629,-0.00000863,0.,0.00011006,-
0.00005893,-0.00000005,-0.00000447,0.00005760,0.00000005,0.00001649,-
0.00006466,-0.00000005,-0.00014090,0.00023087,0.00000011,-
0.00001337,0.00000298,0.,0.00000952,-0.00000242,0.,0.00001976,-0.00000864,-
0.00000002,0.02793900,-0.01295855,-0.00000879,-0.00237224,-0.00557779,-
0.00000232,0.13203353,-0.14697052,-0.00004857,-
0.01421038,0.01240708,0.00000459,-0.00295108,-0.00100211,-0.00000154,-
0.00067726,-0.00055076,0.00000008,0.00157236,-0.00068387,-
0.00000061,0.00005631,0.00007735,0.00000027,-0.14272837,0.15228564,-
0.00000022,-0.00000014,-0.00016007,0.00000025,-
0.00000011,0.00024784,0.00000006,0.00000010,0.00031428,-
0.00000051,0.00000012,-0.00045389,0.00000056,0.00000070,-
0.00006040,0.00000032,0.00000086,-0.00349409,-0.00000007,0.,-0.00003379,-
0.00000002,-0.00000003,-0.00000832,-0.00000001,-0.00000003,-
0.00000315,0.00000002,0.00000002,0.00001226,-0.00000012,-0.00000002,-
0.00001590,0.00000031,0.00000019,-0.00010714,0.00000031,-
0.00000017,0.00024584,-0.00000002,0.,0.00000137,0.,0.,0.00000472,0.00000003,-
0.00000002,0.00000659,0.00000612,-0.00000422,0.00351535,0.00000034,-
0.00000256,0.00573060,0.00006845,-0.00004865,-0.03868957,0.00000050,-
0.00000057,0.00343790,0.00000112,-0.00000144,0.00767625,-
0.00000029,0.00000008,-0.00037329,0.00000044,-0.00000029,0.00068433,-
0.00000018,0.00000029,-0.00109567,-0.00007652,0.00005466,0.02679007,-
0.00056260,-0.00038441,-0.00000005,0.00013551,-0.00008458,-0.00000007,-
0.00008687,0.00007008,0.00000004,0.00008197,-0.00006148,-0.00000004,-
0.00010201,0.00011037,0.00000005,0.00088724,-0.00113955,-0.00000037,-
0.00001159,-0.00000190,0.,0.00000965,0.00001801,0.,0.00000061,0.00001535,0.,-
0.00001942,0.00000206,0.,0.00001685,-0.00000493,0.,-0.00006426,-
0.00000819,0.,-0.00001013,-0.00001887,0.00000001,0.00000939,0.00000180,0.,-
0.00000075,0.00000197,0.,-0.00001336,0.00000520,0.,-0.00494295,-
0.00145206,0.00000185,-0.00100201,0.00014942,0.00000017,0.00243017,-
0.02974376,-0.00000709,-0.06087221,-0.01852139,-
0.00000059,0.00660266,0.02711389,0.00000588,-
0.00496807,0.00222711,0.00000247,0.00008898,-0.00005332,-
0.00000024,0.00037384,0.00153881,0.00000043,0.00105004,0.00044067,-
0.00000081,0.06133413,-0.00031423,-
0.00023534,0.00000002,0.00011850,0.00004893,0.00000007,-0.00009404,-
0.00001125,-0.00000002,0.00014615,-0.00001658,0.00000004,-
0.00024209,0.00003771,0.,0.00071953,-0.00025210,-0.00000019,-
0.00002174,0.00000035,-
0.00000004,0.00000242,0.00001443,0.00000001,0.00000095,0.00000599,0.,-
0.00002472,0.00001034,0.00000003,0.00000567,-0.00000309,-0.00000001,-
0.00002748,-0.00000610,0.,0.,-0.00000896,0.,0.00000826,0.00000033,0.,-
0.00000196,0.00000020,0.,-0.00000391,0.00000324,0.,-0.00217154,0.00099421,-

0.00000133,-0.00002371,0.00061654,0.00000021,0.00072505,-0.00818730,-
0.00000294,-0.01626077,-0.34685080,-0.00007320,-0.00208689,-0.01346307,-
0.00000357,0.00318678,0.00046334,-0.00000189,-0.00004161,-
0.00005862,0.00000029,-0.00026341,-0.00066952,-0.00000029,-
0.00012547,0.00086261,0.00000129,0.02001871,0.36731553,-
0.00000004,0.00000010,-0.00030348,0.00000002,0.00000006,-0.00000272,0.,-
0.00000001,-0.00002962,0.00000002,0.,0.00002133,0.00000002,-
0.00000002,0.00006826,0.00000006,0.00000006,0.00028592,0.,0.,0.00000631,0.,0.
,-0.00000273,-0.00000002,-0.00000001,-
0.00000171,0.,0.,0.00000050,0.,0.00000002,-0.00000481,0.,0.,-
0.00000551,0.,0.,0.00000692,0.,0.,0.00000289,0.00000002,0.,0.00000118,0.,-
0.00000001,0.00000564,0.00000172,-
0.00000141,0.00771213,0.00000009,0.00000016,-
0.00028020,0.00000044,0.00000260,0.00332879,-0.00000013,-0.00007306,-
0.03787170,-0.00000130,-0.00000870,0.00273000,0.00000268,-
0.00000169,0.00583567,-0.00000022,0.00000029,-0.00118377,0.,-
0.00000059,0.00072793,-0.00000097,0.00000114,-0.00426114,-
0.00000109,0.00008036,0.02631717,-0.00769107,-0.04260865,-0.00000979,-
0.10087717,-0.00683452,0.00000522,0.00012182,0.03886120,0.00001195,-
0.00682336,0.00104154,0.00000365,0.00334977,-0.00050593,-0.00000194,-
0.00703160,0.00087664,0.00000369,0.00074313,0.00142315,0.00000040,-
0.00003371,-0.00007674,-0.00000003,0.00003371,-0.00002205,0.,-
0.00004898,0.00005654,0.00000003,0.00005738,0.00016149,0.00000012,-
0.00046880,0.00001646,0.00000006,0.00001407,0.00003332,0.,0.00001332,0.0000
3087,0.00000004,-0.00001288,0.00000121,0.,-
0.00004296,0.00002572,0.00000002,0.00052776,-0.00154729,-0.00000058,-
0.00002776,0.00007306,0.00000004,-
0.00046487,0.00006454,0.00000008,0.00002588,-0.00016317,-0.00000005,-
0.00008324,-0.00007834,-
0.00000002,0.00004278,0.00002053,0.00000002,0.00002309,-0.00002450,-
0.00000001,-0.00001279,-0.00000105,0.,0.00001908,-0.00002355,-0.00000002,-
0.00004777,-0.00002622,0.,0.70555399,-0.02408679,-0.02995788,-0.00000460,-
0.00677533,-0.20728151,-0.00003739,0.02035207,-0.03791886,-
0.00001643,0.00257579,0.00820018,-0.00000059,-0.00049704,-0.00478941,-
0.00000007,-0.00065453,0.00845330,0.00000045,0.00012301,-0.00043723,-
0.00000024,0.00005796,-0.00008497,0.00000007,-0.00006703,0.00004296,-
0.00000002,0.00018994,-0.00019959,-0.00000015,0.00010567,-0.00001462,-
0.00000003,-0.00000384,0.00021217,0.00000014,-
0.00006080,0.00009708,0.00000010,-
0.00007330,0.00005275,0.,0.00002128,0.00000169,-0.00000001,0.00001282,-
0.00001451,-0.00000002,-0.00024622,-0.00022126,-0.00000003,-0.00005978,-
0.00009149,0.,0.00008572,0.00019905,0.00000010,-
0.00010862,0.00002520,0.00000003,-0.00019527,-0.00018316,-
0.00000005,0.00006053,0.00003489,0.00000002,0.00007774,0.00004702,-
0.00000001,-0.00001493,-0.00000023,-0.00000001,0.00007149,0.00009273,0.,-
0.00000835,-0.00001588,-0.00000002,-0.00460014,0.63904214,-0.00000407,-
0.00000066,0.00420373,0.00000481,-0.00003594,-0.07237217,0.00000712,-
0.00002034,0.00424164,0.00000395,-0.00000096,0.00907341,-
0.00000192,0.00000040,-0.00486985,0.00000316,-0.00000067,0.00906018,-
0.00000004,-0.00000042,0.00008836,0.,-
0.00000003,0.00001858,0.00000008,0.00000007,0.00005961,0.00000010,-

0.00000007,-0.00001314,-0.00000006,-0.00000008,-
0.00002409,0.00000008,0.00000013,-0.00015037,-0.00000005,0.00000003,-
0.00001389,-0.00000006,0.00000005,-0.00003269,-0.00000007,-
0.00000004,0.00000633,0.00000001,0.00000003,0.00003584,-
0.00000016,0.00000033,0.00008835,0.,-
0.00000006,0.00001868,0.00000006,0.00000004,-0.00014459,-
0.00000002,0.00000009,-0.00002941,-0.00000002,-0.00000002,-0.00000685,-
0.00000006,0.00000004,0.00005677,-0.00000001,0.,-0.00003108,0.00000005,-
0.00000003,0.00000554,0.00000003,0.00000002,-0.00001452,0.00000002,-
0.00000006,0.00003558,-
0.00014570,0.00013429,0.15621698,0.00379597,0.00697807,0.00000188,0.005463
12,-0.01881983,-0.00000603,-0.00476988,0.00202702,0.00000209,-0.00001145,-
0.00068153,-0.00000020,-0.00108999,0.00114828,0.00000057,0.00075978,-
0.00289011,-0.00000102,-0.00008172,-0.00004196,-0.00000002,-
0.00001089,0.00056843,0.00000046,-0.00002302,-0.00015542,-0.00000007,-
0.00003742,0.00009686,0.00000004,-0.00008899,-0.00005900,0.,-0.00003578,-
0.00011980,-0.00000006,0.00002280,-0.00008793,-0.00000007,-
0.00017330,0.00020396,-0.00000018,-0.00003601,0.00002446,0.,-
0.00000461,0.00000052,0.,0.00004854,0.00028205,0.00000006,0.00005351,0.0001
7137,0.00000004,0.00006809,-0.00012548,-
0.00000005,0.00006777,0.00000388,0.00000002,0.00009174,0.00011286,-
0.00000001,-0.00004881,-0.00004673,0.00000001,-
0.00005869,0.00004471,0.00000002,0.00002061,0.00001082,-0.00000001,-
0.00002551,-0.00004042,-0.00000001,0.00001339,0.00001055,-0.00000001,-
0.28347229,-0.09675731,0.00003253,0.70891003,0.00236167,-0.00262857,-
0.00000173,-0.03120939,-0.02199475,0.00000176,-0.00311731,-0.00460683,-
0.00000060,-0.00125150,0.00193253,0.00000081,-
0.00129983,0.00007715,0.00000043,-0.00020629,-0.00326752,-
0.00000141,0.00009060,-0.00003889,0.00000007,0.00000680,-0.00045026,-
0.00000036,-0.00001908,0.00012780,0.00000005,0.00005530,-0.00012756,-
0.00000008,0.00010925,0.00004685,0.,0.00006156,0.00011693,0.00000005,-
0.00001600,0.00006352,0.00000005,0.00000712,-
0.00010345,0.00000017,0.00004383,-0.00003430,-0.00000002,0.00000649,-
0.00000199,0.,0.00030133,-0.00030439,-0.00000005,-0.00001007,0.00002733,0.,-
0.00027061,-0.00010327,0.00000003,0.00011325,-0.00003648,-0.00000004,-
0.00010609,0.00000744,0.00000002,-0.00002073,-0.00002913,-
0.00000002,0.00003556,-0.00002406,0.,-
0.00000539,0.00000046,0.,0.00001234,0.00002197,0.00000001,-0.00001520,-
0.00001203,0.,-0.04176006,-0.15633141,-0.00001271,-
0.04477081,0.72086276,0.00000010,-0.00000331,0.00220914,-0.00000946,-
0.00000206,0.00229766,0.00000071,-0.00000176,0.00094544,-
0.00000041,0.00000064,-0.00025688,-0.00000002,-0.00000021,-0.00005336,-
0.00000019,-0.00000031,0.00037681,0.00000005,0.00000003,0.00001912,0.,-
0.00000043,0.00006282,0.,0.00000009,-0.00000036,0.00000001,-0.00000005,-
0.00000588,0.00000005,0.00000003,0.00001304,0.00000004,0.00000008,-
0.00002040,0.,0.00000004,0.00000034,0.00000008,-0.00000008,-
0.00010677,0.00000003,-0.00000002,-0.00000550,0.,0.,-0.00000268,0.00000008,-
0.00000008,-0.00015035,-0.00000002,-0.00000002,-0.00002045,-0.00000009,0.,-
0.00004851,0.00000005,-0.00000004,0.00004010,-0.00000008,-0.00000002,-
0.00001695,0.,0.00000002,0.00003442,0.00000002,-0.00000002,-0.00000890,0.,0.,-
0.00000253,0.,0.00000002,-0.00000592,0.,-0.00000002,-

0.00000130,0.00004777,0.00000268,-0.06659301,-
0.00016824,0.00016702,0.13678402,0.00072153,-0.00096465,-0.00000044,-
0.00603524,-
0.00191767,0.00000315,0.00017200,0.00148438,0.00000011,0.00055379,-
0.00040330,-0.00000010,0.00001109,0.00053702,0.00000010,-0.00049241,-
0.00104531,-0.00000044,0.00000163,0.00000357,-0.00000002,-0.00001335,-
0.00016738,-0.00000015,0.00003601,0.00005485,0.00000003,-
0.00006180,0.00001666,0.00000003,0.00002355,0.00001338,0.,-
0.00000430,0.00002150,0.00000003,0.00000517,-0.00000032,0.,0.00010776,-
0.00009138,0.00000003,0.00000203,-0.00000358,0.,-
0.00000046,0.00000308,0.,0.00011891,-0.00012587,-
0.00000011,0.00003472,0.00013053,0.,-0.00006501,-0.00004963,0.,0.00005691,-
0.00004063,-0.00000002,0.00002237,0.00005340,0.00000005,-0.00002069,-
0.00003915,-
0.00000004,0.00001215,0.00001918,0.00000003,0.00001169,0.00000788,0.000000
01,-0.00000852,-0.00002802,0.,-0.00000276,-0.00000124,0.,0.02576756,-
0.03058836,-0.00001334,-0.13279240,-0.04680709,0.00000736,0.72320425,-
0.00183580,-0.00344961,-0.00000078,-
0.00211065,0.00512841,0.00000032,0.00044292,0.00153386,0.00000042,-
0.00048177,-0.00145419,-0.00000028,0.00165152,-0.00034394,-0.00000059,-
0.00091676,0.00245822,0.00000140,-0.00003685,0.00007573,-0.00000007,-
0.00002705,0.00012073,0.00000010,0.00004375,-0.00004093,-0.00000002,-
0.00002081,0.00006857,0.00000005,-0.00006599,-0.00000516,0.00000002,-
0.00000477,-0.00007317,-0.00000003,0.00000255,-0.00000886,-
0.00000002,0.00001566,0.00003836,-0.00000004,-0.00001410,0.00001157,0.,-
0.00000319,0.00000094,0.,-0.00007024,-0.00007646,-0.00000008,-0.00003782,-
0.00007681,0.,0.00005576,0.00009480,0.00000002,-
0.00008493,0.00000411,0.,0.00001314,-
0.00005952,0.,0.00004036,0.00003917,0.,0.00000368,-0.00001420,-0.00000001,-
0.00000788,-0.00000542,0.,-0.00000073,-0.00000346,0.,-
0.00000248,0.00000012,0.00000001,-0.07553802,-
0.02342522,0.00001226,0.01926259,-0.35493953,-
0.00008200,0.04094786,0.71165472,-0.00000059,-
0.00000053,0.00029959,0.00000292,-0.00000014,0.00663746,-0.00000014,0.,-
0.00059166,-0.00000014,-0.00000024,0.00022775,0.00000035,-0.00000023,-
0.00004122,-0.00000015,0.00000112,-0.00077426,-
0.00000003,0.00000002,0.00005683,0.00000001,0.00000007,-
0.00000016,0.00000001,0.00000002,0.00000790,0.00000002,0.,0.00000033,-
0.00000003,-0.00000003,-0.00001155,0.,-0.00000003,0.00003443,0.,0.,-
0.00000342,-0.00000003,0.00000002,-0.00001564,-0.00000001,0.,-
0.00000479,0.,0.,-0.00000350,-0.00000010,0.00000003,-0.00002428,0.,-
0.00000005,0.00001294,0.00000002,0.00000002,0.00003838,-
0.00000005,0.00000004,-0.00000738,0.,-0.00000003,0.00000537,0.,0.,-
0.00001158,0.,0.,-0.00000281,0.00000001,0.,-0.00000162,0.00000002,0.,-
0.00000278,0.,0.,-0.00000371,-0.00002578,0.00000052,0.00672919,0.00002539,-
0.00006375,-0.06078570,-0.00015238,0.00013842,0.13717158,-0.00060809,-
0.00224444,-0.00000059,-0.00203387,0.00005272,0.00000035,-
0.00039060,0.00221406,0.00000069,-0.00090647,-0.00166798,-
0.00000033,0.00158363,-0.00012522,-0.00000039,-
0.00057853,0.00180877,0.00000076,-0.00003959,0.00005331,0.00000003,-
0.00002317,0.00009934,0.00000006,0.00002327,-0.00004522,-

0.00000001,0.00001543,0.00004919,0.00000001,-0.00006784,-
0.00000131,0.00000002,0.00002567,-0.00007473,-0.00000004,-0.00000041,-
0.00000033,-0.00000001,-0.00001234,0.00002924,-0.00000002,-
0.00000695,0.00000515,0.,-0.00000205,0.00000031,0.,-0.00005524,-
0.00006005,0.,-0.00003909,-0.00009789,-
0.00000001,0.00002517,0.00007403,0.00000002,-
0.00006713,0.00001151,0.00000003,-0.00000959,-0.00006355,-
0.00000004,0.00003416,0.00003878,0.00000002,-0.00000299,-0.00002503,-
0.00000002,-0.00000942,-0.00000517,0.,0.00000225,0.00000463,0.,-0.00000257,-
0.00000135,0.,-
0.05991004,0.00409027,0.00001507,0.03754646,0.06796736,0.00001015,-
0.26904717,0.05798680,0.00007199,0.71311514,-0.00072013,-0.00240476,-
0.00000052,0.00033685,0.00336268,0.00000095,0.00045378,-0.00277838,-
0.00000089,-0.00119636,0.00188987,0.00000091,-0.00019080,-0.00123925,-
0.00000038,0.00152236,0.00148086,0.00000015,0.00017016,-0.00022680,-
0.00000012,0.00004961,-0.00014279,-0.00000004,-0.00009040,-0.00001163,-
0.00000001,0.00030062,-0.00021979,-
0.00000016,0.00003877,0.00003068,0.00000001,0.00014424,0.00007353,0.,-
0.00004145,0.00009348,0.00000007,-0.00015381,0.00001735,0.,0.00003186,-
0.00000226,0.,0.00000889,-0.00001637,0.,-0.00019313,-0.00019355,-0.00000002,-
0.00005594,-0.00012536,-0.00000003,-0.00014197,0.00007497,0.00000006,-
0.00002193,0.00004130,0.00000003,-0.00031499,-0.00019475,0.,0.00007615,-
0.00002732,-0.00000002,0.00015798,0.00000097,-0.00000002,-0.00002505,-
0.00000485,0.,0.00005307,0.00008796,0.,-0.00001013,-0.00002279,-
0.00000001,0.00370991,-0.00075980,0.00000094,0.02253673,-0.03579202,-
0.00001787,0.11593561,-0.17716732,-0.00006129,-0.00228012,0.63925472,-
0.00000003,0.00000007,-0.00013079,0.00000048,0.00000104,-
0.00041664,0.00000022,-0.00000133,-0.00013339,-
0.00000006,0.00000095,0.00002137,-0.00000049,-
0.00000031,0.00004639,0.00000051,-0.00000013,0.00001954,0.00000008,-
0.00000010,-0.00000682,0.00000002,-0.00000005,-0.00000705,-0.00000007,-
0.00000004,0.00000032,0.00000005,-0.00000007,-
0.00000054,0.00000006,0.00000006,0.00000533,0.00000002,0.00000004,-
0.00001688,0.,0.00000002,0.00000315,-
0.00000004,0.00000001,0.00001257,0.00000004,0.00000001,0.00000053,0.,-
0.00000003,0.00000040,0.,-0.00000005,-0.00001315,-0.00000003,-0.00000002,-
0.00000586,-0.00000002,0.00000003,-0.00001527,0.,-0.00000002,0.00000513,-
0.00000007,-0.00000004,-0.00000051,0.00000004,-
0.00000001,0.00000031,0.00000005,0.,0.00001206,-
0.00000004,0.00000001,0.00000036,0.,0.00000004,0.00000288,0.,0.00000001,0.00
000032,0.00001551,0.00000066,-0.00755152,-0.00000253,-
0.00002999,0.00727553,0.00008701,-0.00004534,-0.06483992,-
0.00015933,0.00012994,0.14363227,-0.00007573,-0.00133653,-0.00000059,-
0.00533760,0.00328723,0.00000405,0.00102175,0.00035834,-0.00000019,-
0.00021791,0.00139272,0.00000030,-0.00025513,-0.00056499,-
0.00000012,0.00064358,0.00017925,0.00000002,0.00014311,0.00009521,-
0.00000006,0.00002079,-0.00014424,-0.00000005,-0.00002067,0.00004527,-
0.00000001,0.00001895,-0.00006126,0.,0.00007171,0.00003214,-0.00000002,-
0.00006184,0.00007043,0.00000005,-
0.00000536,0.00002960,0.00000003,0.00000802,-
0.00002261,0.00000002,0.00001197,-0.00000884,0.,-

0.00000202,0.00000154,0.00000001,0.00000179,0.00000367,-
0.00000005,0.00001437,0.00017691,0.00000005,-0.00000989,-
0.00002933,0.,0.00002964,-0.00001592,-0.00000004,-0.00006156,-
0.00000396,0.00000004,0.00002099,-0.00006350,-
0.00000002,0.00011719,0.00007971,0.00000002,0.00000570,0.00000377,0.,0.0000
0549,-0.00000015,0.,-0.00000046,-
0.00000471,0.00000001,0.03774334,0.02243157,-0.00000207,-0.01951792,-
0.02995369,-0.00000442,-0.05585210,0.02930284,0.00002533,-0.28982517,-
0.10121913,0.00003535,0.71693036,-
0.00023206,0.00178669,0.00000082,0.00337714,0.00440725,-
0.00000140,0.00126379,-0.00368404,-
0.00000143,0.00125996,0.00215281,0.00000057,-0.00166664,-
0.00004813,0.00000040,0.00020199,-0.00156005,-0.00000071,0.00004092,-
0.00009204,-0.00000002,0.00002457,-0.00006338,-0.00000002,-
0.00003136,0.00003854,0.,-0.00002745,-0.00004470,-0.00000002,0.00007637,-
0.00001053,-0.00000003,-0.00003902,0.00009242,0.00000004,0.00000159,-
0.00000817,0.,-0.00000458,-0.00001137,0.,0.00000548,-
0.00000508,0.,0.00000272,-
0.00000032,0.,0.00004897,0.00007455,0.,0.00004055,0.00008999,0.00000002,-
0.00000075,-0.00006982,-0.00000003,0.00006035,-0.00001181,-
0.00000002,0.00004148,0.00007319,0.00000002,-0.00005243,-0.00002584,0.,-
0.00003035,0.00002755,0.00000003,0.00001371,0.00001058,0.,-0.00000483,-
0.00000898,0.,0.00000344,0.00000226,0.,0.06815460,-0.03584275,-0.00002979,-
0.02937482,-0.04715438,-0.00000281,-0.01437665,0.06346432,0.00001840,-
0.04338689,-0.15833942,-0.00001374,-0.04348678,0.70974212,-
0.00000020,0.00000106,-0.00058325,0.00000414,-
0.00000153,0.00660592,0.00000009,-
0.00000131,0.00031154,0.00000029,0.00000046,-0.00077337,-
0.00000039,0.00000017,-0.00004334,-0.00000002,-0.00000056,0.00023090,-
0.00000007,-0.00000002,-
0.00002942,0.,0.00000002,0.00001310,0.00000002,0.00000003,-
0.00001140,0.,0.,0.00000511,-0.00000002,-0.00000004,-0.00000734,0.00000002,-
0.00000002,0.00004008,0.,0.,-0.00000325,0.,-0.00000002,-0.00000311,-
0.00000001,0.,-0.00000173,0.,0.,-
0.00000384,0.,0.00000001,0.00005958,0.00000003,0.,-0.00000033,0.,-
0.00000004,0.00003513,0.00000002,0.00000002,-
0.00001136,0.00000002,0.00000003,0.00000026,-0.00000004,0.,0.00000793,-
0.00000005,-0.00000001,-0.00001594,0.00000002,0.,-0.00000487,0.,-0.00000001,-
0.00000368,0.,0.,-0.00000370,0.00000963,-0.00001731,0.00678914,-0.00000435,-
0.00000262,-
0.00654617,0.00001368,0.00000654,0.00844383,0.00005007,0.00000194,-
0.06257871,-0.00016888,0.00016533,0.13451516,-0.00461823,-
0.00209529,0.00000079,0.01118660,0.01462093,0.00000201,0.00253161,-
0.00759205,-0.00000214,0.00108725,0.00232747,0.00000065,-0.00104415,-
0.00099921,-0.00000017,0.00023544,0.00088754,0.00000026,-0.00003247,-
0.00031406,-0.00000019,0.00003527,-0.00017112,-0.00000009,-
0.00004168,0.00004787,0.,0.00009191,-0.00011252,-0.00000004,0.00005205,-
0.00001801,-0.00000004,0.00011088,0.00010058,0.00000001,-
0.00002074,0.00004500,0.00000005,-0.00006655,-
0.00003924,0.00000002,0.00002216,-0.00001084,0.00000001,0.00001397,-
0.00001037,0.00000001,-0.00008061,0.00004873,-0.00000004,-0.00009173,-

0.00061603,-0.00000009,-0.00002542,0.00013594,0.00000003,-
0.00009427,0.00007790,0.00000002,-0.00004848,-
0.00011379,0.00000001,0.00000290,0.00017651,0.00000003,-0.00020332,-
0.00018986,-0.00000012,-0.00004478,-
0.00002655,0.00000002,0.00003621,0.00009062,0.00000002,-0.00000562,-
0.00000045,0.00000002,-0.26520568,0.11095214,0.00008235,-0.05446240,-
0.01322720,0.00001369,-0.01282816,0.02489636,0.00000829,0.02493989,-
0.03053466,-0.00001298,-0.13219754,-
0.04681797,0.00000670,0.72179227,0.00311645,-0.00479968,-
0.00000257,0.02704938,-0.02778668,-0.00001553,-0.00300517,-0.00141847,-
0.00000031,-0.00036018,-0.00357786,-0.00000101,0.00145688,0.00002177,-
0.00000038,0.00148016,0.00168350,0.00000046,-0.00033555,-0.00023357,-
0.00000001,0.00001263,0.00004583,0.00000002,0.00001930,-0.00003776,-
0.00000002,0.00010360,0.00000811,-0.00000002,-0.00012360,-
0.00002236,0.,0.00024563,-0.00014630,-0.00000009,-0.00000743,0.00001910,0.,-
0.00003314,-0.00001439,-0.00000001,0.00000492,0.00000207,0.,0.00001231,-
0.00001034,-0.00000001,-0.00008467,-0.00003890,-0.00000002,-0.00005280,-
0.00036942,-0.00000007,-0.00004786,0.00010618,0.00000005,-
0.00009094,0.00005139,0.00000002,-0.00006435,-0.00011748,-
0.00000002,0.00003646,0.00010403,0.00000003,0.00000921,-0.00007337,-
0.00000004,-0.00004160,-0.00002840,0.,0.00002029,0.00005047,0.,-0.00000631,-
0.00000224,0.,0.05593212,-0.17617095,-
0.00004489,0.02769773,0.06227679,0.00000687,0.02605046,-0.05386723,-
0.00001973,-0.07679322,-0.02324878,0.00001274,0.02030618,-0.35040813,-
0.00008363,0.04281069,0.70645841,0.00000230,-
0.00000153,0.00097706,0.00000522,-0.00001187,0.00229189,-
0.00000094,0.00000094,0.00218522,-0.00000032,-
0.00000156,0.00037646,0.00000067,0.00000018,-
0.00005567,0.00000037,0.00000048,-0.00025599,-0.00000010,0.00000002,-
0.00014444,-0.00000002,0.00000006,-0.00002216,0.00000001,-
0.00000006,0.00003513,0.,0.00000004,-0.00001528,-0.00000003,-
0.00000002,0.00003839,0.00000005,-0.00000001,-0.00004854,0.,0.,-
0.00000552,0.,0.00000003,-0.00000821,-0.00000001,0.,-0.00000272,0.,0.,-
0.00000132,-0.00000001,-
0.00000005,0.00001874,0.00000002,0.00000010,0.00006262,0.,0.,-
0.00002206,0.,0.,0.00001312,-0.00000003,0.,-0.00000702,0.00000001,-
0.00000003,-0.00000022,0.00000006,0.00000007,-0.00010658,0.,0.,-
0.00000524,0.,0.,0.00000059,0.,0.,-0.00000240,0.00006758,-0.00005979,-
0.06669978,0.00002441,0.00001811,0.00872673,0.00000872,-0.00001934,-
0.00653273,-0.00002479,-0.00000012,0.00752804,0.00002413,-0.00006581,-
0.06038495,-0.00014263,0.00014374,0.13634814,0.00065427,-0.00191573,-
0.00000076,-0.00140594,-0.00220305,-
0.00000125,0.00014240,0.00037320,0.00000004,-0.00034251,-
0.00020578,0.00000004,0.00048622,-0.00040519,-
0.00000013,0.00088237,0.00071980,-
0.00000015,0.00005340,0.00000372,0.00000004,-0.00000111,-0.00006904,-
0.00000006,-0.00000327,0.00000514,0.00000001,0.00004140,-0.00002721,-
0.00000003,0.00000769,0.00001854,0.00000001,0.00002238,0.00001288,-
0.00000001,-0.00000198,0.00001791,0.00000001,0.00000279,-
0.00001312,0.,0.00000686,-0.00000325,0.,0.00000060,-0.00000113,0.,0.00002448,-
0.00002329,0.00000003,-0.00002868,-0.00011920,-0.00000007,-

0.00005849,0.00004374,0.,-0.00001814,0.00000737,0.00000002,-0.00004417,-
0.00004729,-0.00000002,0.00002326,0.00004237,0.00000001,0.00006973,-
0.00006907,0.,-0.00001786,-0.00001394,0.,0.00000644,0.00001527,0.,-
0.00000588,-0.00000311,0.,-0.02030939,-0.01264636,0.00000287,-
0.00235586,0.00424683,0.00000321,0.00028281,0.00058641,0.,0.00138633,0.0005
2647,0.00000172,0.00407279,0.00151088,0.00000023,-0.29046389,-
0.11437454,0.00003436,0.30694732,0.00168991,-0.00255153,-0.00000108,-
0.00022203,0.00054720,0.00000116,-
0.00042578,0.00017568,0.00000020,0.00026357,-0.00002653,-0.00000017,-
0.00046951,0.00035353,0.00000014,-0.00054494,-0.00068416,0.00000003,-
0.00009156,0.00005802,0.00000007,0.00000132,0.00008789,0.00000003,0.000009
83,-0.00000035,0.00000001,-0.00007579,0.00004870,0.00000003,-0.00000865,-
0.00001979,-0.00000001,-0.00003972,-0.00002588,0.,0.00000972,-0.00002299,-
0.00000002,0.00000935,0.00000991,0.,-0.00000970,0.00000277,0.,-
0.00000164,0.00000338,0.,0.00008244,0.00004096,0.,0.00007617,-0.00024874,-
0.00000001,0.00003789,-0.00002230,-0.00000002,-
0.00001077,0.00001465,0.00000001,0.00013554,0.00004935,-0.00000003,-
0.00006464,0.00012303,0.00000005,-0.00042378,-0.00012758,-
0.00000011,0.00000319,0.00000098,0.,-
0.00000724,0.00000628,0.,0.00000348,0.00000876,0.,0.01534100,0.01342576,-
0.00000136,0.00318622,-0.00234645,-0.00000310,0.00076590,-0.00069718,-
0.00000029,0.00119247,-0.00518603,-0.00000387,-0.02861365,-
0.00957082,0.00000398,-0.11645268,-
0.11654675,0.00000934,0.12490020,0.12164721,0.00000038,-0.00000032,-
0.00006058,-0.00000075,0.00000159,-0.00353481,-0.00000024,0.00000011,-
0.00016152,0.00000020,-0.00000008,0.00024715,-
0.00000017,0.00000011,0.00031356,-0.00000052,-0.00000020,-0.00045155,-
0.00000005,0.00000003,-0.00001457,0.,0.00000006,0.00000048,0.,0.,-0.00000367,-
0.00000004,0.00000002,0.00000287,-0.00000001,0.,-0.00000277,-0.00000002,-
0.00000002,-0.00000598,0.,-
0.00000002,0.00000515,0.,0.,0.00000147,0.,0.,0.00000290,0.,0.,0.00000178,0.0000
0003,0.00000004,-0.00003286,0.,-0.00000013,-0.00010610,0.00000002,0.,-
0.00000819,0.,0.,-0.00000291,0.00000007,0.00000002,0.00001245,-
0.00000002,0.00000007,-0.00001565,-0.00000015,-
0.00000014,0.00026301,0.,0.,0.00000691,0.,0.,0.00000150,0.,0.,0.00000472,0.0000
1032,0.00000630,0.00347901,0.00000311,-0.00000331,0.00572961,0.00000008,-
0.00000026,-0.00037367,0.00000198,-0.00000357,0.00767757,-0.00000777,-
0.00000374,0.00342158,0.00003360,0.00000832,-0.03874238,-0.00003878,-
0.00000674,0.02690598,0.00006011,0.00014765,0.00000008,0.00021462,-
0.00033032,-0.00000015,-0.00001449,-0.00000292,-0.00000004,0.00005562,-
0.00007199,-0.00000012,-0.00001977,0.00005530,0.00000007,-0.00002850,-
0.00008213,-0.00000007,-0.00000878,0.00000367,0.00000005,-0.00000029,-
0.00000260,0.,0.00000212,0.00000318,0.,-0.00001269,0.00000711,-
0.00000002,0.00000102,-0.00000078,0.,-0.00000383,-0.00000070,0.,0.00000174,-
0.00000280,0.,0.00000490,-0.00000321,0.,0.00000002,-
0.00000153,0.,0.00000007,0.00000057,0.,0.00000800,0.00001072,0.00000004,-
0.00000519,-0.00000665,0.,0.00000447,0.00000064,0.,-
0.00000224,0.00000045,0.,0.00001260,0.00000609,-
0.00000002,0.00000144,0.00000345,0.,-0.00000095,-0.00000530,0.,-0.00000157,-
0.00000294,0.,-0.00000184,-0.00000172,0.,-
0.00000004,0.00000149,0.,0.00106922,-0.00130128,0.00000147,0.00016682,-

0.00064831,-0.00000029,-0.00323315,-0.00418535,0.00000135,-
0.01936143,0.01695121,0.00001047,-
0.25988826,0.13131491,0.00009458,0.00703907,-0.00337501,-
0.00000190,0.00077416,0.00028143,-0.00000127,0.27331525,-0.00020358,-
0.00012504,-0.00000002,0.00152745,0.00042088,-0.00000027,0.00006822,-
0.00078355,-0.00000019,0.00013057,0.00012866,0.00000009,-0.00010879,-
0.00007851,-0.00000004,0.00012531,0.00009410,0.00000004,-0.00001412,-
0.00005478,-0.00000005,0.00000323,-0.00000777,0.,-0.00000495,-
0.00000034,0.,0.00001601,-0.00001256,0.,0.00000031,-
0.00000373,0.,0.00002517,0.00000505,0.,-0.00000229,0.00000351,0.,-0.00000973,-
0.00000202,0.,0.00000234,-0.00000053,0.,0.00000248,-0.00000187,0.,-
0.00000939,-0.00001900,-0.00000003,-0.00002593,-0.00006507,-0.00000001,-
0.00001504,0.00001969,0.,-0.00001368,0.00000920,0.,-0.00002062,-
0.00002175,0.00000001,0.00001385,0.00001197,0.,0.00001385,-0.00001769,-
0.00000001,-0.00001201,-0.00000867,0.,0.00000468,0.00001145,0.,-0.00000238,-
0.00000047,0.,-0.00201673,-0.00502980,-0.00000296,-0.00082614,-
0.00054514,0.00000014,-0.00322907,-0.00126573,-0.00000097,-
0.01224758,0.01237553,0.00000589,0.13377425,-0.14741723,-
0.00006499,0.02706979,-0.01279609,-0.00001158,-
0.00027241,0.00114764,0.00000153,-0.14240959,0.15504638,-0.00000006,-
0.00000011,0.00006816,0.00000040,0.00000015,0.00028655,-0.00000004,-
0.00000012,-0.00030367,0.00000002,0.00000003,-0.00000269,-0.00000003,-
0.00000003,-0.00002961,0.00000003,0.00000004,0.00002140,0.00000002,-
0.00000003,0.00003557,0.,0.,-0.00000242,-0.00000002,-0.00000001,-
0.00000368,0.,0.,0.00000032,0.00000001,0.00000002,-0.00000374,0.,0.,-
0.00000127,0.,0.,0.00000179,0.,0.,0.00000474,0.00000001,0.,0.00000118,0.,-
0.00000001,0.00000114,0.,-0.00000002,0.00000633,-0.00000002,-0.00000003,-
0.00000546,0.,0.00000002,-0.00000271,-0.00000001,0.,-0.00000170,0.,-
0.00000001,0.00000053,0.00000002,0.,-0.00000479,0.00000001,0.,0.00000719,-
0.00000001,0.,0.00000566,0.,0.,0.00000290,0.,0.,0.00000118,0.00000132,-
0.00000314,0.00771664,-0.00000041,0.00000007,-0.00028285,0.00000161,-
0.00000073,0.00583460,0.00000281,-0.00000177,0.00272832,0.00009500,-
0.00006449,-0.03791202,0.00000621,-0.00000329,0.00334026,-
0.00000136,0.00000142,-0.00424996,-
0.00010427,0.00007248,0.02634267,0.00014864,-0.00037430,-0.00000020,-
0.00108106,0.00241754,-
0.00000004,0.00050717,0.00152270,0.00000023,0.00083560,-0.00091159,-
0.00000073,0.00059784,0.00037462,0.00000015,-
0.00034416,0.00025031,0.00000020,0.00001835,0.00002872,0.00000002,-
0.00002852,0.00009017,0.00000002,0.00002844,-0.00002861,-0.00000001,-
0.00005736,0.00005636,0.00000003,-0.00001748,-0.00000200,0.,-0.00006769,-
0.00003945,-0.00000002,0.00000558,-0.00001494,-
0.00000001,0.00013272,0.00003594,0.00000001,-0.00001727,0.00001467,0.,-
0.00000596,0.00000346,0.,0.00006403,-0.00000436,-
0.00000001,0.00000832,0.00007941,0.00000002,0.00002517,-0.00001829,-
0.00000001,0.00000575,-0.00002283,-0.00000002,0.00005203,0.00003055,0.,-
0.00000433,-0.00000545,0.,0.00000347,0.00001385,0.,0.00000779,0.00000373,0.,-
0.00000557,-0.00002016,0.,0.00000086,0.00000241,0.,-
0.02011271,0.01689658,0.00001098,-
0.25871591,0.13244413,0.00009579,0.00727589,-0.00348770,-
0.00000198,0.00107308,-0.00131128,0.00000149,0.00010645,-0.00066729,-

0.00000029,-0.00324850,-0.00414106,0.00000132,-
0.00099865,0.00102857,0.00000074,0.00001957,0.00009346,-
0.00000031,0.27277586,0.00042654,0.00017092,0.00000004,0.00042613,0.000193
27,0.00000089,-0.00217033,-0.00250393,-0.00000023,0.00034968,-0.00063082,-
0.00000017,0.00043979,0.00024529,-0.00000010,-0.00022212,-0.00002448,0.,-
0.00007540,0.00005036,0.00000006,-0.00010950,-0.00024878,-
0.00000023,0.00008137,0.00011691,0.00000007,-
0.00012713,0.00005570,0.00000007,0.00001371,0.00001531,0.,-0.00003065,-
0.00001505,0.00000001,0.00000743,0.00000724,0.,0.00039073,-
0.00019577,0.00000004,-0.00000451,-0.00000147,-0.00000002,-
0.00000234,0.00000612,0.,0.00009034,0.00004721,-
0.00000002,0.00000766,0.00007796,0.00000002,0.00003415,-0.00002710,-
0.00000001,0.00000515,-0.00001965,0.,0.00007168,0.00004122,0.,-
0.00000811,0.00000082,0.,-0.00000862,0.00000924,0.,0.00000798,0.00000263,0.,-
0.00001190,-0.00001913,0.,0.00000178,0.00000432,0.,-
0.01108980,0.01325712,0.00000568,0.13471960,-0.14796935,-
0.00006625,0.02653530,-0.01271514,-0.00001160,-0.00192557,-0.00482488,-
0.00000273,-0.00086671,-0.00053883,0.00000017,-0.00307688,-0.00138998,-
0.00000108,-
0.00082350,0.00061369,0.00000022,0.00008166,0.00001096,0.00000029,-
0.14429961,0.15556730,-0.00000003,0.00000009,-0.00016480,-
0.00000052,0.00000062,-0.00348046,-0.00000070,-0.00000107,-0.00005395,-
0.00000030,0.00000012,-0.00045006,0.00000009,-
0.00000017,0.00031180,0.00000003,-0.00000007,0.00024151,-0.00000003,-
0.00000003,-0.00003161,-0.00000003,0.00000020,-0.00010717,0.00000001,-
0.00000003,-0.00001618,0.,0.,0.00001190,0.,0.,-0.00000272,-0.00000001,0.,-
0.00000904,0.,0.,0.00000163,-
0.00000002,0.,0.00025761,0.,0.,0.00000697,0.,0.,0.00000484,0.,0.,-0.00001415,0.,-
0.00000001,0.00000026,0.,0.,-0.00000555,-0.00000002,0.,-
0.00000326,0.00000001,0.,0.00000315,0.00000001,0.,-
0.00000345,0.,0.,0.00000167,0.,0.,0.00000281,0.,0.,0.00000514,0.,0.,0.00000179,0.
00000347,-0.00000231,0.00351535,0.00009667,-0.00006569,-
0.03887262,0.00000598,-0.00000330,0.00349638,0.00000133,-
0.00000289,0.00771691,-0.00000032,0.00000008,-0.00032193,0.00000159,-
0.00000075,0.00577757,0.00000024,-0.00000032,0.00068364,-
0.00000031,0.00000030,-0.00118334,-0.00010600,0.00007397,0.02684248,-
0.00000879,-0.00002032,-
0.00000005,0.00010771,0.00027842,0.00000006,0.00003836,-
0.00011706,0.00000003,-0.00001113,0.00005920,-0.00000003,-0.00001873,-
0.00003895,0.00000002,0.00003764,0.00004747,-0.00000006,0.00000359,-
0.00000841,0.00000004,-0.00000300,0.00000240,-0.00000001,0.00000182,-
0.00000156,0.,0.00000699,-0.00000431,-0.00000002,-0.00000111,-
0.00000050,0.,0.00000329,-0.00000068,0.,-
0.00000122,0.00000139,0.,0.00000097,0.00000322,0.,-
0.00000044,0.00000231,0.,0.,-0.00000074,0.,-0.00000481,-
0.00000373,0.00000005,0.00000046,0.00000249,0.,-
0.00000330,0.00000036,0.,0.00000119,0.00000021,0.,-0.00000889,-0.00000418,-
0.00000001,0.00000061,-
0.00000246,0.,0.00000355,0.00000196,0.,0.00000035,0.00000097,0.,0.00000144,0.
00000169,0.,-0.00000006,-
0.00000069,0.,0.00136839,0.00067614,0.00000205,0.00437388,0.00141004,0.0000

0009,-0.28717990,-0.11493747,0.00003972,-0.02030926,-0.01273987,0.00000342,-
0.00232553,0.00420325,0.00000335,0.00031444,0.00056432,-0.00000005,-
0.00000032,-0.00007852,-0.00000033,-0.00095941,-
0.00076724,0.00000025,0.00073415,-0.00024383,-0.00000150,0.30336354,-
0.00014956,-0.00073139,-0.00000009,-
0.00143734,0.00055300,0.00000047,0.00017978,-0.00011853,-0.00000008,-
0.00010722,0.00009002,0.00000003,0.00010445,-0.00008018,-0.00000004,-
0.00012273,0.00013250,0.00000004,0.00000549,-0.00001661,0.,0.00001752,-
0.00007011,-0.00000006,-0.00001146,0.00001449,0.,0.00001546,-0.00002131,-
0.00000002,0.00001527,0.00000738,0.,0.00001552,0.00001796,0.,-
0.00000285,0.00001154,0.,-0.00001429,-0.00001766,0.00000002,0.00001135,-
0.00000980,0.,0.00000216,-0.00000033,0.,0.00000918,-0.00005254,-0.00000001,-
0.00000358,-0.00000692,0.,-0.00002229,0.00000706,0.,-0.00000090,-
0.00000345,0.,-0.00001280,-0.00000959,0.,0.00000380,-
0.00000006,0.,0.00000780,-0.00000347,0.,-0.00000195,-
0.00000069,0.,0.00000214,0.00000251,0.,-0.00000256,-0.00000174,0.,0.00119091,-
0.00524096,-0.00000371,-0.02839937,-0.00931107,0.00000463,-0.11715183,-
0.11900957,0.00001195,0.01542039,0.01305207,-0.00000188,0.00318254,-
0.00240057,-0.00000295,0.00074691,-0.00071793,-0.00000030,-
0.00009099,0.00001732,0.00000031,0.00103382,0.00066496,-
0.00000027,0.00031651,0.00116268,0.00000133,0.12497905,0.12247027,-
0.00000009,-0.00000009,-0.00030696,-
0.00000035,0.00000002,0.00024024,0.00000006,0.,0.00007550,-
0.00000002,0.,0.00002582,0.00000003,0.,-0.00003264,-
0.00000006,0.00000003,0.00000287,0.00000001,-0.00000002,0.00000565,0.,-
0.00000001,-0.00000521,-0.00000002,-0.00000001,-
0.00000484,0.,0.,0.00000032,0.00000001,0.00000002,-0.00000162,0.,0.,-
0.00000250,0.,0.,0.00000281,0.,0.,0.00000690,0.00000002,0.,0.00000565,0.,0.,0.00
000117,0.00000002,-0.00000004,0.00003589,-0.00000001,-0.00000001,-
0.00000268,0.,0.00000001,-0.00000132,0.,0.,-
0.00000380,0.,0.,0.00000037,0.00000002,0.,-0.00000350,0.,0.,0.00000486,-
0.00000001,0.,0.00000116,0.,0.,0.00000177,0.,0.,0.00000112,0.00000204,-
0.00000365,0.00777483,-0.00000776,-
0.00000378,0.00336808,0.00003951,0.00001160,-
0.03916141,0.00001065,0.00000581,0.00379849,0.00000298,-
0.00000328,0.00560026,-0.00000003,-0.00000027,-0.00034738,-
0.00000031,0.00000033,-0.00109562,0.00000072,0.00000019,0.00072706,-
0.00000132,0.00000151,-0.00431312,-0.00004502,-0.00001023,0.02749337,-
0.00044091,-0.00066622,-0.00000002,-0.00026546,0.00073539,-
0.00000009,0.00000920,0.00016838,0.00000011,0.00018992,-0.00006800,-
0.00000012,-0.00012499,-0.00010565,0.00000006,0.00026906,0.00040536,-
0.00000004,-0.00000858,0.00001862,0.00000005,-
0.00003637,0.00007065,0.00000002,0.00004138,-0.00001987,0.,-
0.00001994,0.00003108,0.00000003,-0.00003446,-0.00000719,-
0.00000001,0.00000523,-0.00003323,-0.00000003,0.00000132,-
0.00001143,0.,0.00003854,-0.00000266,0.,-0.00001808,0.00001922,0.,0.00000055,-
0.00000228,0.,-0.00004156,-0.00004607,0.00000003,-0.00005201,-0.00007575,-
0.00000005,-0.00000721,0.00004526,0.,-0.00003566,0.00001559,0.00000002,-
0.00006978,-0.00005807,0.,0.00005578,0.00001132,-0.00000001,0.00005633,-
0.00000216,0.,-0.00002111,-0.00001750,0.,0.00000951,0.00002176,0.,-
0.00000107,-0.00000083,0.,-0.00407002,-0.00093591,0.00000064,-

0.00525606,0.00720311,0.00000536,0.01394755,0.02292255,0.00000263,-
0.09923572,0.00470466,0.00000695,0.00614753,-0.03004355,-0.00000950,-
0.00638883,-0.00361374,0.00000289,0.00010801,-0.00151591,-0.00000034,-
0.00233830,-0.00085391,-0.00000068,0.00024492,0.00161524,0.00000047,-
0.00320383,0.00257650,0.00000054,0.88843111,0.00241941,0.00524157,0.000001
02,-0.00062378,-0.00953667,-0.00000219,-
0.00171712,0.00547320,0.00000195,0.00159301,-0.00363613,-
0.00000152,0.00017171,0.00228513,0.00000069,-0.00210391,-0.00342024,-
0.00000072,-0.00035779,0.00049090,0.00000031,-0.00006575,0.00016475,-
0.00000003,0.00016820,0.00006084,0.00000005,-
0.00063203,0.00042229,0.00000031,-0.00003346,-0.00004789,-0.00000002,-
0.00029206,-0.00012995,0.00000001,0.00007787,-0.00016164,-
0.00000013,0.00027690,-0.00004351,0.00000002,-0.00003828,-0.00001915,-
0.00000001,-
0.00001275,0.00003415,0.00000002,0.00042659,0.00043918,0.00000011,0.000074
92,0.00016725,0.00000003,0.00028281,-0.00015569,-0.00000011,0.00002294,-
0.00006959,0.,0.00066584,0.00039348,-0.00000003,-
0.00014892,0.00007876,0.00000004,-0.00029065,-
0.00000831,0.00000004,0.00002644,-0.00000805,-0.00000002,-0.00009911,-
0.00015420,-
0.00000001,0.00001550,0.00004923,0.,0.00045151,0.00358006,0.00000095,0.0028
5321,0.00363330,-0.00000158,0.01431367,-0.02974656,-0.00001241,-0.00737226,-
0.19828495,-0.00002975,-0.01673373,-0.01520082,0.00000098,-
0.00103607,0.00497252,-0.00000025,0.00043200,0.00062098,-
0.00000004,0.00287634,0.00156769,0.00000054,-
0.00035233,0.00035821,0.00000011,-
0.00275215,0.00082176,0.00000198,0.17324129,0.71251044,0.00000078,0.000001
51,0.00008892,-0.00000038,-0.00000246,-0.00120939,-
0.00000043,0.00000141,0.00009213,0.00000034,-0.00000096,-
0.00007523,0.00000015,0.00000059,0.00015187,-0.00000064,-0.00000095,-
0.00009613,-0.00000014,0.00000018,-0.00001717,0.,0.00000004,-
0.00000488,0.00000007,0.00000007,-0.00000456,-
0.00000015,0.00000010,0.00000062,-0.00000004,-0.00000007,0.00000024,-
0.00000007,-0.00000003,-0.00000560,0.00000002,-
0.00000004,0.00000260,0.00000006,-0.00000003,0.00001135,-0.00000002,-
0.00000002,0.00000509,0.,0.00000002,0.00000240,0.00000009,0.00000015,-
0.00001693,0.00000005,0.00000007,-0.00000643,0.00000006,-0.00000007,-
0.00000663,0.00000003,0.,0.00000042,0.00000019,0.00000012,0.00000052,-
0.00000007,0.00000002,-0.00000413,-0.00000009,-
0.00000001,0.00001216,0.00000003,0.,0.00000515,-0.00000002,-
0.00000006,0.00000283,0.,0.,0.00000248,0.00000117,0.00000160,-
0.00077927,0.00000420,-0.00000320,0.00714716,-0.00000009,-
0.00001412,0.00202980,0.00000354,-0.00003214,-0.07777884,-
0.00000587,0.00000409,0.00005915,0.00000350,0.00000017,0.00787069,0.000000
18,0.00000048,0.00035390,0.00000037,0.00000173,-0.00398996,-0.00000006,-
0.00000049,0.00033607,-0.00000076,0.00000042,-0.00387460,-
0.00018183,0.00006899,0.20049405,-0.00012832,-0.00069444,-
0.00000023,0.00017576,0.00120334,0.00000038,0.00034800,-0.00094523,-
0.00000038,-0.00045738,0.00058892,0.00000034,0.00017620,-0.00037041,-
0.00000021,0.00022894,0.00050041,0.00000021,0.00006992,-0.00009565,-
0.00000010,0.00003548,-0.00009387,-0.00000003,-

0.00005430,0.00000662,0.,0.00012619,-0.00009624,-
0.00000006,0.00002896,0.00001617,0.,0.00005233,0.00004419,0.00000001,-
0.00001542,0.00003986,0.00000003,-0.00006871,0.00000193,0.,0.00002000,-
0.00001039,0.,0.00000218,-0.00000452,0.,-0.00005288,-0.00005723,-
0.00000005,0.00001724,0.00002893,0.00000002,-
0.00004633,0.00000205,0.00000002,0.00001643,0.00000126,-0.00000001,-
0.00008158,-0.00003864,0.00000002,-0.00000281,-
0.00002560,0.,0.00002902,0.00000925,0.,0.00000822,0.00001238,0.,0.00001207,0.
00001346,0.,-0.00000251,-
0.00000885,0.,0.00012346,0.00042640,0.00000009,0.00101863,-0.00201069,-
0.00000105,-0.00682692,0.00605960,0.00000343,-0.01138244,-
0.03095004,0.00000241,0.00327986,0.00041074,-0.00000010,0.00067081,-
0.00134304,-0.00000112,-0.00001162,-0.00018896,-
0.00000012,0.00105277,0.00080374,0.00000024,0.00001465,-0.00032645,-
0.00000008,0.00279381,-0.00086145,-0.00000114,-0.57292077,-
0.26482102,0.00010037,0.68497356,-0.00104920,-0.00245627,-
0.00000053,0.00012401,0.00408458,0.00000109,0.00080919,-0.00240839,-
0.00000086,-0.00075567,0.00155985,0.00000069,0.00004231,-0.00102330,-
0.00000037,0.00085099,0.00156742,0.00000036,0.00016980,-0.00022878,-
0.00000014,0.00003135,-0.00006858,0.00000001,-0.00008106,-0.00003171,-
0.00000002,0.00029774,-0.00019478,-
0.00000015,0.00001334,0.00002216,0.00000001,0.00013369,0.00005838,0.,-
0.00003554,0.00007312,0.00000006,-
0.00013005,0.00002407,0.,0.00001782,0.00000864,0.,0.00000482,-0.00001533,-
0.00000001,-0.00019244,-0.00020225,-0.00000004,-0.00001843,-0.00003557,-
0.00000001,-0.00012841,0.00006046,0.00000004,0.00000059,0.00002547,0.,-
0.00029807,-0.00016925,0.00000002,0.00005794,-0.00004722,-
0.00000002,0.00012918,0.00001294,-0.00000001,-
0.00000504,0.00000986,0.,0.00004263,0.00006201,0.,-0.00000646,-0.00002316,0.,-
0.00148690,-0.00160186,-0.00000004,-0.00074581,0.00077189,0.00000050,-
0.00330652,-0.00058893,0.00000095,-0.04624044,-
0.02985116,0.00000291,0.00681414,-0.00561183,-0.00000395,-0.00194992,-
0.00253885,0.00000005,-0.00005193,-0.00075424,-0.00000013,-
0.00040620,0.00008433,0.00000006,0.00012867,-0.00012945,-0.00000006,-
0.00071152,-0.00158895,-0.00000003,-0.26456910,-
0.27201761,0.00003942,0.30620440,0.27180732,-0.00000023,-0.00000040,-
0.00000512,0.,0.00000069,0.00013949,0.00000011,-0.00000034,0.00002650,-
0.00000007,0.00000023,-0.00000592,-0.00000005,-0.00000014,-
0.00002970,0.00000013,0.00000022,0.00003275,0.00000005,-
0.00000006,0.00000205,0.,0.,0.00000764,-0.00000003,-
0.00000005,0.00000564,0.00000003,-0.00000002,-
0.00000062,0.00000002,0.00000004,0.00000166,0.,0.,0.00000298,0.,0.,-
0.00000310,-0.00000001,0.00000002,-0.00001306,0.00000001,0.00000001,-
0.00000650,0.,-0.00000002,-0.00000217,0.,-0.00000005,0.00000477,-0.00000002,-
0.00000002,0.00000532,0.,0.00000002,0.00000368,-0.00000001,-
0.00000002,0.00000116,-0.00000005,-0.00000003,-
0.00000046,0.00000003,0.,0.00000329,0.00000002,0.00000001,-0.00000845,-
0.00000002,0.,-0.00000364,0.,0.00000002,-0.00000296,0.,0.00000001,-
0.00000196,-0.00000045,-0.00000065,0.00005914,-0.00000060,0.00000103,-
0.00056261,0.00000140,-0.00000226,0.00014289,-0.00000146,-
0.00000261,0.01900377,0.00000125,-0.00000197,0.00264420,-

0.00000096,0.00000019,-0.00156159,-0.00000009,-0.00000004,-0.00026610,-
0.00000027,-0.00000042,0.00064001,0.00000003,0.00000007,0.00003991,-
0.00000111,-0.00000009,-0.00033061,0.00010045,0.00004004,-0.07428025,-
0.00014091,-0.00004245,0.03212480,0.00072484,0.00129756,0.00000022,-
0.00009180,-0.00214575,-0.00000058,-
0.00026866,0.00116342,0.00000040,0.00021824,-0.00080935,-
0.00000033,0.00022949,0.00052337,0.00000014,-0.00064298,-0.00081303,-
0.00000014,-0.00007031,0.00009960,0.00000007,0.00000624,-0.00001938,-
0.00000004,0.00001431,0.00002862,0.00000002,-
0.00012407,0.00007239,0.00000003,0.00001335,-0.00000396,0.00000001,-
0.00006546,-0.00000814,0.00000002,0.00001564,-0.00002582,-
0.00000002,0.00004401,-0.00001462,0.00000001,0.00000299,-0.00001585,0.,-
0.00000323,0.00000885,0.,0.00011501,0.00011640,0.00000003,0.00004705,0.0000
9003,0.00000003,0.00006696,-0.00005730,-0.00000002,0.00002449,-
0.00002595,0.,0.00018209,0.00011587,-0.00000001,-
0.00006269,0.00000807,0.00000001,-
0.00008927,0.00000361,0.00000001,0.00001863,0.00000945,0.,-0.00002702,-
0.00004722,0.,0.00000374,0.00001063,0.,0.00021366,0.00082585,0.00000018,0.00
111496,0.00001715,-0.00000037,0.00138213,-0.00165638,-
0.00000042,0.00045956,0.01274853,0.00000563,-0.00412677,-
0.00109596,0.00000118,0.00074188,0.00107665,-
0.00000009,0.00007706,0.00025579,0.00000005,0.00137157,-0.00071559,-
0.00000054,-0.00001936,-0.00001468,-0.00000005,0.00098251,-0.00157165,-
0.00000056,-0.22707037,0.04516937,0.00006514,-
0.10381876,0.01433799,0.00004699,0.40395626,-0.00099429,-0.00196476,-
0.00000031,0.00035003,0.00348688,0.00000090,0.00058873,-0.00215493,-
0.00000091,-0.00048503,0.00139964,0.00000063,-0.00020547,-0.00085006,-
0.00000018,0.00084138,0.00123660,0.00000018,0.00013119,-0.00018707,-
0.00000013,0.00001347,-0.00001514,0.00000002,-0.00005478,-0.00003563,-
0.00000003,0.00023448,-0.00014903,-0.00000010,-
0.00000117,0.00001211,0.,0.00010999,0.00003756,-0.00000002,-
0.00002788,0.00005233,0.00000005,-
0.00010125,0.00002532,0.,0.00000815,0.00001458,0.00000002,0.00000478,-
0.00001369,0.,-0.00017137,-0.00017383,-0.00000003,-0.00003577,-0.00007028,-
0.00000002,-0.00010911,0.00006385,0.00000004,-
0.00001070,0.00002837,0.00000001,-0.00026839,-0.00015721,0.,0.00006648,-
0.00003051,-0.00000002,0.00011941,0.00000411,-0.00000001,-
0.00001356,0.00000017,0.,0.00003850,0.00006107,0.,-0.00000539,-
0.00001865,0.,0.00093474,-0.00155692,-0.00000067,0.00150140,-0.00191900,-
0.00000078,-0.00595611,-0.00468378,0.00000002,0.03938141,-0.03207981,-
0.00002274,0.00127945,-0.00464897,-
0.00000201,0.00041714,0.00043673,0.00000028,-0.00013400,-
0.00013180,0.,0.00019924,-0.00335272,-0.00000114,-0.00004213,-0.00064787,-
0.00000012,0.00003851,0.00047851,0.00000004,0.08712007,-0.17169499,-
0.00005544,-0.00539673,0.03641692,0.00000359,0.01218649,0.62625797,-
0.00000049,-
0.00000095,0.00003281,0.00000019,0.00000153,0.00007034,0.00000022,-
0.00000094,0.00000755,-0.00000023,0.00000065,-0.00000821,-0.00000013,-
0.00000038,-0.00000599,0.00000046,0.00000058,-0.00000620,0.00000006,-
0.00000008,0.00000308,0.,-
0.00000003,0.00000513,0.00000002,0.,0.00000322,0.00000012,-0.00000006,-

0.00000060,-0.00000002,-
0.00000003,0.00000154,0.00000007,0.00000003,0.00000318,-
0.00000002,0.00000002,-0.00000297,-0.00000004,0.00000002,-0.00000740,-
0.00000004,-0.00000001,-0.00000363,0.,0.00000002,-0.00000205,-0.00000011,-
0.00000005,0.00000177,0.,-0.00000003,0.00000877,-
0.00000006,0.,0.00000284,0.,0.00000005,0.00000212,-0.00000014,-0.00000007,-
0.00000096,0.,-0.00000001,0.00000552,0.00000005,0.,-0.00001380,0.00000003,-
0.00000002,-0.00000674,0.00000003,0.00000002,-0.00000325,0.,-0.00000003,-
0.00000233,0.00000024,-0.00000071,0.00008307,-0.00000005,-0.00000044,-
0.00025428,-0.00000158,-0.00000097,0.00156301,0.00001265,-
0.00001469,0.00861217,0.00000171,-0.00000109,0.00091420,-
0.00000032,0.00000007,-0.00077838,-0.00000006,-0.00000009,-0.00001117,-
0.00000023,-0.00000089,0.00044259,-0.00000004,-0.00000012,-0.00017213,-
0.00000025,0.00000061,0.00013882,0.00007429,-0.00004130,-
0.06111108,0.00004231,-0.00000268,0.02876062,-
0.00011718,0.00013442,0.03815367,0.00165431,-0.00119952,-0.00000064,-
0.00176281,0.00327871,0.00000100,0.00306242,-0.00444536,-0.00000197,-
0.00727084,0.00466795,0.00000319,0.00478017,-0.00103572,-0.00000156,-
0.00333978,0.00044426,0.00000099,0.00189045,-0.00388260,-
0.00000243,0.00412683,-0.00504103,-
0.00000239,0.01044076,0.01427705,0.00000751,-
0.17237338,0.04956866,0.00003947,-0.03493859,0.01392340,0.00001194,-
0.00292822,-0.00419768,-0.00000162,-
0.00021559,0.00061865,0.00000044,0.00096317,-0.00092430,-0.00000063,-
0.00028095,-0.00403383,-0.00000288,-
0.00010511,0.00096533,0.00000038,0.00051293,0.00005748,-
0.00000002,0.00004425,0.00026948,0.00000007,0.00011287,-0.00002040,-
0.00000007,-0.00000867,-0.00013942,-0.00000001,0.00057217,0.00029020,-
0.00000006,-0.00002651,0.00002926,0.00000003,-
0.00014970,0.00000936,0.00000002,0.00003230,-0.00000555,-0.00000002,-
0.00014150,-0.00017310,0.00000002,-0.00001066,0.00003397,0.,0.00015366,-
0.00053923,-0.00000011,0.00011303,-0.00017053,-
0.00000008,0.00013272,0.00010465,0.,0.00001423,-0.00071454,-0.00000023,-
0.00007773,0.00000118,0.00000003,-0.00026043,-0.00013660,0.,-
0.00007966,0.00015071,0.00000007,0.00003023,-0.00003368,-
0.00000002,0.00013444,0.00024594,0.00000003,-0.00001515,-0.00002976,-
0.00000001,0.00008264,0.00151916,0.00000041,-0.00032254,-0.00071961,-
0.00000011,0.00027790,-0.00055221,-0.00000022,0.60632986,-
0.00092059,0.00050564,0.00000023,0.00076677,-0.00138753,-0.00000035,-
0.00135419,0.00173756,0.00000078,0.00330362,-0.00253302,-0.00000112,-
0.00291979,0.00070376,0.00000103,0.00210277,-0.00010548,-0.00000059,-
0.00250100,-0.00236980,-0.00000058,-0.00245397,-0.00556962,-
0.00000777,0.02761281,-0.01945458,-0.00001444,0.03740815,-0.12516364,-
0.00003214,0.00530223,0.01917284,0.00000973,-0.00854655,0.00131696,-
0.00000239,-0.00134872,0.00081963,0.00000039,0.00102365,-0.00023372,-
0.00000041,-0.00035858,-0.00053474,0.00000201,-0.00433378,-
0.00228046,0.00000140,-0.00031858,-0.00003092,0.00000007,0.00001074,-
0.00016927,-0.00000007,-
0.00015705,0.00001624,0.00000005,0.00004903,0.00010974,0.00000002,-
0.00037246,-0.00016927,0.00000002,0.00000230,-0.00005511,-
0.00000003,0.00009853,-0.00001068,0.,-

0.00001604,0.00000241,0.,0.00010649,0.00013681,0.,-0.00002348,-0.00002655,0.,-
0.00006631,0.00024333,0.00000008,-0.00001965,0.00000252,-0.00000001,-
0.00005611,0.00000217,0.,0.00000965,0.00030851,0.00000010,0.00000905,-
0.00002737,-0.00000004,0.00010083,0.00009767,0.00000003,0.00002942,-
0.00006486,-0.00000003,-0.00001717,0.00001785,0.00000002,-0.00004277,-
0.00008675,0.,0.00001404,-0.00001408,0.,0.00010003,-0.00073701,-
0.00000027,0.00006448,0.00033234,0.00000009,-
0.00021903,0.00030730,0.00000016,-0.01069708,0.99411581,-
0.00000074,0.00000026,0.00015197,0.00000082,-0.00000115,-0.00009640,-
0.00000124,0.00000154,0.00008920,0.00000250,-0.00000138,-0.00120877,-
0.00000199,0.00000038,0.00009204,0.00000149,0.00000011,-0.00007536,-
0.00000189,-0.00000070,-0.00077923,-0.00000089,-
0.00000732,0.00787024,0.00001505,-0.00001251,0.00006057,0.00003134,-
0.00003248,-0.07780658,0.00000743,0.00000841,0.00201443,-0.00000396,-
0.00000279,0.00714705,-0.00000077,0.00000025,0.00033613,0.00000056,-
0.00000027,0.00035356,-0.00000052,0.00000231,-0.00395536,-
0.00000316,0.00000076,-0.00389003,-0.00000029,0.,-0.00001710,0.,-0.00000013,-
0.00000557,-0.00000010,0.,-0.00000490,0.00000001,0.00000013,-0.00000454,-
0.00000027,-0.00000012,0.00000059,0.,-
0.00000005,0.00000022,0.00000007,0.,0.00000259,0.00000001,0.,0.00000240,0.00
000009,0.00000008,0.00001132,0.,-0.00000003,0.00000508,-
0.00000008,0.00000019,-0.00001691,0.,0.00000004,-0.00000641,-0.00000005,-
0.00000005,-0.00000413,0.,0.00000027,0.00000049,-0.00000002,-
0.00000002,0.00000047,0.00000007,0.00000006,-0.00000663,0.00000005,-
0.00000005,0.00000281,0.,0.00000001,0.00000245,-0.00000004,-
0.00000008,0.00001214,0.00000003,0.,0.00000515,0.00000007,-
0.00000054,0.00000620,0.00000005,0.00000026,-0.00000775,-
0.00000015,0.00000023,-0.00000538,-0.00003878,0.00048956,0.20029332,-
0.00058179,0.00039511,0.00000024,0.00064805,-0.00109359,-0.00000039,-
0.00111986,0.00162270,0.00000071,0.00253430,-0.00148536,-0.00000104,-
0.00166107,0.00035021,0.00000055,0.00112773,-0.00015913,-0.00000033,-
0.00070989,0.00197181,0.00000118,-0.00031420,0.00251926,0.00000128,-
0.00652185,-0.00115762,0.00000030,0.00818945,0.03501561,0.00002243,-
0.00337012,0.00127585,0.00000128,0.00202917,0.00016048,-0.00000014,-
0.00000810,-0.00011576,-0.00000006,-
0.00046494,0.00031308,0.00000021,0.00015282,0.00092637,0.00000060,0.000239
88,0.00222965,0.00000114,-0.00018619,-0.00002212,0.00000002,-0.00001999,-
0.00009184,-0.00000003,-0.00001565,-0.00000186,0.00000002,-
0.00000488,0.00005270,0.00000001,-0.00020197,-
0.00010841,0.00000002,0.00001097,-0.00000304,0.,0.00004913,-0.00000178,0.,-
0.00001113,0.00000286,0.,0.00005623,0.00005797,-0.00000001,0.00000825,-
0.00001111,0.,-0.00005654,0.00019484,0.00000004,-
0.00002708,0.00004414,0.00000001,-0.00005030,-0.00003060,0.,-
0.00000144,0.00025184,0.00000008,0.00002257,-
0.00000392,0.,0.00008850,0.00005069,0.,0.00002513,-0.00005027,-0.00000002,-
0.00001132,0.00001159,0.,-0.00004286,-0.00008783,0.,0.00000622,0.00000610,0.,-
0.00001627,-0.00054758,-0.00000014,0.00010688,0.00025846,0.00000003,-
0.00010848,0.00020368,0.00000008,-
0.11798624,0.00221771,0.00000518,0.10983313,0.00080755,-0.00026559,-
0.00000021,-0.00074187,0.00114850,0.00000032,0.00126584,-0.00146521,-
0.00000062,-0.00256283,0.00172726,0.00000116,0.00210425,-0.00039965,-

0.00000071,-0.00150260,-0.00002568,0.00000039,0.00005523,-0.00076413,-
0.00000044,0.00190611,-0.00155473,-
0.00000003,0.00524131,0.00418230,0.00000036,0.01968379,-0.04930621,-
0.00004346,-0.00811228,-0.00405353,-0.00000167,0.00143256,-
0.00023825,0.00000005,0.00033899,-0.00010681,-0.00000012,0.00045040,-
0.00030105,-0.00000009,-
0.00028237,0.00098070,0.00000011,0.00243879,0.00105669,0.00000040,0.000242
85,0.00002762,-0.00000003,-0.00000253,0.00013070,0.00000005,0.00012355,-
0.00001776,-0.00000004,-0.00003544,-0.00008051,0.,0.00028557,0.00013306,-
0.00000002,-0.00000557,0.00003983,0.00000002,-
0.00007706,0.00000866,0.,0.00001222,-0.00000207,0.,-0.00007396,-
0.00010109,0.,0.00001714,0.00002043,0.,0.00005905,-0.00019833,-0.00000005,-
0.00000319,0.00000845,0.00000002,0.00005317,-0.00000008,0.,-0.00000771,-
0.00024890,-0.00000008,-0.00000488,0.00001936,0.00000002,-0.00007981,-
0.00007443,-0.00000002,-0.00002081,0.00004885,0.00000002,0.00001386,-
0.00001429,-0.00000002,0.00003213,0.00008515,0.,-0.00001121,0.00001187,0.,-
0.00007438,0.00059191,0.00000020,-0.00005476,-0.00026795,-
0.00000007,0.00017305,-0.00024682,-0.00000012,0.00248120,-0.72657658,-
0.00040187,0.02537120,0.84639118,0.00000054,-0.00000020,-0.00002977,-
0.00000055,0.00000083,0.00003293,0.00000091,-0.00000106,-0.00000527,-
0.00000175,0.00000112,0.00013942,0.00000138,-0.00000028,0.00002642,-
0.00000102,-0.00000003,-0.00000582,0.00000014,-
0.00000067,0.00005790,0.00000098,-0.00000032,-
0.00156079,0.00000410,0.00000103,0.00264535,0.00001309,-
0.00004501,0.01901177,-0.00000461,-
0.00000251,0.00013319,0.00000058,0.00000011,-0.00056290,0.00000022,-
0.00000008,0.00003919,0.00000029,-0.00000005,-0.00026624,-
0.00000012,0.00000016,0.00064217,0.00000142,0.00000067,-
0.00035062,0.00000022,0.00000001,0.00000196,0.,0.00000009,0.00000296,0.0000
0007,0.,0.00000765,0.,-0.00000010,0.00000561,0.00000019,0.00000009,-
0.00000059,0.,0.00000004,0.00000167,-0.00000005,0.,-0.00000309,-
0.00000001,0.,-0.00000216,-0.00000006,-0.00000005,-0.00001306,0.,0.00000003,-
0.00000649,0.00000006,-0.00000014,0.00000471,0.,-
0.00000002,0.00000531,0.00000005,0.00000003,0.00000328,-0.00000001,-
0.00000018,-0.00000041,0.00000001,0.00000001,0.00000111,-0.00000004,-
0.00000004,0.00000368,-0.00000003,0.00000003,-0.00000294,0.,0.,-
0.00000193,0.00000002,0.00000006,-0.00000844,-0.00000002,0.,-0.00000363,-
0.00000006,0.00000038,-0.00000549,-0.00000003,-
0.00000018,0.00000628,0.00000011,-0.00000017,0.00000474,0.00000534,-
0.00040148,-0.07412535,0.00000936,0.00050001,0.03200582,-0.00013848,-
0.00026124,-0.00000006,0.00006775,0.00042802,0.00000011,0.00006916,-
0.00024747,-0.00000008,-0.00003144,0.00015388,0.00000008,-0.00003764,-
0.00010292,-0.00000005,0.00012739,0.00017394,0.00000005,0.00001458,-
0.00002127,-0.00000002,-0.00000166,0.00000188,0.,-0.00000236,-
0.00000545,0.,0.00002563,-0.00001480,0.,-
0.00000294,0.00000130,0.,0.00001408,0.00000186,0.,-0.00000317,0.00000531,0.,-
0.00000789,0.00000177,0.,-0.00000069,0.00000317,0.,0.00000066,-0.00000181,0.,-
0.00002619,-0.00002558,-0.00000001,-0.00000732,-0.00000962,0.,-
0.00001381,0.00001058,0.,-0.00000329,0.00000429,0.,-0.00004043,-
0.00002357,0.,0.00001358,-0.00000497,0.,0.00001967,0.00000156,0.,-0.00000238,-
0.00000105,0.,0.00000532,0.00000867,0.,-0.00000043,-0.00000235,0.,-

0.00014151,-0.00034219,-0.00000005,-
0.00043832,0.00023441,0.00000003,0.00070405,0.00126476,0.00000030,-
0.00632435,0.00174235,0.00000418,-0.00063779,-0.00068971,-
0.00000016,0.00032781,-0.00012202,-0.00000011,-0.00001118,-
0.00001382,0.,0.00070457,0.00010669,-0.00000020,0.00003043,0.00011219,0.,-
0.00009874,0.00012610,0.00000012,0.01208091,0.05390491,0.00001032,0.000611
74,-0.00802894,-0.00000418,-0.07553728,-0.13154905,-0.00001146,-
0.00005667,0.00004739,0.00000003,0.00002233,-0.00003714,-
0.00000002,0.06868888,0.00014132,0.00030336,0.00000005,-0.00003992,-
0.00053081,-0.00000020,-0.00009697,0.00030752,0.00000022,0.00007974,-
0.00021452,-0.00000017,0.00001407,0.00013001,0.00000004,-0.00012577,-
0.00020482,-0.00000004,-0.00002295,0.00002900,0.00000004,-
0.00000076,0.00000354,0.,0.00000785,0.00000462,0.,-
0.00004034,0.00002537,0.00000001,0.00000154,-0.00000242,0.,-0.00001791,-
0.00000530,0.,0.00000464,-0.00000909,0.,0.00001435,-0.00000180,0.,-
0.00000111,-0.00000333,-0.00000001,-
0.00000043,0.00000227,0.,0.00002859,0.00002690,0.00000002,0.00000150,0.0000
0390,0.,0.00001794,-0.00000756,0.,-0.00000170,-
0.00000345,0.,0.00004493,0.00002471,0.,-0.00000801,0.00000647,0.,-0.00001715,-
0.00000092,0.,0.00000015,-0.00000211,0.,-0.00000614,-
0.00000899,0.,0.00000044,0.00000329,0.,-
0.00001015,0.00028956,0.00000010,0.00058676,0.00003932,-0.00000004,-
0.00058025,-0.00096714,-0.00000025,0.00417075,0.00113695,-
0.00000225,0.00026507,0.00083919,0.00000033,-
0.00013943,0.00005660,0.,0.00001239,-0.00000393,-0.00000001,-0.00013285,-
0.00000645,0.00000009,-0.00004844,-0.00008200,0.00000001,0.00015765,-
0.00010136,-0.00000011,0.00170012,-0.03867528,-0.00001098,-
0.00808541,0.00478110,0.00000539,-0.08103580,-0.44361189,-
0.00007242,0.00009665,-0.00005532,-0.00000004,-
0.00003629,0.00004444,0.00000003,0.08316101,0.47645160,0.00000008,0.000000
18,-0.00001985,-0.00000006,-0.00000026,-0.00000447,-0.00000005,0.00000017,-
0.00002519,0.00000005,-0.00000012,0.00003435,0.,0.00000007,-0.00001886,-
0.00000009,-0.00000012,0.00001746,0.,0.,-0.00000023,0.,0.00000001,-
0.00000405,-0.00000002,-0.00000001,-0.00000255,-
0.00000003,0.,0.00000037,0.00000002,0.00000003,-0.00000144,-0.00000002,-
0.00000001,-
0.00000205,0.,0.,0.00000225,0.,0.,0.00000552,0.00000002,0.,0.00000315,0.,-
0.00000001,0.00000157,0.00000003,0.,-0.00000108,-0.00000001,0.,-
0.00000539,0.00000002,0.00000001,-0.00000208,0.,-0.00000002,-
0.00000161,0.00000003,0.,0.00000055,0.00000001,0.,-
0.00000346,0.,0.,0.00000811,-0.00000002,0.,0.00000434,-
0.00000001,0.,0.00000231,0.,0.00000001,0.00000173,0.,0.00000017,-
0.00000590,0.00000022,0.00000009,-0.00042688,-0.00000019,-
0.00000090,0.00045835,0.00000482,-
0.00000170,0.00539267,0.00000011,0.00000056,-0.00045723,-0.00000004,-
0.00000005,0.00015826,0.00000001,0.,0.00002948,-0.00000027,0.00000005,-
0.00023496,-0.00000004,-0.00000004,-0.00005063,0.00000012,-
0.00000011,0.00017588,-0.00000180,-0.00002793,0.00459541,-
0.00000432,0.00000529,-0.00657053,0.00000023,-0.00005558,-
0.01642916,0.00000004,-0.00000002,0.00000457,-0.00000001,0.00000001,-
0.00000397,0.00000118,0.00008020,0.01337885,0.00343391,0.00369439,0.000000

48,-0.00201847,-0.00286647,-0.00000045,0.00171171,0.00088832,-0.00000012,-
0.00323267,0.00008202,0.00000091,0.00457922,0.00047702,-0.00000092,-
0.00716729,-0.00336063,0.00000017,0.00048333,-0.00012356,-
0.00000017,0.00002378,-0.00026507,-0.00000010,-0.00004454,-0.00002849,-
0.00000002,0.00054051,-0.00031311,-
0.00000019,0.00001337,0.00012485,0.00000005,0.00011248,0.00001959,-
0.00000009,-0.00011948,0.00018927,0.00000017,-
0.00014644,0.00000931,0.,0.00004298,0.00000987,-0.00000002,-0.00000749,-
0.00001829,0.,0.00146028,0.00274960,0.00000030,0.00576389,0.00351528,0.0000
0120,0.00011550,0.00478373,0.00000244,-0.03014857,-0.00419639,0.00000435,-
0.17452495,-0.03612067,0.00000845,-0.00267830,-0.02392235,-
0.00000513,0.00087640,0.00071821,0.00000007,-
0.00028605,0.00444861,0.00000049,0.00002293,-0.00048138,-
0.00000003,0.00141939,-0.00063497,-
0.00000105,0.00021910,0.00048900,0.00000006,-
0.00023762,0.00015295,0.00000005,-
0.00006194,0.00001794,0.00000006,0.00005161,0.00067270,0.00000015,0.000120
25,-0.00012280,-0.00000005,0.00011685,0.00015481,0.00000003,0.00009556,-
0.00024386,-0.00000010,-0.00002727,0.00003316,0.00000002,-0.00009158,-
0.00013210,0.,0.00001904,0.00002344,0.,0.00016886,-0.00146869,-
0.00000042,0.00017048,0.00065485,0.00000011,-
0.00041016,0.00059378,0.00000027,-
0.00123363,0.00065860,0.00000053,0.00045242,-0.00051118,-
0.00000038,0.00009444,-0.00010208,-
0.00000005,0.90663364,0.00181072,0.00204658,0.00000018,-0.00120813,-
0.00142781,-0.00000012,0.00116658,0.00044921,-0.00000021,-
0.00242940,0.00008269,0.00000074,0.00356798,0.00021968,-0.00000081,-
0.00472273,-0.00263337,0.00000055,0.00034914,-0.00008612,-0.00000015,-
0.00002764,-0.00018632,-0.00000007,-0.00002025,-0.00006083,-
0.00000004,0.00041083,-0.00021719,-0.00000017,-
0.00003615,0.00012106,0.00000010,0.00017101,0.00000316,-0.00000006,-
0.00009945,0.00016705,0.00000012,-
0.00011197,0.00000210,0.,0.00002623,0.00000511,0.,0.00002541,-0.00002175,-
0.00000002,0.00413226,-0.00194334,-0.00000108,-0.00084382,-0.00738690,-
0.00000332,0.00737735,-0.00153576,-
0.00000361,0.00910066,0.02107607,0.00000346,-0.04826663,-0.12270512,-
0.00000224,-0.03255439,-0.01310483,0.00000206,-0.00124995,-
0.00027290,0.00000007,-0.00086698,-
0.00219519,0.00000051,0.00146834,0.00070652,-0.00000014,0.00306975,-
0.00227596,-0.00000017,0.00011303,0.00026345,0.00000002,-
0.00010691,0.00011236,0.00000007,-0.00000891,-0.00002000,-
0.00000002,0.00001033,0.00035088,0.00000011,0.00006542,-0.00001805,-
0.00000003,0.00001775,0.00001195,0.00000002,0.00003575,-0.00010086,-
0.00000005,-0.00002442,-0.00001005,0.,-0.00004252,-
0.00006831,0.,0.00001304,0.00001541,0.,-0.00005055,-0.00082385,-
0.00000023,0.00018251,0.00040524,0.00000007,-
0.00014015,0.00029789,0.00000013,-
0.00075950,0.00057849,0.00000039,0.00027228,-0.00044232,-
0.00000026,0.00003341,-0.00006167,-0.00000003,-0.16277229,0.69393512,-
0.00000018,-0.00000020,0.00009202,0.00000011,0.00000020,-0.00007507,-
0.00000003,-0.00000004,0.00015179,-0.00000009,0.,-0.00009605,0.00000013,-

0.00000005,0.00008912,-0.00000008,0.00000029,-0.00120882,0.,0.,-
0.00001708,0.,-0.00000001,-
0.00000661,0.00000002,0.,0.00000041,0.00000002,0.,0.00000057,-0.00000003,-
0.00000001,-0.00000412,0.00000005,0.,-
0.00000645,0.,0.,0.00001218,0.,0.,0.00000283,-0.00000003,-
0.00000002,0.00000249,0.,0.,0.00000516,0.00000055,-0.00000078,-0.00077970,0.,-
0.00000421,0.00714702,0.00000318,-
0.00000319,0.00786978,0.00000740,0.00000596,0.00007243,0.00000539,-
0.00000471,-0.07779797,-0.00000711,0.00000055,0.00200856,-0.00000040,-
0.00000027,0.00033593,-0.00000084,-0.00000036,-
0.00382684,0.00000043,0.00000018,0.00035387,-0.00000022,0.00000054,-
0.00396614,-0.00000004,-0.00000009,-0.00001726,0.00000004,0.00000001,-
0.00000557,0.00000004,-0.00000005,0.00000018,0.,-0.00000003,0.00000066,-
0.00000006,0.,-0.00000455,-0.00000004,-0.00000004,-
0.00000491,0.00000003,0.00000003,0.00001134,0.,0.,0.00000511,0.,0.,0.00000261
,0.00000002,0.,0.00000244,0.,0.00000013,0.00000616,-0.00000002,-0.00000003,-
0.00000544,0.00000004,-0.00000003,-
0.00000802,0.00000005,0.00000008,0.00000617,-0.00000002,-0.00000005,-
0.00000772,-0.00000001,0.,0.00000572,-0.00016051,0.00014753,0.20031556,-
0.00177697,-0.00203613,-0.00000026,0.00098310,0.00158670,0.00000028,-
0.00086590,-0.00064402,-0.00000001,0.00181817,0.00006621,-0.00000044,-
0.00262907,-0.00039359,0.00000050,0.00368079,0.00189973,-0.00000013,-
0.00027307,0.00006568,0.00000008,-
0.00002111,0.00014319,0.00000005,0.00002405,0.00000422,0.,-
0.00029296,0.00017577,0.00000013,-0.00002220,-0.00006937,-0.00000003,-
0.00001816,-0.00000178,0.00000005,0.00007728,-0.00010146,-
0.00000010,0.00007365,-0.00000728,0.,-0.00002453,-
0.00000622,0.00000001,0.00001266,0.00000745,0.,-
0.00162735,0.00047529,0.00000041,-0.00035763,-0.00005504,-0.00000009,-
0.00316075,-0.00026205,0.00000025,-0.00026526,-0.00885014,-0.00000164,-
0.05860328,0.01892174,0.00001787,-0.00093687,0.00669140,0.00000184,-
0.00017911,-0.00024106,-0.00000002,-0.00119016,-0.00163386,-0.00000025,-
0.00067348,-0.00033001,-0.00000003,0.00049445,0.00008811,0.00000004,-
0.00012192,-0.00028029,-0.00000006,0.00013403,-0.00008688,-
0.00000003,0.00003646,-0.00000908,-0.00000003,-0.00002737,-0.00038623,-
0.00000008,-0.00006943,0.00007238,0.00000003,-0.00006968,-0.00009239,-
0.00000001,-0.00005458,0.00013814,0.00000006,0.00001477,-
0.00002096,0.,0.00005265,0.00007506,0.,-0.00001077,-0.00001230,0.,-
0.00011501,0.00083899,0.00000024,-0.00008595,-0.00036954,-
0.00000006,0.00024599,-0.00034472,-0.00000016,0.00070942,-0.00034408,-
0.00000028,-0.00026253,0.00026778,0.00000020,-
0.00005685,0.00005859,0.00000003,-
0.57684581,0.26235030,0.00014919,0.64069749,-0.00028274,-0.00028263,-
0.00000001,0.00024319,0.00011965,-0.00000003,-
0.00023116,0.00001826,0.00000008,0.00041778,-0.00002176,-0.00000013,-
0.00075059,0.00004456,0.00000018,0.00082171,0.00057884,-0.00000008,-
0.00006990,0.00001793,0.00000004,0.00001270,0.00003502,0.,0.00000299,0.0000
1787,0.00000001,-0.00008363,0.00004237,0.00000004,0.00001455,-0.00002791,-
0.00000003,-0.00004925,-0.00000052,0.,0.00001847,-0.00003499,-
0.00000002,0.00002220,0.00000031,0.,-0.00000390,-0.00000103,0.,-
0.00000983,0.00000550,0.,-

0.00143949,0.00015303,0.00000029,0.00122096,0.00215002,0.00000044,-
0.00087302,0.00129410,0.00000086,-0.00245849,-0.00207780,-
0.00000073,0.00352706,0.01750012,-0.00000097,-0.00264732,-0.00647037,-
0.00000121,0.00021455,0.00006407,-0.00000003,-
0.00141244,0.00252411,0.00000101,-0.00019339,-0.00009342,0.00000007,-
0.00112031,0.00063989,0.00000020,-0.00001521,-0.00003694,0.,0.00000287,-
0.00002303,-0.00000002,-0.00001172,0.00001446,0.00000002,0.00000758,-
0.00004142,-0.00000002,-0.00001539,-
0.00001637,0.,0.00003322,0.00003656,0.,0.00000613,0.00001001,0.,0.00000730,0.
00001393,0.,-0.00000032,0.00000483,0.,-0.00000300,-
0.00000233,0.,0.00005538,0.00012505,0.00000003,-0.00005668,-0.00007517,-
0.00000001,-0.00000860,-0.00003450,-0.00000001,0.00012862,-0.00013350,-
0.00000007,-
0.00004523,0.00010137,0.00000005,0.00000074,0.00001162,0.,0.26261238,-
0.26811904,-0.00009172,-
0.33180656,0.31587381,0.00000024,0.00000028,0.00002658,-0.00000013,-
0.00000026,-0.00000615,0.00000010,0.00000012,-0.00002956,-0.00000021,-
0.00000003,0.00003252,0.00000027,0.00000009,-0.00000507,-0.00000045,-
0.00000025,0.00013925,0.00000005,-0.00000002,0.00000495,-0.00000001,-
0.00000001,0.00000366,-0.00000002,-0.00000003,0.00000119,0.00000003,-
0.00000002,-0.00000053,0.00000002,0.00000003,0.00000330,-
0.00000003,0.,0.00000533,0.,0.00000001,-0.00000847,0.,0.,-
0.00000297,0.00000003,0.00000001,-0.00000198,0.,-0.00000001,-0.00000366,0.,-
0.00000005,0.00005792,0.00000025,0.00000067,-
0.00056257,0.00000005,0.00000077,-0.00156270,-
0.00000006,0.00000031,0.00264395,0.00001429,-0.00000358,0.01901802,-
0.00000045,-0.00000274,0.00012956,0.00000009,0.00000005,0.00003926,-
0.00000021,0.00000100,-0.00036554,0.00000002,0.00000010,-0.00026603,-
0.00000025,0.,0.00064228,0.00000004,0.00000007,0.00000218,-
0.00000003,0.,0.00000296,-
0.00000002,0.00000004,0.00000173,0.00000001,0.00000006,-
0.00000069,0.00000004,-
0.00000002,0.00000565,0.00000003,0.00000004,0.00000767,-0.00000002,-
0.00000003,-0.00001306,0.,0.00000001,-0.00000652,0.,-0.00000001,-0.00000312,-
0.00000002,0.,-0.00000222,0.,-0.00000016,-
0.00000769,0.00000002,0.00000006,0.00000622,-
0.00000005,0.00000006,0.00001014,-0.00000011,0.,-0.00000543,0.00000004,-
0.00000001,0.00000623,0.,0.,-0.00000713,0.00014905,-0.00009162,-0.07422543,-
0.00018423,0.00012532,0.03212932,-0.00092379,-0.00096606,-
0.00000013,0.00054349,0.00067200,0.00000012,-0.00044215,-
0.00018216,0.00000001,0.00077808,-0.00002061,-0.00000020,-0.00115957,-
0.00009446,0.00000028,0.00181287,0.00074958,-0.00000014,-
0.00012516,0.00003249,0.00000003,-
0.00000730,0.00006584,0.00000003,0.00001156,0.00000340,0.00000001,-
0.00013836,0.00008331,0.00000004,-0.00000618,-0.00003401,0.,-0.00001747,-
0.00000138,0.00000003,0.00003410,-0.00004811,-0.00000005,0.00003519,-
0.00000246,0.,-0.00001085,-0.00000302,0.,0.00000300,0.00000451,0.,-
0.00035271,-0.00126024,-0.00000023,-0.00050201,-0.00243817,-
0.00000058,0.00116310,-0.00017613,-0.00000038,-0.00443958,-
0.00148084,0.00000057,-0.00131838,-0.04056946,-0.00000811,-
0.00646485,0.00141531,0.00000177,-0.00051523,-0.00024454,0.00000001,-

0.00005689,-0.00063964,-0.00000013,-0.00002682,0.00007419,0.00000002,-
0.00239124,-0.00248470,-0.00000016,-0.00005799,-0.00012394,-
0.00000001,0.00005597,-0.00003978,-0.00000004,0.00001037,-0.00000078,0.,-
0.00000991,-0.00016819,-0.00000004,-0.00003457,0.00002662,0.,-0.00001249,-
0.00002443,-0.00000001,-0.00002017,0.00006415,0.00000003,0.00000830,-
0.00000505,0.,0.00001997,0.00003030,0.,-0.00000520,-0.00000586,0.,-
0.00003345,0.00037188,0.00000009,-0.00004930,-0.00016932,-
0.00000001,0.00009756,-0.00014904,-0.00000007,0.00031710,-0.00017342,-
0.00000015,-0.00011726,0.00013467,0.00000011,-
0.00002276,0.00002700,0.00000002,-0.12811658,-
0.02996174,0.00000430,0.00515752,0.06839737,0.00002071,0.58109817,-
0.00154192,-0.00149865,-0.00000014,0.00099759,0.00107423,0.00000012,-
0.00091169,-0.00017808,0.00000014,0.00163471,-0.00018563,-0.00000050,-
0.00238735,-0.00007841,0.00000056,0.00324689,0.00158108,-0.00000025,-
0.00025496,0.00006656,0.00000009,0.00001442,0.00013821,0.00000005,0.000018
18,0.00004147,0.00000003,-0.00030097,0.00016324,0.00000012,0.00002473,-
0.00008693,-0.00000007,-0.00012764,-0.00000576,0.00000005,0.00006785,-
0.00011923,-0.00000008,0.00008316,-0.00000128,0.,-0.00001897,-0.00000408,0.,-
0.00001850,0.00001579,0.00000001,-0.00115415,-0.00098750,-0.00000005,-
0.00094666,-0.00030225,0.00000011,-
0.00083854,0.00001873,0.00000037,0.00096735,-0.00435324,-0.00000147,-
0.01373214,-0.03011543,-0.00000719,-0.00287358,0.00316765,0.00000090,-
0.00027206,-0.00015241,0.00000006,0.00096367,0.00151510,0.00000019,-
0.00031642,-0.00002892,0.00000004,-0.00173289,0.00051537,0.00000041,-
0.00009249,-0.00019941,-0.00000001,0.00007839,-0.00008381,-
0.00000005,0.00000223,0.00001516,0.,-0.00000655,-0.00026311,-0.00000007,-
0.00005678,0.00001323,0.00000001,0.00000855,0.00000331,-0.00000002,-
0.00002252,0.00008953,0.00000005,0.00001894,0.00000919,0.,0.00002828,0.0000
4812,0.,-0.00001002,-0.00001173,0.,0.00003937,0.00061919,0.00000016,-
0.00013900,-0.00030617,-0.00000004,0.00010377,-0.00022376,-
0.00000010,0.00056030,-0.00042956,-0.00000030,-
0.00020121,0.00032890,0.00000021,-
0.00002503,0.00004672,0.00000003,0.01185393,-0.27035887,-
0.00005305,0.04867050,-0.07256750,-0.00003301,0.09066929,0.44881885,-
0.00000023,-0.00000021,0.00000733,0.00000018,0.00000003,-0.00000819,-
0.00000016,0.,-0.00000598,0.00000026,-0.00000005,-0.00000635,-
0.00000039,0.00000002,0.00003304,0.00000052,0.00000022,0.00007055,-
0.00000006,0.00000005,0.00000177,0.,0.00000002,0.00000286,0.00000006,0.0000
0005,0.00000211,-0.00000003,0.00000003,-0.00000091,-0.00000002,-
0.00000007,0.00000555,-0.00000003,0.,0.00000873,0.,-0.00000002,-
0.00001380,0.00000001,0.,-0.00000325,-0.00000004,-0.00000001,-
0.00000233,0.,0.00000003,-0.00000675,-0.00000021,0.,0.00008156,-
0.00000015,0.00000042,-0.00025394,-0.00000057,0.00000020,-
0.00078026,0.00000119,-0.00000101,0.00089131,-0.00000149,-
0.00000235,0.00864767,0.00000063,0.00000015,0.00156410,-
0.00000001,0.00000004,-0.00017210,0.00000030,0.00000043,0.00014023,-
0.00000007,-0.00000003,-
0.00001150,0.00000005,0.00000044,0.00041953,0.00000002,0.00000007,0.000003
09,0.00000002,0.,0.00000324,-0.00000007,0.,0.00000149,0.00000003,-
0.00000002,-0.00000066,0.,0.,0.00000328,-0.00000002,0.00000002,0.00000509,-
0.00000001,0.00000001,-0.00000743,0.00000003,-0.00000002,-

0.00000364,0.00000002,0.,-0.00000298,0.,0.,-0.00000203,0.,0.00000005,-
0.00000541,-0.00000002,-0.00000004,0.00000478,0.00000002,-
0.00000002,0.00000682,0.00000010,-0.00000012,-0.00000793,-
0.00000003,0.00000008,0.00001001,0.,0.00000002,-0.00000493,0.00001450,-
0.00004614,-0.06104248,0.00001582,-0.00003632,0.02875468,-
0.00007063,0.00008477,0.03812401,0.00009858,0.00011342,0.00000002,-
0.00006757,-0.00010037,-0.00000005,0.00004945,0.00003606,0.00000003,-
0.00010055,-0.00000589,0.,0.00014038,0.00002020,-0.00000003,-0.00021249,-
0.00012209,0.,0.00001486,-0.00000477,0.,0.00000307,-0.00000783,0.,-
0.00000229,0.00000221,0.,0.00001770,-0.00001141,-
0.00000001,0.00000254,0.00000171,0.,-0.00000244,0.00000126,0.,-
0.00000249,0.00000430,0.,-0.00000432,0.00000043,0.,0.00000160,0.00000073,0.,-
0.00000255,-0.00000027,0.,0.00002955,0.00010923,0.00000002,0.00027543,-
0.00017462,-0.00000015,0.00001181,-
0.00004402,0.00000002,0.00028662,0.00026879,-
0.00000006,0.00183828,0.00054557,0.00000071,-
0.00025189,0.00002799,0.00000013,-0.00002621,0.00001558,0.,0.00002287,-
0.00008747,0.,-0.00000661,-0.00000952,0.,0.00015473,-0.00018284,-
0.00000011,0.00000634,0.00001831,0.00000002,-
0.00000735,0.00000530,0.00000002,-0.00000156,-
0.00000053,0.,0.00000154,0.00002624,0.,0.00000406,-
0.00000417,0.,0.00000279,0.00000339,0.,0.00000362,-0.00000768,0.,-
0.00000167,0.00000044,0.,-0.00000342,-
0.00000466,0.,0.00000091,0.00000053,0.,0.00000623,-
0.00006015,0.,0.00000732,0.00002761,0.,-0.00001620,0.00002510,0.00000002,-
0.00005225,0.00001712,0.00000002,0.00002011,-0.00001396,-
0.00000002,0.00000386,-0.00000511,0.,-0.00185916,-0.00975120,-0.00000122,-
0.00327009,0.00586168,0.00000084,-0.44363599,-
0.13186066,0.00004054,0.44649913,0.00026454,0.00027775,0.00000003,-
0.00017480,-0.00017832,-0.00000004,0.00015335,0.00004267,0.,-
0.00030585,0.00002363,0.00000006,0.00042258,0.00002387,-0.00000008,-
0.00058994,-0.00024989,0.00000004,0.00004934,-0.00001424,0.,-0.00000080,-
0.00002451,0.,-0.00000367,-0.00000451,0.,0.00005713,-0.00003340,-0.00000003,-
0.00000140,0.00001746,0.00000002,0.00001252,-0.00000103,0.,-
0.00001444,0.00002127,0.00000001,-
0.00001423,0.00000062,0.,0.00000348,0.00000132,0.,0.00000094,-
0.00000192,0.,0.00044112,0.00011983,-0.00000004,0.00017807,-0.00067488,-
0.00000008,-0.00006070,0.00037337,0.00000006,0.00122378,-0.00008483,-
0.00000013,0.00297553,-0.00701917,-0.00000356,-0.00181756,-
0.00001074,0.00000021,-0.00014500,-0.00002529,0.00000003,-0.00005456,-
0.00022336,-0.00000009,0.00001665,-0.00000871,-0.00000001,-
0.00042198,0.00054265,0.00000027,0.00001649,0.00003742,0.00000001,-
0.00001426,0.00001613,0.00000002,-0.00000044,-
0.00000399,0.,0.00000090,0.00004939,0.,0.00000912,-0.00000165,0.,0.00000066,-
0.00000079,0.,0.00000489,-0.00001336,0.,-0.00000375,-0.00000226,0.,-
0.00000542,-0.00000896,0.,0.00000194,0.00000202,0.,-0.00001102,-0.00011878,-
0.00000003,0.00002845,0.00005970,0.,-0.00001779,0.00004238,0.00000003,-
0.00011819,0.00008516,0.00000006,0.00004373,-0.00006542,-
0.00000004,0.00000429,-0.00000950,0.,-0.06194511,-
0.02473112,0.00000342,0.00579766,0.00864682,0.00000275,-0.08135498,-
0.07577998,-0.00000071,0.13532330,0.09891251,0.00000006,0.00000006,-

0.00002512,-0.00000005,0.00000002,0.00003443,0.00000004,0.,-0.00001893,-
0.00000006,0.00000002,0.00001764,0.00000008,0.,-0.00002003,-0.00000013,-
0.00000003,-0.00000457,0.00000003,-0.00000002,-0.00000115,0.,0.,-0.00000209,-
0.00000003,-0.00000002,-0.00000162,0.,-
0.00000001,0.00000056,0.00000002,0.00000004,-0.00000349,0.,0.,-
0.00000537,0.,0.,0.00000811,0.,0.,0.00000232,0.00000002,0.,0.00000175,0.,-
0.00000001,0.00000436,0.00000011,0.,-0.00000661,-0.00000009,-0.00000003,-
0.00042619,0.00000002,0.00000008,0.00015873,0.00000017,0.00000003,-
0.00045735,0.00000138,-0.00000309,0.00539123,-0.00000029,-
0.00000012,0.00045881,-0.00000004,0.,-0.00005072,0.,-
0.00000007,0.00017705,0.,0.,0.00002949,-0.00000018,0.00000024,-0.00023059,0.,-
0.00000003,-0.00000030,-0.00000001,0.,-0.00000208,0.00000003,0.,-0.00000143,-
0.00000002,0.,0.00000044,0.,0.,-0.00000260,0.00000002,0.,-
0.00000403,0.,0.,0.00000554,-
0.00000001,0.,0.00000318,0.,0.,0.00000226,0.,0.,0.00000158,0.,-
0.00000001,0.00000456,0.,0.,-0.00000397,0.,0.,-0.00000586,-
0.00000003,0.00000004,0.00000565,0.,-0.00000003,-0.00000704,0.,0.,0.00000426,-
0.00001409,-0.00000548,0.00460698,0.00000088,0.00000272,-
0.00658886,0.00005288,0.00000787,-0.01645093,-0.00004071,-
0.00000212,0.01340270,-0.00071838,0.00075311,0.00000029,0.00075442,-
0.00164954,-0.00000048,-0.00142363,0.00223622,0.00000099,0.00337244,-
0.00234997,-0.00000160,-0.00244356,0.00066612,0.00000091,0.00166498,-
0.00035025,-0.00000049,-0.00187932,0.00027121,0.00000034,-
0.00013070,0.00008873,-0.00000002,-0.00464720,-0.00101685,-0.00000018,-
0.04647438,-0.01226253,-0.00000372,0.00013933,0.00667881,0.00000401,-
0.00182405,0.00019476,0.00000050,-0.00046659,0.00030025,0.00000017,-
0.00013341,0.00025511,0.00000017,-
0.00196015,0.00187066,0.00000132,0.00126267,-0.00020493,-0.00000034,-
0.00025782,-0.00002326,0.,-0.00003209,-0.00012910,-0.00000003,-0.00000750,-
0.00000568,0.00000002,-0.00001150,0.00006987,0.,-0.00027580,-
0.00014700,0.00000004,0.00001449,0.00000037,-0.00000002,0.00006641,-
0.00000151,0.,-0.00001706,0.00000322,0.00000002,0.00007996,0.00008052,-
0.00000002,0.00001502,-0.00001511,0.,-0.00007750,0.00028340,0.00000005,-
0.00006462,0.00010064,0.00000004,-0.00007128,-0.00006168,0.,-
0.00000637,0.00037719,0.00000012,0.00004380,0.00000023,-
0.00000002,0.00013667,0.00006911,-0.00000001,0.00004258,-0.00007893,-
0.00000003,-0.00001505,0.00001629,0.00000002,-0.00007221,-0.00012947,-
0.00000001,0.00000638,0.00002041,0.00000001,-0.00007618,-0.00078795,-
0.00000021,0.00018901,0.00037808,0.00000007,-
0.00012377,0.00027712,0.00000009,-0.24280090,-0.07792757,-0.00003549,-
0.00243493,-0.05305361,-0.00003063,0.00002458,-0.00004863,-
0.00000001,0.00063415,0.00034645,-0.00000006,-0.00037552,-
0.00004953,0.00000008,-0.00016311,-0.00025432,-
0.00000005,0.00003031,0.00005568,0.00000002,0.56052315,0.00002355,0.000097
61,0.00000003,0.00000660,-0.00016144,-0.00000003,-
0.00005816,0.00018547,0.00000004,0.00014935,0.00002398,0.,0.00008998,0.0000
1867,-0.00000004,-0.00002450,-
0.00004673,0.,0.00037876,0.00053198,0.00000021,-
0.00057113,0.00131356,0.00000125,0.00134961,-0.00417464,-
0.00000295,0.01463191,0.01502203,0.00000279,0.00239225,-0.00342419,-
0.00000318,0.00167651,0.00101738,0.00000051,0.00027261,-0.00020087,-

0.00000004,-0.00013590,0.00007802,0.00000006,0.00263246,0.00025271,-
0.00000014,0.00140149,0.00019216,-0.00000014,0.00000830,0.00000030,-
0.00000002,-0.00001103,0.00000417,0.00000002,0.00002726,-0.00000154,0.,-
0.00001367,-0.00000943,0.,0.00001492,0.00000256,0.,0.00000331,0.00001073,0.,-
0.00000344,0.00000163,0.,-0.00000088,0.00000001,0.,-0.00000621,-
0.00001076,0.,0.00000892,0.00000255,0.,-0.00000528,0.00001809,0.,-
0.00002551,0.00004107,0.00000003,-0.00000280,-0.00002257,-0.00000001,-
0.00000704,0.00003155,0.00000001,0.00001437,0.00000942,0.,0.00001700,-
0.00000596,0.,0.00000716,-0.00000876,0.,0.00000022,0.00000052,0.,-0.00001358,-
0.00001258,0.,-0.00000233,0.00001234,0.,-0.00005271,-
0.00003623,0.00000001,0.00004156,0.00002289,-0.00000001,0.00002409,-
0.00000210,-0.00000001,-0.03609556,-0.15585575,-0.00005630,-0.07278699,-
0.06493395,-0.00005280,-0.00000579,0.00000127,0.,0.00001381,-0.00004832,-
0.00000002,-0.00001905,0.00002006,0.00000001,-
0.00000139,0.00003675,0.00000002,0.00000349,-0.00000628,0.,-
0.10266039,0.46939449,0.00000005,-0.00000004,-0.00000623,-0.00000002,0.,-
0.00000623,0.00000008,-0.00000010,0.00003306,-
0.00000019,0.00000020,0.00007029,0.00000022,-0.00000003,0.00000757,-
0.00000011,0.,-0.00000812,0.00000037,0.00000025,0.00008173,-
0.00000043,0.00000121,-0.00077753,0.00000132,-
0.00000292,0.00089912,0.00001338,0.00000482,0.00863838,0.00000165,-
0.00000351,0.00156609,0.00000113,0.00000077,-0.00025542,0.00000018,-
0.00000005,-0.00017219,-0.00000007,0.00000002,-0.00001159,0.00000166,-
0.00000028,0.00042712,0.00000080,0.00000006,0.00013941,0.00000002,0.000000
03,0.00000309,0.00000002,0.00000002,0.00000321,-0.00000002,-
0.00000002,0.00000509,0.,-0.00000001,0.00000323,0.00000002,0.00000002,-
0.00000063,-0.00000005,0.00000002,0.00000150,0.,0.,-0.00000298,0.00000003,-
0.00000002,-0.00000204,0.,-0.00000002,-0.00000740,0.,-0.00000001,-
0.00000362,0.00000002,0.00000004,0.00000175,0.,0.00000003,0.00000878,-
0.00000003,0.,0.00000550,0.00000002,-0.00000002,-0.00000096,-
0.00000001,0.,0.00000216,-0.00000003,0.00000001,0.00000282,0.,0.,-
0.00000327,0.00000003,-0.00000002,-0.00000234,0.00000001,0.,-
0.00001380,0.,0.,-0.00000672,-0.00000002,0.00000002,-0.00000796,0.,-
0.00000001,0.00001005,0.00000002,-0.00000003,0.00000680,-0.00000948,-
0.00005340,-0.06105052,-0.00004267,-0.00005405,0.02874922,0.,0.00000002,-
0.00000581,-0.00000005,-0.00000005,-
0.00000546,0.,0.00000001,0.00000485,0.00000003,0.00000004,0.00000680,0.,0.,-
0.00000495,-0.00010379,0.00027745,0.03812291,0.00012683,-0.00010891,-
0.00000006,-0.00014250,0.00028194,0.00000008,0.00024436,-0.00036228,-
0.00000013,-0.00054921,0.00039596,0.00000026,0.00039675,-0.00009230,-
0.00000015,-0.00026956,0.00005812,0.00000009,0.00033535,-0.00026493,-
0.00000017,0.00023705,0.00019124,0.00000012,0.00065465,-0.00101226,-
0.00000065,-0.00328067,-0.00592141,-0.00000306,-
0.00084440,0.00147415,0.00000094,-0.00043563,-0.00058807,-0.00000032,-
0.00008165,0.00011305,0.00000008,-0.00000504,-0.00001562,-
0.00000001,0.00018153,0.00043522,0.00000022,-0.00022349,-
0.00008565,0.,0.00004454,0.00000562,0.,0.00000645,0.00002217,0.,0.00000181,-
0.00000111,0.,0.00000172,-0.00001034,0.,0.00004925,0.00002671,0.,-0.00000373,-
0.00000103,0.,-0.00001191,0.00000047,0.,0.00000288,-0.00000087,0.,-
0.00001026,-0.00001293,0.,-0.00000355,0.00000277,0.,0.00001211,-0.00005167,-
0.00000001,0.00000478,-0.00000863,0.,0.00001432,0.00000771,0.,-0.00000080,-

0.00006764,-0.00000002,-0.00000512,0.00000219,0.,-0.00002155,-0.00001366,0.,-
0.00000631,0.00001304,0.,0.00000303,-0.00000244,0.,0.00001078,0.00002228,0.,-
0.00000163,-0.00000039,0.,0.00000742,0.00015310,0.00000004,-0.00003138,-
0.00007345,-0.00000001,0.00002852,-0.00005658,-0.00000002,-
0.05005042,0.03413250,0.00002550,0.01068358,0.00227700,0.00000007,-
0.00000583,0.00001055,0.,-0.00011808,-
0.00006092,0.00000002,0.00007076,0.00000833,-
0.00000002,0.00003094,0.00004492,0.,-0.00000661,-0.00001031,0.,-
0.26001576,0.18731389,0.00013589,0.30297569,0.00000788,-0.00003420,0.,-
0.00001813,0.00006515,0.00000003,0.00004036,-0.00008046,-0.00000004,-
0.00007185,0.00008635,0.00000006,0.00005855,-0.00002042,0.,-
0.00001639,0.00002374,0.,0.00006733,-0.00018595,-
0.00000012,0.00017439,0.00014753,0.,-0.00005783,-0.00045045,0.,-0.00349938,-
0.00190885,-0.00000422,-0.00037124,0.00058350,0.00000011,-
0.00023622,0.00003566,0.00000033,-
0.00004757,0.00003002,0.00000005,0.00001031,-0.00001021,-
0.00000002,0.00019862,0.00051466,0.00000038,-0.00005376,0.00002381,-
0.00000010,0.00000400,-0.00000088,0.,0.00000253,0.00000241,0.,-
0.00000390,0.00000003,0.,0.00000309,0.00000049,0.,0.00000119,0.00000164,0.,-
0.00000082,-0.00000160,0.,-0.00000027,-
0.00000039,0.,0.00000052,0.00000014,0.,-0.00000038,0.00000056,0.,-0.00000153,-
0.00000046,0.,0.00000346,-0.00001089,0.,0.00000259,-
0.00000786,0.,0.00000443,0.00000630,0.,0.00000064,-0.00001505,0.,-0.00000304,-
0.00000203,0.,-0.00000606,0.00000019,0.,-0.00000199,0.00000280,0.,0.00000037,-
0.00000040,0.,0.00000361,0.00000773,0.,0.00000058,-
0.00000276,0.,0.00001681,0.00002518,0.,-0.00001542,-0.00001402,0.,-
0.00000462,-0.00000464,0.,-0.01805710,0.02346247,0.00001300,0.00222166,-
0.00533594,0.00000066,0.00000132,0.00000035,0.,-
0.00001191,0.00000580,0.,0.00000915,-0.00000392,0.,0.00000203,-0.00000467,0.,-
0.00000079,0.00000090,0.,0.23781425,-0.25938189,-0.00017046,-
0.21816948,0.24245054,0.00000002,0.00000003,-0.00001877,-
0.00000004,0.00000003,0.00001754,0.00000002,0.,-0.00002001,-
0.00000001,0.00000003,-0.00000461,0.00000001,0.,-0.00002520,-0.00000001,-
0.00000002,0.00003437,0.00000006,-0.00000008,-
0.00000651,0.00000006,0.,0.00015928,-0.00000011,0.00000002,-0.00045748,-
0.00000142,-0.00000416,0.00539442,-0.00000022,0.00000007,0.00045887,-
0.00000017,0.00000023,-0.00042703,-0.00000003,0.00000004,-0.00005080,0.,-
0.00000001,0.00002932,0.00000010,0.00000041,-0.00023247,0.,-
0.00000009,0.00017624,0.00000001,-0.00000002,-0.00000027,-0.00000001,0.,-
0.00000207,0.00000002,0.00000001,-0.00000403,0.,0.,-
0.00000256,0.,0.,0.00000041,0.00000002,0.,-0.00000143,0.,0.,0.00000226,-
0.00000002,0.,0.00000158,-0.00000001,0.,0.00000552,0.,0.,0.00000316,0.,-
0.00000003,-0.00000111,0.,0.,-0.00000539,0.00000002,0.,-0.00000346,-
0.00000001,0.,0.00000058,0.,0.,-0.00000165,0.00000002,0.,-
0.00000207,0.,0.,0.00000233,-
0.00000001,0.,0.00000175,0.,0.,0.00000811,0.,0.,0.00000434,0.,0.,0.00000568,0.,0.
,-0.00000708,0.,0.,-0.00000494,-
0.00000766,0.00000940,0.00460345,0.00000009,0.00000055,-
0.00657486,0.,0.,0.00000425,0.,0.,0.00000460,0.,0.,-0.00000402,0.,0.,-
0.00000583,0.,0.,0.00000427,0.00016793,-0.00016675,-0.01644473,-
0.00015866,0.00016036,0.01338606\\-0.00004672,0.00004302,-

0.00000081,0.00008463,-0.00002983,-0.00000058,-0.00001776,0.00000692,-
0.00000090,-0.00001911,-0.00001622,0.00000456,-0.00004706,0.00001581,-
0.00000168,0.00000010,-0.00001073,0.00000375,0.00005216,0.00001776,-
0.00000260,0.00007686,-0.00003255,0.,0.00001262,0.00001439,0.00000048,-
0.00006389,-0.00002589,0.00000043,-0.00006741,0.00003664,0.00000029,-
0.00001838,-0.00001027,-0.00000091,-0.00002190,0.00001204,0.00000054,-
0.00003098,-0.00000558,0.00000018,0.00001719,-
0.00001763,0.00000017,0.00002672,0.00000670,-0.00000014,0.00000039,-
0.00002213,-0.00000167,0.00005539,-0.00004358,0.00000023,0.00001476,-
0.00000225,-0.00000061,-
0.00006018,0.00004409,0.00000082,0.00001565,0.00002186,-0.00000060,-
0.00000924,0.00001076,-0.00000059,0.00001851,0.00000030,-0.00000023,-
0.00000273,-0.00000195,0.00000047,-0.00001248,-0.00001121,0.00000025,-
0.00001886,-0.00000060,-0.00000026,-0.00001040,0.00005847,0.00000026,-
0.00000879,-0.00000477,-0.00000014,0.00005071,0.00000827,-
0.00000016,0.00003468,-0.00003969,-0.00000092,0.00001917,0.00001209,-
0.00000011,-0.00007143,0.00000342,0.00000040,0.00004538,-0.00003481,-
0.00000053,-0.00001756,-0.00000938,-
0.00000011,0.00001399,0.00001188,0.00000014,-0.00000978,-0.00000392,-
0.00000025,-0.00000333,-0.00001219,0.00000363,-0.00002659,0.00001497,-
0.00000160,0.00001000,0.00002067,-0.00000060,0.00007494,-0.00000592,-
0.00000263,-0.00000588,-0.00000225,0.00000091,0.00000612,-0.00001053,-
0.00000030,-0.00003242,-0.00002074,0.00000122,-0.00000529,-0.00000465,-
0.00000047,0.00003165,-0.00000130,-0.00000029,-0.00000233,-0.00000542,-
0.00000006,-0.00004189,0.00002520,0.00000114,0.00001079,0.00000074,-
0.00000013\\\@

[1] Fukaya, Y.; Mihara, Y.; Morizono, D.; Ohtake, Y.; Oishi, T.; Shoji, T.; Takashima Y. (Wakamoto Pharma), *patent application*, **2006**, WO2006051851 A1.

[2] Bhat, L.; Mohapatra, P. P.; Bhat S. R. (Reviva Pharmaceuticals), *patent application*, **2008**, US20080293736 A1.

[3] Li, X.; Longenecker, K. L.; Pei, Z.; Sham, H. L.; Wiedeman P. E. (Abbott Laboratories), *patent application*, **2005**, WO2005023762 A1.

[4] Burrows, A. D.; Frost, C. G.; Mahon, M. F.; Richardson, C. *Angew. Chem.* **2008**, *120*, 8610-8614; *Angew. Chem. Int. Ed.* **2008**, *47*, 8482–8416.

[5] Failli, A.; Quagliato, D.; Andrae, P.; Heffernan, G.; Coghlan, R.; Shen, E. (Wyeth), *patent application*, **2006**, US20060199806 A1.

[6] Gaussian 09, Revision D.01, M. J. Frisch, G. W. Trucks, H. B. Schlegel, G. E. Scuseria, M. A. Robb, J. R. Cheeseman, G. Scalmani, V. Barone, B. Mennucci, G. A. Petersson, H. Nakatsuji, M. Caricato, X. Li, H. P. Hratchian, A. F. Izmaylov, J. Bloino, G. Zheng, J. L. Sonnenberg, M. Hada, M. Ehara, K. Toyota, R. Fukuda, J. Hasegawa, M. Ishida, T. Nakajima, Y. Honda, O. Kitao, H. Nakai, T. Vreven, J. A. Montgomery, Jr., J. E. Peralta, F. Ogliaro, M. Bearpark, J. J. Heyd, E. Brothers, K. N. Kudin, V. N. Staroverov, T. Keith, R. Kobayashi, J. Normand, K. Raghavachari, A. Rendell, J. C. Burant, S. S. Iyengar, J. Tomasi, M. Cossi, N. Rega, J. M. Millam, M.

Klene, J. E. Knox, J. B. Cross, V. Bakken, C. Adamo, J. Jaramillo, R. Gomperts, R. E. Stratmann, O. Yazyev, A. J. Austin, R. Cammi, C. Pomelli, J. W. Ochterski, R. L. Martin, K. Morokuma, V. G. Zakrzewski, G. A. Voth, P. Salvador, J. J. Dannenberg, S. Dapprich, A. D. Daniels, O. Farkas, J. B. Foresman, J. V. Ortiz, J. Cioslowski, D. J. Fox, Gaussian, Inc., Wallingford CT, 2013.
[7] Gaussian 09, Revision D.01, M. J. Frisch, G. W. Trucks, H. B. Schlegel, G. E. Scuseria, M. A. Robb, J. R. Cheeseman, G. Scalmani, V. Barone, B. Mennucci, G. A. Petersson, H. Nakatsuji, M. Caricato, X. Li, H. P. Hratchian, A. F. Izmaylov, J. Bloino, G. Zheng, J. L. Sonnenberg, M. Hada, M. Ehara, K. Toyota, R. Fukuda, J. Hasegawa, M. Ishida, T. Nakajima, Y. Honda, O. Kitao, H. Nakai, T. Vreven, J. A. Montgomery, Jr., J. E. Peralta, F. Ogliaro, M. Bearpark, J. J. Heyd, E. Brothers, K. N. Kudin, V. N. Staroverov, T. Keith, R. Kobayashi, J. Normand, K. Raghavachari, A. Rendell, J. C. Burant, S. S. Iyengar, J. Tomasi, M. Cossi, N. Rega, J. M. Millam, M. Klene, J. E. Knox, J. B. Cross, V. Bakken, C. Adamo, J. Jaramillo, R. Gomperts, R. E. Stratmann, O. Yazyev, A. J. Austin, R. Cammi, C. Pomelli, J. W. Ochterski, R. L. Martin, K. Morokuma, V. G. Zakrzewski, G. A. Voth, P. Salvador, J. J. Dannenberg, S. Dapprich, A. D. Daniels, O. Farkas, J. B. Foresman, J. V. Ortiz, J. Cioslowski, D. J. Fox, Gaussian, Inc., Wallingford CT, 2013.
